# Temporal Network Based Analysis of Cell Specific Vein Graft Transcriptome Defines Key Pathways and Hub Genes in Implantation Injury

**DOI:** 10.1371/journal.pone.0039123

**Published:** 2012-06-15

**Authors:** Manoj Bhasin, Zhen Huang, Leena Pradhan-Nabzdyk, Junaid Y. Malek, Philip J. LoGerfo, Mauricio Contreras, Patrick Guthrie, Eva Csizmadia, Nicholas Andersen, Olivier Kocher, Christiane Ferran, Frank W. LoGerfo

**Affiliations:** 1 Division of Vascular and Endovascular Surgery, Department of Surgery, Beth Israel Deaconess Medical Center, Harvard Medical School, Boston, Massachusetts, United States of America; 2 Genomics and Proteomics Center, Division of Interdisciplinary Medicine and Biotechnology, Beth Israel Deaconess Medical Center, Harvard Medical School, Boston, Massachusetts, United States of America; 3 Deptartment of Pathology, Beth Israel Deaconess Medical Center, Harvard Medical School, Boston, Massachusetts, United States of America; 4 Center for Vascular Biology Research, Beth Israel Deaconess Medical Center, Harvard Medical School, Boston, Massachusetts, United States of America; 5 Center for Vascular Biology Research and Division of Nephrology, Department of Medicine, Beth Israel Deaconess Medical Center, Harvard Medical School, Boston, Massachusetts, United States of America; University of Jaén, Spain

## Abstract

Vein graft failure occurs between 1 and 6 months after implantation due to obstructive intimal hyperplasia, related in part to implantation injury. The cell-specific and temporal response of the transcriptome to vein graft implantation injury was determined by transcriptional profiling of laser capture microdissected endothelial cells (EC) and medial smooth muscle cells (SMC) from canine vein grafts, 2 hours (H) to 30 days (D) following surgery. Our results demonstrate a robust genomic response beginning at 2 H, peaking at 12–24 H, declining by 7 D, and resolving by 30 D. Gene ontology and pathway analyses of differentially expressed genes indicated that implantation injury affects inflammatory and immune responses, apoptosis, mitosis, and extracellular matrix reorganization in both cell types. Through backpropagation an integrated network was built, starting with genes differentially expressed at 30 D, followed by adding upstream interactive genes from each prior time-point. This identified significant enrichment of IL-6, IL-8, NF-κB, dendritic cell maturation, glucocorticoid receptor, and Triggering Receptor Expressed on Myeloid Cells (TREM-1) signaling, as well as PPARα activation pathways in graft EC and SMC. Interactive network-based analyses identified IL-6, IL-8, IL-1α, and Insulin Receptor (INSR) as focus hub genes within these pathways. Real-time PCR was used for the validation of two of these genes: IL-6 and IL-8, in addition to Collagen 11A1 (COL11A1), a cornerstone of the backpropagation. In conclusion, these results establish causality relationships clarifying the pathogenesis of vein graft implantation injury, and identifying novel targets for its prevention.

## Introduction

Surgical bypass grafting using autologous vein conduits is the cornerstone therapy for coronary and peripheral arterial occlusive disease. About 250,000 coronary artery bypass grafts (CABG) and about 80,000 lower extremity vein graft implantations are performed each year with an average cost of 44 billion dollars [1]–[3]. More than 50% of CABG fail within 10 years, and 30–50% of lower extremity vein grafts fail within 5 years from surgery [4]. Vein bypass graft failure is classified into three distinct phases: early (less than 30 days), mid-term (3 to 24 months) and late (greater than 2 years) [5]. Mid-term failure due to intimal hyperplasia (IH) causing stenosis and ultimately occlusion is by far the most common cause (>70%) of vein graft failure [6]. These numbers beg better understanding of the molecular basis of these lesions, in order to define targeted therapies that would reduce failure rate.

Although some pharmacological therapies such as Aspirin and dipyridamole, as well as statins have shown modest benefit in improving CABG outcome [7]–[10], there has been no corresponding benefit for lower extremity vein grafts [11]. A more recent mechanistically oriented clinical trial, Project of Ex-Vivo vein graft Engineering via Transfection (PREVENT-III), employing *ex vivo* treatment of lower extremity vein grafts with a decoy of cell cycle transcription factor, E2F, during the surgical procedure was also ineffective in improving outcome [12].

Trauma to the vein graft at the time of implantation and subsequent exposure to a new environment of arterial hemodynamics [13], [14] are considered two major pathogenic factors involved in delayed graft failure. In response to this implantation injury the vein graft wall undergoes an obligatory remodeling, which if exaggerated, may result in IH, stenosis, and thrombosis [15]–[17].

Using transcriptional profiling of canine vein bypass grafts, our laboratory has already identified critical transcriptome responses to implantation injury [18]. However, the findings of these previous studies were limited by the unavailability of a canine specific gene array, and the inability to examine the individual contributions of endothelial (EC) and smooth muscle cell (SMC) layers to the altered transcriptome.

The principal hypothesis of our present study is that implantation injury causes temporal genetic changes in EC and SMC of vein grafts, triggering a cascade of interrelated molecular events causing vessel wall remodeling and IH. Accordingly, we performed transcriptional profiling of EC and SMC after their retrieval by laser capture microdissection (LCM) from canine vein grafts, a clinically relevant large animal model, at time-points ranging 2 hours (H) to 30 days (D) following the surgery. Backpropagation analysis of transcriptional profile helped in ascribing the time dependent genomic alterations to a specific vessel layer/cell type, and in identifying most significantly affected pathways, as well as gene-interaction focus hubs critically involved in implantation injury. This allowed us to establish a vein graft implantation injury signature, and to identify causality relationships that clarify its pathogenesis, laying the foundation for strategies to prevent or treat it.

## Results

### Purity of EC and SMC isolated by LCM

Purity of EC and medial SMC retrieved by laser capture microdissection (LCM) from control veins and vein grafts was determined by Q-RT-PCR using the cell-specific markers, Platelet Endothelial Cell Adhesion Molecule-1 (PECAM-1/CD31) for EC and Myosin Heavy Chain II (MHCII) for SMC. Gene expression of CD31 was lower in control SMC as compared to control EC (range of RQ=0.009±0.004 to 0.2±0.2), and in graft SMC as compared to graft EC (range of RQ=0.06±0.009 to 0.24±0.019) at all time-points, suggesting that there was negligible contamination of SMC with EC (Figure S1A). Similarly, MHCII expression was lower in control EC as compared to control SMC (range of RQ=0.22±0.05 to 0.4±0.1), and in graft EC as compared to graft SMC (range of RQ=0.16±0.07 to 0.4±0.14) at all time-points, suggesting that there was negligible contamination of EC with SMC (Figure S1B). Altogether, these results indicate that our LCM samples were enriched by 80–99% for SMC, and by 60–80% for EC.

Vein graft immunostaining for CD3 and CD18 showed almost no positive cells on vascular graft sections retrieved at 12 and 24 H, while few CD3 and some CD18 positive cells were noted within vein grafts media at 7 D, and adventitia at 30 D (Figure S2A & S2B). Accordingly, transcriptional changes observed in vein graft LCM samples were mostly representative of EC or SMC transcriptomes.

### Microarray quality control

The array data was determined to be of high quality as assessed by the scaling factor, average background, percent present calls, and 3′-5′ RNA ratio. In addition, dChip software for outlier analysis did not identify any outlier array using the default criteria.

### Implantation injury leads to time-dependent qualitative and quantitative changes in the transcriptome of graft EC and SMC

**Figure 1:**
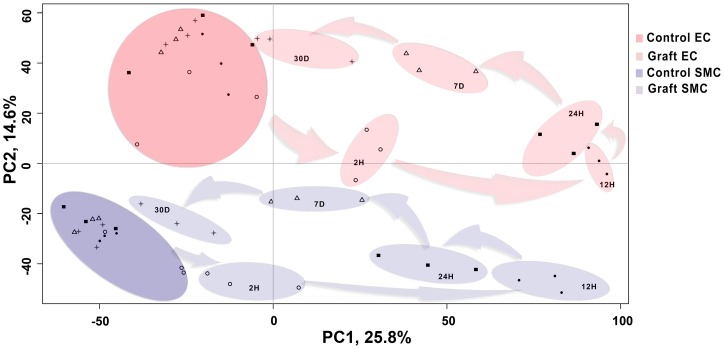
Principal Component Analysis(PCA) of the temporal expression data from control vein and vein graft. The preprocessed transcriptional data from the control vein and vein graft endothelial cells (EC) as well as smooth muscle cells (SMC) is plotted along the top two components from the PCA. The first component with highest variance (25.8%) is shown on the X-axis and second highest (14.6%) is displayed on the Y-axis. On the basis of these components, the data can be differentiated in four major clusters i.e. control EC (dark red), control SMC (dark blue), graft EC (light red) and graft SMC (light blue). Each cluster further consists of sub-clusters representing time dependent segregation. Each time-point is represented with unique symbols (2 H =♦, 12 H=•, 24 H=▪, 7 D=Δ and 30 D=*). In the plot, the distance between the samples is proportional to correlation at the transcriptional profile level. For example, control and graft sample clusters from both EC and SMC have minimum correlation at 12 H (maximum distance) and maximum correlation at 30 D (minimum distance).

Principal Component Analysis (PCA) of pre-processed microarray data demonstrated that samples separated on the basis of graft vs. control along Principal Component 1 (PC1), which accounts for 25.8% of the variance; and on the basis of cell type (EC vs. SMC) along PC2, which accounts for 14.3% of the variance (Figure 1). This demonstrates that transcriptional differences were greater between grafts and controls as compared to transcriptional differences between cell types. Transcription profiles of graft EC and SMC clustered temporally, and followed a counter clockwise pattern, with 12 and 24 H graft samples being most distant, and 7 and 30 D graft samples being less distant from their corresponding controls (Figure 1). Unsupervised hierarchical clustering depicted more transcriptional differences between control vs. graft than between cell types at 12 H, 24 H and 7 D. Clustering also depicted less transcriptional differences between control vs. graft than between cell types at 2 H and 30 D consistent with PCA results (Figure 1, & Figure S3). This suggests that injury at the time of implantation triggers a potent acute response, manifesting in early robust qualitative and quantitative changes in gene transcription that resolves over time. Transcription profiles of control EC and SMC clustered by cell type regardless of time-points.

### Characterization of the vein graft acute and sustained genetic response to implantation injury

Using a bioconductor package [19], [20] for statistical linear model of microarray data, (LIMMA), we determined that a total of 3,651 EC genes and 4,299 SMC genes were differentially expressed at the five time-points. All these genes achieved a false discovery rate (FDR) adjusted *p-value* <0.05, and an absolute fold change between control and graft veins ≥2. To focus on evolutionarily conserved transcripts, canine transcripts were mapped to human orthologues, as determined by Affymetrix array comparison database. A summary of the differentially expressed genes at each time-point in EC and SMC is provided in Table 1.

**Table 1:** Differentially expressed genes in EC and SMC with valid gene symbols and human orthologous genes.

Cell Type	EC					SMC				
	2H	12H	24H	7D	30D	2H	12H	24H	7D	30D
**Down-regulated**	↓32	↓956	↓535	↓387	↓4	↓1	↓1353	↓299	↓604	↓3
**Up-regulated**	↑197	↑787	↑605	↑748	↑41	↑27	↑1304	↑309	↑897	↑19
**Total**	229	1743	1140	1135	45	28	2657	608	1501	22

In both EC and SMC, the number of differentially expressed genes peaked at 12 H through 7 D, and substantially decreased by 30 D. Interestingly, at 2 H, a considerably higher number of genes were differentially expressed in EC as compared to SMC (229 vs. 28), likely reflecting EC being the first physical target of implantation injury. The most robust response was noted between 12 H and 7 D, with differentially expressed genes ranging 608 to 2,657. In contrast, only 45 (∼1%) and 22 (0.5%) genes were differentially expressed at 30 D in EC and SMC, respectively. Those included, components of the extra cellular matrix such as major collagens and integrins, both indicators of healing. Remarkably, at 12 H, 43% of differentially expressed genes (up and down-regulated) were common in graft EC and SMC. Those genes were mainly inflammatory and immune-regulated genes, which indicated the central role of acute inflammatory processes in driving vascular remodeling, associated with implantation injury. Details of EC's and SMC's unique and shared up- and down-regulated genes at all time-points are provided in Figures S4A & S4B, and Table S2. Top differentially expressed genes from all time-points, based on absolute fold-change, are listed in Table 2.

**Table 2:** List of selected differentially expressed genes in graft EC and SMC.

Gene SYMBOL	Graft EC					Graft SMC				
	2H	12H	24H	7D	30D	2H	12H	24H	7D	30D
**CDKN1A**	1.79	1.60	1.65	1.44	2.41		2.77	2.33	1.34	
**CLEC5A**		4.07	3.94	2.25	3.15		4.37	2.64	3.41	3.66
**LAPTM5**	2.51	3.44	4.17	4.71	3.39		3.35	2.64	3.23	
**TFPI2**		5.19	5.70	6.44	4.66		6.46	7.57	7.14	5.79
**LYZ**	1.84	1.99	2.63	2.22			4.47	2.57	2.83	2.39
**TMEM49**	1.64	1.43	2.11	1.64			2.79	2.97	3.46	2.85
**BIRC3**	2.94	1.33	1.24	2.50		2.61	1.35	1.65	1.31	
**SERPINE1**	4.07	2.41	2.32	2.43		3.14	2.63	3.58	2.76	
**SPP1**		3.22	4.01	6.38	2.32		3.14	3.88	3.94	
**VCAN**		2.01	3.07	2.92	2.87		3.18	2.60	3.60	
**ALOX5AP**		2.90	3.36	3.09	3.19		2.96	2.30	2.27	
**IL18**	2.01	3.79	4.21	2.40	3.46		3.20		1.91	
**EGR1**	2.20	1.81	1.26		3.59		2.58	1.90		4.90
**CLEC12A**	3.63	2.50	2.79	3.42	4.44		1.73		1.13	
**SERPINE2**		1.37	2.77	2.56	5.20		2.70	2.07	2.33	
**AIM1**	1.66	1.67	2.50	2.43			1.72	1.83	1.97	
**ARHGAP9**	1.94	3.58	3.52	1.83			3.18	2.40	3.19	
**CD44**	3.77	1.54	2.51	1.62			1.67	1.44	1.09	
**FYB**	3.84	4.17	4.01	3.50			4.28	3.03	1.98	
**GK**	2.49	3.16	3.58	2.59			3.71	2.09	1.38	
**GMFG**	2.37	2.50	2.79	2.04			2.57	2.52	2.49	
**IL-8**	5.88	5.71	5.69	1.53			7.30	6.28	1.46	
**KMO**	2.80	1.87	2.04	2.11			3.01	2.06	1.47	
**LCP1**	2.25	3.57	4.38	4.43			3.47	2.38	2.56	
**MTHFD2**	1.58	2.01	1.69	1.48			1.74	1.66	1.39	
**NCKAP1L**	2.92	1.31	3.89	3.67			1.87	2.88	2.42	
**NRG1**	2.22	2.81	2.96	2.45			2.00	1.68	1.22	
**PLAUR**	2.50	3.65	2.73	1.57			3.73	4.98	2.92	
**FCGR1A**		4.35	5.55	3.77			4.40	3.63	3.94	2.89
**SLC22A1**		−2.10	−3.04	−2.65			−1.29	−2.83	−3.38	−2.28
**ENPP2**				−2.86	−3.52		−1.76	−3.41	−2.49	−2.63
**BTC**		−2.28	−3.68		−3.27		−2.58	−3.14	−3.07	
**HPSE2**		−3.06	−2.81		−2.45		−3.30	−2.56	−1.38	
**CCDC88C**	−1.83	−2.05	−1.58	−2.02			−2.12		−1.53	
**ADHFE1**		−2.01	−1.70	−1.15			−1.70	−1.52	−1.11	
**AGPHD1**		−2.67	−2.90	−1.44			−3.86	−2.55	−1.54	
**AKAP6**		−3.41	−2.32	−2.51			−2.69	−2.59	−1.60	
**ALDH7A1**		−1.86	−2.05	−1.46			−1.95	−1.38	−1.51	
**AMIGO2**		−3.87	−3.43	−3.58			−4.95	−3.69	−3.31	
**ANGPTL1**		−4.23	−4.03	−2.57			−3.67	−2.87	−1.88	
**ARMC4**		−2.24	−2.32	−1.99			−2.42	−2.43	−1.39	
**CALCOCO1**		−2.71	−2.68	−1.61			−3.30	−3.49	−1.69	
**CCDC3**		−1.77	−2.19	−3.22			−1.43	−1.72	−1.60	
**CHN1**		−2.19	−1.77	−1.29			−3.35	−1.99	−1.34	
**CKM**		−3.39	−3.96	−3.13			−3.48	−3.51	−1.45	
**COCH**		−1.65	−3.11	−3.74			−1.81	−2.60	−2.93	
**COL14A1**		−2.56	−3.85	−3.34			−2.18	−2.00	−1.70	

**Figure 2:**
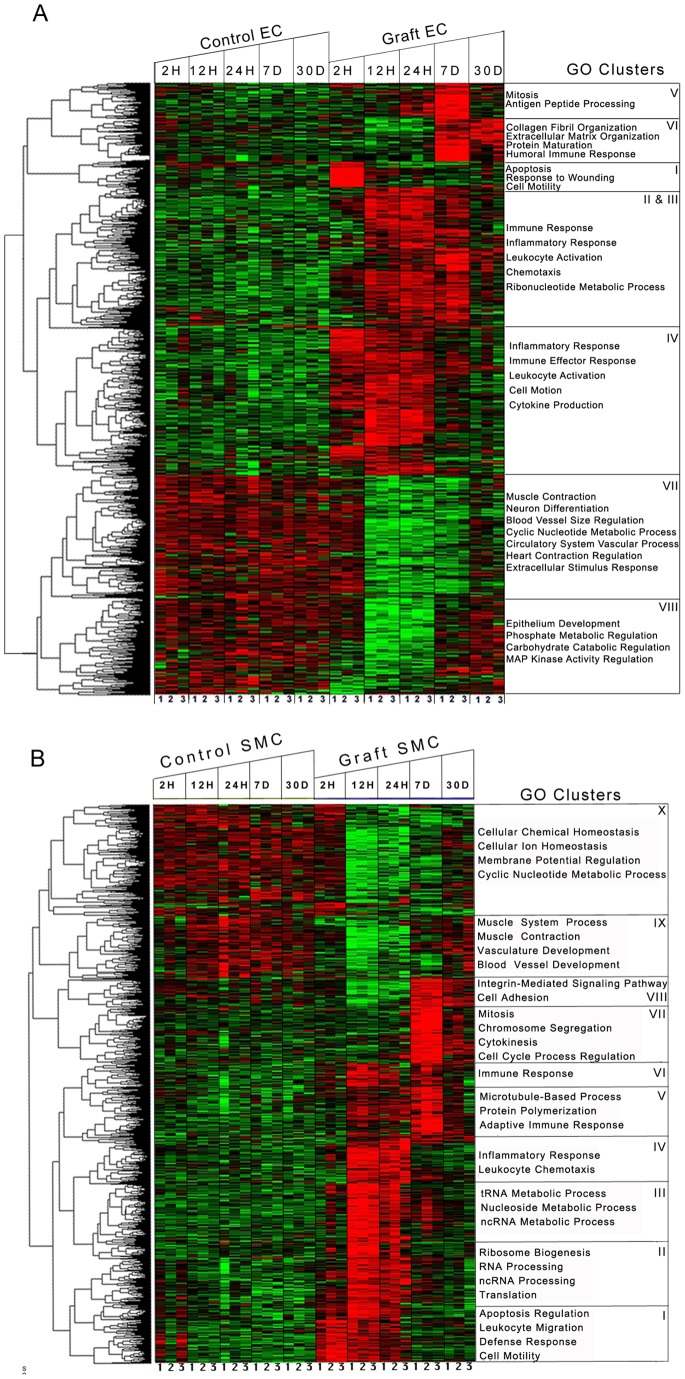
Time series analysis of differentially expressed genes following vein graft implantation. A) EC, B) SMC: The columns represent samples and the rows represent genes. Gene expression is shown with a pseudocolor scale (−1 to 1) with red color denoting increase and green color denoting decrease in gene expression. The heatmaps depict the differential gene expression patterns of endothelial cells (EC) and smooth muscle cells (SMC) that are clustered using K means. The gene ontology categories enriched in each K means cluster are represented with heatmaps. The detailed patterns of gene expression are provided for EC and SMC in Figures S3A & S3B respectively.

Additionally, we performed time-series analysis, using the improved empirical ‘bayes’ approach, which considers correlations within samples and between time-points. We identified 1,850 (3,748 probes) and 1,851 (3,750 probes) significantly modified genes in graft EC and SMC, respectively, at *p-value* <0.01. From these genes, 1,525 EC genes (82% of 1,850) were identified, by both time-series and individual time-point analyses (Figure 2A). Using K-means clustering, we partitioned these 1,525 genes into 8 clusters with different expression patterns (Figure S5A), and performed gene ontology (GO) enrichment analysis on each cluster (Figure 2A). Clusters represent a range of expression patterns that may be specific to one particular time-point or span multiple time-points. Cluster I consisted of acute response genes linked to injury response that peaked at 2 H. Clusters II–IV consisted of immune and inflammatory response genes that peaked at 12 and 24 H. Cluster V consisted of genes linked to mitosis and antigen processing and presentation, peaking at 7 D. Cluster VI consisted of genes primarily involved in immune responses and extracellular matrix organization that peaked at 7 and 30 D. Clusters VII and VIII consisted of genes involved in muscle contraction, neuronal differentiation and regulation of mitogen activated protein kinase (MAPK) activity, that were down-regulated at 12, 24 H and 7 D. The detail of genes involved in each enriched GO Biological term is provided in Table S3A.

In SMC, we identified a total of 1,675 genes (90% of 1851), by both time-series and individual time-point analyses (Figure 2B). Similar to EC, we partitioned these 1,675 genes into 10 clusters with different expression patterns (Figure S5B), and performed GO enrichment analysis on each cluster (Figure 2B). Cluster I consisted of genes involved in apoptosis and defense responses that peaked at 2 and 12 H. Clusters II–IV consisted of inflammatory response, leukocyte chemotaxis, nucleotide metabolism and ribosome biogenesis genes, that peaked at 12 and 24 H. Clusters V–VII consisted of genes involved in chromosomal segregation, mitosis and integrin mediated signaling, peaking at 7 D. Cluster VIII consisted of genes involved in integrin-mediated signaling pathway and cell adhesion that peaked at 7 and 30 D. Cluster IX primarily consisted of genes involved in muscle contraction, vasculature and blood vessel development that were down-regulated between 2 and 24 H. Cluster X primarily consisted of genes involved in chemical homeostasis and cyclic nucleotide metabolic processes that were down-regulated from 12 H to 7 D. The detail of genes involved in each enriched GO Biological term is provided in Table S3B.

### Canonical Pathways Enrichment Analysis identifies sequential biological processes driving vein graft implantation injury

We performed pathways enrichment analysis (PEA), using Ingenuity Pathway Analysis (IPA) tools, to determine the relationship between temporal modification of gene expression in graft EC and SMC and cellular biological outcomes. This analysis was based on differentially expressed genes at individual time-points, and enriched pathways with multiple test corrected P value <0.01. This PEA provided useful insight into the pathophysiology of implantation injury

**Figure 3:**
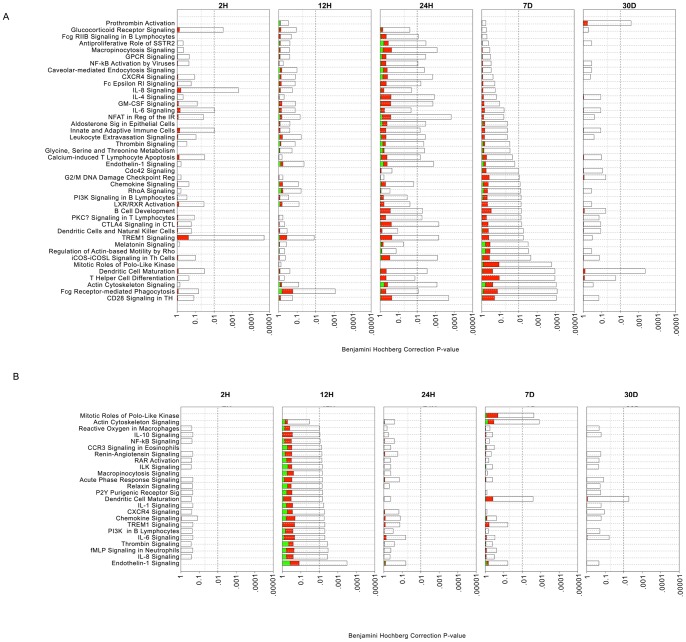
Analysis of canonical pathways enrichment in vein grafts at different time-points. A) **EC, B**) **SMC**: Each panel denotes the effect on canonical pathways at particular time-points after graft implantation. Each bar represents a pathway with significance of enrichment determined using the Benjamini-Hochberg hypothesis corrected *p-value* (shown on primary X-axis). The directionality of the genes in each pathway is depicted using a pseudocolor (red for up-regulated genes, green for down-regulated genes and clear for unmodified genes).

In EC, inflammatory and immune-related pathways were enriched at multiple time-points. These included, interleukin-6 (IL-6) (2 and 24 H), interleukin-8 (IL-8) (2 and 24 H), innate and adaptive immune cell signaling (2 and 24 H and 7 D), as well as dendritic cell maturation (2 and 24 H and 7 and 30 D). In contrast, some pathways were enriched at a single time-point, such as G2/M DNA damage checkpoint regulation (7 D), and prothrombin activation (30 D) (Figure 3A). Sequentially, enrichment of the IL-6, IL-8 and glucocorticoid receptor pro-inflammatory signaling started at 2 H, promoting chemotaxis of immune cells into the vein graft wall. This was followed by enrichment of primary cell mediated immune defense pathways involving macrophage and monocyte mediated phagocytosis at 12 H, with associated reduction of the acute phase response signals, and T cell and B cell differentiation and development by 24 H. Subsequently, inflammatory and immune-mediated damage to the vessel wall triggered enrichment of cell cycle pathways (Mitotic role of Polo kinases, G2/M DNA damage checkpoint regulation) that were sustained up to 7D. This was followed by enrichment of cell repair pathways such as actin/cytoskeleton signaling by 30 D (Figure 3A). Table S4A provides detailed information of genes involved in each significantly enriched pathway.

In SMC, no pathways were significantly enriched at 2 H, reinforcing that EC are first to be impacted by, and respond to implantation injury. However, the12 H time-point showed the highest number of enriched pathways in SMC. Similar to EC, these mostly included inflammatory and immune-related pathways such as IL-10, IL-8, chemokine receptor type 4 (CXCR4), and chemokine and macrophages signaling. As in EC, several pathways were enriched at multiple time-points, such as dendritic cell maturation (12 H, 7 and 30 D), actin cytoskeleton signaling (12 H and 7 D), and endothelin-1 signaling (12 and 24 H and 7 D) (Figure 3B). In contrast, other pathways were enriched at a single time-point, such as chemokine and CXCR4 signaling (12 H), and Mitotic Roles of Polo-like Kinase (7 D). Table S4B provides detailed information of genes involved in each significantly enriched pathway.

In both EC and SMC, inflammatory and immune-related pathways were enriched in an early and sustained manner, highlighting the key role of inflammation in initiation and progression of vein graft implantation injury. In contrast, other pathways such as cell cycle regulation were enriched at later single time-points, which implies a narrower therapeutic window for cell cycle based therapies.

**Figure 4:**
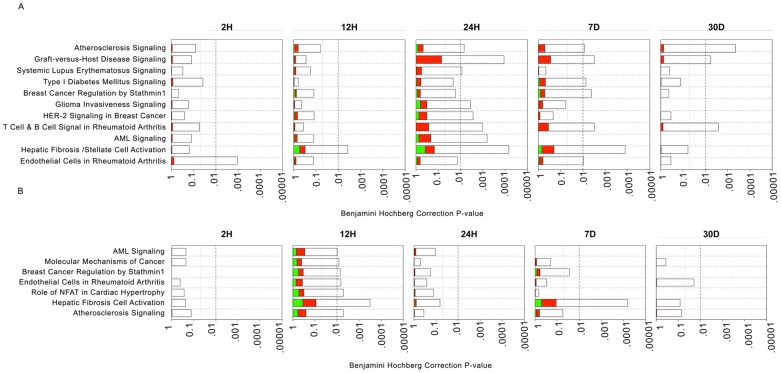
Analysis of disease-related pathways enrichment in vein grafts at different time-points. A ) **EC, B**) **SMC:** Each panel denotes the effect on disease pathways at particular time-points after graft implantation. Each bar represents a pathway with significance of enrichment determined using the Benjamini-Hochberg hypothesis corrected *p-value* (shown on primary X-axis). The directionality of the genes in each pathway is depicted using a pseudocolor (red for up-regulated genes, green for down-regulated genes and clear for unmodified genes).

In addition to cell-specific PEA, we performed disease specific pathways enrichment analysis using IPA, which includes a set of manually curated pathways related to various disease processes ranging from cancer to metabolic diseases. Several disease-specific pathways that are relevant to the pathogenesis of vein graft implantation injury were enriched at different time-points in both cell types, including immune dysfunction related diseases such as rheumatoid arthritis, atherosclerosis, and hepatic fibrosis (Figures 4A & 4B). These results highlight the central role of inflammation and immune dysfunction in the pathogenesis of implantation injury. A list of genes involved in disease specific-pathways is provided in Tables S5A & S5B.

### Backpropagation based interactive network analysis provides insight into key biological pathways linked to implantation injury

**Figure 5:**
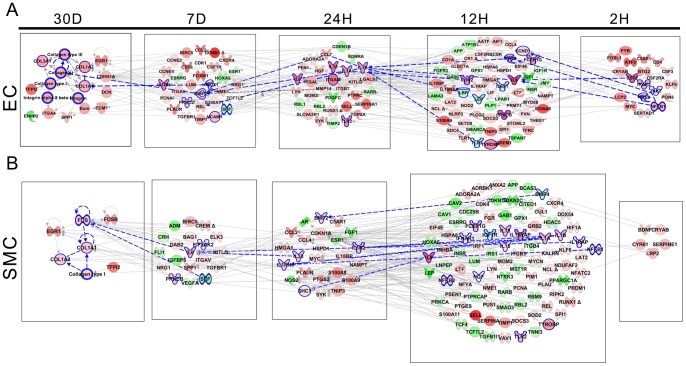
Backpropagation based interactive network analysis of the genes. **A**) **EC, B**) **SMC:** A hierarchical network of interactive genes was developed consisting of differentially expressed genes from 30 D to 2 H. First level of the network was developed from the genes that are significantly differentially expressed at the final time-point (30 D). Second level of the network was built using differentially expressed upstream interactive genes at the prior time-point (7 D). This process was repeated until we reached the starting time-point of 2 H.

To get a mechanistic insight into the pathophysiology of vein graft implantation injury, we combined stage specific transcriptional changes using interactive network analysis. Through a backpropagation approach we generated a multilayered network for each cell type. Accordingly, genes at a given time-point directly interacted with partners at the immediate upstream level, thereby connecting final lesions to initiating events (Figures 5A & 5B).

We then analyzed all genes encompassing all layers of the backpropagation network, using Ingenuity Systems, in a way that selected pathways that affected at least 10% of those genes. This approach identified 6 and 5 dominant pathways in EC and SMC, respectively (Figures S6A & S6B). Remarkably, 4 of these pathways were common to both cell types, and included IL-6 signaling, nuclear factor kappa B (NF-κB) signaling, dendritic cell maturation, and glucocorticoid receptor signaling. Interestingly, most genes within the NFκB pathway were pro-inflammatory and were upregulated at multiple time-points in graft EC and SMC, indicating active and sustained inflammation. The most striking observation within the GC pathway related to down-regulation of the glucocorticoid receptor at multiple time-points, indicating loss of regulatory anti-inflammatory pathways, thereby amplifying inflammatory responses. IL-8 signaling and triggering receptor expressed on myeloid cells 1 (TREM-1) signaling qualified as dominant in EC specifically, while peroxisome proliferator-activated receptor alpha (PPARα signaling, qualified as dominant in SMC specifically.

To get further insight into time specific events, we also analyzed each layer of the backpropagation network (Figures 5A & 5B). In EC, the base 30 D network mainly comprised up-regulated genes associated with cellular assembly and organization (Figure 5A). Significantly interacting genes at 7 D, upstream of the 30 D base layer were enriched for glucocorticoid receptor signaling, cell cycle and IL-8 signaling. At 24 H, genes that significantly interacted with the 7 D layer were enriched for TREM-1 signaling, liver X receptor/retinoid X receptor (LXR-RXR) signaling, and dendritic cell maturation. At 12 H, genes that significantly interacted with the 24 H layer were enriched for TREM-1, NF-κB, and IL-6 signaling. At 2 H, genes that significantly interacted with the 12 H layer were enriched for TREM-1, interleukin-15 (IL-15) and granulocyte macrophage colony stimulating factor (GM-CSF) signaling.

In SMC, the base 30 D network mainly comprised up-regulated genes associated with Prothrombin activation, Cyclin Dependent Kinase 5 (CDK5) signaling, and IL-6 signaling (Figure 5B). Significantly interacting genes at 7 D, upstream of the 30 D base layer were enriched for NF-κB activation, Platelet Derived Growth Factor (PDGF) signaling, and RXR activation. At 24 H, genes that significantly interacted with the 7 D layer were enriched for glucocorticoid receptor, RXR, and PPAR signaling. At 12 H, genes that significantly interacted with the 24 H layer were enriched for TREM-1, PPARα, and NF-κB signaling. At 2 H, there were few genes interacting with the 12 H layer, however we could not link them to a known canonical pathway. This indicated that the integrated response of the SMC started later than that of the EC. This time-specific analysis differs from the previous integrated pathway analysis in that it offers a temporal appreciation of the pathogenic events occurring during implantation injury, while the other allows a global view of the network.

Remarkably, IL-6 and IL-8 signaling pathways spanned all EC layers and 4 consecutive SMC layers, which qualifies them as key to the pathogenesis of vein implantation injury. We propose the following cascade of events, exemplifying the temporal dysregulation of IL-6 and IL-8 signaling pathways. In EC, NF-κB up-regulated at 2 H, is a direct transcriptional activator of IL-8, which is up-regulated at 12 H [21]. In turn, vascular expression of IL-8 is further enhanced by IL-1β and IL-1α that are up-regulated at 24H [22], [23]. IL-1α and β induce MMP2 expression, that is highly up-regulated in the 7 D layers [24]. MMP2 drives the proteolytic processing of collagen, which likely feeds back into up-regulation of collagen genes, namely COL1A1 collagen 1A (COL1A), collagen 1A2 (COL1A2), collagen 3A1 (COL3A1), and Collagen Type I at 30 D.

In SMC, IL-1 α/β, that are up-regulated at 12 H increase the transcription of IL-8 within the same layer (Figure 5B) [23]. This is in turn amplified by increased expression of NF-κB, a direct transcriptional activator of IL-8 [21]. Similarly, IL-1 α/β increase the transcription of IL-6 at 7 D [23], which is also amplified by increased expression of NF-κB, a direct transcriptional activator of IL-6 [25]. Interestingly, IL-1 signaling is modulated by increased expression at 24 H of IL-1 receptor II (IL1-R2), an antagonist of IL-1 signaling [26]. Increased 7 D expression of IL-6 up-regulates the proto-oncogenes Fos (as seen at 30 D) namely c-Fos, through a direct STAT3-dependent-mechanism [27], [28]. In turn c-Fos transcriptionally represses Collagen Type I transcription by binding an AP1 element on the Collagen type I promoter [29], which likely feeds back into up-regulation of other Collagen genes, namely COL1A1 collagen 1A (COL1A), collagen 1A2 (COL1A2), and even Collagen Type I at 30 D, similar to what seen in EC.

### Identification of the signature network of vein graft implantation injury

**Figure 6:**
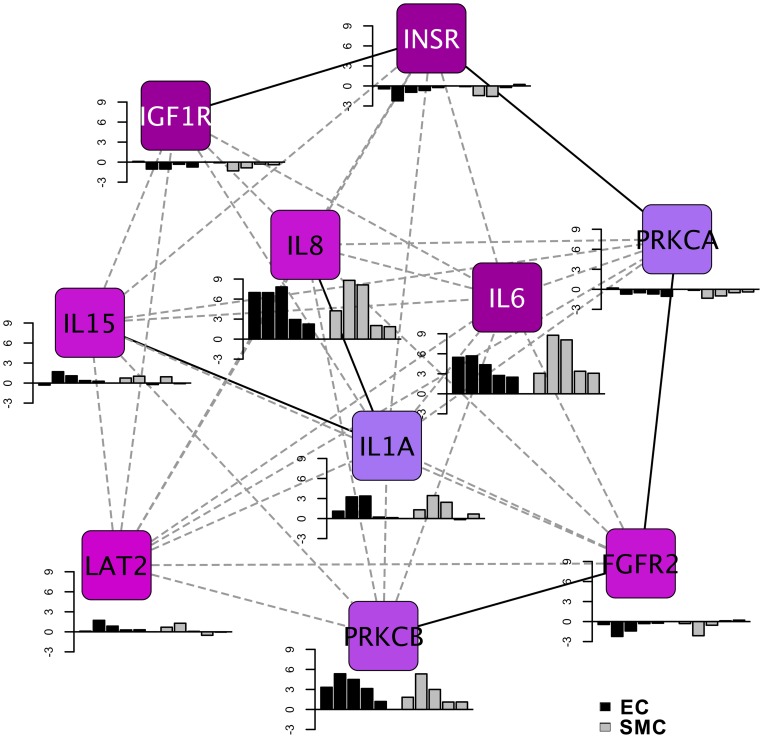
Signature network of vein graft implantation injury. The network represents top ten focal gene hubs identified using density of maximum neighborhood component (DMNC) from backpropagation interaction network. The pseudocolor scale from violet to blue represents the DMNC rank from 1–10. DMNC rank is a level of significance with smaller rank indicating increasing confidence of criticality for network functioning. The bar graphs represent fold change for each focal gene hub in EC and SMC. Fold change of temporal data (2, 12 and 24 H, and 7 and 30 D) of EC and SMC is depicted by black and grey colored bars respectively.

Having established the key interactive networks that are involved in vein graft implantation injury in EC and SMC, we sought to identify a single signature network that would best qualify vein graft implantation injury. This signature network consists of the 10 most interactive focus gene hubs common to EC and SMC, as identified by the density of maximum neighborhood component (DMNC). These genes comprised growth factor receptors such as insulin receptor (INSR), and insulin like growth factor receptor (IGFR), fibroblast growth factor receptor 2 (FGFR2), that were significantly down-regulated, several pro-inflammatory cytokines namely IL-6, IL-8, IL-15 and IL-1A, that were very significantly up-regulated, and signaling molecules such as the serine threonine kinase, the Protein Kinase C beta (PRKCB) that was significantly up-regulated, while the alpha isoform, PRKCA was moderately down-regulated (Figure 6). Importantly, IL-8 and IL-6 were the most up-regulated focus gene hubs at all time-points, as depicted in the histograms in Figure 6. To our knowledge, this is the first systematically defined signature network described for vein graft implantation injury. We propose that it could be used for diagnostic, prognostic and therapeutic purposes.

### Quantitative validation of vein graft implantation injury focus hub genes IL-6 and IL-8

**Figure 7:**
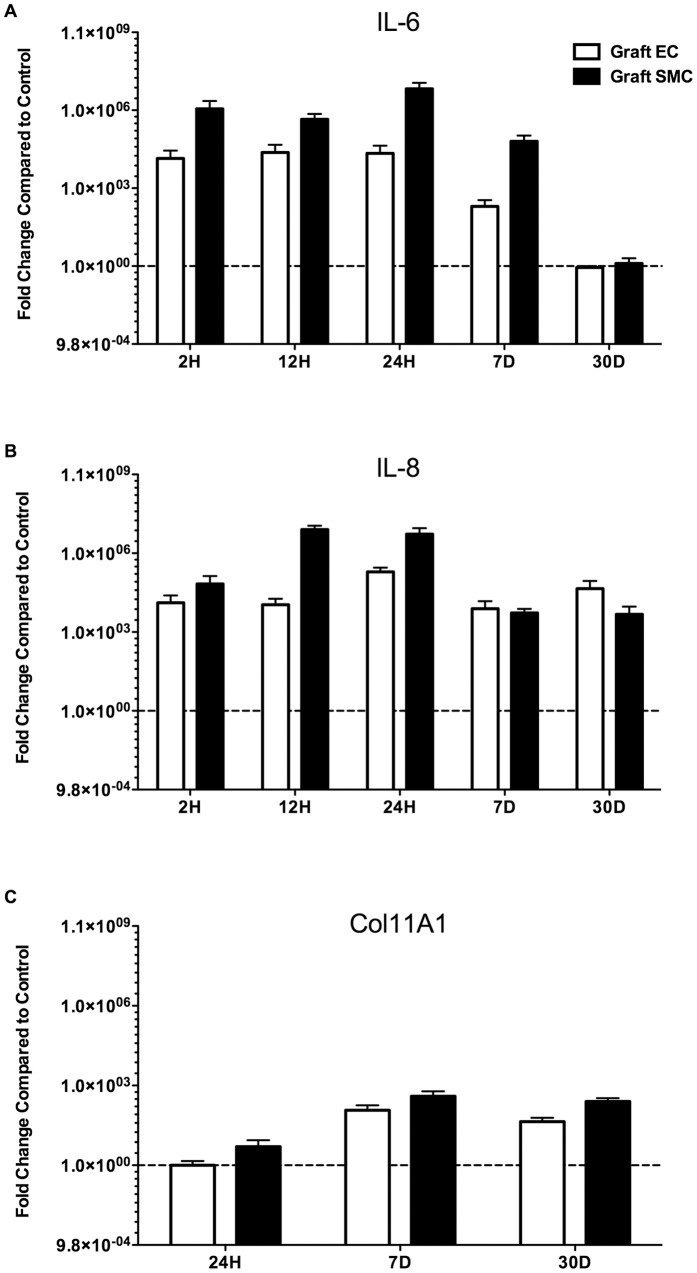
Confirmation of change in gene expression of A) IL-6, B) IL-8 and C) Col11A1. A) Relative gene expression of IL-6 in graft EC and graft SMC compared to control EC and control SMC respectively at 2, 12 and 24 H, and 7 and 30 D, B) Relative gene expression of IL-8 in graft EC and graft SMC compared to control EC and control SMC respectively at 2, 12 and 24 H, and 7 and 30 D and C) Relative gene expression of Collagen11A1 (Col11A1) in graft EC and graft SMC compared to control EC and control SMC respectively at 24 H, and 7 and 30 D.

To validate the most differentially expressed focus hub genes that were identified in the vein graft implantation injury signature network, we analyzed the mRNA expression levels of IL-6 and IL-8 in EC and SMC. IL-6 mRNA levels, analyzed by qRT-PCR were increased by more than 1,000-fold in vein graft EC and SMC at 2, 12, and 24 H, and 7 D, as compared to control vein EC and SMC (Figure 7A). Similarly, IL-8 mRNA levels were increased by more than 1,000-fold in vein graft EC and SMC, at 2, 12, and 24 H, 7 and 30 D, as compared to controls (Figure 7B). This indicated that the vein graft was inundated with local pro-inflammatory cytokines and chemokines, mainly produced by SMC, the most abundant cell type in the vessel wall.

Downstream of IL-6 and IL-8, we analyzed Col11A1 mRNA levels by qRT-PCR. Based on our backpropagation analysis, Col11A1, a critical component of the extracellular matrix was one of the most highly expressed genes later in the process of vein graft implantation injury We were able to detect Col11A1 mRNA levels by 24 H following vein graft implantation. However, ColA11A1 significantly up-regulated in vein graft EC and SMC starting at 7 D and persisted at 30 D after vein graft implantation as compared to control vein EC and SMC (Figure 7C). These data confirm the microarray findings and, in particular those focus hub genes that were identified by interactive Systems Biology Analysis of focus hub identification [30].

## Discussion

Recently, major efforts have been undertaken to decrease the rate of vein graft failure. One such endeavor was the PREVENT-III trial aimed at ameliorating vein graft implantation injury by delivering Edifoligide, an oligonucleotide decoy of E2F, a key transcription factor involved in cell cycle regulation [12]. Although this study failed to show any effect of E2F blockade, it did establish the technology and demonstrate feasibility of exposure of the vein graft to molecular therapies at the time of surgery, setting the stage for future trials using alternative molecular targets.

Discoveries of novel therapies to prevent/treat vein graft implantation injury have been hampered by the choice of experimental animal models whose results often fail to translate to the clinic, mostly due to inability to recapitulate human disease^6^. The canine model of autologous vein grafting is one of few models that reproduce failure patterns and modes of healing in clinical disease^7^. In particular, these veins develop lesions of IH within 30–90 days post-implantation. In the present study, we used this canine model, and interrogated the transcriptome in a temporal and cell type specific manner in order to better understand the pathophysiology of vein graft implantation injury and identify novel therapeutic strategies. Specifically, we used Laser Capture Microdissection (LCM) to isolate endothelial and medial layers for subsequent RNA extraction and transcription profiling. Our study focused on early transcriptional events that we believe to be key in dictating outcome. We did not interrogate transcriptional changes of the neointima since this layer does not develop in this model, or in clinical vein grafts before 30 D. In fact IH is not significant in this model before 60–90 days. Future work is focused on interrogating neointimal transcriptome in a long-term model of canine vein graft failure

LCM yielded highly enriched EC and SMC samples allowing the study of cell specific transcriptional changes. To our knowledge, this is the first study using this approach. Unsupervised PCA analysis of our gene chips demonstrated that graft EC and SMC genes formed time dependent clusters with maximal transcriptional changes appearing 12 to 24 H following implantation, and diminishing over the 30 D time period. This tapered response suggests that early intervention during the peri-operative period may be sufficient to prevent/inhibit vein graft implantation injury, and therefore limit the development of IH.

Supervised analysis of the transcriptional data depicted temporal modification of gene expression in EC and SMC. Interestingly, some genes were modified over continuous time-points, whereas others were either non-continuously modified or modified at a single time-point, highlighting the importance of defining optimal therapeutic windows for each given target. For example, the proinflammatory chemokine IL-8 was up-regulated in EC from 2 H up to 7 D, and in SMC from 12 H up to 7 D. This pattern of up-regulation in both cell types could be ideal for a targeted therapy aiming at interrupting early and mid pathogenic inflammatory culprits involved in vein graft implantation injury. In contrast, Col1A1 was up-regulated in both EC and SMC only at 30 D, suggesting that this extracellular matrix gene is likely a downstream participant in the pathogenesis of vein graft implantation injury, and key to the vascular remodeling that occurs in vein grafts. In addition this analysis also suggested that graft EC might be the first responders to vein graft implantation injury, as they obviate a change in their transcriptome as early as 2 H following implantation. In contrast, SMC do not show any significant changes at this early time-point.

Gene ontology analysis on temporally, and differentially expressed genes indicated that implantation injury affected multiple ontological categories in both, EC and SMC. Enriched GO clusters included apoptosis, inflammatory and immune responses, mitosis and extracellular matrix reorganization. In EC, apoptosis related genes were up-regulated and peaked at 2 H following implantation confirming that rapid EC damage and loss was critical in triggering the cascade of events leading to vein graft injury [31]. In contrast, apoptosis related genes were up-regulated and peaked in SMC at 12 H. The significance of increased expression of pro-apoptotic genes in medial SMC is still unclear. Several studies suggest that increased medial SMC apoptosis promotes vascular damage and influences the phenotypic switch of these cells from contractile to synthetic, therefore promoting IH. On the other hand, increased apoptosis in neointimal SMC is beneficial through reduction of established IH lesions [32], [33].

Significantly heightened inflammatory responses detected in both EC and SMC at 12 and 24 H suggested a central role for inflammation in driving the injury response to vein graft implantation. By 7 D, inflammation related genes tapered down without returning to basal levels. In fact, inflammatory genes spanned all time-points, were the most abundant of differentially regulated genes, and were likely the key regulators of other pathogenic processes. We propose that early targeting of key inflammatory molecules could be an ideal approach to prevent vein graft implantation injury. Previous *in vivo* studies have demonstrated that blocking inflammatory responses do attenuate IH [34], [35]. Following heightened inflammation driven injury response, we observed at later time-points, a significant up-regulation of cell cycle related genes in both EC and SMC. Recovery of the endothelium through cell proliferation is beneficial to the healing process, while that of SMC may be deleterious, as it promotes the development of a neointimal layer, i.e. IH, the pathognomonic feature of mid-term vein graft failure. Accordingly, therapies aimed at preventing/treating vein graft failure using cell cycle inhibitors must spare EC, specifically target SMC, and ideally be effective for at least a week following vein graft implantation. Failure of the PREVENT trial may be related to the therapeutic agent not fulfilling all of these criteria.

Genes involved in the extracellular matrix (ECM) reorganization were significantly up-regulated by 30 D in both EC and SMC. In particular, collagen genes were the most enriched class of ECM components, consistent with the prominence of collagen as a constituent of IH lesion [36]. Deposition of ECM is essential for vein graft healing in response to injury under normal circumstances, however in the special case of implantation injury it can lead to stenosis and graft failure. Delayed therapies that could tackle ECM deposition need to be optimized for reducing pathogenic vascular remodeling while promoting positive vascular remodeling [37].

In order to delineate a causality relationship between differentially expressed genes after vein graft implantation, we analyzed the data using a backpropagation approach that integrates interactions between differentially expressed genes from the different points, starting at the latest time-point; i.e. 30 D. This means of analyzing the data offered a unique perspective of identifying the upstream pathogenic effectors of vein graft implantation injury, based on endpoint molecular signals involved in lesion formation. Furthermore, this approach allowed us to integrate into the same network genes derived from significantly affected biological pathways and define interconnectivity between these pathways. We identified 6 and 5 biological pathways that were dominant in the backpropagation networks of EC and SMC, respectively. Remarkably 4 of these pathways were common to both cell types, three of which spanned all time-points, namely the IL-8, IL-6 and dendritic cell maturation pathways. We surmise that these 3 pathways are not only critical pathogenic effectors of vein graft implantation injury, but also harbor promising therapeutic targets. Within these pathways, the IL-8 gene itself was up-regulated from 2 H to 7 D in EC and from 12 H to 7 D in SMC, and the IL-6 gene was up-regulated from 12 H to 7 D in both EC and SMC.

We propose that both IL-8 and IL-6 are central to the pathogenesis of vein graft implantation injury. Specifically, IL-8 is a pro-inflammatory CXC chemokine produced mainly by neutrophils, monocytes and macrophages and also by EC and vascular SMC in response to pro-inflammatory stimuli in a NF-κB and activator protein-1 (AP-1) dependent manner [38]–[41]. IL-8 stimulates vascular endothelial growth factor (VEGF) expression and the autocrine activation of VEGF Receptor 2 (VEGFR2) in EC by activating NFκB thus promoting pro-inflammatory angiogenesis, which has been associated with increased adventitial neovascularization and IH [42]–[44]. IL-8 also leads to proliferation and migration of vascular SMC thereby contributing to IH [45].

IL-6 is a pro-inflammatory cytokine mainly secreted by activated macrophages and lymphocytes but also by EC and SMC [46]–[48]. IL-6 is involved in immune regulation, hematopoiesis, inflammation and oncogenesis [49]. Although little is known about the role of IL-6 in the pathophysiology of IH leading to vein bypass graft failure, several studies have demonstrated that IL-6 is pro-atherogenic through promoting EC dysfunction, SMC proliferation and migration as well as recruitment and activation of inflammatory cells [50]–[53].

Besides IL-8 and IL-6 several other genes from these pathways have been associated with vascular remodeling and hence could affect vein graft implantation injury. In particular, we noted increased levels of transcriptional regulators such as NF-κB (P100/P50), cytokines and cytokine receptors such as IL1A, IL1B and IL1R2, regulators of extracellular matrix such as MMP2, COL1A1 and collagen type 1; and decreased levels of regulators of cell differentiation such as serum response factor (SRF) [54]–[56].

Based on the backpropagation networks, we also delineated the top focus gene hubs that had the greatest interaction density in both EC and SMC. The choice of these focus gene hubs was based on the fact that they provide maximum stability to the backpropagation network. In fact, targeting any of these genes as modeled by their removal from the network offers the most effective means to disrupt the network. Among those focus gene hubs, IL-6, INSR and IGF1R are the genes showing the greatest interaction density, closely followed by IL-8, IL-15 and FGFR2. In fact, among those genes, IL-6 and IL-8 were the most up-regulated genes, whereas INSR, IGF1R and FGFR2 were down-regulated. These results further validate the key role of IL-6 and IL-8 as pathogenic, and therefore as high profile therapeutic targets to prevent vein graft implantation injury.

We have validated the up-regulation of IL-6 and IL-8 in graft EC and SMC by qRT-PCR, and confirmed that IL-6 significantly increases from 2 H and up to 7 D post-implantation while IL-8 is up-regulated at all time-points including the 30 D time-point in both cell types. Using qRT-PCR we also validated the up-regulation of Coll11A1 at the later time-points, 7 and 30 D suggesting a potential role for extracellular remodeling in driving the healing process, while being the major component in lesion of implantation injury. Current work in our laboratory is aimed at developing local siRNA based therapies to concomitantly target IL-6 and IL-8 secretion within the vein graft and evaluate how this would impact vein graft implantation injury.

In conclusion, this study represents the first comprehensive analysis of the genomic response to vein graft implantation injury in a large animal model. LCM has made it possible to separately define the genomic response of EC from that of medial SMC. Our data indicates that a robust genomic response begins by 2 H, peaks at 12–24 H, starts resolving by 7 D, and declines markedly by 30 D. Inflammatory pathways dominate the early response, followed by modulators of cell cycling, and culminate in pathways involved in extra-cellular matrix remodeling. By using a back-propagation based systems biology analysis of the data, we were able to establish a temporal and causative link between these pathways that helped us identify the molecular signature of vein graft implantation injury, including high intensity hubs. This information provides a foundation for designing strategies for therapeutic intervention to prevent or diminish implantation injury.

## Materials and Methods

### Canine Surgery

Unilateral reversed autologous cephalic vein to femoral artery interposition graft surgery was performed, as described [18], [57], [58] on 25-kg female mongrel dogs (n=3 animals per time-point). Cephalic vein grafts, along with unperturbed contralateral cephalic vein, which served as an experimental control, were excised at the same time-points (2, 12 and 24 H and at 7 and 30 D). Surgical details are in Materials S1. All animal work protocol (# 02606) was approved by Harvard Medical Area (HMA) Standing Committee on Animals. The principal investigator on the animal protocol is Dr. Mauricio Contreras. For detailed surgical procedures please refer to Materials S1. Figure S7 depicts representative histology images of control veins and vein grafts at 2, 12 and 24 H, and 7 and 30 D.

### Laser Capture Microdissection (LCM) preparation and RNA preparation

Six microns OCT embedded frozen cross-sections were immediately placed on glass slides coated with LPC-membrane (POL or PET Foil 1.35 mm, P.A.L.M. Microlaser Technologies, Bernried, Germany), and stored at −80°C until microdissection. LCM was performed using a P.A.L.M. microscope. We first dissected the entire intimal layer throughout the entire vessel circumference thereby isolating the endothelium, and repeated this for the entire medial layer to capture medial SMC (Figures S8A & S8B). We used 6–7 cross-sections per sample in order to obtain sufficient amount of total RNA (minimum of 500 picograms) for amplification by the NuGEN WT-Ovation Pico RNA Amplification System (Version 1.0). Before labeling and hybridization to arrays, we combined layers from different cross-sections of each sample for EC and SMC. RNA was subsequently extracted from EC and medial SMC, amplified, fragmented and biotinylated using NuGen FL-Ovation kit (NuGen, San Carlos, CA). For details about RNA isolation, amplification, fragmentation and biotinylation from EC and SMC please refer to the Materials S1.

### Quantitative Real time PCR

Quantitative RT-PCR (qRT-PCR) was performed as described to evaluate sample purity and validate target genes [59]. For validation of tissue sample purity, EC (CD31) and SMC (Myosin Heavy Chain II – MHCII) specific probes were used. For validation of differentially expressed genes IL-6, IL-8 and Collagen11A1 (Col11A1) specific primers were used. PCR was performed in each cell-type from control vein and vein graft of three different animals at each time-point. All primers were synthesized by Integrated DNA Technologies (Coralville, IA). Primer sequences are provided in Table S1.

### Transcriptional profiling and data analysis

Transcriptional profiling was performed on canine genome 2.0 Affymetrix GeneChip, that contains >43,000 transcripts. Three microarrays of both control veins and vein grafts per cell type and per time-point were used. The three arrays were biological not technical replicates, as they were obtained from 3 different animals at each time. From each animal, we retrieved both control vein and vein graft. After quality control analysis, scanned array images were normalized by dChip [60]. Unsupervised analysis was performed on normalized and preprocessed data using Principal Component Analysis (PCA) and hierarchical clustering [61], [62]. The Pearson Correlation test with complete-linkage method was used to cluster control and graft samples from EC and SMC at each time-point. Differentially expressed genes were identified using a linear model from the ‘LIMMA’ package [63]. Transcripts with absolute fold change ≥2 between control vein and vein graft with a multiple test corrected *p-value* ≤0. 05 were considered differentially expressed. Details of analyses are described in Materials S1.

### Time series analysis of gene expression data

To use full time and class information of the results, we also analyzed the preprocessed data in a time series manner using the Bayesian Estimation of Temporal Regulation (BETR) [64]. Details of analyses are described in Materials S1.

### Gene Ontology (GO) enrichment analysis

To identify the over-represented GO categories in differentially expressed genes, we used the Biological Processes and Molecular functions Enrichment Analysis available from the Database for Annotation, Visualization and Integrated Discovery (DAVID) [65]. Details of GO analyses are described in Materials S1.

### Pathways and interactive network Systems Biology analysis

The Ingenuity Pathway Analysis (IPA 7.0) (http//www.ingenuity.com) was used to identify key interaction networks and pathways significantly affected at different time-points in EC and SMC. We developed a hierarchical network based on a backpropagation approach starting from transcriptional changes at 30 days back to 2 hours (Figure S9). Using the 30 days base-layer of differentially expressed genes, we build a network comprising upstream interactive genes, using the network building and growing utility in the IPA tool. Enriched pathways within this hierarchical network were ranked using ratio of affected genes and Fisher's exact test. We then selected the top pathways that included at least 10% of the genes from the hierarchical network, and generated an integrated network using protein-protein, protein-DNA, and protein-RNA known interactions. To identify the key regulatory molecules within this integrated network, we used density of maximum neighborhood component (DMNC) algorithm [30]. Details of analyses are described in Materials S1.

## Supporting Information

Materials S1 **Text file for supplemental materials.** A) List of supplemental materials B) Supplemental Methods and, C) Supplemental Material References.
(DOCX)

Figure S1 **Q-RT-PCR based purity analysis of EC and SMC isolated by LCM technique.** A) mRNA expression of CD31 in SMC compared to EC B) mRNA expression of MHCII in EC compared to SMC. Results are expressed as mean ± SEM of 3 animals.
(TIF)

Figure S2 **Immune cell contamination of vein grafts.** A) Representative immunohistochemistry image of CD3+ cell infiltration within control veins and vein grafts B) Representative immunohistochemistry image of CD18+ cells within control veins and vein grafts.
(TIF)

Figure S3 **Unsupervised Pearson Correlation based clusters of EC and SMC arrays at each time-point after normalization and preprocessing of data.** In most cases biological replicates of each cell type have better correlation with each other than with other cell types. Unsupervised hierarchical clustering depicted more transcriptional differences between control vs. graft than between cell types at 12 H, 24 H and 7 D. Clustering also depicted less transcriptional differences between control vs. graft than between cell types at 2 H and 30 D consistent with PCA results (Figure 1).
(TIF)

Figure S4 **Venn diagram analysis on significantly differentially expressed genes at five different time-points** (**2, 12 and 24 H, and 7 and 30 D**) **from graft vein EC and SMC compared to control vein EC and SMC.** A) up-regulated genes B) down-regulated genes. Each eclipse represents one time-point as indicated by zones of overlapping expression. With each of these zones, overlapping circles represent 3 sets differentially expressed genes, that is, only EC, only SMC and common to EC and SMC. Pink and Blue circles denote genes differentially expressed in EC and SMC respectively. The list of genes from each quadrant is provided in Table S2.
(TIF)

Figure S5 **Expression patterns of temporally differentially expressed genes identified using K means clusters.** A) EC clusters B) SMC clusters. Each cluster represents a set of genes that depict similar expression pattern and are biologically linked to a specific function. Genes are selected using time-series analysis of vein graft and control veins. X-axis represents different time-points and Y-axis represents gene expression on pseudoscale from −3 to +3. For details on clusters please refer to Figure 2.
(TIF)

Figure S6 **Top pathways comprising backpropagation network.** A) EC and B) SMC. This analysis identified 6 pathways in EC and 5 pathways in SMC, affecting at least 10% of backpropagation genes. The up- and down-regulated genes are represented in red and green color respectively. The intensity of the color representing each gene corresponds to the magnitude of up- or down regulation of that gene in graft and control vein EC and SMC. Size of the symbol representing the gene indicates the number of connections that gene makes.
(TIF)

Figure S7 **Representative histology images of control vein and vein graft.** H&E stained histological images at low (4X or 10X) and high (40X) magnification of control vein and vein graft at 2 H, 12 H, 24 H, 7 D and 30 D following implantation.
(TIF)

Figure S8 **Representative Image of Laser Capture Mircodissection** (**LCM**)**.** A) Medial and endothelial layers in vein graft prior to LCM (Mag 40X), B) Isolated endothelial layer after LCM (Mag 40X).
(TIF)

Figure S9 **Workflow for generation of hierarchical back-propagation network.**
(TIF)

Table S1 **List of Q-RT-PCR Primers.**
(DOCX)

Table S2 **List of unique differentially expressed genes identified by comparing graft vs. control vein EC and SMC at individual time points** (**2, 12, 24 H, and 7 and 30 D**)**.** The table represents fold change of significantly dysregulated genes from each zone of the Venn diagram shown in Figure S2.
(PDF)

Table S3 **Gene Ontology analysis of K-means clusters. A) EC, and B) SMC:** This analysis is performed using the Database for Annotation, Visualization and Integrated Discovery (DAVID) v6.7 and gene ontology categories with multiple tests (Holm–Bonferroni method) corrected P value <0.05 were considered significant.
(PDF)

Table S4 **List of genes from significantly enriched canonical pathways in vein grafts at different time points.** A) EC, B) SMC. This analysis is performed using Igenuity Pathway Analysis System and Pathways with multiple test (Holm–Bonferroni method) corrected P value <0.01 was considered significant.
(PDF)

Table S5 **List of genes from significantly enriched disease** **pathways in vein grafts at different time points.** A) EC, B) SMC. This analysis is performed using Ingenuity Pathway Analysis System and Pathways with multiple test (Holm–Bonferroni method) corrected P value <0.01 was considered significant.
(PDF)
