# Supplementary material for: Temporal Network Based Analysis of Cell Specific Vein Graft Transcriptome Defines Key Pathways and Hub Genes in Implantation Injury
Source: PLoS One. 2012 Jun 15;7(6):e39123. doi: 10.1371/journal.pone.0039123 (PMC3376111; doi:10.1371/journal.pone.0039123)
Supplement: Materials S1 — Text file for supplemental materials. A) List of supplemental materials B) Supplemental Methods and, C) Supplemental Material References. (DOCX) [file pone.0039123.s001.docx]

**Materials S1**

**Temporal network based analysis of cell specific vein graft transcriptome defines key pathways and hub genes in implantation injury**

Manoj Bhasin^2^, Zhen Huang^1^, Leena Pradhan-Nabzdyk^1^, Junaid Y. Malek^1^, Philip J. LoGerfo^2^, Mauricio Contreras^1^, Patrick Guthrie^1^, Eva Csizmadia^4^, Nicholas Andersen^1^, Olivier Kocher^3^, Christiane Ferran^1, 5^, Frank W. LoGerfo^1^

**Author Affiliation:**

^1^Department of Surgery, Division of Vascular and Endovascular Surgery, Beth Israel Deaconess Medical Center, Harvard Medical School, Boston, MA, USA

^2^Genomics and Proteomics Center, Div. of Interdisciplinary Medicine and Biotechnology, Beth Israel Deaconess Medical Center, Harvard Medical School, Boston, MA, USA

^3^Deptartment of Pathology, Beth Israel Deaconess Medical Center, Harvard Medical School, Boston, MA, USA

^4^Center for Vascular Biology Research, Beth Israel Deaconess Medical Center, Harvard Medical School, Boston, MA, USA

^5^Center for Vascular Biology Research and Division of Nephrology, Dept. of Medicine, Beth Israel Deaconess Medical Center, Harvard Medical School, Boston, MA, USA

**Corresponding Authors:**

Frank W. LoGerfo, MD

Beth Israel Deaconess Medical Center

110 Francis St. Suite 2B

Boston, MA 02215

Email: flogerfo@bidmc.harvard.edu

Christiane Ferran, MD, PhD

Center for Vascular Biology Research

Beth Israel Deaconess Medical Center

99 Brookline Ave., RN 370

Boston, MA 02215

Email: cferran@bidmc.harvard.edu

**A) List of Supplemental Materials:**

1. **Methods:**
   1. Details of vein bypass surgery procedure
   2. Histology and Immunohistochemistry
   3. RNA Isolation, Amplification, Fragmentation, Biotinylation
   4. Transcriptional Profiling
   5. Quality Control and Normalization of data
   6. Unsupervised Analysis
   7. Identification of Differentially Expressed Genes
   8. Time Series Analysis Of Gene Expression Data
   9. Mapping Of Canine Transcripts To Human Transcripts
   10. Gene Ontology (GO) Enrichment Analysis
   11. Pathways And Interactive Network Analysis
   12. Development of Back-propagation Network
   13. Identification Of Top/Dominant Pathways
   14. Systems Biology Networks Generation and Focus Gene Hubs identification
2. **Supplemental Material References**
3. **Supplemental Tables:**
   1. Table S1: List of Q-RT-PCR Primers
   2. Table S2: **List of unique differentially expressed genes identified by comparing graft vs. control vein EC and SMC at individual time points (2, 12, 24 H, and 7 and 30 D)**
   3. Table S3: **Gene Ontology analysis of K-means clusters. A) EC, and B) SMC**
   4. Table S4: List of genes from significantly enriched canonical pathways in vein grafts at different time points
   5. Table S5: List of genes from significantly enriched disease pathways in vein grafts at different time points
4. **Supplemental Figures**
   1. Figure S1: Q-RT-PCR based purity analysis of EC and SMC isolated by LCM technique
   2. Figure S2 : Immune cell infiltration within vein graft
   3. Figure S3 : Unsupervised Pearson Correlation based clusters of EC and SMC arrays at each time points after normalization and preprocessing of data
   4. Figure S4: Venn diagram analysis on significantly differentially expressed genes at five different time points (2, 12 and 24 H, and 7 and 30 D) from graft vein EC and SMC compared to control vein EC and SMC
   5. Figure S5: Expression patterns of temporally differentially expressed genes identified using K means clusters
   6. Figure S6: Top pathways comprising backpropagation network
   7. Figure S7: Histology images of control veins and vein grafts
   8. Figure S8: Representative Image of Laser Capture Mircodissection (LCM)
   9. Figure S9: Workflow for generation of hierarchical back-propagation network.

**B. Supplemental Methods:**

1. **Details of vein bypass surgery procedure**

**Bilateral Cephalic Vein Excision and Preparation**

General anesthesia was established and maintained with an initial sodium pentothal injection and subsequent 1% isofluorane inhalation after orotracheal intubation. Autologous cephalic vein was harvested by infiltration of perivenous tissues with papaverine solution (papaverine hydrochloride 60mg/ Lactated Ringer’s 500ml and 2,000U of heparin sodium) prior to skin incision. Approximately 10 cm of cephalic vein was excised and placed in papaverine solution until implantation.

**Femoral Artery Autologous Vein Bypass Surgery and Harvest: 24 H and 7 and 30 D**

The Superficial Femoral Artery (SFA) was exposed and controlled with vessel loops, and 3,000U of heparin were given IV. A 8 to 10 cm segment of SFA was bypassed using a 10 cm segment of previously harvested autologous cephalic vein and sutured end-to-side at the proximal and distal anastomoses with running 7-0 proline sutures. The intervening SFA segment was ligated and transected, establishing a functional end-to-end anastomosis.

At the end of the procedure, protamine sulphate (0.3mg/kg IV) was administered to reverse the heparin effects, and the procedure was completed. Vein grafts, as well as contralateral cephalic veins (used as control veins) were harvested at 24 H and 7, and 30 D after implantation and were flushed with papaverine solution. Mid-portions of vein graft and control vein were placed in O.C.T. compound (Sakura Finetek, Torrance, CA), snap frozen and stored at -80°C for LCM/mRNA isolation. All bypass grafts were patent at the time of excision.

**Femoral Artery Autologous Vein Bypass Surgery and Harvest: 2 and 12 H**

The initial bypass was performed in the same manner as for the other time points described above. After the initial bypass was completed, the wound was packed with saline-laden sterile gauze. While the first femoral bypass vein graft is open (with blood flowing through the vein graft) for the initial 2 H, a second cephalic vein femoral artery bypass graft was completed in the contralateral groin (12 H time point), following the same anastomotic technique described earlier (sutured end-to-side with running 7-0 proline sutures) for both, the proximal and distal anastomoses. After 2 H, the first bypass graft and the contralateral cephalic vein were harvested. A 3 cm portion of contralateral cephalic vein served as 2 H control tissue and the remainder was placed as the 12 H bypass conduit. As described above at the end of procedure, protamine sulphate (0.3mg/kg, IV) was administered.

For the 12 H vein graft harvest, the left saphenous vein was harvested as control vein (in the same manner as cephalic vein harvest). All vein grafts and control veins were immediately placed in O.C.T. and stored at -80°C until LCM/mRNA isolation. All bypass grafts were patent at harvest.

Both control vein and vein graft were harvested and stored in a similar manner. Thus any changes observed in subsequent analyses are solely due to the differences evoked by implantation injury and not by technical differences in tissue harvesting.

1. **Histology and Immunohistochemistry of control vein and vein graft**

Tissue samples were fixed in formalin and embedded in paraffin (24 H and, 7 and 30 D) or placed in OCT and frozen (2, 12 and 24 H and, 7 and 30 D)). Six μm sections were cut and deparaffinized in xylene and rehydrated, or in case of OCT sections fixed in cold acetone. Standard H&E staining was performed. For IHC, only frozen sections fixed in 10% acetone were used. Standard IHC techniques were used. To confirm specific staining for each primary antibody, isotype negative control and no-primary antibody control were used. CD3 primary antibody was obtained from Epitomics, Burlingame, CA (catalog # 3256-1) and CD18 primary antibody was obtained from AbD Serotec, Raleigh, NC (catalog # MCA1780).

1. **RNA Isolation, Amplification, Fragmentation, Biotinylation**

Total RNAs were isolated with PicoPure RNA Isolation Kit according to the manufacturer’s protocol, and amplified using the NuGEN WT-Ovation Pico RNA Amplification System (Version 1.0; NuGEN Technologies, Inc., San Carlos, CA), according to the manufacturer’s protocol except for the addition of yeast tRNA carrier during the purification step. Zymo Research DNA Clean & Concentrator (Zymo Research Corporation, Orange, CA) was used for cleaning and concentrating the RNA. Yield was quantified using a Nanodrop (Nanodrop Products, Wilmington, DE) and a minimum of 5 μg total RNA was obtained prior to the next step. The sample was fragmented and biotinylated using NuGEN FL-Ovation cDNA Biotin Module V2 and stored at -20°C until microarray hybridization. Before fragmentation, 100ng of total RNA was kept for qRT-PCR studies. Samples were at room temperature for a maximum of 1 H.

Captured EC and SMC were stored in extraction buffer provided by PicoPure RNA Isolation Kit (Arcturus Bioscience, Inc. Mountain View, CA) and incubated at 42°C for 30 minutes prior to storing at -80°C until RNA extraction.

1. **Transcriptional Profiling**

For transcriptional profiling, the canine genome canine 2.0 Affymetrix GeneChip, containing more than 43,000 transcripts, was used. Three microarrays of control veins and vein grafts were performed at each time point using RNA from one animal per microarray. Microarray experiment was conducted by the Genomics Center at the Beth Israel Deaconess Medical Center using standard Affymetrix protocol. All the hybridized arrays were scanned using Affymetrix  GeneChip® **Scanner** 3000 and images were processed using Affymetrix GCOS software.

1. **Quality Control and Normalization of data:**

The quality of scanned array images were determined on the basis of background values, percent present calls, scaling factors, and 3’-5’ ratio of βactin and GAPDH using the Simpleaffy package for R [1,2]. Scanned array images were analyzed by dChip for normalization and outlier analysis. The raw probe level data was normalized using smoothing-spline invariant set method, and the signal value for each transcript was summarized using the PM-only based signal modeling algorithm in which the signal value corresponds to the absolute level of expression of a transcript [3]. To calculate model based expression signal values, array and probe outliers were interrogated and image spikes were treated as signal outliers. The outlier detection was carried out using dChip outlier detection algorithm. A chip was considered to be an outlier if the probe, signal or array outlier percentage exceeded a threshold of 10%.

1. **Unsupervised Analysis**

The unsupervised analysis was performed using Principal Component Analysis (PCA), which projects multivariate data objects onto a lower dimensional space while retaining as much of the original variance as possible [4,5]. Before PCA, transcripts were filtered to include only those with covariance > 0.5 and absolute expression ≥ 20 in at least 10% of samples. This yielded 20% of the total transcripts (8314). PCA of this data set illustrates a temporal expression profile of graft and control EC and SMC within a two-dimensional plane. Unsupervised analysis was also performed using hierarchical clustering analysis (HCA). For HCA, Pearson correlation test with complete-linkage method was used to cluster control and graft samples from EC and SMC.

1. **Identification of Differentially Expressed Genes**

To identify differentially expressed genes, a linear model was implemented using linear model microarray analysis software package (LIMMA) [6]. At each time point, differentially expressed probes were identified using LIMMA, which estimates the differences between graft and control veins by fitting a linear model and using an empirical Bayes method to moderate standard errors of the estimated log-fold changes for expression values from each probe set. In LIMMA, all probes were ranked by t statistic using a pooled variance, a technique particularly suited to small numbers of samples per cell type. The differentially expressed probes were identified on the basis of absolute fold change and Benjamini and Hochberg corrected P value [7].

# Time Series Analysis Of Gene Expression Data

# The analysis method described above identifies differentially expressed genes treating each time point as independent, but ignores important correlations, including those within samples and between sampling times. In order to use the full information of time and class, we analyzed the preprocessed data in a time series manner using the Bayesian Estimation of Temporal Regulation (BETR) [8]. BETR is a flexible linear random-effects modeling framework that takes into account correlations between samples and sampling times. Time series analysis was performed on the pre-pressed data by removing all the low expressing transcripts and transcripts with low variance. The probes with critical *p-value* <0.01 were considered significantly different between class and time. The differentially expressed genes were further partitioned in expression dependent subsets using K-mean clustering.

# Mapping Of Canine Transcripts To Human Transcripts

We developed a script in Practical Extraction and Report Language (PERL) to map canine transcripts to human orthologues, using Affymetrix array comparison database. The probes of canine 2.0 probe arrays were mapped to human U133 plus 2.0 probes. The mapping was performed by developing a script in PERL using array comparison database from Affymetrix. The script will be available to public from authors on request.

1. **Gene Ontology (GO) Enrichment Analysis**

To identify the over-represented GO categories in temporally differentially expressed genes, we used the Biological Processes and Molecular functions Enrichment Analysis available from the Database for Annotation, Visualization and Integrated Discovery (DAVID) [9]. DAVID is an online implementation of the EASE software that produces a list of over-represented categories using jackknife iterative re-sampling of the Fisher exact probabilities. A *p-value* gets assigned to each category on the basis of enrichments. Smaller P values reflect increasing confidence in over-representation. The GO categories with multiple test corrected *p-values* <0.05 (Holm–Bonferroni method) were considered significant.

1. **Pathways And Interactive Network Analysis**

The Ingenuity Pathway Analysis (IPA 7.0) was used to identify the key interaction networks and pathways significantly affected at different time points in EC and SMC. The knowledge base of this software consists of functions, pathways and network models derived by systematically exploring the peer reviewed scientific literature. A detailed description of IPA analysis is available at the Ingenuity Systems’ web site (http//www.ingenuity.com). It calculates the multiple test corrected P-value for each pathway according to the fit of user’s data to IPA database using one tailed Fisher exact test. The pathways with multiple test corrected P-values <0.01 were considered significantly affected.

For each network, IPA calculates a score derived from the P value of one tailed Fisher exact test [Score=-log(P value] and indicates the likelihood of focus genes appearing together in the network due to random chance. A score of 2 or higher has at least a 99% probability of not being generated by random chance alone. The ability to rank the networks based on their relevance to the queried data sets allows for prioritization of networks with highest impact on the disease process.

1. **Development of Back-propagation Network**

To decipher the time dependent transcriptional events responsible for implantation injury, we employed a back-propagation approach starting from 30 days (D) transcriptional changes to 2 hours (H) transcriptional changes in developing hierarchical network. First layer of the network was developed from the genes that are significantly differentially expressed at 30 D. Second layer of the network was built by adding genes that are i) differentially expressed at 7 days ii) should have physical or transcriptional interaction with genes at 30 D iii) genomics location upstream of interactive genes at 30 D. In similar manner, rest three layers of the network was developed by adding genes perturbed at 24 H, 12 H and 2 H (Figure 6).  The network generation was based on successful relay of signal from upstream regulators to change in target gene expression with a time lag.

1. **Identification Of Top/Dominant Pathways**

To identify the major pathways involved in implantation injury, we performed canonical pathways enrichment analysis on hierarchical back-propagation network. The top pathways were selected on the basis of i) Enrichment P value obtained using Fisher Exact test and ii) percentage of hierarchical back-propagation network genes linked to a canonical pathways. The top pathways should included at least 10% of the genes from the hierarchical network and achieve an enrichment of P value <0.05.

1. **Systems Biology Networks Generation and Focus Gene Hubs identification:**

Genes from the top pathways were used to generate an interactive network using protein-protein, protein-DNA, and protein-RNA known interactions. The interaction information was obtained using the literature search, information from knowledge base databases such as MIPS, DIPS, and HPRD[10-12]. Network was analyzed using the cyto-Hubba plug-in for Cytoscape 2.8 platform to identify network hubs and bottlenecks, which may represent the key regulatory nodes in the network [13]. The key hubs in the network were identified using density of maximum neighborhood component (DMNC) algorithm [14].

**C. Supplemental Material References**

1. R_Development_Core_Team (2009) R: A language and environment for statistical computing. 2.10.11 ed. p.

2. Wilson CL, Miller CJ (2005) Simpleaffy: a BioConductor package for Affymetrix Quality Control and data analysis. Bioinformatics 21: 3683-3685.

3. Li C, Wong WH (2001) Model-based analysis of oligonucleotide arrays: expression index computation and outlier detection. Proceedings of the National Academy of Sciences of the United States of America 98: 31-36.

4. Wang C, Rao N, Wang Y (2007) [Principal component analysis for exploring gene expression patterns]. Sheng wu yi xue gong cheng xue za zhi = Journal of biomedical engineering = Shengwu yixue gongchengxue zazhi 24: 736-741.

5. Yeung KY, Ruzzo WL (2001) Principal component analysis for clustering gene expression data. Bioinformatics 17: 763-774.

6. Smyth GK (2004) Linear models and empirical bayes methods for assessing differential expression in microarray experiments. Stat Appl Genet Mol Biol 3: Article3.

7. Benjamini Y, Hochberg Y (1995) Controlling the false discovery rate: a practical and powerful approach to multiple testing. Journal of the Royal Statistical Society: Series B 57: 11.

8. Aryee MJ, Gutierrez-Pabello JA, Kramnik I, Maiti T, Quackenbush J (2009) An improved empirical bayes approach to estimating differential gene expression in microarray time-course data: BETR (Bayesian Estimation of Temporal Regulation). BMC bioinformatics 10: 409.

9. Huang da W, Sherman BT, Tan Q, Kir J, Liu D, et al. (2007) DAVID Bioinformatics Resources: expanded annotation database and novel algorithms to better extract biology from large gene lists. Nucleic Acids Res 35: W169-175.

10. Pagel P, Kovac S, Oesterheld M, Brauner B, Dunger-Kaltenbach I, et al. (2005) The MIPS mammalian protein-protein interaction database. Bioinformatics 21: 832-834.

11. Peri S, Navarro JD, Amanchy R, Kristiansen TZ, Jonnalagadda CK, et al. (2003) Development of human protein reference database as an initial platform for approaching systems biology in humans. Genome research 13: 2363-2371.

12. Xenarios I, Salwinski L, Duan XJ, Higney P, Kim SM, et al. (2002) DIP, the Database of Interacting Proteins: a research tool for studying cellular networks of protein interactions. Nucleic acids research 30: 303-305.

13. Smoot ME, Ono K, Ruscheinski J, Wang PL, Ideker T (2011) Cytoscape 2.8: new features for data integration and network visualization. Bioinformatics 27: 431-432.

14. Lin CY, Chin CH, Wu HH, Chen SH, Ho CW, et al. (2008) Hubba: hub objects analyzer--a framework of interactome hubs identification for network biology. Nucleic acids research 36: W438-443.
