# Supplementary figures and images for: Temporal Network Based Analysis of Cell Specific Vein Graft Transcriptome Defines Key Pathways and Hub Genes in Implantation Injury
Source: PLoS One. 2012 Jun 15;7(6):e39123. doi: 10.1371/journal.pone.0039123 (PMC3376111; doi:10.1371/journal.pone.0039123)

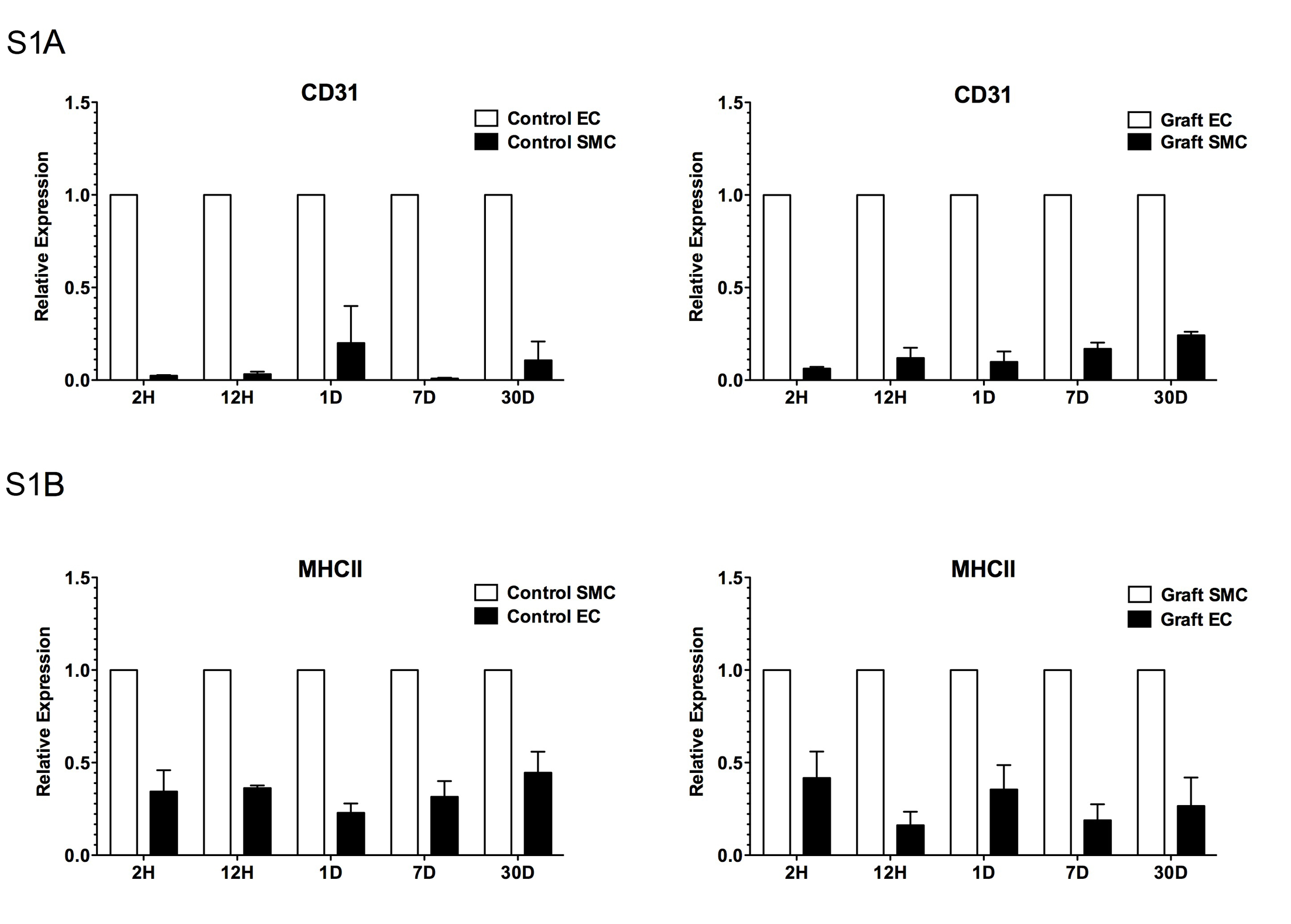

Supplement: Figure S1 — Q-RT-PCR based purity analysis of EC and SMC isolated by LCM technique. A) mRNA expression of CD31 in SMC compared to EC B) mRNA expression of MHCII in EC compared to SMC. Results are expressed as mean ± SEM of 3 animals. (TIF) [file pone.0039123.s002.tif]

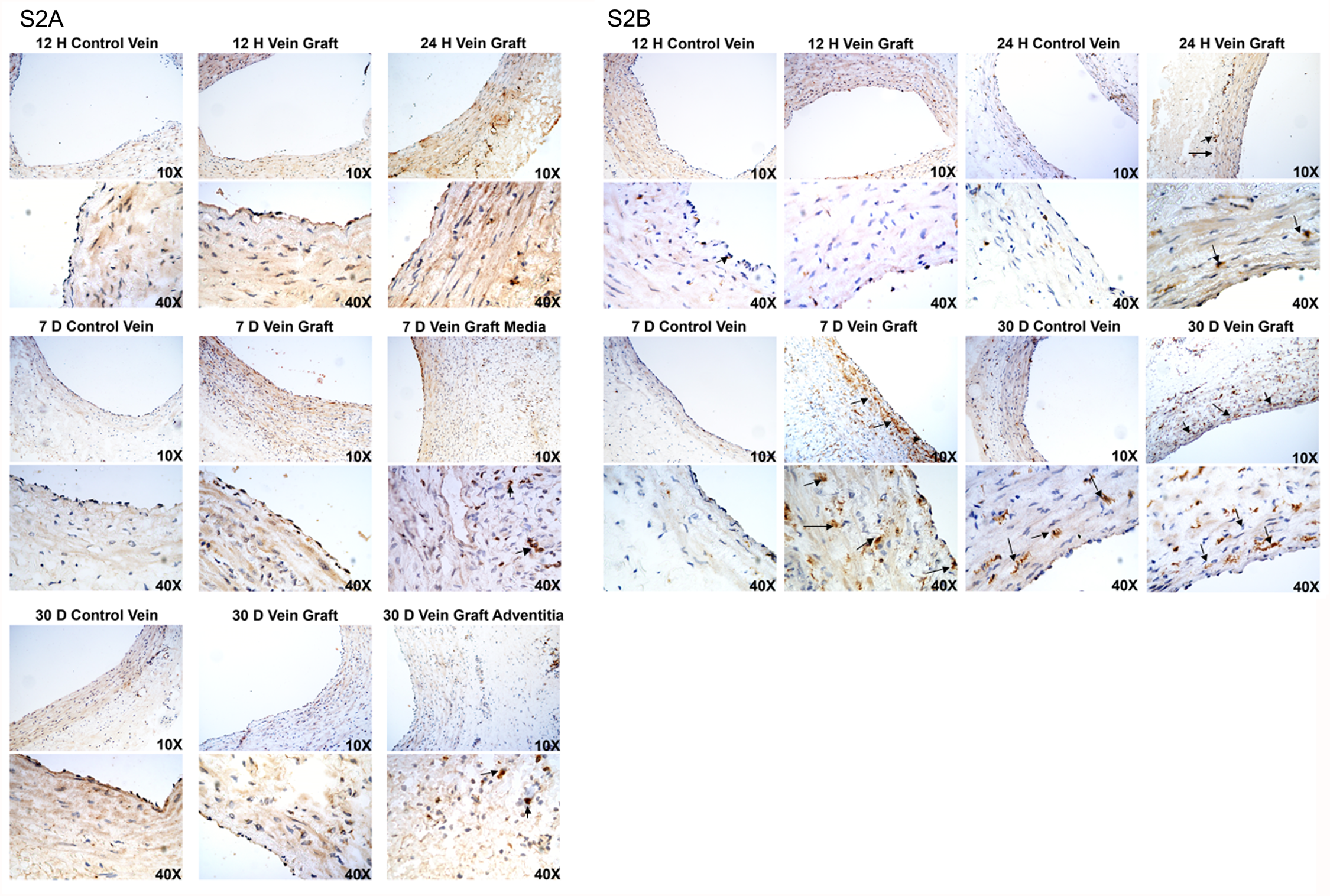

Supplement: Figure S2 — Immune cell contamination of vein grafts. A) Representative immunohistochemistry image of CD3+ cell infiltration within control veins and vein grafts B) Representative immunohistochemistry image of CD18+ cells within control veins and vein grafts. (TIF) [file pone.0039123.s003.tif]

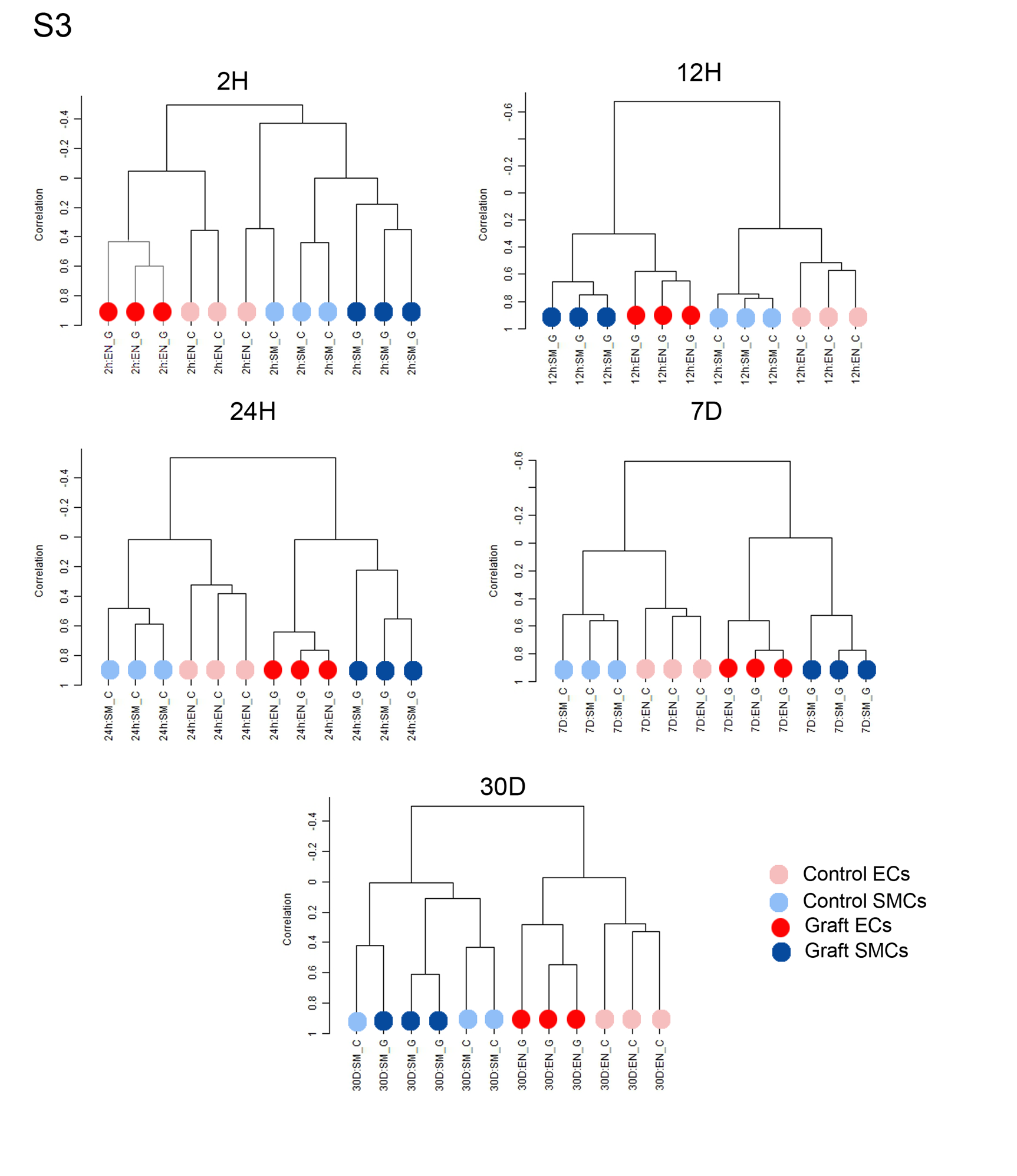

Supplement: Figure S3 — Unsupervised Pearson Correlation based clusters of EC and SMC arrays at each time-point after normalization and preprocessing of data. In most cases biological replicates of each cell type have better correlation with each other than with other cell types. Unsupervised hierarchical clustering depicted more transcriptional differences between control vs. graft than between cell types at 12 H, 24 H and 7 D. Clustering also depicted less transcriptional differences between control vs. graft than between cell types at 2 H and 30 D consistent with PCA results (Figure 1). (TIF) [file pone.0039123.s004.tif]

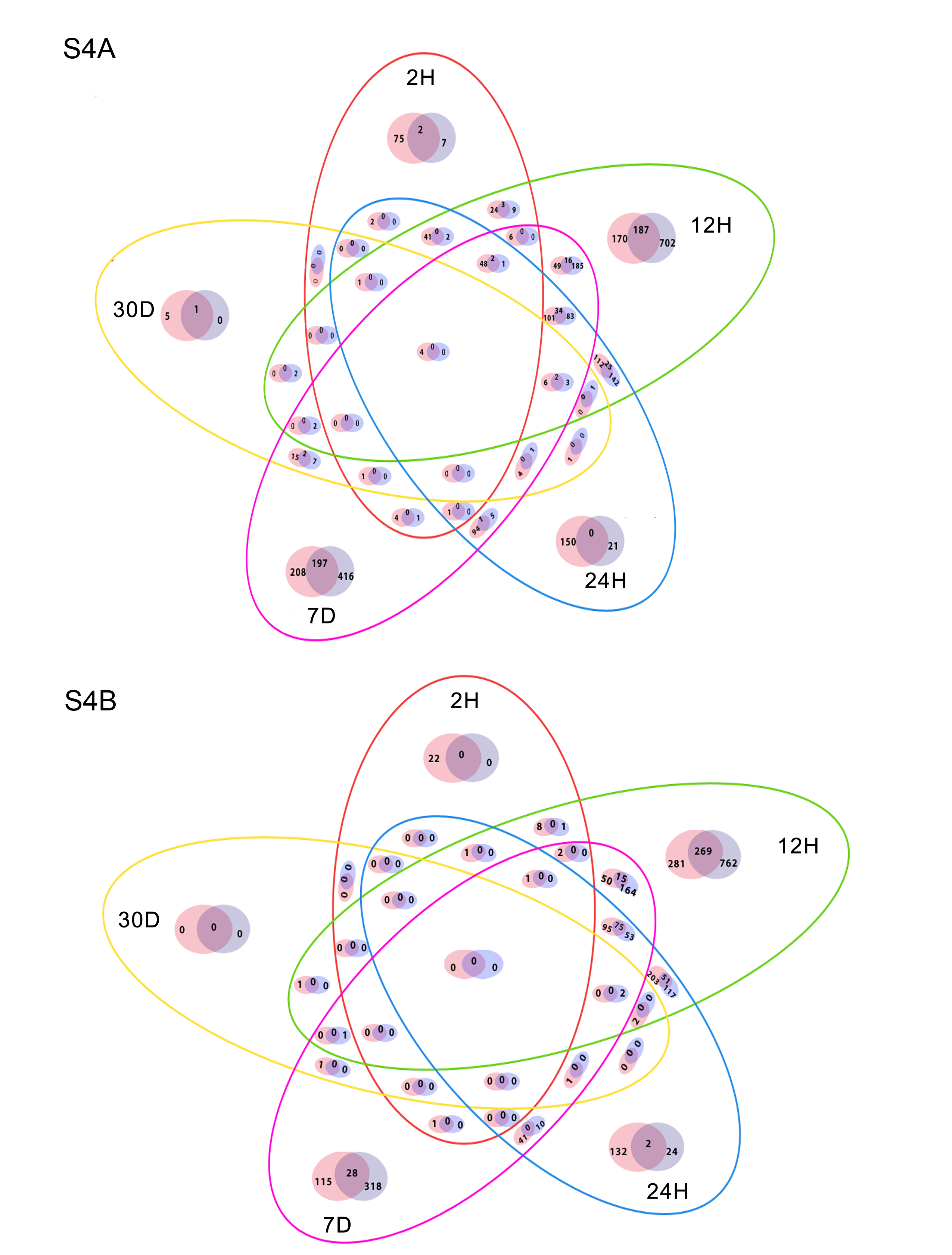

Supplement: Figure S4 — Venn diagram analysis on significantly differentially expressed genes at five different time-points (2, 12 and 24 H, and 7 and 30 D) from graft vein EC and SMC compared to control vein EC and SMC. A) up-regulated genes B) down-regulated genes. Each eclipse represents one time-point as indicated by zones of overlapping expression. With each of these zones, overlapping circles represent 3 sets differentially expressed genes, that is, only EC, only SMC and common to EC and SMC. Pink and Blue circles denote genes differentially expressed in EC and SMC respectively. The list of genes from each quadrant is provided in Table S2. (TIF) [file pone.0039123.s005.tif]

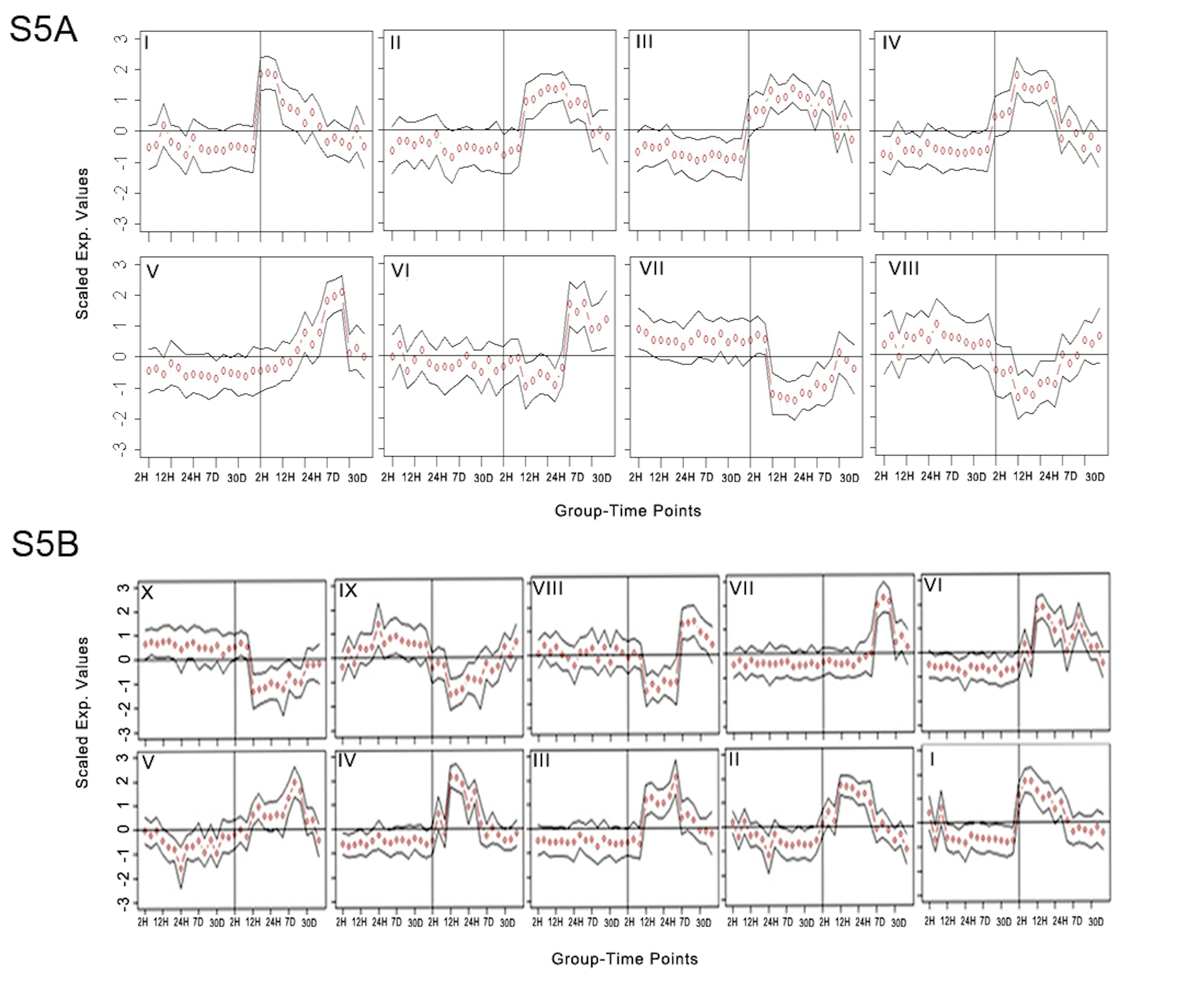

Supplement: Figure S5 — Expression patterns of temporally differentially expressed genes identified using K means clusters. A) EC clusters B) SMC clusters. Each cluster represents a set of genes that depict similar expression pattern and are biologically linked to a specific function. Genes are selected using time-series analysis of vein graft and control veins. X-axis represents different time-points and Y-axis represents gene expression on pseudoscale from −3 to +3. For details on clusters please refer to Figure 2. (TIF) [file pone.0039123.s006.tif]

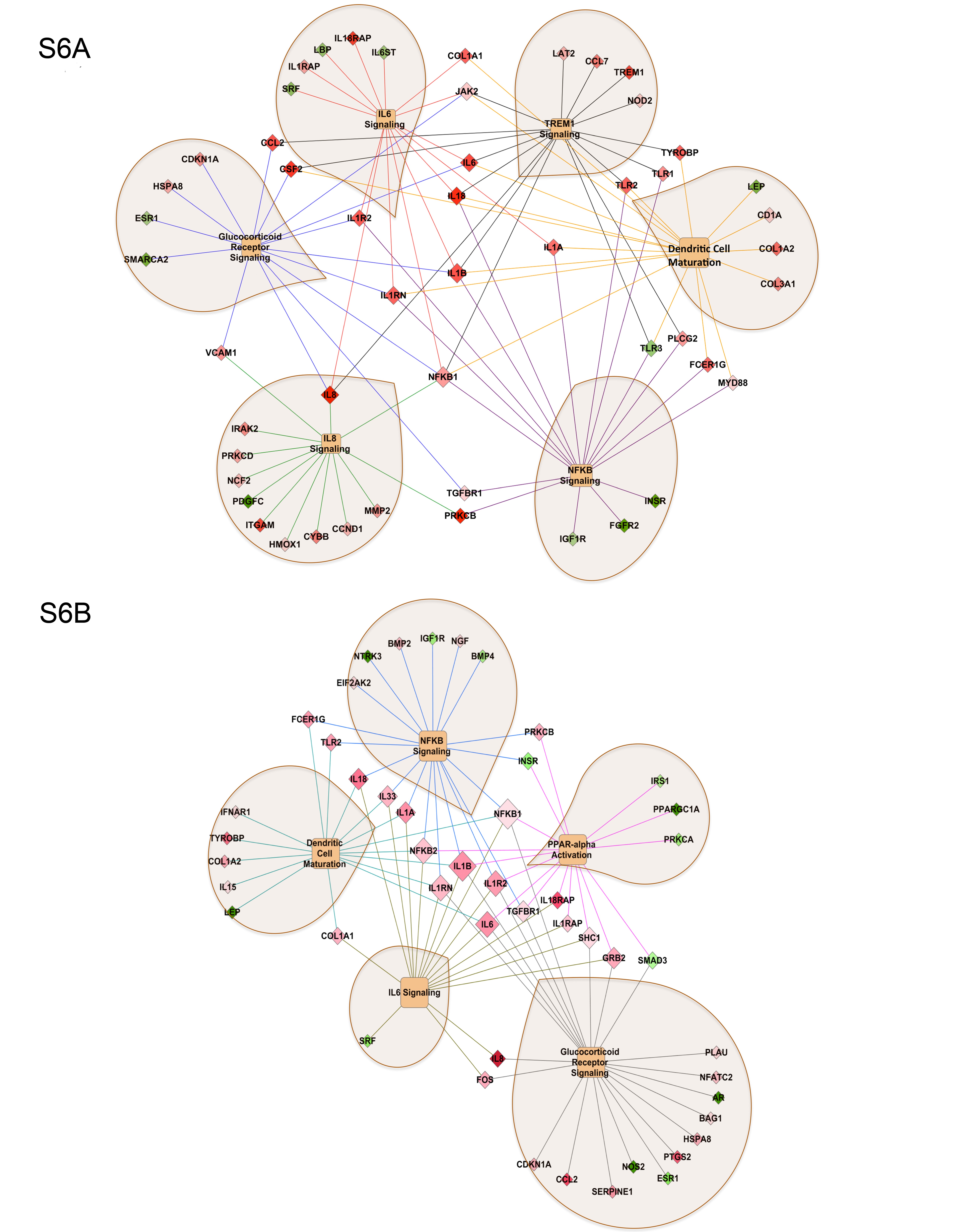

Supplement: Figure S6 — Top pathways comprising backpropagation network. A) EC and B) SMC. This analysis identified 6 pathways in EC and 5 pathways in SMC, affecting at least 10% of backpropagation genes. The up- and down-regulated genes are represented in red and green color respectively. The intensity of the color representing each gene corresponds to the magnitude of up- or down regulation of that gene in graft and control vein EC and SMC. Size of the symbol representing the gene indicates the number of connections that gene makes. (TIF) [file pone.0039123.s007.tif]

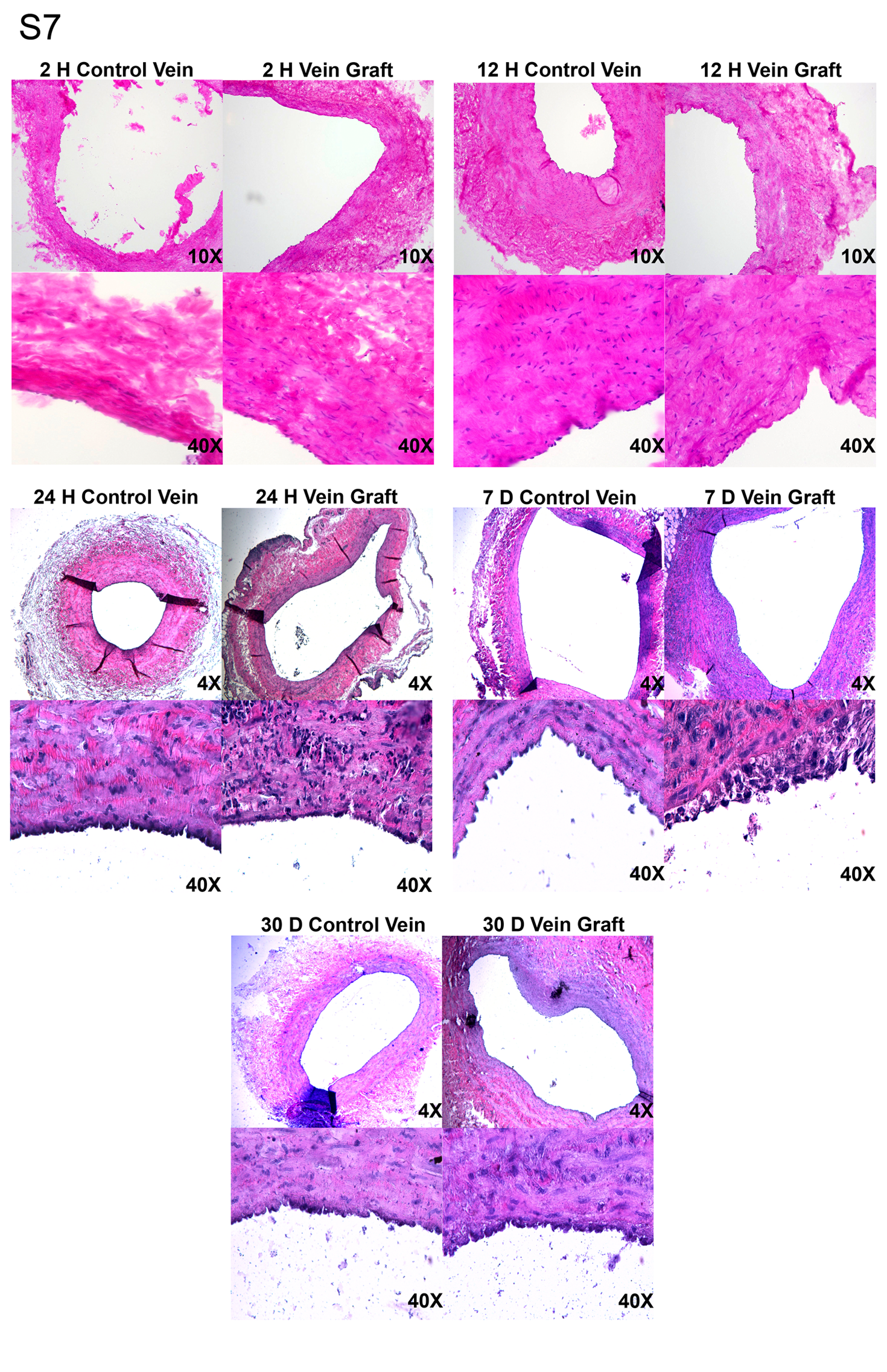

Supplement: Figure S7 — Representative histology images of control vein and vein graft. H&E stained histological images at low (4X or 10X) and high (40X) magnification of control vein and vein graft at 2 H, 12 H, 24 H, 7 D and 30 D following implantation. (TIF) [file pone.0039123.s008.tif]

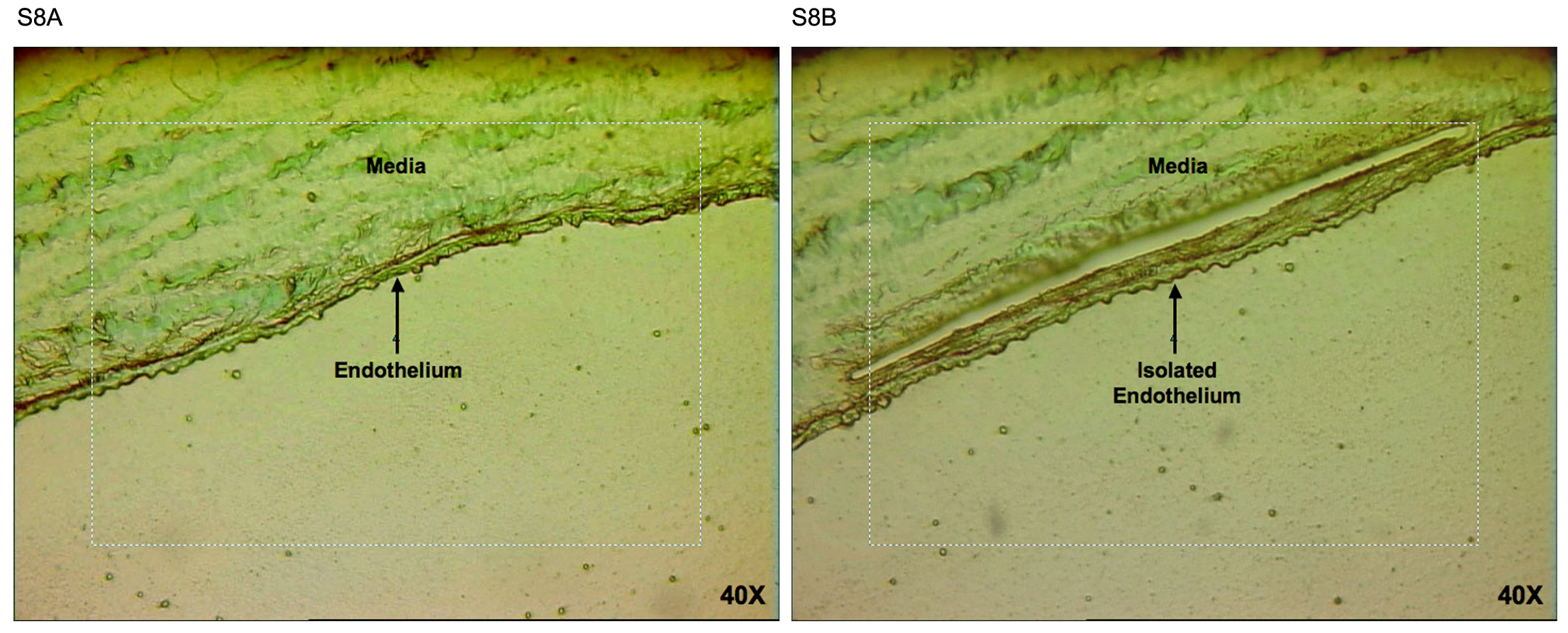

Supplement: Figure S8 — Representative Image of Laser Capture Mircodissection (LCM). A) Medial and endothelial layers in vein graft prior to LCM (Mag 40X), B) Isolated endothelial layer after LCM (Mag 40X). (TIF) [file pone.0039123.s009.tif]

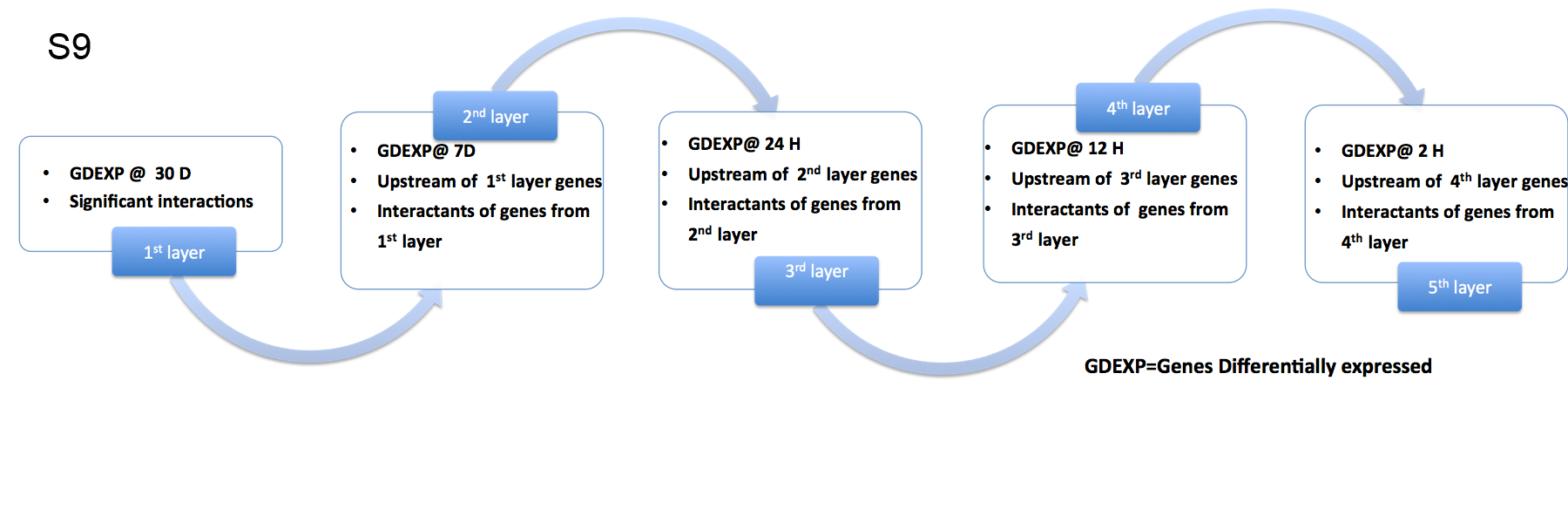

Supplement: Figure S9 — Workflow for generation of hierarchical back-propagation network. (TIF) [file pone.0039123.s010.tif]
