# Supplementary material for: Temporal Network Based Analysis of Cell Specific Vein Graft Transcriptome Defines Key Pathways and Hub Genes in Implantation Injury
Source: PLoS One. 2012 Jun 15;7(6):e39123. doi: 10.1371/journal.pone.0039123 (PMC3376111; doi:10.1371/journal.pone.0039123)
Supplement: Table S1 — List of Q-RT-PCR Primers. (DOCX) [file pone.0039123.s011.docx]

| **Gene** | **Forward (5’- 3’)** | **Reverse (5’- 3’)** |
| --- | --- | --- |
| 18S | CGG CTA CCA CAT CCA AGG AA | GCT GGA ATT ACC GCG GCT |
| CD31 | AGA TGT CCA GGC CAG CAG TA | TTG GGG TCT AAC ATC TTC |
| SMMHC II | CAG AAA AGC AAG GCT TCG AG | CAA TCA CCT CAT CCC CTT TC |
| IL-6 | ATG AAG TGA CCA CTC CTG ACC CAA | TGC CCA GGCTAC ATT ATC CGA ACA |
| IL-8 | ACA TGA CTT CCA AGC TGG CTG TTG | ATG TGG GCC ACT GTC AAT CAC TCT |
| Col11A1 | AGG TCC TCA AGG TAT CTC AGG GAA | GAG CTC CAG GAA GAC CTC TTT CAC |

**Table S1:** List of Q-RT-PCR Primers
