# Supplementary material for: Temporal Network Based Analysis of Cell Specific Vein Graft Transcriptome Defines Key Pathways and Hub Genes in Implantation Injury
Source: PLoS One. 2012 Jun 15;7(6):e39123. doi: 10.1371/journal.pone.0039123 (PMC3376111; doi:10.1371/journal.pone.0039123)
Supplement: Table S2 — List of unique differentially expressed genes identified by comparing graft vs. control vein EC and SMC at individual time points (2, 12, 24 H, and 7 and 30 D). The table represents fold change of significantly dysregulated genes from each zone of the Venn diagram shown in Figure S2. (PDF) [file pone.0039123.s012.pdf]

Table S2: List of unique differentially expressed genes

		EC- Fold Change					SMC-Fold Change				
**Gene SYMBOL**	**EN2H**	**EN12H**	**EN24H**	**EN7D**	**EN30D**	**SM2H**	**SM12H**	**SM24H**	**SM7D**	**SM30D**	**Venn Diagram Zones**
**SLC22A1**		-2.101	-3.038	-2.653			-1.288	-2.828	-3.377	-2.277	EN.SM.Down.12H.24H.7D, SM.Only.Down.30D
**ENPP2**				-2.86	-3.521		-1.757	-3.411	-2.492	-2.626	EN.SM.Down.7D.30D, SM.Only.Down.12H.24H
**BTC**		-2.282	-3.677		-3.267		-2.579	-3.137	-3.068		EN.Only.Down.30D, EN.SM.Down.12H.24H, SM.Only.Down.7D
**HPSE2**		-3.063	-2.812		-2.448		-3.301	-2.563	-1.38		EN.Only.Down.30D, EN.SM.Down.12H.24H, SM.Only.Down.7D
**CCDC88C**	-1.829	-2.05	-1.581	-2.022			-2.121		-1.525		EN.Only.Down.2H.24H, EN.SM.Down.12H.7D
**ADHFE1**		-2.009	-1.695	-1.151			-1.697	-1.523	-1.11		EN.SM.Down.12H.24H.7D
**AGPHD1**		-2.666	-2.898	-1.441			-3.862	-2.548	-1.538		EN.SM.Down.12H.24H.7D
**AKAP6**		-3.406	-2.316	-2.513			-2.685	-2.589	-1.595		EN.SM.Down.12H.24H.7D
**ALDH7A1**		-1.858	-2.045	-1.461			-1.947	-1.379	-1.514		EN.SM.Down.12H.24H.7D
**AMIGO2**		-3.871	-3.426	-3.58			-4.953	-3.685	-3.312		EN.SM.Down.12H.24H.7D
**ANGPTL1**		-4.231	-4.031	-2.566			-3.667	-2.873	-1.876		EN.SM.Down.12H.24H.7D
**ARMC4**		-2.237	-2.315	-1.991			-2.422	-2.429	-1.394		EN.SM.Down.12H.24H.7D
**CALCOCO1**		-2.711	-2.676	-1.611			-3.295	-3.493	-1.687		EN.SM.Down.12H.24H.7D
**CCDC3**		-1.77	-2.194	-3.223			-1.43	-1.718	-1.595		EN.SM.Down.12H.24H.7D
**CHN1**		-2.185	-1.768	-1.289			-3.353	-1.985	-1.337		EN.SM.Down.12H.24H.7D
**CKM**		-3.385	-3.955	-3.134			-3.478	-3.514	-1.453		EN.SM.Down.12H.24H.7D
**COCH**		-1.652	-3.11	-3.741			-1.809	-2.6	-2.929		EN.SM.Down.12H.24H.7D
**COL14A1**		-2.558	-3.852	-3.339			-2.179	-1.995	-1.702		EN.SM.Down.12H.24H.7D
**DES**		-1.952	-2.237	-1.777			-1.969	-1.724	-1.463		EN.SM.Down.12H.24H.7D
**DMD**		-2.405	-2.916	-2.899			-2.349	-2.448	-2.59		EN.SM.Down.12H.24H.7D
**DTNA**		-2.832	-3.397	-3.438			-2.723	-2.881	-2.981		EN.SM.Down.12H.24H.7D
**EBF1**		-2.329	-2.423	-2.092			-2.119	-1.852	-1.354		EN.SM.Down.12H.24H.7D
**FHL1**		-2.736	-2.968	-1.438			-3.346	-2.169	-1.215		EN.SM.Down.12H.24H.7D
**FILIP1L**		-2.193	-1.946	-1.324			-2.287	-2.533	-1.633		EN.SM.Down.12H.24H.7D
**FN3K**		-3.748	-3.455	-2.167			-3.642	-3.986	-2.382		EN.SM.Down.12H.24H.7D
**GNAO1**		-1.793	-1.412	-1.329			-2.087	-1.685	-1.163		EN.SM.Down.12H.24H.7D
**GPM6A**		-4.068	-3.047	-1.977			-4.158	-2.459	-2.637		EN.SM.Down.12H.24H.7D
**GSN**		-1.491	-2.028	-1.984			-1.254	-1.504	-2.56		EN.SM.Down.12H.24H.7D
**GULP1**		-2.119	-2.264	-2.079			-1.873	-2.05	-1.153		EN.SM.Down.12H.24H.7D
**HSPA2**		-2.305	-2.507	-1.202			-2.872	-2.203	-1.214		EN.SM.Down.12H.24H.7D
**ITIH1**		-3.592	-5.244	-2.539			-3.207	-5.17	-3.658		EN.SM.Down.12H.24H.7D
**ITPR1**		-2.012	-1.824	-1.931			-2.46	-2.206	-1.14		EN.SM.Down.12H.24H.7D
**KCNMB3**		-2.49	-2.149	-2.648			-2.058	-2.97	-2.095		EN.SM.Down.12H.24H.7D
**LAMC1**		-1.995	-1.938	-1.032			-1.679	-1.914	-1.706		EN.SM.Down.12H.24H.7D
**LIN7B**		-1.375	-1.991	-1.889			-2.298	-2.438	-1.782		EN.SM.Down.12H.24H.7D
**LOC478802**		-1.46	-1.862	-1.726			-1.659	-1.322	-1.657		EN.SM.Down.12H.24H.7D
**LOC481389**		-3.499	-2.955	-1.8			-3.672	-4.075	-1.148		EN.SM.Down.12H.24H.7D
**LOC482906**		-2.488	-2.472	-1.661			-3.673	-2.354	-1.445		EN.SM.Down.12H.24H.7D
**LOC482907**		-2.426	-2.366	-1.729			-1.884	-2.094	-1.228		EN.SM.Down.12H.24H.7D
**LOC488991**		-3.054	-2.879	-2.506			-3.073	-2.346	-1.711		EN.SM.Down.12H.24H.7D
**LOC607380**		-3.446	-2.988	-2.078			-2.464	-2.196	-2.228		EN.SM.Down.12H.24H.7D
**LOC607460**		-3.057	-2.541	-1.271			-3.302	-2.871	-1.303		EN.SM.Down.12H.24H.7D
**LOC609117**		-2.422	-2.911	-2.92			-2.519	-3.133	-2.778		EN.SM.Down.12H.24H.7D
**LOC611771**		-1.96	-2.308	-2.489			-2.55	-2.768	-2.066		EN.SM.Down.12H.24H.7D
**MCAM**		-2.488	-2.127	-2.358			-2.735	-1.977	-1.756		EN.SM.Down.12H.24H.7D
**ME1**		-2.039	-1.836	-1.352			-2.167	-2.034	-1.075		EN.SM.Down.12H.24H.7D
**MXI1**		-1.955	-1.878	-1.698			-2.437	-2.85	-1.343		EN.SM.Down.12H.24H.7D
**MYOM1**		-3.757	-4.079	-3.001			-3.989	-3.623	-1.641		EN.SM.Down.12H.24H.7D
**NDE1**		-2.556	-2.822	-2.338			-2.611	-2.327	-1.391		EN.SM.Down.12H.24H.7D
**NDRG2**		-3.176	-2.749	-2.743			-2.94	-2.289	-2.323		EN.SM.Down.12H.24H.7D
**NTF3**		-1.346	-2.122	-2.519			-2.285	-2.272	-2.411		EN.SM.Down.12H.24H.7D
**NTRK3**		-3.062	-2.705	-2.568			-2.792	-3.885	-2.415		EN.SM.Down.12H.24H.7D
**PCGF5**		-1.357	-1.615	-1.788			-1.169	-1.922	-1.63		EN.SM.Down.12H.24H.7D
**PDE5A**		-2.702	-3.04	-2.794			-2.837	-3.054	-2.187		EN.SM.Down.12H.24H.7D
**PGM5**		-2.507	-2.506	-1.561			-2.287	-1.908	-1.186		EN.SM.Down.12H.24H.7D
**PHYHIPL**		-2.728	-3.44	-2.303			-2.89	-2.178	-1.849		EN.SM.Down.12H.24H.7D
**PKIG**		-3.064	-2.572	-1.196			-2.285	-2.231	-1.664		EN.SM.Down.12H.24H.7D
**PLN**		-4.399	-3.969	-2.628			-5.214	-3.889	-2.078		EN.SM.Down.12H.24H.7D
**PPP1R12B**		-3.158	-3.106	-2.791			-3.817	-2.925	-2.483		EN.SM.Down.12H.24H.7D
**PPP1R3C**		-3.211	-2.462	-1.397			-3.624	-2.867	-1.307		EN.SM.Down.12H.24H.7D
**PPP1R9A**		-2.085	-1.706	-1.844			-2.67	-1.591	-1.897		EN.SM.Down.12H.24H.7D
**RAB9B**		-2.229	-1.664	-1.672			-2.766	-1.746	-1.126		EN.SM.Down.12H.24H.7D
**RBPMS**		-1.54	-2.66	-1.489			-1.865	-1.487	-1.537		EN.SM.Down.12H.24H.7D
**RYR2**		-1.725	-2.353	-2.368			-2.173	-2.915	-2.058		EN.SM.Down.12H.24H.7D
**SCP2**		-2.521	-2.707	-2.946			-1.844	-2.282	-1.57		EN.SM.Down.12H.24H.7D
**SCUBE3**		-2.155	-1.858	-2.126			-1.901	-1.278	-1.93		EN.SM.Down.12H.24H.7D
**SGCG**		-2.337	-2.263	-3.666			-2.829	-2.121	-2.854		EN.SM.Down.12H.24H.7D
**SH3RF2**		-3.393	-2.625	-1.546			-3.368	-2.805	-1.975		EN.SM.Down.12H.24H.7D
**SIAE**		-1.652	-2.53	-2.004			-1.989	-1.695	-2.174		EN.SM.Down.12H.24H.7D
**SLC22A3**		-3.319	-3.937	-3.462			-3.751	-4.46	-2.789		EN.SM.Down.12H.24H.7D
**SOX5**		-3.321	-2.522	-1.977			-3.155	-2.572	-1.161		EN.SM.Down.12H.24H.7D
**SVIL**		-1.862	-1.575	-1.129			-2.086	-2.408	-1.016		EN.SM.Down.12H.24H.7D
**SYNPO2**		-2.924	-3.244	-2.334			-3.08	-2.284	-1.883		EN.SM.Down.12H.24H.7D
**TCF7L2**		-2.039	-1.681	-1.504			-2.088	-1.904	-1.22		EN.SM.Down.12H.24H.7D
**TESC**		-2.824	-3.574	-2.992			-3.187	-3.346	-1.182		EN.SM.Down.12H.24H.7D
**TIMP3**		-2.525	-2.692	-1.758			-2.757	-3.063	-1.644		EN.SM.Down.12H.24H.7D
**TMEM63C**		-2.203	-2.27	-2.12			-2.425	-1.92	-1.965		EN.SM.Down.12H.24H.7D
**VAMP2**		-2.061	-1.782	-2.473			-2.016	-1.434	-2.429		EN.SM.Down.12H.24H.7D
**ZMAT1**		-2.112	-2.168	-2.249			-1.925	-2.572	-1.94		EN.SM.Down.12H.24H.7D
**C9orf5**		-2.13	-2.015	-1.75			1.022	-2.558	-1.759		EN.SM.Down.12H.24H.7D, SM.Only.Up.12H
**CYP21A**		-1.384	-2.017	-2.753				-1.431	-4.014		EN.Only.Down.12H, EN.SM.Down.24H.7D
**EPB41L4B**		-3.413	-1.976	-2.394				-2.259	-1.484		EN.Only.Down.12H, EN.SM.Down.24H.7D
**SFRP4**		-1.747	-3.03	-2.64				-2.19	-2.235		EN.Only.Down.12H, EN.SM.Down.24H.7D
**ADRA2B**		-2.146	-1.438	-2.271			-2.692		-1.86		EN.Only.Down.24H, EN.SM.Down.12H.7D
**ASB2**		-3.33	-1.904	-2.158			-3.501		-2.144		EN.Only.Down.24H, EN.SM.Down.12H.7D
**ATP2A2**		-1.518	-1.988	-1.803			-1.197		-1.391		EN.Only.Down.24H, EN.SM.Down.12H.7D
**CSRP1**		-1.832	-1.811	-1.445			-2.139		-1.794		EN.Only.Down.24H, EN.SM.Down.12H.7D
**EFHA2**		-1.237	-1.3	-1.577			-1.925		-1.41		EN.Only.Down.24H, EN.SM.Down.12H.7D
**EFHD1**		-2.297	-1.59	-1.3			-2.409		-1.867		EN.Only.Down.24H, EN.SM.Down.12H.7D
**ENPP6**		-2.051	-2.573	-6.141			-1.897		-4.356		EN.Only.Down.24H, EN.SM.Down.12H.7D
**FGF13**		-2.285	-2.396	-1.534			-1.75		-1.144		EN.Only.Down.24H, EN.SM.Down.12H.7D
**FLNA**		-1.355	-1.627	-1.017			-1.874		-1.693		EN.Only.Down.24H, EN.SM.Down.12H.7D
**FRMD5**		-2.653	-1.865	-2.465			-1.697		-2.033		EN.Only.Down.24H, EN.SM.Down.12H.7D
**GOT1**		-1.991	-1.48	-1.709			-2.263		-1.665		EN.Only.Down.24H, EN.SM.Down.12H.7D
**GPRC5B**		-1.708	-1.633	-2.083			-1.568		-1.756		EN.Only.Down.24H, EN.SM.Down.12H.7D
**GUCY1A3**		-2.518	-2.855	-2.31			-3.484		-1.434		EN.Only.Down.24H, EN.SM.Down.12H.7D
**INADL**		-2.633	-1.739	-1.91			-2.147		-1.991		EN.Only.Down.24H, EN.SM.Down.12H.7D
**KLHL32**		-3.281	-2.63	-1.736			-3.442		-1.119		EN.Only.Down.24H, EN.SM.Down.12H.7D
**LGI1**		-4.037	-4.641	-3.901			-4.558		-4.238		EN.Only.Down.24H, EN.SM.Down.12H.7D
**LOC484118**		-1.893	-2.339	-1.862			-1.852		-2.144		EN.Only.Down.24H, EN.SM.Down.12H.7D
**LOC490237**		-2.282	-2.133	-1.66			-2.831		-1.981		EN.Only.Down.24H, EN.SM.Down.12H.7D
**MGLL**		-2.528	-2.303	-1.894			-2.649		-2.158		EN.Only.Down.24H, EN.SM.Down.12H.7D
**NEXN**		-1.316	-1.395	-2.551			-1.523		-1.83		EN.Only.Down.24H, EN.SM.Down.12H.7D
**PBXIP1**		-2.391	-1.25	-1.596			-2.946		-2.455		EN.Only.Down.24H, EN.SM.Down.12H.7D
**PDZRN4**		-3.756	-2.608	-2.976			-3.969		-2.404		EN.Only.Down.24H, EN.SM.Down.12H.7D
**PLA2G16**		-1.751	-1.879	-1.709			-1.172		-1.469		EN.Only.Down.24H, EN.SM.Down.12H.7D
**PLCE1**		-2.789	-2.416	-2.087			-2.801		-1.373		EN.Only.Down.24H, EN.SM.Down.12H.7D
**SFRS5**		-1.015	-1.073	-1.049			-1.381		-1.037		EN.Only.Down.24H, EN.SM.Down.12H.7D
**SKI**		-1.145	-1.427	-1.232			-1.203		-1.596		EN.Only.Down.24H, EN.SM.Down.12H.7D
**SLMAP**		-1.462	-2.025	-2.347			-2.455		-1.51		EN.Only.Down.24H, EN.SM.Down.12H.7D
**ST5**		-1.443	-1.893	-1.353			-1.239		-1.536		EN.Only.Down.24H, EN.SM.Down.12H.7D
**STARD9**		-1.676	-1.336	-1.251			-1.291		-1.068		EN.Only.Down.24H, EN.SM.Down.12H.7D
**TGFBR3**		-2.864	-1.619	-1.559			-2.243		-2.339		EN.Only.Down.24H, EN.SM.Down.12H.7D
**TMOD2**		-2.322	-1.941	-3.05			-2.322		-2.472		EN.Only.Down.24H, EN.SM.Down.12H.7D
**TTLL7**		-2.006	-1.215	-1.024			-2.187		-1.15		EN.Only.Down.24H, EN.SM.Down.12H.7D
**CCDC107**	-2.736	-1.98	-2.56				-1.932	-2.575			EN.Only.Down.2H, EN.SM.Down.12H.24H
**ADRA1A**	-3.092	-3.028		-2.409			-2.981		-2.506		EN.Only.Down.2H, EN.SM.Down.12H.7D
**C1QTNF7**		-2.138	-2.614	-2.328			-1.79	-2.852			EN.Only.Down.7D, EN.SM.Down.12H.24H
**CAP2**		-1.431	-1.647	-1.482			-1.29	-1.302			EN.Only.Down.7D, EN.SM.Down.12H.24H
**DST**		-1.694	-1.78	-1.009			-2.08	-1.792			EN.Only.Down.7D, EN.SM.Down.12H.24H
**ECM2**		-2.586	-3.242	-2.154			-2.09	-2.722			EN.Only.Down.7D, EN.SM.Down.12H.24H

**FXYD1**		-2.32	-2.853	-1.653			-2.061	-2.524			EN.Only.Down.7D, EN.SM.Down.12H.24H
**GCOM1**		-1.881	-1.509	-1.23			-2.724	-2.203			EN.Only.Down.7D, EN.SM.Down.12H.24H
**GSTT1**		-2.322	-2.664	-1.132			-1.947	-1.883			EN.Only.Down.7D, EN.SM.Down.12H.24H
**LOC612293**		-2.634	-2.158	-1.411			-2.973	-3.162			EN.Only.Down.7D, EN.SM.Down.12H.24H
**LOC612466**		-2.192	-3.687	-1.993			-2.953	-3.166			EN.Only.Down.7D, EN.SM.Down.12H.24H
**MACF1**		-1.151	-1.387	-1.259			-1.441	-2.206			EN.Only.Down.7D, EN.SM.Down.12H.24H
**MFAP4**		-2.636	-3.24	-2.657			-2.3	-2.455			EN.Only.Down.7D, EN.SM.Down.12H.24H
**NPNT**		-2.678	-2.704	-1.703			-3.171	-2.585			EN.Only.Down.7D, EN.SM.Down.12H.24H
**NR1D2**		-1.211	-1.169	-1.004			-1.825	-2.884			EN.Only.Down.7D, EN.SM.Down.12H.24H
**PDE6H**		-4.71	-2.831	-3.726			-3.97	-1.726			EN.Only.Down.7D, EN.SM.Down.12H.24H
**SAT2**		-2.63	-2.966	-1.452			-2.797	-3.439			EN.Only.Down.7D, EN.SM.Down.12H.24H
**SEPP1**		-5.876	-4.029	-2.383			-3.654	-3.067			EN.Only.Down.7D, EN.SM.Down.12H.24H
**SGCA**		-2.972	-2.828	-1.586			-2.77	-3.048			EN.Only.Down.7D, EN.SM.Down.12H.24H
**SPA17**		-1.751	-1.974	-1.355			-1.721	-1.912			EN.Only.Down.7D, EN.SM.Down.12H.24H
**SPARCL1**		-2.046	-2.504	-1.724			-1.441	-1.643			EN.Only.Down.7D, EN.SM.Down.12H.24H
**SYTL2**		-1.892	-3.063	-2.07			-2.93	-2.797			EN.Only.Down.7D, EN.SM.Down.12H.24H
**TSPAN7**		-2.408	-3.227	-1.58			-1.514	-2.156			EN.Only.Down.7D, EN.SM.Down.12H.24H
**ZNF331**		-2.101	-2.11	-1.5			-1.638	-2.192			EN.Only.Down.7D, EN.SM.Down.12H.24H
**CCK**		-4.187	-3.801				-4.924	-4.804	-2.762		EN.SM.Down.12H.24H, SM.Only.Down.7D
**CDKN1B**		-2.036	-1.942				-1.907	-2.076	-1.289		EN.SM.Down.12H.24H, SM.Only.Down.7D
**DMPK**		-2.061	-2.746				-2.355	-2.444	-1.099		EN.SM.Down.12H.24H, SM.Only.Down.7D
**EPB41L2**		-1.848	-2.059				-2.358	-1.33	-1.639		EN.SM.Down.12H.24H, SM.Only.Down.7D
**GHR**		-2.312	-1.824				-2.276	-1.199	-1.188		EN.SM.Down.12H.24H, SM.Only.Down.7D
**GM2A**		-1.76	-2.404				-1.421	-2.144	-1.543		EN.SM.Down.12H.24H, SM.Only.Down.7D
**IGFBP5**		-1.426	-2.342				-1.142	-2.048	-1.888		EN.SM.Down.12H.24H, SM.Only.Down.7D
**ITIH3**		-2.209	-2.039				-1.855	-1.665	-1.449		EN.SM.Down.12H.24H, SM.Only.Down.7D
**MAMDC2**		-2.718	-3.649				-2.806	-3.434	-2.134		EN.SM.Down.12H.24H, SM.Only.Down.7D
**MOSPD2**		-1.132	-1.865				-2.13	-1.646	-1.134		EN.SM.Down.12H.24H, SM.Only.Down.7D
**PCBD1**		-2.33	-2.26				-2.038	-1.647	-1.564		EN.SM.Down.12H.24H, SM.Only.Down.7D
**PCMTD2**		-1.158	-1.241				-1.373	-1.648	-1.013		EN.SM.Down.12H.24H, SM.Only.Down.7D
**SMTN**		-1.42	-2.148				-1.358	-1.954	-1.553		EN.SM.Down.12H.24H, SM.Only.Down.7D
**SYNE2**		-3.41	-1.914				-3.041	-1.704	-1.169		EN.SM.Down.12H.24H, SM.Only.Down.7D
**TPM1**		-2.112	-2.102				-2.532	-2.151	-1.737		EN.SM.Down.12H.24H, SM.Only.Down.7D
**LMO3**		-2.611		-1.268			-3.334	-2.354	-1.301		EN.SM.Down.12H.7D, SM.Only.Down.24H
**PER2**		-2.686		-1.939			-2.735	-3.55	-2.57		EN.SM.Down.12H.7D, SM.Only.Down.24H
**TMEM59L**		-1.34		-1.538			-1.798	-1.419	-1.283		EN.SM.Down.12H.7D, SM.Only.Down.24H
**TRPV1**		-1.856		-1.511			-2.889	-3.25	-1.197		EN.SM.Down.12H.7D, SM.Only.Down.24H
**CEP72**			-1.813	-1.023			-1.457	-2.068	-1.087		EN.SM.Down.24H.7D, SM.Only.Down.12H
**cOR52E8**			-1.661	-1.006			-1.709	-1.876	-1.694		EN.SM.Down.24H.7D, SM.Only.Down.12H
**KCNA5**			-3.437	-3.214			-2.25	-1.993	-2.131		EN.SM.Down.24H.7D, SM.Only.Down.12H
**KCNMA1**			-2.654	-2.942			-2.169	-1.674	-2.546		EN.SM.Down.24H.7D, SM.Only.Down.12H
**KLHDC8B**			-2.649	-2.964			-1.955	-2.401	-2.302		EN.SM.Down.24H.7D, SM.Only.Down.12H
**PDE8B**			-1.489	-1.422			-1.933	-1.841	-1.805		EN.SM.Down.24H.7D, SM.Only.Down.12H
**PIP5K1B**			-2.624	-2.441			-1.301	-3.377	-1.798		EN.SM.Down.24H.7D, SM.Only.Down.12H
**SVIP**			-1.711	-2.16			-2.302	-1.493	-1.946		EN.SM.Down.24H.7D, SM.Only.Down.12H
**FILIP1**	2.306	-2.047	-1.406	-2.071			-1.842				EN.Only.Down.24H.7D, EN.SM.Down.12H, EN.Only.Up.2H
**LAMA3**		-2.062	-2.971	-1.15			-2.133		1.016		EN.Only.Down.24H.7D, EN.SM.Down.12H, SM.Only.Up.7D
**KDM2B**		-1.803		1.093			-1.894	-1.857	-1.261		EN.SM.Down.12H, SM.Only.Down.24H.7D, EN.Only.Up.7D
**SYNE1**		-1.812	1.186				-2.388	-2.186	-2.436		EN.SM.Down.12H.24H, SM.Only.Down.7D, EN.Only.Up.24H
**ASPN**		-3.073	-3.664				-2.256	-3.057	1.482		EN.SM.Down.12H.24H, SM.Only.Up.7D
**LOC607395**		-2.435	-2.257				-2.347	-2.867	1.039		EN.SM.Down.12H.24H, SM.Only.Up.7D
**HPGDS**			-2.314	-3.333	-2.675				-1.368		EN.Only.Down.24H.30D, EN.SM.Down.7D
**HPGD**			-2.314	-3.333	-2.675				-1.898		SM.Only.Down.7D
**ICA1**		-2.342	-1.455				-1.482	-2.509			EN.Only.Down.12H.24H
**ARHGEF3**		-1.528	-2.158	-1.468					-1.038		EN.Only.Down.12H.24H, EN.SM.Down.7D
**LDB2**		-1.885	-1.749	-1.328					-1.234		EN.Only.Down.12H.24H, EN.SM.Down.7D
**NTRK2**		-1.289	-2.161	-3.567					-2.694		EN.Only.Down.12H.24H, EN.SM.Down.7D
**USP11**		-1.07	-1.262	-1.094					-1.222		EN.Only.Down.12H.24H, EN.SM.Down.7D
**ANK2**		-2.049	-2.285				-2.286		-1.257		EN.Only.Down.24H, EN.SM.Down.12H, SM.Only.Down.7D
**ANKRD10**		-1.295	-1.858				-1.425		-1.058		EN.Only.Down.24H, EN.SM.Down.12H, SM.Only.Down.7D
**BICD1**		-1.597	-1.904				-2.436		-1.752		EN.Only.Down.24H, EN.SM.Down.12H, SM.Only.Down.7D
**FEZ1**		-2.402	-2.372				-1.996		-1.696		EN.Only.Down.24H, EN.SM.Down.12H, SM.Only.Down.7D
**HOXA7**		-3.177	-2.273				-1.753		-1.691		EN.Only.Down.24H, EN.SM.Down.12H, SM.Only.Down.7D
**INPP5A**		-1.341	-1.419				-1.485		-1.01		EN.Only.Down.24H, EN.SM.Down.12H, SM.Only.Down.7D
**KCNS3**		-1.797	-1.831				-1.702		-1.556		EN.Only.Down.24H, EN.SM.Down.12H, SM.Only.Down.7D
**LOC478991**		-1.399	-1.412				-1.917		-1.704		EN.Only.Down.24H, EN.SM.Down.12H, SM.Only.Down.7D
**LOC607351**		-1.001	-1.074				-1.36		-1.33		EN.Only.Down.24H, EN.SM.Down.12H, SM.Only.Down.7D
**MOBKL1A**		-1.729	-1.348				-1.492		-1.194		EN.Only.Down.24H, EN.SM.Down.12H, SM.Only.Down.7D
**NFIA**		-1.93	-1.601				-1.733		-1.012		EN.Only.Down.24H, EN.SM.Down.12H, SM.Only.Down.7D
**PLCB4**		-2.675	-2.163				-3.02		-1.92		EN.Only.Down.24H, EN.SM.Down.12H, SM.Only.Down.7D
**SHROOM3**		-1.234	-1.757				-1.721		-2.152		EN.Only.Down.24H, EN.SM.Down.12H, SM.Only.Down.7D
**TAGLN**		-1.654	-1.601				-1.856		-1.177		EN.Only.Down.24H, EN.SM.Down.12H, SM.Only.Down.7D
**TCTEX1D2**		-1.861	-1.151				-1.448		-1.304		EN.Only.Down.24H, EN.SM.Down.12H, SM.Only.Down.7D
**TENC1**		-1.528	-1.99				-1.694		-1.482		EN.Only.Down.24H, EN.SM.Down.12H, SM.Only.Down.7D
**ZBTB4**		-1.422	-1.43				-1.131		-1.233		EN.Only.Down.24H, EN.SM.Down.12H, SM.Only.Down.7D
**BCL9**			-2.217	-1.174			-1.054		-1.558		EN.Only.Down.24H, EN.SM.Down.7D, SM.Only.Down.12H
**CNN1**			-1.146	-1.858			-1.494		-2.301		EN.Only.Down.24H, EN.SM.Down.7D, SM.Only.Down.12H
**DNAJB5**			-2.224	-2.199			-1.688		-1.797		EN.Only.Down.24H, EN.SM.Down.7D, SM.Only.Down.12H
**KCND3**			-2.449	-2.29			-1.721		-2.329		EN.Only.Down.24H, EN.SM.Down.7D, SM.Only.Down.12H
**LOC487765**			-3.059	-3.108			-1.931		-1.856		EN.Only.Down.24H, EN.SM.Down.7D, SM.Only.Down.12H
**LOC489360**			-1.734	-1.903			-1.179		-1.341		EN.Only.Down.24H, EN.SM.Down.7D, SM.Only.Down.12H
**PTGDS**			-1.548	-1.606			-1.21		-1.649		EN.Only.Down.24H, EN.SM.Down.7D, SM.Only.Down.12H
**PXDNL**			-2.078	-1.746			-1.775		-1.339		EN.Only.Down.24H, EN.SM.Down.7D, SM.Only.Down.12H
**RORB**			-2.331	-2.324			-1.951		-1.501		EN.Only.Down.24H, EN.SM.Down.7D, SM.Only.Down.12H
**SLC8A1**			-2.642	-1.925			-2.117		-1.934		EN.Only.Down.24H, EN.SM.Down.7D, SM.Only.Down.12H
**SLIT2**			-1.738	-2.206			-1.254		-1.564		EN.Only.Down.24H, EN.SM.Down.7D, SM.Only.Down.12H
**ABI2**		-1.289	-1.361	-1.004			-1.266				EN.Only.Down.24H.7D, EN.SM.Down.12H
**ABLIM1**		-2.372	-2.582	-1.486			-2.369				EN.Only.Down.24H.7D, EN.SM.Down.12H
**BHMT**		-2.467	-2.677	-2.482			-2.266				EN.Only.Down.24H.7D, EN.SM.Down.12H
**CLYBL**		-1.799	-2.486	-1.465			-2.171				EN.Only.Down.24H.7D, EN.SM.Down.12H
**CPE**		-1.447	-1.998	-2.167			-1.61				EN.Only.Down.24H.7D, EN.SM.Down.12H
**EDNRA**		-1.841	-1.811	-1.69			-1.728				EN.Only.Down.24H.7D, EN.SM.Down.12H
**FHOD3**		-3.834	-3.276	-2.159			-2.26				EN.Only.Down.24H.7D, EN.SM.Down.12H
**FMOD**		-1.959	-1.95	-1.868			-1.303				EN.Only.Down.24H.7D, EN.SM.Down.12H
**GUCY1B3**		-2.147	-2.278	-1.456			-2.525				EN.Only.Down.24H.7D, EN.SM.Down.12H
**LOC608636**		-2.391	-2.513	-1.357			-1.028				EN.Only.Down.24H.7D, EN.SM.Down.12H
**MAOB**		-2.729	-2.547	-1.209			-2.09				EN.Only.Down.24H.7D, EN.SM.Down.12H
**MEOX2**		-2.004	-2.026	-1.213			-1.954				EN.Only.Down.24H.7D, EN.SM.Down.12H
**MYH11**		-1.749	-2.267	-1.886			-1.965				EN.Only.Down.24H.7D, EN.SM.Down.12H
**MYO7B**		-1.027	-1.332	-1.106			-1.25				EN.Only.Down.24H.7D, EN.SM.Down.12H
**NKD1**		-1.002	-1.069	-1.332			-1.862				EN.Only.Down.24H.7D, EN.SM.Down.12H
**OLFML1**		-3.673	-2.635	-1.604			-3.856				EN.Only.Down.24H.7D, EN.SM.Down.12H
**PALMD**		-3.563	-2.686	-1.685			-2.076				EN.Only.Down.24H.7D, EN.SM.Down.12H
**PCDH7**		-1.809	-2.124	-1.291			-2.081				EN.Only.Down.24H.7D, EN.SM.Down.12H
**PGCP**		-2.342	-2.708	-1.86			-2.028				EN.Only.Down.24H.7D, EN.SM.Down.12H
**PRELP**		-1.904	-2.932	-2.397			-1.984				EN.Only.Down.24H.7D, EN.SM.Down.12H
**RAB4A**		-1.462	-1.057	-1.143			-1.531				EN.Only.Down.24H.7D, EN.SM.Down.12H
**RBMS3**		-1.891	-1.543	-1.734			-1.62				EN.Only.Down.24H.7D, EN.SM.Down.12H
**TCEAL1**		-1.332	-1.518	-1.195			-1.647				EN.Only.Down.24H.7D, EN.SM.Down.12H
**VPS13B**		-1.454	-1.603	-1.358			-1.675				EN.Only.Down.24H.7D, EN.SM.Down.12H
**CISD1**	-1.33	-1.341		-1.001			-1.89				EN.Only.Down.2H.7D, EN.SM.Down.12H
**MEF2C**		-1.985		-1.05			-2.742	-2.032			EN.Only.Down.7D, EN.SM.Down.12H, SM.Only.Down.24H
**PPFIBP2**		-1.473		-1.505			-2.357	-2.743			EN.Only.Down.7D, EN.SM.Down.12H, SM.Only.Down.24H
**SCUBE2**		-2.614		-1.524			-2.188	-2.46			EN.Only.Down.7D, EN.SM.Down.12H, SM.Only.Down.24H
**SGIP1**		-1.974		-2.051			-1.843	-2.6			EN.Only.Down.7D, EN.SM.Down.12H, SM.Only.Down.24H
**SH3D19**		-2.1		-1.048			-2.289	-1.538			EN.Only.Down.7D, EN.SM.Down.12H, SM.Only.Down.24H
**TCTN2**		-2.378		-1.642			-2.598	-1.559			EN.Only.Down.7D, EN.SM.Down.12H, SM.Only.Down.24H
**RGN**			-1.238	-2.107			-3.448	-2.483			EN.Only.Down.7D, EN.SM.Down.24H, SM.Only.Down.12H
**ANK3**		-1.188					-1.98	-1.419	-1.97		EN.SM.Down.12H, SM.Only.Down.24H.7D
**LOC479837**		-1.247					-1.228	-1.427	-1.239		EN.SM.Down.12H, SM.Only.Down.24H.7D
**LPL**		-1.958					-2.568	-1.891	-1.32		EN.SM.Down.12H, SM.Only.Down.24H.7D
**MFAP3L**		-1.394					-2.061	-1.739	-2.498		EN.SM.Down.12H, SM.Only.Down.24H.7D
**MPDZ**		-1.612					-1.8	-1.742	-1.418		EN.SM.Down.12H, SM.Only.Down.24H.7D

**PPARGC1A**		-2.454					-2.487	-3.001	-2.424		EN.SM.Down.12H, SM.Only.Down.24H.7D
**RPS6KA5**		-1.641					-3.207	-2.838	-1.38		EN.SM.Down.12H, SM.Only.Down.24H.7D
**A2M**		-1.881	-1.911				-1.858	-2.302			EN.SM.Down.12H.24H
**AGRN**		-1.651	-1.729				-1.881	-1.602			EN.SM.Down.12H.24H
**AIG1**		-2.321	-1.785				-2.848	-2.259			EN.SM.Down.12H.24H
**AKR1CL2**		-3.777	-2.264				-3.807	-3.242			EN.SM.Down.12H.24H
**ART4**		-2.291	-2.549				-2.341	-1.917			EN.SM.Down.12H.24H
**CABC1**		-2.914	-2.439				-1.868	-2.429			EN.SM.Down.12H.24H
**CFI**		-2.353	-2.804				-1.881	-2.103			EN.SM.Down.12H.24H
**CHMP4C**		-2.525	-2.272				-3.251	-1.72			EN.SM.Down.12H.24H
**CTSF**		-1.818	-1.684				-1.459	-1.375			EN.SM.Down.12H.24H
**DDX26B**		-1.363	-2.046				-2.222	-2.445			EN.SM.Down.12H.24H
**DUT**		-1.407	-1.469				-2.136	-1.683			EN.SM.Down.12H.24H
**ENPP5**		-1.103	-1.235				-1.6	-1.606			EN.SM.Down.12H.24H
**FAHD2A**		-2.188	-1.223				-2.221	-1.766			EN.SM.Down.12H.24H
**FBXL20**		-1.064	-1.085				-1.29	-1.937			EN.SM.Down.12H.24H
**GLRB**		-3.2	-2.093				-2.902	-2.375			EN.SM.Down.12H.24H
**GOLIM4**		-2.05	-1.857				-2.208	-1.957			EN.SM.Down.12H.24H
**GSTA4**		-1.956	-1.908				-2.024	-1.658			EN.SM.Down.12H.24H
**HEYL**		-1.938	-1.551				-1.319	-2.02			EN.SM.Down.12H.24H
**HSD17B8**		-1.607	-1.298				-1.382	-1.501			EN.SM.Down.12H.24H
**ITSN1**		-1.836	-2.018				-2.004	-2.646			EN.SM.Down.12H.24H
**JAM2**		-3.709	-2.71				-1.738	-1.393			EN.SM.Down.12H.24H
**KLHL38**		-3.544	-3.559				-3.982	-3.975			EN.SM.Down.12H.24H
**LOC475615**		-3.868	-2.713				-3.351	-3.11			EN.SM.Down.12H.24H
**LOC476167**		-1.844	-1.292				-1.968	-1.504			EN.SM.Down.12H.24H
**LOC476578**		-1.615	-1.365				-1.655	-1.535			EN.SM.Down.12H.24H
**LOC478827**		-1.549	-1.761				-1.521	-1.254			EN.SM.Down.12H.24H
**LOC487557**		-2.152	-1.92				-2.132	-3.059			EN.SM.Down.12H.24H
**LOC607874**		-1.663	-1.26				-1.578	-1.22			EN.SM.Down.12H.24H
**LOC609239**		-1.574	-1.662				-1.114	-1.711			EN.SM.Down.12H.24H
**LOC611318**		-3.011	-2.076				-2.533	-2.703			EN.SM.Down.12H.24H
**LOC611359**		-2.386	-2.547				-2.603	-2.116			EN.SM.Down.12H.24H
**LRIG3**		-2.205	-2.343				-2.028	-1.9			EN.SM.Down.12H.24H
**MATN2**		-2.344	-2.535				-1.68	-1.879			EN.SM.Down.12H.24H
**MBNL1**		-1.309	-1.211				-1.839	-1.262			EN.SM.Down.12H.24H
**NET1**		-2.636	-2.082				-3.216	-2.594			EN.SM.Down.12H.24H
**P2RX1**		-1.679	-2.249				-1.947	-1.56			EN.SM.Down.12H.24H
**PBX1**		-2.409	-2.27				-2.045	-1.709			EN.SM.Down.12H.24H
**PDCD4**		-2.296	-1.814				-1.806	-1.5			EN.SM.Down.12H.24H
**PHKG1**		-1.416	-1.336				-1.777	-1.498			EN.SM.Down.12H.24H
**PLSCR4**		-2.221	-1.289				-2.351	-1.976			EN.SM.Down.12H.24H
**RBL2**		-1.793	-1.264				-1.604	-1.85			EN.SM.Down.12H.24H
**RCAN2**		-1.791	-1.863				-2.707	-2.202			EN.SM.Down.12H.24H
**SLC44A2**		-1.941	-1.647				-1.548	-1.412			EN.SM.Down.12H.24H
**SMARCA2**		-1.823	-1.926				-2.051	-1.746			EN.SM.Down.12H.24H
**SPINK2**		-3.209	-2.073				-2.661	-3.019			EN.SM.Down.12H.24H
**TACC1**		-1.581	-1.313				-1.627	-1.368			EN.SM.Down.12H.24H
**TCP11L2**		-1.938	-1.877				-1.753	-2.192			EN.SM.Down.12H.24H
**THBS3**		-1.516	-1.859				-1.645	-1.746			EN.SM.Down.12H.24H
**TSC22D1**		-2.419	-1.641				-3.312	-2.064			EN.SM.Down.12H.24H
**AXIN2**		-1.727		-1.746			-2.128		-1.373		EN.SM.Down.12H.7D
**CABP1**		-1.617		-2.851			-1.546		-2.041		EN.SM.Down.12H.7D
**CHKA**		-1.441		-1.175			-1.596		-1.302		EN.SM.Down.12H.7D
**DLG3**		-1.325		-1.896			-2.954		-1.429		EN.SM.Down.12H.7D
**HOXA5**		-2.952		-1.983			-1.801		-1.333		EN.SM.Down.12H.7D
**LIMS2**		-2.126		-1.104			-2.172		-2.167		EN.SM.Down.12H.7D
**LOC486761**		-1.033		-1.396			-1.194		-1.412		EN.SM.Down.12H.7D
**LOC487009**		-1.385		-1.237			-1.328		-1.231		EN.SM.Down.12H.7D
**MPP7**		-2.503		-1.869			-2.556		-1.137		EN.SM.Down.12H.7D
**MTSS1L**		-1.707		-1.785			-1.775		-1.467		EN.SM.Down.12H.7D
**PPP1R12A**		-1.355		-1.336			-1.66		-1.802		EN.SM.Down.12H.7D
**RALGPS2**		-1.827		-3.057			-1.625		-1.265		EN.SM.Down.12H.7D
**SLC38A4**		-1.464		-1.637			-2.125		-1.588		EN.SM.Down.12H.7D
**SMARCD3**		-1.585		-1.271			-2.047		-1.237		EN.SM.Down.12H.7D
**TLE1**		-1.205		-1.246			-1.596		-1.052		EN.SM.Down.12H.7D
**CHD6**			-1.02				-1.687	-2.018	-1.347		EN.SM.Down.24H, SM.Only.Down.12H.7D
**GATM**			-2.557				-2.228	-2.344	-1.059		EN.SM.Down.24H, SM.Only.Down.12H.7D
**ARHGAP10**				-1.725			-2.66	-2.176	-1.531		EN.SM.Down.7D, SM.Only.Down.12H.24H
**CST9L**				-1.925			-3.215	-2.65	-3.359		EN.SM.Down.7D, SM.Only.Down.12H.24H
**DGKG**				-2.484			-1.651	-1.485	-1.928		EN.SM.Down.7D, SM.Only.Down.12H.24H
**STOX2**				-1.056			-1.242	-1.985	-1.303		EN.SM.Down.7D, SM.Only.Down.12H.24H
**COL3A1**		-3.181	-3.224	1.09	2.778		-3.304		1.26		EN.Only.Down.24H, EN.SM.Up.7D, EN.SM.Down.12H, EN.Only.Up.30D
**ATP1B3**		-1.917	-1.839	1.738			1.633	-2.81	1.13		EN.SM.Up.7D, EN.SM.Down.12H.24H, SM.Only.Down.7D, SM.Only.Up.12H
**SVEP1**		-1.528			2.644		-2.493	-2.056		1.975	EN.SM.Up.30D, EN.SM.Down.12H, SM.Only.Down.24H
**NTS**		-2.213	-2.983	-2.339			3.443	3.438			EN.Only.Down.12H.24H.7D, SM.Only.Up.12H.24H
**CTSK**		-2.094	-1.936	1.605			-2.515		1.464		EN.Only.Down.24H, EN.SM.Up.7D, EN.SM.Down.12H
**LOC610234**		-1.079			3.313		-2.459	-1.835			EN.SM.Down.12H, SM.Only.Down.24H, EN.Only.Up.30D
**MXRA8**		-3.304	-3.124	1.444			-3.133				EN.Only.Down.24H, EN.SM.Down.12H, EN.Only.Up.7D
**FAP**		-2.007	-2.265				-1.333		1.323		EN.Only.Down.24H, EN.SM.Down.12H, SM.Only.Up.7D
**LOC488984**		-2.012	-1.981				-1.855		1.651		EN.Only.Down.24H, EN.SM.Down.12H, SM.Only.Up.7D
**LOC609688**		-2.142	-2.164				-2.612		1.044		EN.Only.Down.24H, EN.SM.Down.12H, SM.Only.Up.7D
**PRSS35**		-2.876	-2.777				-3.058		1.735		EN.Only.Down.24H, EN.SM.Down.12H, SM.Only.Up.7D
**SESN3**		-2.433	-2.329				-1.898		1.355		EN.Only.Down.24H, EN.SM.Down.12H, SM.Only.Up.7D
**SLC17A5**		-1.952	-1.219				-2.231		1.735		EN.Only.Down.24H, EN.SM.Down.12H, SM.Only.Up.7D
**TNFSF10**		-3.05	-2.03				-1.495		1.221		EN.Only.Down.24H, EN.SM.Down.12H, SM.Only.Up.7D
**DAAM1**	1.36		-1.846	-1.523					-1.246		EN.Only.Down.24H, EN.SM.Down.7D, EN.Only.Up.2H
**ZAR1L**		-1.518					-1.467	-3.61	1.298		EN.SM.Down.12H, SM.Only.Down.24H, SM.Only.Up.7D
**SLC27A3**			-1.635	1.061			-1.058	-1.908			EN.SM.Down.24H, SM.Only.Down.12H, EN.Only.Up.7D
**BCL2**	1.491			-1.47			-1.072		-1.667		EN.SM.Down.7D, SM.Only.Down.12H, EN.Only.Up.2H
**HAND2**		-4.805			-2.227		-6.734				EN.Only.Down.30D, EN.SM.Down.12H
**FAH**		-1.688					-2.221	-1.766			EN.Only.Down.12H
**FXYD6**		-2.033	-1.335					-1.721			EN.Only.Down.12H, EN.SM.Down.24H
**ITGA11**		-2.877	-3.285					-2.875			EN.Only.Down.12H, EN.SM.Down.24H
**EMX2**		-1.357		-1.755					-1.344		EN.Only.Down.12H, EN.SM.Down.7D
**LOC490316**		-1.069		-1.049					-1.212		EN.Only.Down.12H, EN.SM.Down.7D
**PTBP2**		-1.286		-1.94					-1.694		EN.Only.Down.12H, EN.SM.Down.7D
**THRB**		-1.31		-1.464					-1.049		EN.Only.Down.12H, EN.SM.Down.7D
**GABARAPL1**		-1.182	-1.423						-1.194		EN.Only.Down.12H.24H, SM.Only.Down.7D
**LRIG1**		-2.011	-2.374						-1.128		EN.Only.Down.12H.24H, SM.Only.Down.7D
**RABGAP1L**		-1.066	-1.051						-1.033		EN.Only.Down.12H.24H, SM.Only.Down.7D
**EPCAM**		-2.974	-2.891	-3.243							EN.Only.Down.12H.24H.7D
**LOC487291**		-2.135	-2.152	-1.092							EN.Only.Down.12H.24H.7D
**MYRIP**		-2.157	-2.461	-2.451							EN.Only.Down.12H.24H.7D
**NIPAL1**		-2.159	-2.539	-1.335							EN.Only.Down.12H.24H.7D
**PKP4**		-1.068	-1.588	-1.173							EN.Only.Down.12H.24H.7D
**RDH16**		-6.273	-4.6	-2.374							EN.Only.Down.12H.24H.7D
**FBXL2**			-1.207				-1.29	-1.937			EN.Only.Down.24H
**6-Sep**		-2.341	-2.462				-1.986				EN.Only.Down.24H, EN.SM.Down.12H
**AMT**		-1.301	-1.334				-1.368				EN.Only.Down.24H, EN.SM.Down.12H
**ANKRD50**		-1.266	-1.871				-1.19				EN.Only.Down.24H, EN.SM.Down.12H
**ANXA4**		-1.926	-1.639				-1.182				EN.Only.Down.24H, EN.SM.Down.12H
**APLP2**		-1.202	-1.537				-1.145				EN.Only.Down.24H, EN.SM.Down.12H
**ATG13**		-1.861	-1.426				-1.238				EN.Only.Down.24H, EN.SM.Down.12H
**BACE1**		-1.56	-1.167				-1.194				EN.Only.Down.24H, EN.SM.Down.12H
**BBS2**		-1.98	-1.378				-1.509				EN.Only.Down.24H, EN.SM.Down.12H
**BCAM**		-2.033	-1.798				-1.75				EN.Only.Down.24H, EN.SM.Down.12H
**BMP4**		-3.468	-2.007				-1.176				EN.Only.Down.24H, EN.SM.Down.12H
**CALCOCO2**		-1.921	-1.151				-1.583				EN.Only.Down.24H, EN.SM.Down.12H
**CCDC28A**		-1.819	-1.535				-1.669				EN.Only.Down.24H, EN.SM.Down.12H
**CCNDBP1**		-1.542	-1.619				-1.38				EN.Only.Down.24H, EN.SM.Down.12H
**CLIP3**		-1.479	-1.286				-1.452				EN.Only.Down.24H, EN.SM.Down.12H
**CLSTN1**		-1.849	-1.532				-2.024				EN.Only.Down.24H, EN.SM.Down.12H

**CPXM2**		-1.732	-2.525				-1.541				EN.Only.Down.24H, EN.SM.Down.12H
**DECR1**		-2.017	-1.245				-1.836				EN.Only.Down.24H, EN.SM.Down.12H
**DHRS4**		-1.979	-1.39				-1.886				EN.Only.Down.24H, EN.SM.Down.12H
**DIXDC1**		-1.15	-1.459				-1.499				EN.Only.Down.24H, EN.SM.Down.12H
**DMGDH**		-3.347	-1.552				-1.997				EN.Only.Down.24H, EN.SM.Down.12H
**DNALI1**		-2.116	-1.322				-2.287				EN.Only.Down.24H, EN.SM.Down.12H
**EBPL**		-1.672	-1.169				-1.076				EN.Only.Down.24H, EN.SM.Down.12H
**EFEMP2**		-1.474	-1.315				-1.491				EN.Only.Down.24H, EN.SM.Down.12H
**EPHX1**		-1.822	-1.558				-1.479				EN.Only.Down.24H, EN.SM.Down.12H
**EPHX2**		-3.11	-2.428				-2.891				EN.Only.Down.24H, EN.SM.Down.12H
**FBLIM1**		-1.619	-1.123				-2.231				EN.Only.Down.24H, EN.SM.Down.12H
**FBXO32**		-3.672	-1.96				-3.298				EN.Only.Down.24H, EN.SM.Down.12H
**FHL5**		-3.433	-4.233				-1.132				EN.Only.Down.24H, EN.SM.Down.12H
**FYCO1**		-1.843	-1.661				-1.916				EN.Only.Down.24H, EN.SM.Down.12H
**GAS6**		-1.912	-2.204				-2.317				EN.Only.Down.24H, EN.SM.Down.12H
**GJA8**		-2.102	-2.844				-2.238				EN.Only.Down.24H, EN.SM.Down.12H
**GLT8D2**		-2.341	-2.031				-2.507				EN.Only.Down.24H, EN.SM.Down.12H
**GNG11**		-2.332	-1.786				-1.678				EN.Only.Down.24H, EN.SM.Down.12H
**GSTM4**		-1.285	-1.522				-1.325				EN.Only.Down.24H, EN.SM.Down.12H
**GTF3C2**		-1.349	-1.182				-1.088				EN.Only.Down.24H, EN.SM.Down.12H
**HIBADH**		-1.303	-1.207				-1.562				EN.Only.Down.24H, EN.SM.Down.12H
**HMGN3**		-2.03	-1.282				-3.042				EN.Only.Down.24H, EN.SM.Down.12H
**ICK**		-1.901	-1.815				-1.69				EN.Only.Down.24H, EN.SM.Down.12H
**IFT172**		-2.046	-1.408				-2.428				EN.Only.Down.24H, EN.SM.Down.12H
**JAG1**		-1.514	-1.522				-1.741				EN.Only.Down.24H, EN.SM.Down.12H
**KCNMB1**		-1.289	-1.741				-1.368				EN.Only.Down.24H, EN.SM.Down.12H
**KDELC2**		-1.486	-1.026				-1.428				EN.Only.Down.24H, EN.SM.Down.12H
**LDOC1**		-2.078	-1.406				-2.154				EN.Only.Down.24H, EN.SM.Down.12H
**LHFP**		-1.732	-1.376				-1.871				EN.Only.Down.24H, EN.SM.Down.12H
**LOC474709**		-1.644	-1.188				-1.164				EN.Only.Down.24H, EN.SM.Down.12H
**LOC480441**		-1.835	-1.211				-2.259				EN.Only.Down.24H, EN.SM.Down.12H
**LOC480657**		-1.876	-1.192				-1.221				EN.Only.Down.24H, EN.SM.Down.12H
**LOC483247**		-1.22	-1.689				-1.769				EN.Only.Down.24H, EN.SM.Down.12H
**LOC489163**		-2.184	-1.533				-1.351				EN.Only.Down.24H, EN.SM.Down.12H
**LOC490959**		-2.055	-1.423				-1.07				EN.Only.Down.24H, EN.SM.Down.12H
**LOC610479**		-1.784	-1.133				-1.957				EN.Only.Down.24H, EN.SM.Down.12H
**LOC611366**		-2.182	-1.59				-1.502				EN.Only.Down.24H, EN.SM.Down.12H
**LOC611894**		-1.836	-1.457				-1.747				EN.Only.Down.24H, EN.SM.Down.12H
**LOC612614**		-2.545	-2.033				-1.88				EN.Only.Down.24H, EN.SM.Down.12H
**LRBA**		-1.726	-1.342				-2.101				EN.Only.Down.24H, EN.SM.Down.12H
**LZTFL1**		-1.835	-1.551				-1.817				EN.Only.Down.24H, EN.SM.Down.12H
**MAP1LC3B**		-1.475	-1.452				-1.157				EN.Only.Down.24H, EN.SM.Down.12H
**MLLT3**		-1.663	-1.277				-1.721				EN.Only.Down.24H, EN.SM.Down.12H
**MTMR15**		-1.87	-2.285				-1.519				EN.Only.Down.24H, EN.SM.Down.12H
**NAT14**		-1.5	-1.197				-1.639				EN.Only.Down.24H, EN.SM.Down.12H
**NEK7**		-1.191	-1.163				-1.082				EN.Only.Down.24H, EN.SM.Down.12H
**NFIX**		-1.749	-1.849				-2.187				EN.Only.Down.24H, EN.SM.Down.12H
**NICN1**		-1.433	-1.281				-1.656				EN.Only.Down.24H, EN.SM.Down.12H
**NOTCH3**		-1.16	-1.333				-1.1				EN.Only.Down.24H, EN.SM.Down.12H
**NR2F1**		-2.536	-1.938				-2.214				EN.Only.Down.24H, EN.SM.Down.12H
**NR2F2**		-1.502	-1.29				-1.992				EN.Only.Down.24H, EN.SM.Down.12H
**NRP1**		-1.587	-1.567				-2.365				EN.Only.Down.24H, EN.SM.Down.12H
**NUCB2**		-1.698	-1.002				-1.523				EN.Only.Down.24H, EN.SM.Down.12H
**OGN**		-2.75	-2.852				-1.131				EN.Only.Down.24H, EN.SM.Down.12H
**PAK3**		-1.176	-2.192				-1.925				EN.Only.Down.24H, EN.SM.Down.12H
**PAM**		-1.703	-1.899				-1.343				EN.Only.Down.24H, EN.SM.Down.12H
**PDGFC**		-1.755	-3.654				-2.907				EN.Only.Down.24H, EN.SM.Down.12H
**PDGFRL**		-2.362	-1.573				-1.863				EN.Only.Down.24H, EN.SM.Down.12H
**PHACTR3**		-1.283	-1.176				-1.07				EN.Only.Down.24H, EN.SM.Down.12H
**PPAP2A**		-1.531	-1.862				-1.471				EN.Only.Down.24H, EN.SM.Down.12H
**PRUNE2**		-3.134	-1.811				-3.094				EN.Only.Down.24H, EN.SM.Down.12H
**RARB**		-2.397	-1.812				-1.063				EN.Only.Down.24H, EN.SM.Down.12H
**RARRES2**		-1.663	-2.065				-1.255				EN.Only.Down.24H, EN.SM.Down.12H
**RECK**		-1.613	-1.838				-1.784				EN.Only.Down.24H, EN.SM.Down.12H
**REEP3**		-1.167	-1.115				-1.329				EN.Only.Down.24H, EN.SM.Down.12H
**RNASE4**		-2.949	-2.402				-2.316				EN.Only.Down.24H, EN.SM.Down.12H
**RRAGB**		-2.08	-1.815				-1.254				EN.Only.Down.24H, EN.SM.Down.12H
**RUNX1T1**		-2.363	-1.785				-2.676				EN.Only.Down.24H, EN.SM.Down.12H
**RXFP1**		-2.333	-1.882				-1.077				EN.Only.Down.24H, EN.SM.Down.12H
**SELENBP1**		-1.715	-1.692				-2.313				EN.Only.Down.24H, EN.SM.Down.12H
**SH3BGRL**		-1.979	-2.171				-2.07				EN.Only.Down.24H, EN.SM.Down.12H
**SLC25A27**		-2.929	-1.761				-3.259				EN.Only.Down.24H, EN.SM.Down.12H
**SNCG**		-2.279	-1.923				-1.489				EN.Only.Down.24H, EN.SM.Down.12H
**SNED1**		-2.71	-1.709				-1.387				EN.Only.Down.24H, EN.SM.Down.12H
**SORBS3**		-1.158	-1.066				-1.291				EN.Only.Down.24H, EN.SM.Down.12H
**SPG21**		-2.371	-1.38				-2.278				EN.Only.Down.24H, EN.SM.Down.12H
**SPTAN1**		-1.18	-1.2				-1.19				EN.Only.Down.24H, EN.SM.Down.12H
**SSBP2**		-2.498	-1.298				-2.006				EN.Only.Down.24H, EN.SM.Down.12H
**SSH3**		-1.427	-1.15				-1.755				EN.Only.Down.24H, EN.SM.Down.12H
**STOM**		-1.725	-1.887				-1.451				EN.Only.Down.24H, EN.SM.Down.12H
**THR**		-1.226	-1.232				-1.075				EN.Only.Down.24H, EN.SM.Down.12H
**TIMP2**		-1.234	-1.139				-1.007				EN.Only.Down.24H, EN.SM.Down.12H
**TMEM47**		-1.774	-1.469				-1.806				EN.Only.Down.24H, EN.SM.Down.12H
**TMEM59**		-1.383	-1.142				-1.207				EN.Only.Down.24H, EN.SM.Down.12H
**TMTC1**		-1.865	-1.245				-1.308				EN.Only.Down.24H, EN.SM.Down.12H
**TMTC4**		-2.852	-2.085				-1.122				EN.Only.Down.24H, EN.SM.Down.12H
**TNIK**		-1.885	-1.291				-2.15				EN.Only.Down.24H, EN.SM.Down.12H
**TRIM63**		-2.863	-1.446				-3.351				EN.Only.Down.24H, EN.SM.Down.12H
**UBE2H**		-1.012	-1.609				-1.054				EN.Only.Down.24H, EN.SM.Down.12H
**UBE2Q2**		-1.722	-1.571				-1.695				EN.Only.Down.24H, EN.SM.Down.12H
**USP53**		-1.389	-1.71				-1.419				EN.Only.Down.24H, EN.SM.Down.12H
**VLDLR**		-3.488	-2.772				-3.222				EN.Only.Down.24H, EN.SM.Down.12H
**WDR90**		-3.112	-1.942				-2.791				EN.Only.Down.24H, EN.SM.Down.12H
**XPA**		-1.438	-1.355				-1.269				EN.Only.Down.24H, EN.SM.Down.12H
**YPEL1**		-2.808	-2.868				-1.513				EN.Only.Down.24H, EN.SM.Down.12H
**ZMAT4**		-2.016	-2.087				-1.553				EN.Only.Down.24H, EN.SM.Down.12H
**ZNF532**		-1.587	-1.472				-1.057				EN.Only.Down.24H, EN.SM.Down.12H
**ZNF704**		-1.988	-1.283				-1.91				EN.Only.Down.24H, EN.SM.Down.12H
**ARHGEF15**			-1.329	-1.737					-1.413		EN.Only.Down.24H, EN.SM.Down.7D
**EPAS1**			-1.032	-1.123					-1.412		EN.Only.Down.24H, EN.SM.Down.7D
**LOC606881**			-1.308	-1.638					-1.961		EN.Only.Down.24H, EN.SM.Down.7D
**LOC611604**			-2.045	-1.178					-1.196		EN.Only.Down.24H, EN.SM.Down.7D
**PMVK**			-1.453	-1.148					-1.39		EN.Only.Down.24H, EN.SM.Down.7D
**DDAH2**			-1.02				-1.649		-1.28		EN.Only.Down.24H, SM.Only.Down.12H.7D
**PDLIM7**			-1.239				-1.356		-1.56		EN.Only.Down.24H, SM.Only.Down.12H.7D
**ADCY5**			-1.359	-1.188			-2.687				EN.Only.Down.24H.7D, SM.Only.Down.12H
**BAG2**			-1.298	-1.147			-1.058				EN.Only.Down.24H.7D, SM.Only.Down.12H
**CEP63**			-1.839	-1.53			-1.702				EN.Only.Down.24H.7D, SM.Only.Down.12H
**ELP3**			-2.61	-1.449			-1.651				EN.Only.Down.24H.7D, SM.Only.Down.12H
**HEPHL1**			-3.299	-1.591			-1.228				EN.Only.Down.24H.7D, SM.Only.Down.12H
**LOC480041**			-1.216	-1.245			-1.071				EN.Only.Down.24H.7D, SM.Only.Down.12H
**LOC486484**			-1.95	-1.187			-1.427				EN.Only.Down.24H.7D, SM.Only.Down.12H
**LOC609616**			-2.019	-1.817			-1.261				EN.Only.Down.24H.7D, SM.Only.Down.12H
**LOC611878**			-1.901	-2.384			-1.455				EN.Only.Down.24H.7D, SM.Only.Down.12H
**SMOC2**			-1.384	-2.551			-1.407				EN.Only.Down.24H.7D, SM.Only.Down.12H
**ZRANB1**			-1.422	-1.167			-1.451				EN.Only.Down.24H.7D, SM.Only.Down.12H
**BRCA2**	-1.792	-1.819					-2.003				EN.Only.Down.2H, EN.SM.Down.12H
**LOC610949**	-1.409	-1.884					-1.532				EN.Only.Down.2H, EN.SM.Down.12H
**RSPH3**	-1.717	-1.27					-1.796				EN.Only.Down.2H, EN.SM.Down.12H
**SLC25A12**	-1.821	-1.636					-1.898				EN.Only.Down.2H, EN.SM.Down.12H
**LOC482778**	-1.802						-1.519	-1.397			EN.Only.Down.2H, SM.Only.Down.12H.24H
**BMPR1A**		-1.712		-1.279			-1.595				EN.Only.Down.7D, EN.SM.Down.12H
**BVES**		-1.804		-1.766			-2.656				EN.Only.Down.7D, EN.SM.Down.12H

CAB39L		-1.595		-1.518			-2.187				EN.Only.Down.7D, EN.SM.Down.12H
CCDC30		-2.916		-1.229			-1.158				EN.Only.Down.7D, EN.SM.Down.12H
DUSP23		-1.971		-1.106			-1.722				EN.Only.Down.7D, EN.SM.Down.12H
KBTBD3		-1.724		-1.006			-1.71				EN.Only.Down.7D, EN.SM.Down.12H
KLHDC1		-1.72		-1.708			-2.27				EN.Only.Down.7D, EN.SM.Down.12H
KLHDC6		-2.488		-1.132			-2.359				EN.Only.Down.7D, EN.SM.Down.12H
KLHL13		-1.745		-1.545			-1.515				EN.Only.Down.7D, EN.SM.Down.12H
LOC480082		-1.114		-1.155			-1.044				EN.Only.Down.7D, EN.SM.Down.12H
LOC484162		-1.368		-1.096			-1.615				EN.Only.Down.7D, EN.SM.Down.12H
LOC484542		-1.677		-1.362			-1.907				EN.Only.Down.7D, EN.SM.Down.12H
MDGA1		-2.297		-2.628			-1.812				EN.Only.Down.7D, EN.SM.Down.12H
NEO1		-1.667		-1.34			-1.197				EN.Only.Down.7D, EN.SM.Down.12H
PEG3		-1.654		-1.127			-1.937				EN.Only.Down.7D, EN.SM.Down.12H
PLP1		-1.829		-2.192			-1.833				EN.Only.Down.7D, EN.SM.Down.12H
PPP4R4		-1.645		-1.404			-1.285				EN.Only.Down.7D, EN.SM.Down.12H
RASGRF2		-2.234		-2.573			-1.069				EN.Only.Down.7D, EN.SM.Down.12H
RORA		-1.529		-1.21			-1.705				EN.Only.Down.7D, EN.SM.Down.12H
SLC26A7		-1.342		-1.604			-1.255				EN.Only.Down.7D, EN.SM.Down.12H
SLC7A2		-2.246		-2.006			-3.1				EN.Only.Down.7D, EN.SM.Down.12H
SRMS		-1.235		-1.081			-1.026				EN.Only.Down.7D, EN.SM.Down.12H
TMEM55A		-2.676		-1.646			-2.961				EN.Only.Down.7D, EN.SM.Down.12H
TSPAN12		-1.509		-1.242			-1.623				EN.Only.Down.7D, EN.SM.Down.12H
TUBB2B		-2.03		-1.776			-1.325				EN.Only.Down.7D, EN.SM.Down.12H
ESR1				-1.129			-1.517	-1.83			EN.Only.Down.7D, SM.Only.Down.12H.24H
GPRC5C				-1.833			-1.397	-1.695			EN.Only.Down.7D, SM.Only.Down.12H.24H
KLHL23				-1.517			-2.028	-1.81			EN.Only.Down.7D, SM.Only.Down.12H.24H
PLG				-1.047			-1.423	-2.372			EN.Only.Down.7D, SM.Only.Down.12H.24H
VSIG2				-1.967			-1.976	-2.6			EN.Only.Down.7D, SM.Only.Down.12H.24H
AKAP5		-2.141					-1.687	-1.617			EN.SM.Down.12H, SM.Only.Down.24H
ASPA		-1.689					-1.793	-3.262			EN.SM.Down.12H, SM.Only.Down.24H
BDH2		-3.15					-3.799	-1.96			EN.SM.Down.12H, SM.Only.Down.24H
CCNG2		-2.186					-2.745	-2.22			EN.SM.Down.12H, SM.Only.Down.24H
FABP2		-1.627					-1.779	-2.327			EN.SM.Down.12H, SM.Only.Down.24H
FBXO21		-1.141					-1.378	-1.693			EN.SM.Down.12H, SM.Only.Down.24H
FGGY		-1.607					-1.256	-2.288			EN.SM.Down.12H, SM.Only.Down.24H
FHIT		-2.582					-2.081	-3.05			EN.SM.Down.12H, SM.Only.Down.24H
GAB1		-1.945					-2.384	-1.333			EN.SM.Down.12H, SM.Only.Down.24H
GPLD1		-1.914					-1.336	-1.916			EN.SM.Down.12H, SM.Only.Down.24H
IFFO1		-1.381					-1.312	-2.236			EN.SM.Down.12H, SM.Only.Down.24H
LOC484150		-1.169					-1.542	-1.666			EN.SM.Down.12H, SM.Only.Down.24H
LOC608881		-3.724					-3.33	-3.024			EN.SM.Down.12H, SM.Only.Down.24H
LOC610426		-2.325					-1.266	-1.273			EN.SM.Down.12H, SM.Only.Down.24H
LOC610871		-1.994					-2.347	-1.896			EN.SM.Down.12H, SM.Only.Down.24H
LOC611882		-1.439					-1.685	-1.528			EN.SM.Down.12H, SM.Only.Down.24H
LRRC16A		-2.135					-1.759	-1.819			EN.SM.Down.12H, SM.Only.Down.24H
MDP1		-1.543					-1.142	-1.721			EN.SM.Down.12H, SM.Only.Down.24H
NCEH1		-1.527					-1.809	-1.559			EN.SM.Down.12H, SM.Only.Down.24H
NEIL2		-3.292					-2.842	-1.928			EN.SM.Down.12H, SM.Only.Down.24H
NLGN1		-2.135					-3.443	-2.024			EN.SM.Down.12H, SM.Only.Down.24H
NSMCE1		-1.577					-1.342	-2.176			EN.SM.Down.12H, SM.Only.Down.24H
PLTP		-1.517					-1.267	-1.724			EN.SM.Down.12H, SM.Only.Down.24H
SGCD		-1.48					-2.458	-1.527			EN.SM.Down.12H, SM.Only.Down.24H
SLITRK6		-3.191					-4.32	-3.04			EN.SM.Down.12H, SM.Only.Down.24H
SULT1A1		-2.351					-2.656	-2.485			EN.SM.Down.12H, SM.Only.Down.24H
TCEA3		-1.944					-2.051	-1.628			EN.SM.Down.12H, SM.Only.Down.24H
TET1		-2.366					-2.369	-1.837			EN.SM.Down.12H, SM.Only.Down.24H
WDR19		-1.879					-1.466	-1.472			EN.SM.Down.12H, SM.Only.Down.24H
WDR60		-1.501					-1.697	-1.519			EN.SM.Down.12H, SM.Only.Down.24H
ZEB2		-1.529					-1.428	-1.333			EN.SM.Down.12H, SM.Only.Down.24H
ZNF618		-1.275					-1.781	-1.467			EN.SM.Down.12H, SM.Only.Down.24H
ZNF699		-1.193					-1.198	-1.666			EN.SM.Down.12H, SM.Only.Down.24H
ARHGAP6		-1.404					-1.978		-1.333		EN.SM.Down.12H, SM.Only.Down.7D
ARHGEF17		-1.138					-1.507		-1.045		EN.SM.Down.12H, SM.Only.Down.7D
CA11		-1.411					-1.233		-1.091		EN.SM.Down.12H, SM.Only.Down.7D
CDH19		-3.396					-1.807		-2.462		EN.SM.Down.12H, SM.Only.Down.7D
CDKL5		-1.416					-2.227		-1.174		EN.SM.Down.12H, SM.Only.Down.7D
CHD3		-1.067					-1.252		-1.119		EN.SM.Down.12H, SM.Only.Down.7D
CHRNA1		-3.233					-3.255		-1.504		EN.SM.Down.12H, SM.Only.Down.7D
CUGBP2		-1.082					-1.913		-1.572		EN.SM.Down.12H, SM.Only.Down.7D
DOCK6		-1.507					-1.21		-1.272		EN.SM.Down.12H, SM.Only.Down.7D
F2RL2		-3.357					-2.245		-1.581		EN.SM.Down.12H, SM.Only.Down.7D
FMO4		-1.257					-1.077		-2.786		EN.SM.Down.12H, SM.Only.Down.7D
GNAI1		-1.24					-1.644		-1.357		EN.SM.Down.12H, SM.Only.Down.7D
KLKB1		-1.965					-1.592		-1.194		EN.SM.Down.12H, SM.Only.Down.7D
LOC486544		-1.686					-2.075		-2.037		EN.SM.Down.12H, SM.Only.Down.7D
LOC609596		-1.934					-1.346		-3.986		EN.SM.Down.12H, SM.Only.Down.7D
LOC611494		-2.628					-2.145		-1.407		EN.SM.Down.12H, SM.Only.Down.7D
LOC611848		-2.026					-2.152		-1.77		EN.SM.Down.12H, SM.Only.Down.7D
NDRG3		-1.243					-1.567		-1.211		EN.SM.Down.12H, SM.Only.Down.7D
NEK11		-1.773					-1.768		-1.261		EN.SM.Down.12H, SM.Only.Down.7D
PDK2		-1.466					-1.074		-1.173		EN.SM.Down.12H, SM.Only.Down.7D
PIGP		-1.461					-1.243		-1.388		EN.SM.Down.12H, SM.Only.Down.7D
RAB11B		-1.223					-1.124		-1.269		EN.SM.Down.12H, SM.Only.Down.7D
RERG		-2.163					-2.766		-1.046		EN.SM.Down.12H, SM.Only.Down.7D
SHOX2		-1.641					-1.196		-1.643		EN.SM.Down.12H, SM.Only.Down.7D
SLC22A17		-1.6					-1.047		-1.011		EN.SM.Down.12H, SM.Only.Down.7D
SLC25A36		-1.519					-1.117		-1.039		EN.SM.Down.12H, SM.Only.Down.7D
SSBP3		-1.146					-1.582		-1.107		EN.SM.Down.12H, SM.Only.Down.7D
TBX15		-1.619					-1.715		-1.087		EN.SM.Down.12H, SM.Only.Down.7D
TSPAN2		-2.463					-1.026		-1.401		EN.SM.Down.12H, SM.Only.Down.7D
TTC33		-1.085					-1.55		-1.391		EN.SM.Down.12H, SM.Only.Down.7D
ZCCHC24		-1.746					-1.399		-1.271		EN.SM.Down.12H, SM.Only.Down.7D
ZNF185		-1.906					-2.029		-1.381		EN.SM.Down.12H, SM.Only.Down.7D
ANTXR1			-2.197				-1.413	-1.553			EN.SM.Down.24H, SM.Only.Down.12H
ATP1B2			-2.607				-1.594	-2.775			EN.SM.Down.24H, SM.Only.Down.12H
C1QTNF1			-1.81				-1.364	-2.022			EN.SM.Down.24H, SM.Only.Down.12H
GATA6			-1.764				-1.071	-1.914			EN.SM.Down.24H, SM.Only.Down.12H
LGALS8			-1.059				-1.341	-2.082			EN.SM.Down.24H, SM.Only.Down.12H
LOC475083			-2.555				-2.052	-2.375			EN.SM.Down.24H, SM.Only.Down.12H
PCDH20			-1.839				-3.529	-2.744			EN.SM.Down.24H, SM.Only.Down.12H
PRR15			-3.279				-1.551	-2.254			EN.SM.Down.24H, SM.Only.Down.12H
ZER1			-1.295				-1.329	-1.467			EN.SM.Down.24H, SM.Only.Down.12H
C7			-1.326					-2.829	-3.04		EN.SM.Down.24H, SM.Only.Down.7D
ENTPD1			-1.146					-1.922	-1.184		EN.SM.Down.24H, SM.Only.Down.7D
LYRM1			-1.251					-1.852	-1.017		EN.SM.Down.24H, SM.Only.Down.7D
IER5L	-2.351					-2.78	-1.419				EN.SM.Down.2H, SM.Only.Down.12H
CACNB2				-1.795			-1.633		-1.871		EN.SM.Down.7D, SM.Only.Down.12H
CCDC158				-1.542			-1.443		-1.334		EN.SM.Down.7D, SM.Only.Down.12H
DPF2				-1.295			-1		-1.247		EN.SM.Down.7D, SM.Only.Down.12H
EML5				-1.178			-1.153		-1.103		EN.SM.Down.7D, SM.Only.Down.12H
EXTL3				-1.119			-1.783		-1.319		EN.SM.Down.7D, SM.Only.Down.12H
LOC474650				-1.216			-2.143		-1.627		EN.SM.Down.7D, SM.Only.Down.12H
LOC481669				-1.035			-1.283		-1.237		EN.SM.Down.7D, SM.Only.Down.12H
RIMKLB				-1.785			-1.564		-1.168		EN.SM.Down.7D, SM.Only.Down.12H
SLC25A40				-1.129			-1.231		-1.219		EN.SM.Down.7D, SM.Only.Down.12H
SOSTDC1				-1.163			-1.617		-1.198		EN.SM.Down.7D, SM.Only.Down.12H
SYVN1				-1.261			-1.332		-1.566		EN.SM.Down.7D, SM.Only.Down.12H
ABCA9							-2.653	-1.286	-2.064		SM.Only.Down.12H.24H.7D
ALDH1A1							-4.549	-4.103	-5.003		SM.Only.Down.12H.24H.7D
ARHGAP29							-1.337	-2.09	-1.393		SM.Only.Down.12H.24H.7D
LOC611967							-1.671	-3.234	-1.629		SM.Only.Down.12H.24H.7D
LOC612888							-1.331	-1.625	-1.094		SM.Only.Down.12H.24H.7D
NRXN1							-2.485	-4.03	-1.94		SM.Only.Down.12H.24H.7D

**NTNG1**							-4.338	-4.704	-2.376		SM.Only.Down.12H.24H.7D
**SLC2A12**							-1.533	-1.395	-1.483		SM.Only.Down.12H.24H.7D
**TNFAIP8**		-1.294					-1.142		-1.247		SM.Only.Down.12H.7D
**STMN2**							-2.232		-2.106	-2.865	SM.Only.Down.12H.7D.30D
**MTSS1**		-1.707		-1.785				-1.811			SM.Only.Down.24H
**SNCA**		-1.172	-1.472						-1.363		SM.Only.Down.7D
**COL1A1**		-1.428		2.34	2.522		-1.089		1.697	2.357	EN.SM.Up.7D.30D, EN.SM.Down.12H
**COL1A2**		-2.664		1.451	2.974		-1.742		1.213	2.218	EN.SM.Up.7D.30D, EN.SM.Down.12H
**LOC486411**		-1.588		1.774	2.323		-1.036		1.434		EN.SM.Up.7D, EN.SM.Down.12H, EN.Only.Up.30D
**LOXL2**		-1.577		1.958	2.93		-1.73		1.35		EN.SM.Up.7D, EN.SM.Down.12H, EN.Only.Up.30D
**CCDC88A**		-1.255	1.914	1.881			-2.087		2.115		EN.SM.Up.7D, EN.SM.Down.12H, EN.Only.Up.24H
**HTRA1**		-1.794	-1.343	1.461					2.162		EN.Only.Down.12H.24H, EN.SM.Up.7D
**LOC474886**	-2.008			1.222			-1.942		2.016		EN.Only.Down.2H, EN.SM.Up.7D, SM.Only.Down.12H
**NUSAP1**	-1.707	-1.557		2.306					1.926		EN.Only.Down.2H.12H, EN.SM.Up.7D
**CREB5**		1.647		-1.665			1.099		-2.354		EN.SM.Up.12H, EN.SM.Down.7D
**MEIS2**	2.667	-1.002				2.999	-1.391				EN.SM.Up.2H, EN.SM.Down.12H
**CDC25B**		-1.604		1.164			-1.575		2.334		EN.SM.Up.7D, EN.SM.Down.12H
**COL8A1**		-1.53		2.559			-1.91		2.842		EN.SM.Up.7D, EN.SM.Down.12H
**OLFML2B**		-1.485		1.311			-1.629		1.607		EN.SM.Up.7D, EN.SM.Down.12H
**SCPEP1**		-1.4		1.051			-1.275		1.525		EN.SM.Up.7D, EN.SM.Down.12H
**ADAM22**		-3.033			4.441		-3.037				EN.SM.Down.12H, EN.Only.Up.30D
**VWA5A**		-3.63	-1.995	1.369							EN.Only.Down.12H.24H, EN.Only.Up.7D
**APOC1**		-2.396	-1.696						3.581		EN.Only.Down.12H.24H, SM.Only.Up.7D
**PDIA5**		-2.135	-1.547						1.516		EN.Only.Down.12H.24H, SM.Only.Up.7D
**RASSF9**		-1.343		-2.025			1.28				EN.Only.Down.12H.7D, SM.Only.Up.12H
**MMP2**			-1.768	2.262			-1.118				EN.Only.Down.24H, SM.Only.Down.12H, EN.Only.Up.7D
**NOC3L**		-2.289		-2.345			1.164				EN.Only.Down.7D, EN.SM.Down.12H, SM.Only.Up.12H
**TCP1**		1.164					-1.753	-2.192			EN.Only.Up.12H
**ANKRD1**				1.627			-1.425		-1.058		EN.Only.Up.7D
**F2R**				2.046			-2.245		-1.581		EN.Only.Up.7D
**ZFAND6**		-1.027	1.52				-1.068				EN.SM.Down.12H, EN.Only.Up.24H
**CADM1**		-1.973		1.968			-1.204				EN.SM.Down.12H, EN.Only.Up.7D
**GPX1**		-1.002		1.369			-1.208				EN.SM.Down.12H, EN.Only.Up.7D
**ASAH1**		-1.163					-1.033		1.073		EN.SM.Down.12H, SM.Only.Up.7D
**C1QC**		-3.527					-1.608		1.976		EN.SM.Down.12H, SM.Only.Up.7D
**CDKN2C**		-3.225					-2.763		1.257		EN.SM.Down.12H, SM.Only.Up.7D
**COL16A1**		-1.527					-1.021		1.621		EN.SM.Down.12H, SM.Only.Up.7D
**COPZ2**		-1.307					-1.349		1.856		EN.SM.Down.12H, SM.Only.Up.7D
**DKK2**		-1.08					-1.215		1.889		EN.SM.Down.12H, SM.Only.Up.7D
**HMCN1**		-2.45					-2.423		1.604		EN.SM.Down.12H, SM.Only.Up.7D
**IFI44**		-1.807					-2.501		1.46		EN.SM.Down.12H, SM.Only.Up.7D
**IFI44L**		-2.163					-2.263		2.368		EN.SM.Down.12H, SM.Only.Up.7D
**KIF20B**		-1.311					-1.352		1.515		EN.SM.Down.12H, SM.Only.Up.7D
**LOC403888**		-2.312					-2.082		1.936		EN.SM.Down.12H, SM.Only.Up.7D
**LOC478990**		-1.252					-1.798		1.151		EN.SM.Down.12H, SM.Only.Up.7D
**LOC479114**		-1.518					-1.552		1.236		EN.SM.Down.12H, SM.Only.Up.7D
**LOC488359**		-1.174					-1.149		2.013		EN.SM.Down.12H, SM.Only.Up.7D
**SCN9A**		-1.382					-1.803		1.378		EN.SM.Down.12H, SM.Only.Up.7D
**WDR78**		-3.252					-1.758		1.304		EN.SM.Down.12H, SM.Only.Up.7D
**ZDHHC12**		-1.332					-1.136		1.917		EN.SM.Down.12H, SM.Only.Up.7D
**PPP1R3B**		1.708		-1.726					-1.924		EN.SM.Down.7D, EN.Only.Up.12H
**KCNJ8**				-1.653			2.303		-1.238		EN.SM.Down.7D, SM.Only.Up.12H
**PHACTR1**				-1.152			1.213		-1.369		EN.SM.Down.7D, SM.Only.Up.12H
**C6**				3.537			-2.59	-2.457			SM.Only.Down.12H.24H, EN.Only.Up.7D
**LRRK1**				1.472			-1.717	-1.951			SM.Only.Down.12H.24H, EN.Only.Up.7D
**MAP4**				1.37			-1.165		-1.006		SM.Only.Down.12H.7D
**RFX2**	1.328						-1.141		-1.276		SM.Only.Down.12H.7D, EN.Only.Up.2H
**IGF1**		-4.219					-1.311				EN.Only.Down.12H
**UBXN6**		-1.014						-1.295			EN.Only.Down.12H, SM.Only.Down.24H
**ATP11A**		-1.354							-2.132		EN.Only.Down.12H, SM.Only.Down.7D
**FOXP2**		-1.027							-1.028		EN.Only.Down.12H, SM.Only.Down.7D
**GPM6B**		-1.955							-1.039		EN.Only.Down.12H, SM.Only.Down.7D
**GPR98**		-1.542							-1.67		EN.Only.Down.12H, SM.Only.Down.7D
**HOXB5**		-1.298							-1.378		EN.Only.Down.12H, SM.Only.Down.7D
**LOC476674**		-1.392							-1.411		EN.Only.Down.12H, SM.Only.Down.7D
**MLLT6**		-1.284							-1.065		EN.Only.Down.12H, SM.Only.Down.7D
**NLGN4X**		-1.413							-1.366		EN.Only.Down.12H, SM.Only.Down.7D
**ABCC5**		-1.243	-1.337								EN.Only.Down.12H.24H
**ABLIM3**		-1.402	-1.168								EN.Only.Down.12H.24H
**ANKMY2**		-1.06	-1.028								EN.Only.Down.12H.24H
**CCDC18**		-1.576	-1.027								EN.Only.Down.12H.24H
**CDO1**		-2.197	-2.349								EN.Only.Down.12H.24H
**DKK3**		-1.861	-1.832								EN.Only.Down.12H.24H
**DOCK9**		-1.388	-1.35								EN.Only.Down.12H.24H
**DUOX1**		-1.773	-1.067								EN.Only.Down.12H.24H
**EGFL7**		-1.142	-1.365								EN.Only.Down.12H.24H
**FGD5**		-1.261	-1.129								EN.Only.Down.12H.24H
**FUNDC1**		-1.113	-1.252								EN.Only.Down.12H.24H
**FZD6**		-1.408	-1.215								EN.Only.Down.12H.24H
**GPR116**		-1.573	-2.326								EN.Only.Down.12H.24H
**GPR126**		-2.043	-1.672								EN.Only.Down.12H.24H
**GRIA3**		-1.521	-1.847								EN.Only.Down.12H.24H
**HMGCLL1**		-2.754	-1.541								EN.Only.Down.12H.24H
**HP1BP3**		-1.239	-1.109								EN.Only.Down.12H.24H
**JUP**		-1.364	-1.621								EN.Only.Down.12H.24H
**KANK3**		-1.85	-2.096								EN.Only.Down.12H.24H
**KCNN3**		-2.06	-2.221								EN.Only.Down.12H.24H
**LOC479105**		-1.666	-1.33								EN.Only.Down.12H.24H
**LOC480468**		-2.338	-2.867								EN.Only.Down.12H.24H
**LOC480722**		-1.16	-1.317								EN.Only.Down.12H.24H
**LOC482844**		-2.638	-1.77								EN.Only.Down.12H.24H
**LOC609044**		-2.231	-2.749								EN.Only.Down.12H.24H
**MYL9**		-1.104	-1.575								EN.Only.Down.12H.24H
**PERP**		-1.506	-1.826								EN.Only.Down.12H.24H
**PIK3R3**		-2.658	-2.943								EN.Only.Down.12H.24H
**PKHD1L1**		-2.415	-2.415								EN.Only.Down.12H.24H
**PTGS1**		-1.806	-2.259								EN.Only.Down.12H.24H
**SLC4A4**		-2.13	-1.35								EN.Only.Down.12H.24H
**SNCAIP**		-1.172	-1.472								EN.Only.Down.12H.24H
**SON**		-3.149	-4.518								EN.Only.Down.12H.24H
**STOX1**		-1.581	-1.999								EN.Only.Down.12H.24H
**TSPAN13**		-2.381	-2.189								EN.Only.Down.12H.24H
**ANKRD55**		-3.337		-3.428							EN.Only.Down.12H.7D
**CALCRL**		-1.42		-1.078							EN.Only.Down.12H.7D
**CLEC1A**		-1.348		-1.156							EN.Only.Down.12H.7D
**CLEC9A**		-1.848		-1.553							EN.Only.Down.12H.7D
**GABRE**		-1.319		-1.294							EN.Only.Down.12H.7D
**GALT**		-1.596		-1.239							EN.Only.Down.12H.7D
**KSR1**		-1.112		-1.172							EN.Only.Down.12H.7D
**LOC612322**		-2.469		-2.377							EN.Only.Down.12H.7D
**VEZF1**		-1.043		-1.047							EN.Only.Down.12H.7D
**ALS2**			-1.633				-1.348				EN.Only.Down.24H, SM.Only.Down.12H
**ANKRD35**			-1.351				-1.33				EN.Only.Down.24H, SM.Only.Down.12H
**ARHGAP28**			-2.269				-1.069				EN.Only.Down.24H, SM.Only.Down.12H
**B9D1**			-1.313				-1.543				EN.Only.Down.24H, SM.Only.Down.12H
**CCDC53**			-1.14				-1.304				EN.Only.Down.24H, SM.Only.Down.12H
**CORO6**			-2.238				-1.42				EN.Only.Down.24H, SM.Only.Down.12H
**EGLN1**			-1.04				-1.132				EN.Only.Down.24H, SM.Only.Down.12H
**ENOSF1**			-2.705				-1.809				EN.Only.Down.24H, SM.Only.Down.12H
**EPDR1**			-1.478				-1.112				EN.Only.Down.24H, SM.Only.Down.12H
**FAT4**			-2.004				-1.362				EN.Only.Down.24H, SM.Only.Down.12H
**FBXL7**			-1.218				-1.097				EN.Only.Down.24H, SM.Only.Down.12H
**FGD6**			-1.675				-1.271				EN.Only.Down.24H, SM.Only.Down.12H

**HECA**			-1.119				-1.144				EN.Only.Down.24H, SM.Only.Down.12H
**HIP1**			-1.028				-1.821				EN.Only.Down.24H, SM.Only.Down.12H
**ITGA1**			-1.219				-1.109				EN.Only.Down.24H, SM.Only.Down.12H
**ITGB5**			-1.511				-2.311				EN.Only.Down.24H, SM.Only.Down.12H
**KLHL3**			-1.635				-1.06				EN.Only.Down.24H, SM.Only.Down.12H
**LOC475575**			-1.037				-1.015				EN.Only.Down.24H, SM.Only.Down.12H
**LOC479268**			-1.175				-1.098				EN.Only.Down.24H, SM.Only.Down.12H
**LOC484338**			-1.852				-1.027				EN.Only.Down.24H, SM.Only.Down.12H
**LOC490390**			-1.904				-1.176				EN.Only.Down.24H, SM.Only.Down.12H
**LOC607729**			-2.058				-1.121				EN.Only.Down.24H, SM.Only.Down.12H
**LOC608135**			-1.219				-1.651				EN.Only.Down.24H, SM.Only.Down.12H
**LOC608883**			-1.932				-1.301				EN.Only.Down.24H, SM.Only.Down.12H
**MFGE8**			-1.023				-1.082				EN.Only.Down.24H, SM.Only.Down.12H
**MYL6B**			-1.281				-1.41				EN.Only.Down.24H, SM.Only.Down.12H
**NDRG4**			-1.404				-1.361				EN.Only.Down.24H, SM.Only.Down.12H
**PDGFD**			-1.876				-1.929				EN.Only.Down.24H, SM.Only.Down.12H
**PHTF2**			-1.462				-1.783				EN.Only.Down.24H, SM.Only.Down.12H
**PIK3IP1**			-2.744				-1.545				EN.Only.Down.24H, SM.Only.Down.12H
**PLEKHG5**			-1.49				-1.672				EN.Only.Down.24H, SM.Only.Down.12H
**RELL1**			-1.116				-1.35				EN.Only.Down.24H, SM.Only.Down.12H
**RGAG4**			-1.308				-1.315				EN.Only.Down.24H, SM.Only.Down.12H
**SMAD3**			-1.157				-1.048				EN.Only.Down.24H, SM.Only.Down.12H
**TCEAL3**			-1.536				-1.67				EN.Only.Down.24H, SM.Only.Down.12H
**THNSL2**			-2.283				-1.21				EN.Only.Down.24H, SM.Only.Down.12H
**TPCN1**			-1.629				-1.857				EN.Only.Down.24H, SM.Only.Down.12H
**ZDHHC2**			-1.707				-1.388				EN.Only.Down.24H, SM.Only.Down.12H
**ZNF30**			-1.802				-1.378				EN.Only.Down.24H, SM.Only.Down.12H
**CIRBP**			-1.905						-1.455		EN.Only.Down.24H, SM.Only.Down.7D
**EZH1**			-1.665						-1.781		EN.Only.Down.24H, SM.Only.Down.7D
**SORCS3**			-1.351						-1.318		EN.Only.Down.24H, SM.Only.Down.7D
**TTC19**			-1.576						-1.711		EN.Only.Down.24H, SM.Only.Down.7D
**ALDH1A2**			-2.678	-3.303							EN.Only.Down.24H.7D
**CACNA1C**			-1.239	-1.095							EN.Only.Down.24H.7D
**CDH6**			-2.478	-2.23							EN.Only.Down.24H.7D
**MTMR6**			-1.083	-1.19							EN.Only.Down.24H.7D
**CALHM2**	-2.763						-2.083				EN.Only.Down.2H, SM.Only.Down.12H
**DHRS7**	-2.235						-1.054				EN.Only.Down.2H, SM.Only.Down.12H
**GSPT2**	-1.253						-1.877				EN.Only.Down.2H, SM.Only.Down.12H
**SETMAR**	-2.06						-1.188				EN.Only.Down.2H, SM.Only.Down.12H
**SLC22A16**	-3.188						-1.354				EN.Only.Down.2H, SM.Only.Down.12H
**ZNF606**	-1.658						-1.33				EN.Only.Down.2H, SM.Only.Down.12H
**ZNF845**	-2.346						-1.326				EN.Only.Down.2H, SM.Only.Down.12H
**HISPPD1**	-2.205	-1.206									EN.Only.Down.2H.12H
**NASP**	-1.426	-1.076									EN.Only.Down.2H.12H
**RGS12**	-2.332	-2.621									EN.Only.Down.2H.12H
**LOC477172**	-1.491			-1.472							EN.Only.Down.2H.7D
**FBXO3**				-1.033			-3.298				EN.Only.Down.7D
**BCAS3**				-1.378			-1.509				EN.Only.Down.7D, SM.Only.Down.12H
**CAMK2G**				-1.172			-1.594				EN.Only.Down.7D, SM.Only.Down.12H
**CRISPLD1**				-2.023			-1.715				EN.Only.Down.7D, SM.Only.Down.12H
**ESRRG**				-1.587			-1.505				EN.Only.Down.7D, SM.Only.Down.12H
**EXTL1**				-1.455			-1.614				EN.Only.Down.7D, SM.Only.Down.12H
**GSG1**				-1.324			-1.687				EN.Only.Down.7D, SM.Only.Down.12H
**HSPB8**				-1.019			-1.209				EN.Only.Down.7D, SM.Only.Down.12H
**IGLON5**				-1.053			-1.379				EN.Only.Down.7D, SM.Only.Down.12H
**LOC476778**				-1.203			-1.079				EN.Only.Down.7D, SM.Only.Down.12H
**LOC477528**				-1.29			-1.063				EN.Only.Down.7D, SM.Only.Down.12H
**LOC610770**				-1.35			-1.081				EN.Only.Down.7D, SM.Only.Down.12H
**MSH2**				-1.365			-1.835				EN.Only.Down.7D, SM.Only.Down.12H
**NRN1**				-3.51			-1.388				EN.Only.Down.7D, SM.Only.Down.12H
**PARD3B**				-1.144			-1.124				EN.Only.Down.7D, SM.Only.Down.12H
**PCSK7**				-1.545			-1.628				EN.Only.Down.7D, SM.Only.Down.12H
**PLCL1**				-1.573			-1.598				EN.Only.Down.7D, SM.Only.Down.12H
**PRR12**				-1.05			-1.313				EN.Only.Down.7D, SM.Only.Down.12H
**RASL11A**				-1.489			-1.659				EN.Only.Down.7D, SM.Only.Down.12H
**RET**				-1.092			-1.344				EN.Only.Down.7D, SM.Only.Down.12H
**SH3BGRL2**				-1.242			-1.018				EN.Only.Down.7D, SM.Only.Down.12H
**SYDE2**				-2.232			-1.021				EN.Only.Down.7D, SM.Only.Down.12H
**TOM1L1**				-1.575			-1.519				EN.Only.Down.7D, SM.Only.Down.12H
**XK**				-1.335			-2.538				EN.Only.Down.7D, SM.Only.Down.12H
**ZNF662**				-2.064			-1.264				EN.Only.Down.7D, SM.Only.Down.12H
**ZYG11A**				-1.606			-1.374				EN.Only.Down.7D, SM.Only.Down.12H
**RPGR**				-1.162				-1.762			EN.Only.Down.7D, SM.Only.Down.24H
**ST7L**				-1.207				-1.403			EN.Only.Down.7D, SM.Only.Down.24H
**4-Sep**		-1.695					-1.663				EN.SM.Down.12H
**ACAA1**		-1.596					-1.24				EN.SM.Down.12H
**ACAT1**		-1.53					-2.128				EN.SM.Down.12H
**ACTA2**		-1.304					-1.141				EN.SM.Down.12H
**ADAMTSL3**		-1.313					-1.773				EN.SM.Down.12H
**AER61**		-1.438					-1.395				EN.SM.Down.12H
**AGBL5**		-3.372					-2.832				EN.SM.Down.12H
**AKT3**		-1.078					-1.09				EN.SM.Down.12H
**ALDH6A1**		-2.099					-1.645				EN.SM.Down.12H
**ANGPT4**		-1.035					-1.332				EN.SM.Down.12H
**ANGPTL2**		-2.268					-1.574				EN.SM.Down.12H
**APP**		-1.334					-1.209				EN.SM.Down.12H
**ARHGEF6**		-2.175					-1.64				EN.SM.Down.12H
**ARMC9**		-2.868					-3.455				EN.SM.Down.12H
**AUH**		-1.352					-1.474				EN.SM.Down.12H
**B3GNT1**		-2.062					-2.002				EN.SM.Down.12H
**BAZ2B**		-1.307					-1.225				EN.SM.Down.12H
**BBX**		-1.659					-1.248				EN.SM.Down.12H
**BEND5**		-2.192					-2.217				EN.SM.Down.12H
**BGLAP**		-1.588					-1.177				EN.SM.Down.12H
**BNIP3**		-1.689					-1.893				EN.SM.Down.12H
**BRD8**		-1.101					-1.098				EN.SM.Down.12H
**BST1**		-1.421					-1.078				EN.SM.Down.12H
**C1QA**		-2.9					-1.464				EN.SM.Down.12H
**CAST**		-1.259					-1.258				EN.SM.Down.12H
**CAV2**		-1.456					-2.31				EN.SM.Down.12H
**CC2D2A**		-1.167					-1.056				EN.SM.Down.12H
**CCRL1**		-3.939					-1.426				EN.SM.Down.12H
**CD81**		-1.077					-1.229				EN.SM.Down.12H
**CD99**		-1.469					-1.753				EN.SM.Down.12H
**CDC14B**		-1.828					-1.508				EN.SM.Down.12H
**CDS2**		-1.595					-1.467				EN.SM.Down.12H
**CETN2**		-1.209					-1.196				EN.SM.Down.12H
**CHRDL1**		-1.515					-1.79				EN.SM.Down.12H
**CHURC1**		-1.034					-1.345				EN.SM.Down.12H
**CMBL**		-1.886					-1.587				EN.SM.Down.12H
**CNKSR3**		-1.171					-1.563				EN.SM.Down.12H
**COG1**		-1.387					-1.074				EN.SM.Down.12H
**CPEB3**		-1.253					-1.844				EN.SM.Down.12H
**CRTAP**		-1.446					-1.791				EN.SM.Down.12H
**CTDSPL**		-1.273					-1.185				EN.SM.Down.12H
**CUL4B**		-1.157					-1.335				EN.SM.Down.12H
**CXXC5**		-1.913					-1.296				EN.SM.Down.12H
**DAG1**		-1.486					-1.698				EN.SM.Down.12H
**DGKA**		-1.245					-1.243				EN.SM.Down.12H
**DPYSL2**		-1.631					-1.663				EN.SM.Down.12H
**DSG2**		-2.543					-1.444				EN.SM.Down.12H
**ECHS1**		-1.143					-1.231				EN.SM.Down.12H
**EML1**		-1.255					-1.34				EN.SM.Down.12H

EPB41L3		-1.693					-1.286				EN.SM.Down.12H
EPSTI1		-1.317					-1.361				EN.SM.Down.12H
FADS2		-1.458					-1.082				EN.SM.Down.12H
FAIM2		-1.918					-2.315				EN.SM.Down.12H
FARS2		-1.134					-1.176				EN.SM.Down.12H
FBP2		-1.11					-1.499				EN.SM.Down.12H
FBXO25		-2.247					-2.397				EN.SM.Down.12H
FGFR2		-2.232					-2.964				EN.SM.Down.12H
FGFR2IIIC		-1.205					-2.061				EN.SM.Down.12H
FOLH1		-2.239					-1.715				EN.SM.Down.12H
GATAD2B		-1.068					-1.149				EN.SM.Down.12H
GCDH		-1.327					-1.35				EN.SM.Down.12H
GLB1		-1.148					-1.072				EN.SM.Down.12H
GLB1L		-1.151					-1.104				EN.SM.Down.12H
GLCE		-1.152					-1.673				EN.SM.Down.12H
GNB3		-1.564					-1.082				EN.SM.Down.12H
GNB5		-1.604					-1.656				EN.SM.Down.12H
GPI		-1.229					-1.545				EN.SM.Down.12H
HBP1		-1.564					-1.177				EN.SM.Down.12H
HEBP1		-1.5					-1.668				EN.SM.Down.12H
HINT2		-1.653					-1.545				EN.SM.Down.12H
HOXA10		-1.109					-1.231				EN.SM.Down.12H
IFI35		-1.613					-2.119				EN.SM.Down.12H
IGF1R		-1.045					-1.311				EN.SM.Down.12H
IGFBP2		-1.33					-1.616				EN.SM.Down.12H
IGFBP6		-2.174					-1.109				EN.SM.Down.12H
INSR		-2.228					-1.443				EN.SM.Down.12H
ITGA7		-1.436					-1.698				EN.SM.Down.12H
ITGB8		-2.001					-2.642				EN.SM.Down.12H
ITM2C		-1.403					-1.199				EN.SM.Down.12H
JAZF1		-1.465					-1.338				EN.SM.Down.12H
JMY		-1.407					-1.382				EN.SM.Down.12H
KCNIP3		-1.662					-1.506				EN.SM.Down.12H
KLHL24		-2.779					-2.685				EN.SM.Down.12H
LEP		-1.755					-3.565				EN.SM.Down.12H
LOC100174968		-1.876					-1.76				EN.SM.Down.12H
LOC474570		-2.298					-1.817				EN.SM.Down.12H
LOC474791		-1.425					-1.911				EN.SM.Down.12H
LOC475290		-1.189					-1.482				EN.SM.Down.12H
LOC475409		-1.925					-1.521				EN.SM.Down.12H
LOC475470		-1.489					-1.112				EN.SM.Down.12H
LOC476006		-2.086					-1.542				EN.SM.Down.12H
LOC477395		-1.163					-1.026				EN.SM.Down.12H
LOC477419		-1.2					-1.002				EN.SM.Down.12H
LOC478584		-1.549					-1.851				EN.SM.Down.12H
LOC478866		-1.568					-1.42				EN.SM.Down.12H
LOC479382		-1.285					-1.71				EN.SM.Down.12H
LOC479637		-1.101					-1.219				EN.SM.Down.12H
LOC481385		-1.31					-1.675				EN.SM.Down.12H
LOC482349		-1.283					-1.73				EN.SM.Down.12H
LOC482730		-1.779					-1.865				EN.SM.Down.12H
LOC483174		-1.609					-1.75				EN.SM.Down.12H
LOC484139		-1.057					-1.25				EN.SM.Down.12H
LOC485723		-1.504					-1.156				EN.SM.Down.12H
LOC488476		-1.363					-1.533				EN.SM.Down.12H
LOC489202		-1.384					-2.001				EN.SM.Down.12H
LOC490089		-1.33					-1.593				EN.SM.Down.12H
LOC490956		-1.417					-1.48				EN.SM.Down.12H
LOC607161		-1.182					-1.558				EN.SM.Down.12H
LOC607548		-1.53					-1.741				EN.SM.Down.12H
LOC607830		-2.073					-1.7				EN.SM.Down.12H
LOC608557		-2.562					-2.651				EN.SM.Down.12H
LOC609039		-1.548					-1.349				EN.SM.Down.12H
LOC610195		-2.211					-1.804				EN.SM.Down.12H
LOC610260		-2.348					-2.847				EN.SM.Down.12H
LOC610433		-1.214					-1.3				EN.SM.Down.12H
LOC611081		-1.259					-1.26				EN.SM.Down.12H
LOC611487		-1.536					-1.579				EN.SM.Down.12H
LOC611851		-1.317					-1.054				EN.SM.Down.12H
LRRC28		-1.004					-1.165				EN.SM.Down.12H
LYPLAL1		-1.284					-1.736				EN.SM.Down.12H
LZTS2		-1.208					-1.366				EN.SM.Down.12H
MAGEF1		-1.323					-1.432				EN.SM.Down.12H
MAGI3		-2.136					-1.952				EN.SM.Down.12H
MANEA		-1.046					-1.114				EN.SM.Down.12H
MAP2		-1.684					-1.573				EN.SM.Down.12H
MAP7D3		-1.095					-1.046				EN.SM.Down.12H
MBTD1		-1.798					-1.293				EN.SM.Down.12H
MMP16		-1.992					-1.05				EN.SM.Down.12H
MOCS2		-1.058					-1.168				EN.SM.Down.12H
MPZL1		-1.164					-1.112				EN.SM.Down.12H
MRGPRF		-1.196					-1.597				EN.SM.Down.12H
MRVI1		-1.736					-2.123				EN.SM.Down.12H
MSRB3		-1.619					-1.727				EN.SM.Down.12H
MYLK		-1.378					-1.374				EN.SM.Down.12H
MYLK3		-1.286					-1.314				EN.SM.Down.12H
NAPEPLD		-1.354					-1.209				EN.SM.Down.12H
NBEA		-1.745					-1.55				EN.SM.Down.12H
NDFIP1		-1.049					-1.191				EN.SM.Down.12H
NDUFA3		-1.197					-1.15				EN.SM.Down.12H
NDUFB10		-1.486					-1.452				EN.SM.Down.12H
NDUFS7		-1.171					-1.387				EN.SM.Down.12H
NME5		-2.238					-2.195				EN.SM.Down.12H
NOC2L		-2.2					-1.232				EN.SM.Down.12H
NRGN		-1.48					-1.068				EN.SM.Down.12H
NUDT18		-1.482					-1.163				EN.SM.Down.12H
NUDT2		-1.579					-1.683				EN.SM.Down.12H
NUPR1		-2.322					-1.529				EN.SM.Down.12H
OMD		-2.964					-2.223				EN.SM.Down.12H
OSBPL1A		-1.279					-1.152				EN.SM.Down.12H
PARP2		-2.12					-1.612				EN.SM.Down.12H
PARVA		-1.178					-1.422				EN.SM.Down.12H
PCMTD1		-1.195					-1.572				EN.SM.Down.12H
PDE4DIP		-1.287					-1.175				EN.SM.Down.12H
PDK4		-2.635					-1.815				EN.SM.Down.12H
PEBP1		-1.773					-1.592				EN.SM.Down.12H
PER3		-2.623					-3.125				EN.SM.Down.12H
PFKM		-1.565					-2.057				EN.SM.Down.12H
PGAP1		-1.286					-2.043				EN.SM.Down.12H
PGM2L1		-1.494					-1.438				EN.SM.Down.12H
PIAS2		-1.332					-1.175				EN.SM.Down.12H
PLCXD3		-1.799					-1.248				EN.SM.Down.12H
PNCK		-1.008					-1.475				EN.SM.Down.12H
PPL		-2.239					-2.352				EN.SM.Down.12H
PREX2		-1.842					-1.606				EN.SM.Down.12H
PRIM1		-1.553					-1.759				EN.SM.Down.12H
PRKRA		-1.252					-1.125				EN.SM.Down.12H
PROS1		-2.339					-1.678				EN.SM.Down.12H
PRTFDC1		-1.761					-2.725				EN.SM.Down.12H
PSIP1		-1.412					-1.085				EN.SM.Down.12H
PTGFRN		-1.058					-1.311				EN.SM.Down.12H
PTP4A3		-3.457					-2.913				EN.SM.Down.12H
PTPLAD2		-1.321					-1.188				EN.SM.Down.12H

PTPN13		-2.291					-2.276				EN.SM.Down.12H
PYGB		-1.431					-1.512				EN.SM.Down.12H
RBP4		-1.529					-1.39				EN.SM.Down.12H
RCOR3		-1.275					-1.137				EN.SM.Down.12H
RMND5A		-1.128					-1.523				EN.SM.Down.12H
RNASEH2C		-1.023					-1.087				EN.SM.Down.12H
RNASEL		-1.518					-2.081				EN.SM.Down.12H
RNASEN		-1.835					-1.175				EN.SM.Down.12H
RNF180		-1.883					-1.543				EN.SM.Down.12H
RNLS		-1.968					-1.951				EN.SM.Down.12H
ROR1		-1.578					-1.135				EN.SM.Down.12H
RPH3AL		-1.775					-1.069				EN.SM.Down.12H
RSU1		-1.029					-1.175				EN.SM.Down.12H
RWDD3		-1.477					-1.148				EN.SM.Down.12H
S100A1		-2.205					-1.677				EN.SM.Down.12H
S1PR3		-1.203					-1.061				EN.SM.Down.12H
SESN1		-1.219					-1.199				EN.SM.Down.12H
SGCE		-1.822					-1.6				EN.SM.Down.12H
SGTB		-1.318					-1.332				EN.SM.Down.12H
SLC2A8		-1.852					-1.173				EN.SM.Down.12H
SLC43A3		-1.503					-1.262				EN.SM.Down.12H
SMAD9		-1.434					-1.715				EN.SM.Down.12H
SMARCA1		-1.76					-1.709				EN.SM.Down.12H
SNTA1		-1.42					-1.504				EN.SM.Down.12H
SPATA6		-1.425					-1.296				EN.SM.Down.12H
SPATA7		-1.237					-1.188				EN.SM.Down.12H
ST7		-1.424					-1.668				EN.SM.Down.12H
STAU2		-1.272					-1.228				EN.SM.Down.12H
STON1-GTF2A1L		-1.285					-1.688				EN.SM.Down.12H
STXBP6		-1.307					-1.191				EN.SM.Down.12H
SUOX		-1.311					-1.284				EN.SM.Down.12H
SYT11		-1.451					-1.259				EN.SM.Down.12H
SYT9		-1.627					-2.281				EN.SM.Down.12H
TCEA2		-1.645					-1.546				EN.SM.Down.12H
TDRD7		-1.11					-1.392				EN.SM.Down.12H
TEX264		-1.269					-1.106				EN.SM.Down.12H
TFDP2		-1.303					-1.198				EN.SM.Down.12H
THRA		-1.349					-1.435				EN.SM.Down.12H
TM2D2		-1.752					-1.298				EN.SM.Down.12H
TMEM132D		-1.005					-1.016				EN.SM.Down.12H
TMEM176A		-2.127					-1.706				EN.SM.Down.12H
TMEM43		-1.101					-1.35				EN.SM.Down.12H
TNFAIP8L3		-1.294					-1.617				EN.SM.Down.12H
TRMT11		-1.235					-1.049				EN.SM.Down.12H
TSHZ1		-1.558					-1.094				EN.SM.Down.12H
TSNAXIP1		-1.29					-1.087				EN.SM.Down.12H
TTC14		-1.636					-1.54				EN.SM.Down.12H
TTC21B		-1.5					-2.067				EN.SM.Down.12H
TTC3		-1.106					-1.126				EN.SM.Down.12H
UBE2CBP		-1.158					-1.243				EN.SM.Down.12H
UBE2E3		-1.013					-1.217				EN.SM.Down.12H
UBR5		-1.01					-1.076				EN.SM.Down.12H
UTRN		-1.24					-1.567				EN.SM.Down.12H
VAMP4		-1.072					-1.003				EN.SM.Down.12H
VPS11		-1.142					-1.141				EN.SM.Down.12H
YPEL2		-2.118					-1.555				EN.SM.Down.12H
ZBTB20		-2.358					-2.366				EN.SM.Down.12H
ZBTB25		-1.828					-1.255				EN.SM.Down.12H
ZFHX3		-1.359					-1.464				EN.SM.Down.12H
ZFP106		-1.624					-1.325				EN.SM.Down.12H
ZFYVE1		-1.484					-1.548				EN.SM.Down.12H
ZNF365		-1.305					-1.453				EN.SM.Down.12H
ZNF568		-1.065					-1.154				EN.SM.Down.12H
ZNF575		-1.661					-2.209				EN.SM.Down.12H
ZNF711		-1.763					-1.191				EN.SM.Down.12H
INPP4B			-2.14					-1.399			EN.SM.Down.24H
LOC475625			-1.919					-2.1			EN.SM.Down.24H
ACTG2				-1.982					-2.307		EN.SM.Down.7D
ACY1				-1.191					-1.335		EN.SM.Down.7D
BEX4				-1.136					-1.116		EN.SM.Down.7D
CCND2				-1.27					-1.44		EN.SM.Down.7D
DNAH17				-2.369					-2.043		EN.SM.Down.7D
FLNC				-1.302					-1.609		EN.SM.Down.7D
FRMD3				-1.881					-1.586		EN.SM.Down.7D
GUCY1A2				-2.639					-1.607		EN.SM.Down.7D
KCNH2				-1.173					-1.28		EN.SM.Down.7D
LOC481434				-1.108					-1.496		EN.SM.Down.7D
LZTS1				-1.095					-1.888		EN.SM.Down.7D
MAP3K5				-1.19					-1.011		EN.SM.Down.7D
MAST4				-1.334					-1.303		EN.SM.Down.7D
PGAM5				-1.069					-1.321		EN.SM.Down.7D
SLC25A30				-1.602					-1.585		EN.SM.Down.7D
SRF				-1.448					-1.698		EN.SM.Down.7D
STC1				-1.982					-1.515		EN.SM.Down.7D
TMOD1				-1.081					-2.257		EN.SM.Down.7D
TP53I11				-1.086					-1.339		EN.SM.Down.7D
TPPP				-1.064					-1.196		EN.SM.Down.7D
TTN				-1.247					-1.578		EN.SM.Down.7D
WDR54				-1.03					-1.272		EN.SM.Down.7D
ZFAND3				-1.105					-1.737		EN.SM.Down.7D
ZNF689				-1.093					-1.72		EN.SM.Down.7D
FAIM		-1.918					-1.36				SM.Only.Down.12H
AADAC							-2.535	-2.216			SM.Only.Down.12H.24H
ACSL6							-1.099	-2.126			SM.Only.Down.12H.24H
AR							-2.232	-2.752			SM.Only.Down.12H.24H
ARMC2							-1.347	-1.588			SM.Only.Down.12H.24H
CCBL2							-1.447	-1.643			SM.Only.Down.12H.24H
CCL14							-1.173	-1.332			SM.Only.Down.12H.24H
CDON							-1.507	-1.848			SM.Only.Down.12H.24H
CITED2							-1.904	-1.847			SM.Only.Down.12H.24H
COLEC12							-2.434	-1.876			SM.Only.Down.12H.24H
DYSF							-1.411	-1.39			SM.Only.Down.12H.24H
GRID2							-1.273	-1.946			SM.Only.Down.12H.24H
ICA1L							-1.482	-2.509			SM.Only.Down.12H.24H
ITGA8							-1.939	-1.868			SM.Only.Down.12H.24H
LOC478877							-2.617	-2.757			SM.Only.Down.12H.24H
MGP							-2.011	-2.491			SM.Only.Down.12H.24H
MITF							-3.432	-1.984			SM.Only.Down.12H.24H
MOSC1							-1.584	-1.647			SM.Only.Down.12H.24H
NOS2							-1.281	-2.503			SM.Only.Down.12H.24H
OPHN1							-1.247	-2.063			SM.Only.Down.12H.24H
PIK3R2							-1.368	-1.967			SM.Only.Down.12H.24H
PKD2							-1.192	-2.518			SM.Only.Down.12H.24H
RAB12							-1.067	-1.765			SM.Only.Down.12H.24H
RBM24							-1.603	-2.225			SM.Only.Down.12H.24H
SFMBT2							-2.482	-2.681			SM.Only.Down.12H.24H
SH3GL2							-1.561	-2.315			SM.Only.Down.12H.24H
SPTB							-2.06	-3.442			SM.Only.Down.12H.24H
SPTBN1							-1.504	-1.365			SM.Only.Down.12H.24H
THSD4							-1.35	-1.619			SM.Only.Down.12H.24H
TMEM178							-2.162	-1.76			SM.Only.Down.12H.24H
TMX4							-1.374	-1.718			SM.Only.Down.12H.24H
UNC84A							-1.31	-1.384			SM.Only.Down.12H.24H

UPRT							-1.114	-1.371			SM.Only.Down.12H.24H
WDR66							-1.541	-1.646			SM.Only.Down.12H.24H
ABCA6							-3.541		-2.86		SM.Only.Down.12H.7D
ADAMTSL4							-1.085		-1.199		SM.Only.Down.12H.7D
AKAP11							-1.129		-1.069		SM.Only.Down.12H.7D
ALDH1L1							-1.297		-1.233		SM.Only.Down.12H.7D
ARHGAP12							-1.111		-1.178		SM.Only.Down.12H.7D
ARIH2							-1.043		-1.387		SM.Only.Down.12H.7D
ATP2A3							-1.498		-1.719		SM.Only.Down.12H.7D
CCBL1							-1.288		-1		SM.Only.Down.12H.7D
CCDC33							-1.083		-1.414		SM.Only.Down.12H.7D
CHST3							-1.262		-1.407		SM.Only.Down.12H.7D
CLASP2							-1.24		-1.089		SM.Only.Down.12H.7D
CRIM1							-1.127		-1.308		SM.Only.Down.12H.7D
DTNBP1							-1.059		-1.175		SM.Only.Down.12H.7D
F3							-1.013		-2.86		SM.Only.Down.12H.7D
FGF18							-1.353		-1.279		SM.Only.Down.12H.7D
HMCN2							-1.079		-1.713		SM.Only.Down.12H.7D
ISM1							-1.161		-1.096		SM.Only.Down.12H.7D
ITGB4							-1.488		-1.606		SM.Only.Down.12H.7D
KCNQ1							-1.062		-1.086		SM.Only.Down.12H.7D
KIF7							-1.074		-1.434		SM.Only.Down.12H.7D
LOC403447							-1.621		-1.144		SM.Only.Down.12H.7D
LOC487445							-1.271		-1.18		SM.Only.Down.12H.7D
LOC607837							-1.233		-2.667		SM.Only.Down.12H.7D
LOC611316							-1.189		-1.285		SM.Only.Down.12H.7D
LOC611522							-1.645		-1.312		SM.Only.Down.12H.7D
MAPRE3							-2.087		-1.212		SM.Only.Down.12H.7D
MT3							-1.355		-1.56		SM.Only.Down.12H.7D
MYOC							-1.42		-6.38		SM.Only.Down.12H.7D
NCAM1							-1.249		-1.117		SM.Only.Down.12H.7D
OSGEPL1							-1.291		-1.311		SM.Only.Down.12H.7D
PDLIM5							-1.749		-1.888		SM.Only.Down.12H.7D
PEX26							-1.721		-1.711		SM.Only.Down.12H.7D
PLB1							-1.246		-1.825		SM.Only.Down.12H.7D
PLXDC1							-2.026		-1.626		SM.Only.Down.12H.7D
POU2AF1							-1.104		-1.226		SM.Only.Down.12H.7D
PPP2R2B							-1.012		-1.265		SM.Only.Down.12H.7D
PPP2R5A							-1.105		-1.111		SM.Only.Down.12H.7D
PRKAA2							-1.157		-1.408		SM.Only.Down.12H.7D
PRPF4B							-2.132		-1.434		SM.Only.Down.12H.7D
PTPRD							-2.026		-2.219		SM.Only.Down.12H.7D
RBPMS2							-1.406		-1.639		SM.Only.Down.12H.7D
RRAGD							-1.306		-1.105		SM.Only.Down.12H.7D
STAC2							-1.129		-1.622		SM.Only.Down.12H.7D
STARD6							-1.085		-1.41		SM.Only.Down.12H.7D
TLN1							-1.078		-1.125		SM.Only.Down.12H.7D
TNFRSF19							-1.166		-1.773		SM.Only.Down.12H.7D
VPS26A							-1.056		-1.549		SM.Only.Down.12H.7D
ZBTB16							-3.073		-1.012		SM.Only.Down.12H.7D
ZDBF2							-1.091		-1.498		SM.Only.Down.12H.7D
ZFP36L1							-1.229		-1.227		SM.Only.Down.12H.7D
ZNF503							-1.143		-1.41		SM.Only.Down.12H.7D
ZRANB3							-1.497		-1.291		SM.Only.Down.12H.7D
CES2								-1.782	-1.652		SM.Only.Down.24H.7D
FGF1								-2.697	-1.285		SM.Only.Down.24H.7D
LOC478715								-1.53	-1.378		SM.Only.Down.24H.7D
TNS1								-1.545	-1.031		SM.Only.Down.24H.7D
MPZ		-1.164							-1.399		SM.Only.Down.7D
PON3	2.645	2.73	3.164				2.279	3.268	-2.219		EN.SM.Up.12H.24H, SM.Only.Down.7D, EN.Only.Up.2H
TFPI		5.194	5.695	6.444	4.655		-1.688				SM.Only.Down.12H
LUM				2.392	4.018		-1.145		1.097		EN.SM.Up.7D, SM.Only.Down.12H, EN.Only.Up.30D
P4HA3		-1.341		1.607			1.342		2.69		EN.Only.Down.12H, EN.SM.Up.7D, SM.Only.Up.12H
CRISPLD2	2.235	1.451		-1.809			1.588				EN.Only.Down.7D, EN.SM.Up.12H, EN.Only.Up.2H
LOC480396	4.123			-1.077		4.029	1.825				EN.Only.Down.7D, EN.SM.Up.2H, SM.Only.Up.12H
HTR7		1.533	2.228				1.936		-1.444		EN.SM.Up.12H, SM.Only.Down.7D, EN.Only.Up.24H
PRAM1		2.7	1.674				2.42		-1.566		EN.SM.Up.12H, SM.Only.Down.7D, EN.Only.Up.24H
DUSP16	2.857					2.594	1.907		-1.167		EN.SM.Up.2H, SM.Only.Down.7D, SM.Only.Up.12H
UBE2T			2.175	5.321			-1.027		1.014		EN.SM.Up.7D, SM.Only.Down.12H, EN.Only.Up.24H
LOC484877		-1.508		1.249					1.259		EN.Only.Down.12H, EN.SM.Up.7D
RUFY3		-1.395		1.186					1.085		EN.Only.Down.12H, EN.SM.Up.7D
CH25H		-2.386					1.63		2.08		EN.Only.Down.12H, SM.Only.Up.12H.7D
CD200			-1.854				1.344		1.927		EN.Only.Down.24H
SLC35B4	-1.587						1.115		1.03		EN.Only.Down.2H, SM.Only.Up.12H.7D
PPP2R1B		1.407	1.395	-1.228							EN.Only.Down.7D, EN.Only.Up.12H.24H
FST		1.861		-2.507			2.229				EN.Only.Down.7D, EN.SM.Up.12H
GTPBP4		1.045		-1.216			1.349				EN.Only.Down.7D, EN.SM.Up.12H
EPRS		1.057		-1.474					1.005		EN.Only.Down.7D, SM.Only.Up.7D, EN.Only.Up.12H
C9		1.161					1.29		-1.232		EN.SM.Up.12H, SM.Only.Down.7D
RPL35		1.998					2.073		-1.005		EN.SM.Up.12H, SM.Only.Down.7D
CAMK2D				1.059			-1.436		1.421		EN.SM.Up.7D, SM.Only.Down.12H
CD109				1.608			-1.113		2.643		EN.SM.Up.7D, SM.Only.Down.12H
CEP152				1.449			-1.135		1.465		EN.SM.Up.7D, SM.Only.Down.12H
DMXL2				1.399			-1.185		1.108		EN.SM.Up.7D, SM.Only.Down.12H
FKBP7				1.281			-1.191		2.043		EN.SM.Up.7D, SM.Only.Down.12H
FNDC1				1.094			-1.719		1.036		EN.SM.Up.7D, SM.Only.Down.12H
GINS1				1.308			-1.023		1.197		EN.SM.Up.7D, SM.Only.Down.12H
IDH1				1.481			-1.877		1.285		EN.SM.Up.7D, SM.Only.Down.12H
KIF4A				1.171			-1.28		1.006		EN.SM.Up.7D, SM.Only.Down.12H
LOC487907				1.315			-1.412		1.369		EN.SM.Up.7D, SM.Only.Down.12H
LOX				2.006			-1.62		2.843		EN.SM.Up.7D, SM.Only.Down.12H
MND1				2.367			-1.098		2.611		EN.SM.Up.7D, SM.Only.Down.12H
MXRA5				3.699			-1.67		4.205		EN.SM.Up.7D, SM.Only.Down.12H
PON2				1.094			-1.068		1.45		EN.SM.Up.7D, SM.Only.Down.12H
STMN1				1.776			-1.436		4.049		EN.SM.Up.7D, SM.Only.Down.12H
TBC1D4				1.318			-1.144		1.696		EN.SM.Up.7D, SM.Only.Down.12H
TTC39C				1.89			-1.721		1.341		EN.SM.Up.7D, SM.Only.Down.12H
MAL		1.18	1.235						-1.749		SM.Only.Down.7D
RNF2		1.766	1.772						-1.339		SM.Only.Down.7D
AKAP12	1.774	1.406							-1.491		SM.Only.Down.7D, EN.Only.Up.2H.12H
TXNRD1							1.289	1.25	-1.123		SM.Only.Down.7D, SM.Only.Up.12H.24H
IKZF2	-3.629				2.867						EN.Only.Down.2H, EN.Only.Up.30D
ADAMTS2					3.459		-1.186				EN.Only.Up.30D
TBC1D1		-1.012					1.191				EN.Only.Down.12H
BATF2	2.025	-1.779									EN.Only.Down.12H, EN.Only.Up.2H
LOC477365	1.715	-1.094									EN.Only.Down.12H, EN.Only.Up.2H
FCGRT		-1.127		1.131							EN.Only.Down.12H, EN.Only.Up.7D
SLC40A1		-1.378		1.084							EN.Only.Down.12H, EN.Only.Up.7D
KALRN		-1.004					1.117				EN.Only.Down.12H, SM.Only.Up.12H
ASB9		-1.019							1.893		EN.Only.Down.12H, SM.Only.Up.7D
ATP10D		-1.289							1.32		EN.Only.Down.12H, SM.Only.Up.7D
BRIP1		-1.288							1.196		EN.Only.Down.12H, SM.Only.Up.7D
DCLK1		-1.097							1.015		EN.Only.Down.12H, SM.Only.Up.7D
LBP		-1.345							1.407		EN.Only.Down.12H, SM.Only.Up.7D
MX1		-1.542							3.472		EN.Only.Down.12H, SM.Only.Up.7D
THSD1		-1.513							2.243		EN.Only.Down.12H, SM.Only.Up.7D
ZBTB1		-1.16							1.111		EN.Only.Down.12H, SM.Only.Up.7D
HIVEP2	1.465		-1.157								EN.Only.Down.24H, EN.Only.Up.2H
ACE			-1.558	1.573							EN.Only.Down.24H, EN.Only.Up.7D
FBLN1			-1.579	1.595							EN.Only.Down.24H, EN.Only.Up.7D
ASCC3			-1.192				1.027				EN.Only.Down.24H, SM.Only.Down.12H, SM.Only.Up.12H
MYH10			-1.021						1.021		EN.Only.Down.24H, SM.Only.Up.7D
PALLD			-1.716						1.275		EN.Only.Down.24H, SM.Only.Up.7D

**PCDHGA6**			-2.138						1.333		EN.Only.Down.24H, SM.Only.Up.7D
**SEMA5A**			-2.088						2.1		EN.Only.Down.24H, SM.Only.Up.7D
**LOC478351**	-1.981	2.368									EN.Only.Down.2H, EN.Only.Up.12H
**TBC1D7**	-1.384								1.456		EN.Only.Down.2H, SM.Only.Up.7D
**PAK1**				-1.163			1.516				EN.Only.Down.7D
**RRAD**	2.211			-1.78							EN.Only.Down.7D, EN.Only.Up.2H
**ECSCR**				-1.269			1.698				EN.Only.Down.7D, SM.Only.Up.12H
**LETM2**				-1.416			1.238				EN.Only.Down.7D, SM.Only.Up.12H
**SLC25A15**				-1.182			1.207				EN.Only.Down.7D, SM.Only.Up.12H
**UBE2E1**				-1.026			1.341				EN.Only.Down.7D, SM.Only.Up.12H
**AARS**			1.004					-2.623			EN.Only.Up.24H
**NMI**		-1.603					1.045				EN.SM.Down.12H, SM.Only.Up.12H
**ZNF208**		1.441					-1.137				SM.Only.Down.12H, EN.Only.Up.12H
**DEPDC6**			2.429				-1.634				SM.Only.Down.12H, EN.Only.Up.24H
**HDHD3**			2.497				-1.07				SM.Only.Down.12H, EN.Only.Up.24H
**C3VS**				1.765			-1.199				SM.Only.Down.12H, EN.Only.Up.7D
**DNM1**				1.21			-1.057				SM.Only.Down.12H, EN.Only.Up.7D
**EFCAB1**				1.541			-1.382				SM.Only.Down.12H, EN.Only.Up.7D
**FABP7**				2.618			-1.922				SM.Only.Down.12H, EN.Only.Up.7D
**FMN2**				2.25			-1.34				SM.Only.Down.12H, EN.Only.Up.7D
**HSPG2**				1.334			-1.006				SM.Only.Down.12H, EN.Only.Up.7D
**IQGAP2**				1.665			-1.174				SM.Only.Down.12H, EN.Only.Up.7D
**LOC607775**				1.607			-1.742				SM.Only.Down.12H, EN.Only.Up.7D
**OLFML3**				1.661			-1.204				SM.Only.Down.12H, EN.Only.Up.7D
**RASSF4**				1.027			-1.199				SM.Only.Down.12H, EN.Only.Up.7D
**TTC38**				1.496			-1.201				SM.Only.Down.12H, EN.Only.Up.7D
**ABCG2**							-1.532		1.028		SM.Only.Down.12H, SM.Only.Up.7D
**CCDC150**							-1.626		1.787		SM.Only.Down.12H, SM.Only.Up.7D
**DNASE2**							-1.484		1.021		SM.Only.Down.12H, SM.Only.Up.7D
**EGFLAM**							-1.695		1.555		SM.Only.Down.12H, SM.Only.Up.7D
**FKBP9**							-1.45		1.006		SM.Only.Down.12H, SM.Only.Up.7D
**HAUS1**							-1.254		1.208		SM.Only.Down.12H, SM.Only.Up.7D
**KLK4**							-1.72		3.056		SM.Only.Down.12H, SM.Only.Up.7D
**LOC478796**							-1.653		1.125		SM.Only.Down.12H, SM.Only.Up.7D
**LOC489001**							-1.21		1.086		SM.Only.Down.12H, SM.Only.Up.7D
**LOC607411**							-2.167		1.037		SM.Only.Down.12H, SM.Only.Up.7D
**LOC610051**							-1.347		2.204		SM.Only.Down.12H.7D, SM.Only.Up.7D
**VRK2**			1.29					-1.964			SM.Only.Down.24H, EN.Only.Up.24H
**NPEPPS**		1.393							-1.145		SM.Only.Down.7D, EN.Only.Up.12H
**SF1**		1.097							-1.15		SM.Only.Down.7D, EN.Only.Up.12H
**SFXN4**		1.175							-1.888		SM.Only.Down.7D, EN.Only.Up.12H
**SMEK1**		1.145							-1.421		SM.Only.Down.7D, EN.Only.Up.12H
**ADM**	1.657								-2.092		SM.Only.Down.7D, EN.Only.Up.2H
**HSP70**	2.061								-1.562		SM.Only.Down.7D, EN.Only.Up.2H
**NR4A1**	2.367								-1.795		SM.Only.Down.7D, EN.Only.Up.2H
**PPP1R15A**	2.023								-1.535		SM.Only.Down.7D, EN.Only.Up.2H
**ARFGAP2**							1.028		-1.024		SM.Only.Down.7D, SM.Only.Up.12H
**ARMC3**							1.126		-1.049		SM.Only.Down.7D, SM.Only.Up.12H
**FARSA**							1.351		-1.214		SM.Only.Down.7D, SM.Only.Up.12H
**LOC479778**							1.021		-1.381		SM.Only.Down.7D, SM.Only.Up.12H
**MAPK1IP1L**							1.206		-1.067		SM.Only.Down.7D, SM.Only.Up.12H
**RSAD1**							1.292		-1.93		SM.Only.Down.7D, SM.Only.Up.12H
**TMCO1**							1.134		-1.026		SM.Only.Down.7D, SM.Only.Up.12H
**TMEM185A**							1.282		-1.034		SM.Only.Down.7D, SM.Only.Up.12H
**VEGFA**							1.182		-1.05		SM.Only.Down.7D, SM.Only.Up.12H
**PRPF4**			-1.99				1.159				SM.Only.Up.12H
**PGM2**		-1.494							1.012		SM.Only.Up.7D
**PPARG**		-2.454							1.541		SM.Only.Up.7D
**8-Mar**		-1.415									EN.Only.Down.12H
**A2LD1**		-1.083									EN.Only.Down.12H
**ACADL**		-1.934									EN.Only.Down.12H
**ACSM2A**		-1.269									EN.Only.Down.12H
**ACSS3**		-1.373									EN.Only.Down.12H
**ADD1**		-1.242									EN.Only.Down.12H
**AFAP1L1**		-1.069									EN.Only.Down.12H
**AGBL3**		-1.562									EN.Only.Down.12H
**AHNAK**		-1.157									EN.Only.Down.12H
**AHNAK2**		-1.33									EN.Only.Down.12H
**ALDH3A2**		-1.038									EN.Only.Down.12H
**AMN1**		-1.192									EN.Only.Down.12H
**APBB2**		-1.235									EN.Only.Down.12H
**ARFGEF2**		-1.084									EN.Only.Down.12H
**ARHGDIB**		-1.465									EN.Only.Down.12H
**ARHGEF12**		-1.378									EN.Only.Down.12H
**ARRB1**		-1.256									EN.Only.Down.12H
**ASB13**		-1.17									EN.Only.Down.12H
**ASB5**		-1.724									EN.Only.Down.12H
**ATP5J**		-1.282									EN.Only.Down.12H
**BBS9**		-1.199									EN.Only.Down.12H
**CASC4**		-1.15									EN.Only.Down.12H
**CBR3**		-1.556									EN.Only.Down.12H
**CBX3**		-1.016									EN.Only.Down.12H
**CCDC39**		-1.005									EN.Only.Down.12H
**CCDC85B**		-1.647									EN.Only.Down.12H
**CCS**		-1.027									EN.Only.Down.12H
**CGNL1**		-1.791									EN.Only.Down.12H
**CLCN4**		-1.176									EN.Only.Down.12H
**COL13A1**		-1.075									EN.Only.Down.12H
**CRIPT**		-1.091									EN.Only.Down.12H
**CST6**		-1.39									EN.Only.Down.12H
**CYYR1**		-2.102									EN.Only.Down.12H
**DAK**		-1.28									EN.Only.Down.12H
**DBNDD2**		-1.027									EN.Only.Down.12H
**DDX43**		-1.061									EN.Only.Down.12H
**DENND5B**		-1.002									EN.Only.Down.12H
**DEPDC5**		-1.301									EN.Only.Down.12H
**DHRS3**		-1.063									EN.Only.Down.12H
**DHX40**		-1.077									EN.Only.Down.12H
**DNM3**		-1.419									EN.Only.Down.12H
**DPY19L1**		-1.429									EN.Only.Down.12H
**EFNA1**		-1.799									EN.Only.Down.12H
**EGLN3**		-1.971									EN.Only.Down.12H
**EHMT2**		-1.603									EN.Only.Down.12H
**EIF4EBP2**		-1.042									EN.Only.Down.12H
**EMP2**		-1.432									EN.Only.Down.12H
**EPHX4**		-1.946									EN.Only.Down.12H
**FBXO8**		-1.367									EN.Only.Down.12H
**FECH**		-1.572									EN.Only.Down.12H
**FGF16**		-1.948									EN.Only.Down.12H
**FZD3**		-1.184									EN.Only.Down.12H
**GAL3ST3**		-1.056									EN.Only.Down.12H
**GALNTL2**		-2.418									EN.Only.Down.12H
**GATSL3**		-1.267									EN.Only.Down.12H
**GLTSCR2**		-1.063									EN.Only.Down.12H
**GNA14**		-1.673									EN.Only.Down.12H
**GRK4**		-1.654									EN.Only.Down.12H
**GSTM3**		-1.147									EN.Only.Down.12H
**HADH**		-1.074									EN.Only.Down.12H
**HDDC3**		-1.353									EN.Only.Down.12H
**HOXB7**		-2.335									EN.Only.Down.12H
**IDS**		-1.079									EN.Only.Down.12H
**IL6ST**		-1.232									EN.Only.Down.12H
**KATNAL1**		-1.809									EN.Only.Down.12H

KCNAB1		-1.826									EN.Only.Down.12H
LIFR		-1.629									EN.Only.Down.12H
LIX1L		-1.004									EN.Only.Down.12H
LOC475076		-1.281									EN.Only.Down.12H
LOC475343		-1.024									EN.Only.Down.12H
LOC476501		-1.023									EN.Only.Down.12H
LOC476569		-1.684									EN.Only.Down.12H
LOC478183		-1.128									EN.Only.Down.12H
LOC478894		-1.409									EN.Only.Down.12H
LOC478967		-1.257									EN.Only.Down.12H
LOC479477		-1.015									EN.Only.Down.12H
LOC485665		-1.456									EN.Only.Down.12H
LOC487047		-1.086									EN.Only.Down.12H
LOC487114		-2.123									EN.Only.Down.12H
LOC487992		-1.693									EN.Only.Down.12H
LOC490381		-1.039									EN.Only.Down.12H
LOC490458		-1.35									EN.Only.Down.12H
LOC490815		-1.315									EN.Only.Down.12H
LOC491005		-1.539									EN.Only.Down.12H
LOC491964		-1.053									EN.Only.Down.12H
LOC606939		-1.551									EN.Only.Down.12H
LOC608597		-1.065									EN.Only.Down.12H
LOC608733		-1.66									EN.Only.Down.12H
LOC608829		-1.237									EN.Only.Down.12H
LOC610526		-1.389									EN.Only.Down.12H
LOC611562		-1.251									EN.Only.Down.12H
LOC611631		-1.258									EN.Only.Down.12H
LOC612336		-1.071									EN.Only.Down.12H
LOC612721		-1.63									EN.Only.Down.12H
LPAR1		-1.28									EN.Only.Down.12H
LRP2BP		-1.276									EN.Only.Down.12H
LRRC70		-2.424									EN.Only.Down.12H
LYRM2		-1.169									EN.Only.Down.12H
MAP1A		-1.103									EN.Only.Down.12H
MAP3K1		-2.172									EN.Only.Down.12H
MED30		-1.007									EN.Only.Down.12H
MEIS1		-1.143									EN.Only.Down.12H
MKL2		-1.864									EN.Only.Down.12H
MYLIP		-1.409									EN.Only.Down.12H
NDUFAF3		-1.159									EN.Only.Down.12H
NEK4		-1.021									EN.Only.Down.12H
NFAT5		-1.315									EN.Only.Down.12H
NFX1		-1.063									EN.Only.Down.12H
NISCH		-1.063									EN.Only.Down.12H
NPDC1		-1.674									EN.Only.Down.12H
NSL1		-1.094									EN.Only.Down.12H
OAZ2		-1.274									EN.Only.Down.12H
OFD1		-1.266									EN.Only.Down.12H
OGT		-1.052									EN.Only.Down.12H
OPTN		-1.207									EN.Only.Down.12H
OSBPL6		-1.439									EN.Only.Down.12H
OSBPL9		-1.647									EN.Only.Down.12H
PAPOLA		-1.061									EN.Only.Down.12H
PARP3		-1.362									EN.Only.Down.12H
PHF11		-1.026									EN.Only.Down.12H
PHF21A		-1.029									EN.Only.Down.12H
PLBD2		-1.451									EN.Only.Down.12H
PLD5		-1.849									EN.Only.Down.12H
PLXNB2		-1.065									EN.Only.Down.12H
PMM1		-1.137									EN.Only.Down.12H
PPFIA2		-1.063									EN.Only.Down.12H
PRRX1		-1.39									EN.Only.Down.12H
RALGDS		-1.68									EN.Only.Down.12H
RB1CC1		-1.201									EN.Only.Down.12H
RBM26		-1.098									EN.Only.Down.12H
RFTN1		-1.275									EN.Only.Down.12H
RNF141		-1.332									EN.Only.Down.12H
RTP4		-1.181									EN.Only.Down.12H
SAMD11		-1.983									EN.Only.Down.12H
SCG5		-1.778									EN.Only.Down.12H
SDC2		-1.434									EN.Only.Down.12H
SDR42E1		-3.014									EN.Only.Down.12H
SERPING1		-1.285									EN.Only.Down.12H
SERPINI1		-1.255									EN.Only.Down.12H
SESTD1		-1.08									EN.Only.Down.12H
SIPA1L1		-1.112									EN.Only.Down.12H
SLITRK4		-1.63									EN.Only.Down.12H
SMYD2		-1.211									EN.Only.Down.12H
SNTB2		-1.03									EN.Only.Down.12H
SPEF2		-2.001									EN.Only.Down.12H
ST8SIA1		-1.034									EN.Only.Down.12H
TARBP1		-1.04									EN.Only.Down.12H
TBCE		-1.675									EN.Only.Down.12H
TC2N		-2.752									EN.Only.Down.12H
TDRD3		-1.225									EN.Only.Down.12H
TEX14		-1.759									EN.Only.Down.12H
TEX2		-1.24									EN.Only.Down.12H
THAP10		-1.807									EN.Only.Down.12H
TLR3		-1.337									EN.Only.Down.12H
TMCC3		-1.772									EN.Only.Down.12H
TMEM134		-1.136									EN.Only.Down.12H
TMEM62		-1.341									EN.Only.Down.12H
TRDN		-1.091									EN.Only.Down.12H
TTC28		-2.145									EN.Only.Down.12H
TTC7B		-1.01									EN.Only.Down.12H
UEVLD		-1.565									EN.Only.Down.12H
VNN1		-1.067									EN.Only.Down.12H
VPS13A		-1.178									EN.Only.Down.12H
VWF		-1.124									EN.Only.Down.12H
WDR48		-1.605									EN.Only.Down.12H
WWP1		-1.399									EN.Only.Down.12H
ZKSCAN5		-1.138									EN.Only.Down.12H
ZMYM2		-1.352									EN.Only.Down.12H
ZMYND11		-1.001									EN.Only.Down.12H
ZNF292		-1.219									EN.Only.Down.12H
ZNF33A		-1.537									EN.Only.Down.12H
ZNF362		-1.07									EN.Only.Down.12H
ZNF404		-1.615									EN.Only.Down.12H
ZNF521		-1.503									EN.Only.Down.12H
ZNF726		-1.729									EN.Only.Down.12H
ZNF827		-1.015									EN.Only.Down.12H
ABCB1			-1.04								EN.Only.Down.24H
ABLIM2			-1.079								EN.Only.Down.24H
ACVR1			-1.317								EN.Only.Down.24H
ADAMTS5			-2.262								EN.Only.Down.24H
ADRA2A			-1.068								EN.Only.Down.24H
ADRA2C			-1.073								EN.Only.Down.24H
ARL9			-1.443								EN.Only.Down.24H
ATP1B1			-1.226								EN.Only.Down.24H
AVPI1			-1.262								EN.Only.Down.24H
B4GALT6			-1.141								EN.Only.Down.24H
CBLB			-1.4								EN.Only.Down.24H
CCR6			-1.005								EN.Only.Down.24H

CHST12			-1.446								EN.Only.Down.24H
COPG2			-1.081								EN.Only.Down.24H
cOR51C4			-2.728								EN.Only.Down.24H
CTSE			-2.095								EN.Only.Down.24H
CYPIIB11			-1.048								EN.Only.Down.24H
DCAF4			-1.289								EN.Only.Down.24H
ECE1			-1.173								EN.Only.Down.24H
EFCAB3			-2.616								EN.Only.Down.24H
EFCAB9			-1.986								EN.Only.Down.24H
EFNB2			-1.01								EN.Only.Down.24H
ELSPBP1			-1.222								EN.Only.Down.24H
FBXO7			-1.202								EN.Only.Down.24H
GPR124			-1.284								EN.Only.Down.24H
LOC474456			-1.665								EN.Only.Down.24H
LOC481567			-1.831								EN.Only.Down.24H
LOC482849			-1.32								EN.Only.Down.24H
LOC488758			-1.184								EN.Only.Down.24H
LOC491035			-1.428								EN.Only.Down.24H
LOC612235			-1.584								EN.Only.Down.24H
LRRTM1			-1.263								EN.Only.Down.24H
LTBP2			-2.018								EN.Only.Down.24H
LYSMD2			-1.447								EN.Only.Down.24H
MAOA			-1.138								EN.Only.Down.24H
MAP6			-1.839								EN.Only.Down.24H
MARK4			-1.614								EN.Only.Down.24H
MSRB2			-1.081								EN.Only.Down.24H
MSTO1			-1.206								EN.Only.Down.24H
MYBL1			-1.898								EN.Only.Down.24H
OCLN			-1.958								EN.Only.Down.24H
OLFML2A			-1.772								EN.Only.Down.24H
PCDHGA4			-2.14								EN.Only.Down.24H
PHF17			-1.066								EN.Only.Down.24H
PPP1R13L			-1.263								EN.Only.Down.24H
PRPF40B			-1.99								EN.Only.Down.24H
PTPRR			-1.775								EN.Only.Down.24H
RBL1			-1.287								EN.Only.Down.24H
SCN2A			-1.789								EN.Only.Down.24H
SFT2D2			-1.588								EN.Only.Down.24H
SLC25A21			-1.211								EN.Only.Down.24H
SLC35F1			-2.633								EN.Only.Down.24H
SOD1			-1.068								EN.Only.Down.24H
SSH1			-1.255								EN.Only.Down.24H
SSX2IP			-1.099								EN.Only.Down.24H
TAF9B			-1.271								EN.Only.Down.24H
TRIB2			-1.709								EN.Only.Down.24H
UBXN7			-1.276								EN.Only.Down.24H
ZBTB8B			-1.318								EN.Only.Down.24H
ZMYM4			-1.246								EN.Only.Down.24H
ZNF197			-1.389								EN.Only.Down.24H
ATXN2L	-1.73										EN.Only.Down.2H
BAT4	-1.192										EN.Only.Down.2H
CCDC32	-2.066										EN.Only.Down.2H
LOC479815	-2.743										EN.Only.Down.2H
LOC491116	-1.293										EN.Only.Down.2H
MITD1	-2.246										EN.Only.Down.2H
MTRR	-3.189										EN.Only.Down.2H
VPS25	-1.674										EN.Only.Down.2H
ANXA3				-1.017							EN.Only.Down.7D
ARHGAP15				-1.625							EN.Only.Down.7D
ASS1				-1.572							EN.Only.Down.7D
ASTN2				-2.248							EN.Only.Down.7D
ATP11B				-1.035							EN.Only.Down.7D
BCORL1				-1.11							EN.Only.Down.7D
CAPS2				-1.426							EN.Only.Down.7D
CCDC68				-2.068							EN.Only.Down.7D
CDC42BPA				-1.678							EN.Only.Down.7D
CLCN1				-1.224							EN.Only.Down.7D
CWF19L2				-1.548							EN.Only.Down.7D
CXCR7				-2.108							EN.Only.Down.7D
FUNDC2				-1.126							EN.Only.Down.7D
FXYD2				-1.613							EN.Only.Down.7D
GIPC1				-1.365							EN.Only.Down.7D
GPATCH1				-1.26							EN.Only.Down.7D
GTPBP5				-1.48							EN.Only.Down.7D
GTPBP8				-1.093							EN.Only.Down.7D
HOOK1				-1.139							EN.Only.Down.7D
IFT57				-1.019							EN.Only.Down.7D
IL1RAPL1				-1.059							EN.Only.Down.7D
KCNK16				-1.08							EN.Only.Down.7D
KLHL31				-1.633							EN.Only.Down.7D
LOC475003				-1.014							EN.Only.Down.7D
LOC477862				-1.535							EN.Only.Down.7D
LOC480155				-1.037							EN.Only.Down.7D
LOC480785				-1.397							EN.Only.Down.7D
LOC484412				-1.013							EN.Only.Down.7D
LOC487697				-1.132							EN.Only.Down.7D
LOC609263				-1.27							EN.Only.Down.7D
LOC609833				-1.065							EN.Only.Down.7D
LOC610934				-1.809							EN.Only.Down.7D
MMP23A				-2.283							EN.Only.Down.7D
MTMR11				-1.276							EN.Only.Down.7D
NR3C1				-1.08							EN.Only.Down.7D
PPAP2B				-1.133							EN.Only.Down.7D
PSEN2				-1.684							EN.Only.Down.7D
PYGL				-1.069							EN.Only.Down.7D
RAB11FIP2				-1.14							EN.Only.Down.7D
RASEF				-1.418							EN.Only.Down.7D
RG9MTD2				-1.144							EN.Only.Down.7D
RPRD1A				-1.068							EN.Only.Down.7D
SLC44A3				-1.123							EN.Only.Down.7D
TMEM56				-1.264							EN.Only.Down.7D
TOMM6				-1.058							EN.Only.Down.7D
TRIM23				-1.292							EN.Only.Down.7D
UPF3A				-1.254							EN.Only.Down.7D
ZBTB8A				-1.032							EN.Only.Down.7D
ZFP37				-1.614							EN.Only.Down.7D
ZNF287				-1.169							EN.Only.Down.7D
ZNF471				-1.433							EN.Only.Down.7D
ZNF678				-1.66							EN.Only.Down.7D
ZNF790				-1.013							EN.Only.Down.7D
8-Sep							-1.461				SM.Only.Down.12H
AASS							-1.232				SM.Only.Down.12H
ABCB6							-1.159				SM.Only.Down.12H
ABCD2							-1.595				SM.Only.Down.12H
ABI3BP							-1.342				SM.Only.Down.12H
ACTN4							-1.326				SM.Only.Down.12H
ADAMDEC1							-2.662				SM.Only.Down.12H
ADAMTS20							-1.186				SM.Only.Down.12H
ADAMTS6							-1.698				SM.Only.Down.12H
Adcy6							-1.065				SM.Only.Down.12H
AES							-1.08				SM.Only.Down.12H
AGA							-1.065				SM.Only.Down.12H
AGAP1							-1.318				SM.Only.Down.12H

AGL							-1.311				SM.Only.Down.12H
ALAD							-1.38				SM.Only.Down.12H
ALDH2							-1.17				SM.Only.Down.12H
ALOX12							-1.013				SM.Only.Down.12H
ALS2CL							-1.015				SM.Only.Down.12H
ANKRD29							-1.079				SM.Only.Down.12H
ANP32A							-1.189				SM.Only.Down.12H
ANXA9							-2.233				SM.Only.Down.12H
APLF							-1.039				SM.Only.Down.12H
APPL1							-1.118				SM.Only.Down.12H
AQP4							-1.474				SM.Only.Down.12H
ARHGAP18							-1.072				SM.Only.Down.12H
ARL2							-1.059				SM.Only.Down.12H
ARSA							-1.082				SM.Only.Down.12H
ASMTL							-1.103				SM.Only.Down.12H
ATF7IP							-1.179				SM.Only.Down.12H
ATG16L2							-1.695				SM.Only.Down.12H
ATL3							-1.049				SM.Only.Down.12H
ATPBD4							-1.069				SM.Only.Down.12H
ATPGD1							-1.175				SM.Only.Down.12H
BCAR3							-1.224				SM.Only.Down.12H
BCKDHB							-1.305				SM.Only.Down.12H
BICD2							-1.003				SM.Only.Down.12H
BPHL							-1.399				SM.Only.Down.12H
BRMS1L							-1.032				SM.Only.Down.12H
BTBD6							-1.037				SM.Only.Down.12H
BTBD8							-1.328				SM.Only.Down.12H
CA5B							-1.1				SM.Only.Down.12H
CAV1							-1.252				SM.Only.Down.12H
CCDC110							-1.148				SM.Only.Down.12H
CCDC112							-1.279				SM.Only.Down.12H
CCDC115							-1.024				SM.Only.Down.12H
CCDC148							-1.645				SM.Only.Down.12H
CCDC34							-1.084				SM.Only.Down.12H
CCDC50							-1.217				SM.Only.Down.12H
CCDC77							-1.829				SM.Only.Down.12H
CCDC91							-1.091				SM.Only.Down.12H
CCNA1							-1.05				SM.Only.Down.12H
CDH13							-1.093				SM.Only.Down.12H
CDKL3							-1.395				SM.Only.Down.12H
CENPM							-1.679				SM.Only.Down.12H
CEP290							-1.121				SM.Only.Down.12H
CETN3							-1.344				SM.Only.Down.12H
CFL2							-1.08				SM.Only.Down.12H
CITED1							-1.114				SM.Only.Down.12H
CLUAP1							-1.453				SM.Only.Down.12H
CMAS							-1.225				SM.Only.Down.12H
CNKSR2							-1.364				SM.Only.Down.12H
COL4A4							-1.183				SM.Only.Down.12H
COMMD3							-1.033				SM.Only.Down.12H
CORO1B							-1.005				SM.Only.Down.12H
CPEB4							-1.607				SM.Only.Down.12H
CPNE2							-1.362				SM.Only.Down.12H
CPVL							-1.352				SM.Only.Down.12H
CRYZL1							-1.038				SM.Only.Down.12H
CYB5R3							-1.327				SM.Only.Down.12H
DAB2IP							-1.226				SM.Only.Down.12H
DCLRE1A							-1.154				SM.Only.Down.12H
DDIT4L							-2.47				SM.Only.Down.12H
DDO							-1.25				SM.Only.Down.12H
DHRS1							-1.119				SM.Only.Down.12H
DHTKD1							-1.57				SM.Only.Down.12H
DNAH3							-1.013				SM.Only.Down.12H
DSEL							-1.065				SM.Only.Down.12H
DSPP							-2.178				SM.Only.Down.12H
DSTN							-1.213				SM.Only.Down.12H
DTD1							-1.175				SM.Only.Down.12H
ECEL1							-1.078				SM.Only.Down.12H
ECH1							-1.33				SM.Only.Down.12H
EEF2K							-1.211				SM.Only.Down.12H
EFCAB7							-1.68				SM.Only.Down.12H
EFEMP1							-2.371				SM.Only.Down.12H
EFNA5							-1.296				SM.Only.Down.12H
ELMOD1							-1.036				SM.Only.Down.12H
ELMOD2							-1.225				SM.Only.Down.12H
EPHB6							-1.639				SM.Only.Down.12H
FABP3							-1.118				SM.Only.Down.12H
FARP1							-1.421				SM.Only.Down.12H
FBXO17							-1.11				SM.Only.Down.12H
FDFT1							-1.345				SM.Only.Down.12H
FIS1							-1.262				SM.Only.Down.12H
FMO5							-2.062				SM.Only.Down.12H
FOXN3							-1.305				SM.Only.Down.12H
FREM2							-1.627				SM.Only.Down.12H
FZD4							-1.23				SM.Only.Down.12H
FZD5							-1.078				SM.Only.Down.12H
GAL							-1.209				SM.Only.Down.12H
GALNT12							-1.27				SM.Only.Down.12H
GAS8							-1.5				SM.Only.Down.12H
GBAS							-1.145				SM.Only.Down.12H
GKAP1							-1.246				SM.Only.Down.12H
GMPR							-2.17				SM.Only.Down.12H
GNAL							-1.543				SM.Only.Down.12H
GNB4							-1.118				SM.Only.Down.12H
GNPDA2							-1.025				SM.Only.Down.12H
GPC4							-1.021				SM.Only.Down.12H
GPD1L							-1.196				SM.Only.Down.12H
GPR155							-1.025				SM.Only.Down.12H
GRB14							-1.251				SM.Only.Down.12H
GYPC							-1.296				SM.Only.Down.12H
HCFC1R1							-1.095				SM.Only.Down.12H
HELZ							-1.153				SM.Only.Down.12H
HIF3A							-1.244				SM.Only.Down.12H
HNMT							-1.252				SM.Only.Down.12H
HNRPLL							-1.194				SM.Only.Down.12H
HOXA13							-1.902				SM.Only.Down.12H
HOXC4							-1.059				SM.Only.Down.12H
HOXD10							-1.468				SM.Only.Down.12H
HRASLS5							-1.157				SM.Only.Down.12H
HSD17B11							-1.182				SM.Only.Down.12H
HSD17B14							-1.878				SM.Only.Down.12H
HSPA1L							-1.046				SM.Only.Down.12H
HSPB3							-1.359				SM.Only.Down.12H
HTR1E							-3.214				SM.Only.Down.12H
IDH3G							-1.174				SM.Only.Down.12H
IFT81							-1.374				SM.Only.Down.12H
IGSF11							-1.798				SM.Only.Down.12H
INMT							-1.007				SM.Only.Down.12H
INPP4A							-1.373				SM.Only.Down.12H
INPP5F							-1.082				SM.Only.Down.12H
IQCB1							-1.303				SM.Only.Down.12H
IQCF1							-1.096				SM.Only.Down.12H
IRS1							-1.049				SM.Only.Down.12H

ITFG1							-1.121				SM.Only.Down.12H
ITGBL1							-1.185				SM.Only.Down.12H
IVD							-1.072				SM.Only.Down.12H
JAM3							-1.301				SM.Only.Down.12H
KCTD1							-1.115				SM.Only.Down.12H
KCTD9							-1.055				SM.Only.Down.12H
KIF13A							-1.232				SM.Only.Down.12H
KIF13B							-1.206				SM.Only.Down.12H
KIF22							-1.201				SM.Only.Down.12H
KLHDC5							-1.268				SM.Only.Down.12H
KLHL8							-1.145				SM.Only.Down.12H
KRT27							-1.027				SM.Only.Down.12H
KRT32							-1.064				SM.Only.Down.12H
KRT8							-1.619				SM.Only.Down.12H
L2HGDH							-2.098				SM.Only.Down.12H
LANCL1							-1.43				SM.Only.Down.12H
LARP6							-1.018				SM.Only.Down.12H
LATS1							-1.043				SM.Only.Down.12H
LCAT							-1.145				SM.Only.Down.12H
LDB1							-1.162				SM.Only.Down.12H
LGR4							-1.24				SM.Only.Down.12H
LIPA							-1.068				SM.Only.Down.12H
LNPEP							-1.13				SM.Only.Down.12H
LOC403446							-1.339				SM.Only.Down.12H
LOC474447							-1.969				SM.Only.Down.12H
LOC474640							-1.134				SM.Only.Down.12H
LOC475673							-1.579				SM.Only.Down.12H
LOC475685							-1.347				SM.Only.Down.12H
LOC475941							-1.095				SM.Only.Down.12H
LOC475952							-1.343				SM.Only.Down.12H
LOC476113							-1.755				SM.Only.Down.12H
LOC477486							-1.287				SM.Only.Down.12H
LOC477555							-1.499				SM.Only.Down.12H
LOC477569							-1.103				SM.Only.Down.12H
LOC477905							-1.69				SM.Only.Down.12H
LOC478326							-1.284				SM.Only.Down.12H
LOC478639							-1.101				SM.Only.Down.12H
LOC479151							-1.764				SM.Only.Down.12H
LOC479261							-1.753				SM.Only.Down.12H
LOC479416							-1.308				SM.Only.Down.12H
LOC479461							-1.983				SM.Only.Down.12H
LOC480099							-1.065				SM.Only.Down.12H
LOC480617							-1.134				SM.Only.Down.12H
LOC480670							-1.001				SM.Only.Down.12H
LOC481143							-1.326				SM.Only.Down.12H
LOC481152							-1.522				SM.Only.Down.12H
LOC481249							-1.083				SM.Only.Down.12H
LOC481315							-1.617				SM.Only.Down.12H
LOC481674							-1.642				SM.Only.Down.12H
LOC481825							-1.039				SM.Only.Down.12H
LOC481962							-1.629				SM.Only.Down.12H
LOC482590							-1.098				SM.Only.Down.12H
LOC483182							-1.115				SM.Only.Down.12H
LOC483188							-1.123				SM.Only.Down.12H
LOC483447							-2.112				SM.Only.Down.12H
LOC484172							-1.213				SM.Only.Down.12H
LOC485274							-1.093				SM.Only.Down.12H
LOC485375							-1.946				SM.Only.Down.12H
LOC486500							-1.088				SM.Only.Down.12H
LOC487095							-2.957				SM.Only.Down.12H
LOC487854							-1.144				SM.Only.Down.12H
LOC488576							-1.226				SM.Only.Down.12H
LOC488680							-1.306				SM.Only.Down.12H
LOC489283							-1.309				SM.Only.Down.12H
LOC490496							-1.639				SM.Only.Down.12H
LOC490814							-1.843				SM.Only.Down.12H
LOC490941							-1.251				SM.Only.Down.12H
LOC491385							-1.04				SM.Only.Down.12H
LOC607225							-1.388				SM.Only.Down.12H
LOC607322							-1.462				SM.Only.Down.12H
LOC607417							-1.218				SM.Only.Down.12H
LOC607485							-1.087				SM.Only.Down.12H
LOC608029							-1.437				SM.Only.Down.12H
LOC608130							-2.295				SM.Only.Down.12H
LOC608134							-1.084				SM.Only.Down.12H
LOC608406							-1.21				SM.Only.Down.12H
LOC608676							-1.447				SM.Only.Down.12H
LOC609015							-1.334				SM.Only.Down.12H
LOC609071							-1.15				SM.Only.Down.12H
LOC609433							-2.026				SM.Only.Down.12H
LOC609451							-1.096				SM.Only.Down.12H
LOC609535							-1.011				SM.Only.Down.12H
LOC609559							-1.203				SM.Only.Down.12H
LOC609736							-1.052				SM.Only.Down.12H
LOC610183							-1.092				SM.Only.Down.12H
LOC610231							-1.079				SM.Only.Down.12H
LOC610613							-2.054				SM.Only.Down.12H
LOC610841							-1.286				SM.Only.Down.12H
LOC610965							-1.51				SM.Only.Down.12H
LOC611196							-1.137				SM.Only.Down.12H
LOC611582							-1.086				SM.Only.Down.12H
LOC611583							-2.246				SM.Only.Down.12H
LOC611690							-1.036				SM.Only.Down.12H
LOC611699							-1.351				SM.Only.Down.12H
LOC611747							-1.333				SM.Only.Down.12H
LOC611930							-1.168				SM.Only.Down.12H
LOC612135							-1.248				SM.Only.Down.12H
LOC612165							-1.176				SM.Only.Down.12H
LOC612534							-1.804				SM.Only.Down.12H
LOC612672							-1.151				SM.Only.Down.12H
LPHN3							-2.141				SM.Only.Down.12H
LPIN1							-1.108				SM.Only.Down.12H
LRCH2							-1.08				SM.Only.Down.12H
LRRC14							-1.096				SM.Only.Down.12H
LSMD1							-1.354				SM.Only.Down.12H
MAB21L1							-1.195				SM.Only.Down.12H
MAF1							-2.457				SM.Only.Down.12H
MAN2A2							-1.398				SM.Only.Down.12H
MAP2K6							-1.086				SM.Only.Down.12H
MAP4K3							-1.101				SM.Only.Down.12H
MAP9							-1.123				SM.Only.Down.12H
MAPRE2							-1.463				SM.Only.Down.12H
MBNL2							-1.11				SM.Only.Down.12H
MCC							-1.165				SM.Only.Down.12H
MCF2L							-1.081				SM.Only.Down.12H
MCOLN2							-1.036				SM.Only.Down.12H
ME3							-1.429				SM.Only.Down.12H
MEGF10							-1.144				SM.Only.Down.12H
MERTK							-1.937				SM.Only.Down.12H
MFAP5							-1.053				SM.Only.Down.12H
MGST2							-1.356				SM.Only.Down.12H
MIA3							-1.153				SM.Only.Down.12H
MIPOL1							-1.143				SM.Only.Down.12H

MLXIPL							-1.3				SM.Only.Down.12H
MMP23B							-1.009				SM.Only.Down.12H
MPPE1							-1.328				SM.Only.Down.12H
MPST							-1.106				SM.Only.Down.12H
MSI2							-1.004				SM.Only.Down.12H
MST1R							-1.725				SM.Only.Down.12H
MTMR7							-1.565				SM.Only.Down.12H
MYO6							-1.936				SM.Only.Down.12H
MYOF							-1.067				SM.Only.Down.12H
MYOT							-1.447				SM.Only.Down.12H
NAALAD2							-1.08				SM.Only.Down.12H
NAE1							-1.029				SM.Only.Down.12H
NAP1L3							-1.851				SM.Only.Down.12H
NARF							-1.037				SM.Only.Down.12H
NBEAL1							-1.064				SM.Only.Down.12H
NDUFV1							-1.386				SM.Only.Down.12H
NHEDC2							-1.051				SM.Only.Down.12H
NOX4							-1.269				SM.Only.Down.12H
NPHP1							-2.596				SM.Only.Down.12H
NPR2							-1.644				SM.Only.Down.12H
NR2F6							-1.695				SM.Only.Down.12H
NRP2							-1.329				SM.Only.Down.12H
NSMCE4A							-1.078				SM.Only.Down.12H
NT5E							-2.454				SM.Only.Down.12H
NTNG2							-1.397				SM.Only.Down.12H
NUB1							-1.347				SM.Only.Down.12H
NUBPL							-1.085				SM.Only.Down.12H
NUDT7							-1.244				SM.Only.Down.12H
NXPH2							-1.48				SM.Only.Down.12H
OCA2							-1.18				SM.Only.Down.12H
OPTC							-1.106				SM.Only.Down.12H
OR08B05							-1.284				SM.Only.Down.12H
OSCP1							-1.2				SM.Only.Down.12H
OSR2							-1.331				SM.Only.Down.12H
OXCT1							-1.259				SM.Only.Down.12H
PAH							-1.096				SM.Only.Down.12H
PARK7							-1.223				SM.Only.Down.12H
PARN							-1.286				SM.Only.Down.12H
PARP8							-1.412				SM.Only.Down.12H
PBX3							-1.016				SM.Only.Down.12H
PCK1							-2.36				SM.Only.Down.12H
PDE1A							-2.815				SM.Only.Down.12H
PDK3							-1.489				SM.Only.Down.12H
PECR							-1.578				SM.Only.Down.12H
PEX7							-1.215				SM.Only.Down.12H
PHKA2							-1.085				SM.Only.Down.12H
PIPOX							-1.078				SM.Only.Down.12H
PITPNM1							-1.104				SM.Only.Down.12H
PLA2R1							-1.657				SM.Only.Down.12H
PLEKHA5							-1.041				SM.Only.Down.12H
PLEKHO1							-1.144				SM.Only.Down.12H
PLXDC2							-1.624				SM.Only.Down.12H
POMT1							-1.179				SM.Only.Down.12H
POT1							-1.117				SM.Only.Down.12H
PPAPDC3							-1.352				SM.Only.Down.12H
PPM1B							-1.412				SM.Only.Down.12H
PPM1E							-1.329				SM.Only.Down.12H
PPM1K							-1.516				SM.Only.Down.12H
PPP2R3A							-1.019				SM.Only.Down.12H
PRICKLE1							-2.653				SM.Only.Down.12H
PRKACA							-1.114				SM.Only.Down.12H
PRKCA							-1.335				SM.Only.Down.12H
PRKCI							-1.317				SM.Only.Down.12H
PRKDC							-1.961				SM.Only.Down.12H
PROCA1							-1.155				SM.Only.Down.12H
PRODH							-1.084				SM.Only.Down.12H
PRRG4							-1.64				SM.Only.Down.12H
PRSS23							-1.514				SM.Only.Down.12H
PSPC1							-1.111				SM.Only.Down.12H
PTPN14							-1.052				SM.Only.Down.12H
PTPRCAP							-1.135				SM.Only.Down.12H
PTPRM							-1.357				SM.Only.Down.12H
RAB30							-1.43				SM.Only.Down.12H
RAB38							-1.048				SM.Only.Down.12H
RAB3IP							-1.407				SM.Only.Down.12H
RABL4							-1.086				SM.Only.Down.12H
RAD51L1							-1.009				SM.Only.Down.12H
RAD51L3							-1.088				SM.Only.Down.12H
RANBP9							-1.136				SM.Only.Down.12H
RBM45							-1.425				SM.Only.Down.12H
RBM9							-1.418				SM.Only.Down.12H
RERE							-1.674				SM.Only.Down.12H
RFX7							-1.089				SM.Only.Down.12H
RGS22							-1.574				SM.Only.Down.12H
RGS7BP							-1.018				SM.Only.Down.12H
RHOQ							-1.087				SM.Only.Down.12H
RIN2							-1.614				SM.Only.Down.12H
RMND1							-1.33				SM.Only.Down.12H
RNF125							-1.312				SM.Only.Down.12H
ROBO2							-1.426				SM.Only.Down.12H
ROCK1							-1.091				SM.Only.Down.12H
RPAIN							-1.475				SM.Only.Down.12H
RPUSD1							-1.015				SM.Only.Down.12H
RTKN2							-1.042				SM.Only.Down.12H
RUSC2							-1.109				SM.Only.Down.12H
S100PBP							-1.21				SM.Only.Down.12H
SASH1							-1.325				SM.Only.Down.12H
SATB1							-1.097				SM.Only.Down.12H
SCCPDH							-1.093				SM.Only.Down.12H
SCN5A							-1.151				SM.Only.Down.12H
SCRN1							-1.156				SM.Only.Down.12H
SEPN1							-1.036				SM.Only.Down.12H
SERTAD4							-2.153				SM.Only.Down.12H
SFRS12IP1							-1.142				SM.Only.Down.12H
SFRS18							-1.055				SM.Only.Down.12H
SGCB							-1.057				SM.Only.Down.12H
SHPK							-1.094				SM.Only.Down.12H
SLC1A3							-1.011				SM.Only.Down.12H
SLC22A2							-1.568				SM.Only.Down.12H
SLC43A1							-1.111				SM.Only.Down.12H
SLC44A1							-1.291				SM.Only.Down.12H
SLC7A14							-1.147				SM.Only.Down.12H
SNTB1							-1.357				SM.Only.Down.12H
SNX25							-1.685				SM.Only.Down.12H
SOBP							-1.047				SM.Only.Down.12H
SORD							-1.419				SM.Only.Down.12H
SOX6							-1.119				SM.Only.Down.12H
SPIN4							-1.428				SM.Only.Down.12H
SPINT2							-1.006				SM.Only.Down.12H
SPON1							-1.332				SM.Only.Down.12H
SPTLC3							-1.918				SM.Only.Down.12H
STK33							-1.4				SM.Only.Down.12H
STK39							-1.113				SM.Only.Down.12H

**SULF1**							-1.353				SM.Only.Down.12H
**SULT1C4**							-1.646				SM.Only.Down.12H
**SYCE2**							-1.311				SM.Only.Down.12H
**TACC2**							-1.244				SM.Only.Down.12H
**TACR1**							-1.218				SM.Only.Down.12H
**TBL1X**							-1.046				SM.Only.Down.12H
**TBX5**							-1.097				SM.Only.Down.12H
**TCF7L1**							-1.151				SM.Only.Down.12H
**TGFB1I1**							-1.304				SM.Only.Down.12H
**THY1**							-1.365				SM.Only.Down.12H
**TMEM126A**							-1.035				SM.Only.Down.12H
**TMEM176B**							-1.199				SM.Only.Down.12H
**TMEM41B**							-1.447				SM.Only.Down.12H
**TMEM51**							-1.134				SM.Only.Down.12H
**TMEM67**							-1.373				SM.Only.Down.12H
**TMEM88**							-1.003				SM.Only.Down.12H
**TMEM98**							-1.43				SM.Only.Down.12H
**TMLHE**							-1.569				SM.Only.Down.12H
**TNNI3**							-1.15				SM.Only.Down.12H
**TPD52L1**							-1.43				SM.Only.Down.12H
**TPM2**							-1.525				SM.Only.Down.12H
**TPP1**							-1.201				SM.Only.Down.12H
**TRAF5**							-1.949				SM.Only.Down.12H
**TRAFD1**							-1.836				SM.Only.Down.12H
**TRERF1**							-1.093				SM.Only.Down.12H
**TRPC4**							-1.33				SM.Only.Down.12H
**TRPM3**							-1.981				SM.Only.Down.12H
**TTC12**							-1.072				SM.Only.Down.12H
**TTC21A**							-1.941				SM.Only.Down.12H
**TTC39B**							-1.678				SM.Only.Down.12H
**TTC8**							-1.509				SM.Only.Down.12H
**TUSC4**							-1.201				SM.Only.Down.12H
**TXNDC16**							-1.321				SM.Only.Down.12H
**UBAC1**							-1.051				SM.Only.Down.12H
**UBE4B**							-1.022				SM.Only.Down.12H
**UBR3**							-1.027				SM.Only.Down.12H
**UHRF1BP1L**							-1.184				SM.Only.Down.12H
**ULK4**							-1.096				SM.Only.Down.12H
**UPF3B**							-1.259				SM.Only.Down.12H
**USP25**							-1.479				SM.Only.Down.12H
**USP28**							-1.526				SM.Only.Down.12H
**VWA3B**							-1.215				SM.Only.Down.12H
**WASF3**							-1.734				SM.Only.Down.12H
**WDR52**							-1.899				SM.Only.Down.12H
**WDSUB1**							-1.047				SM.Only.Down.12H
**WEE1**							-1.44				SM.Only.Down.12H
**WHSC1L1**							-1.239				SM.Only.Down.12H
**WWC2**							-1.12				SM.Only.Down.12H
**XRCC6BP1**							-1.298				SM.Only.Down.12H
**YBX2**							-1.019				SM.Only.Down.12H
**YIPF1**							-1.181				SM.Only.Down.12H
**ZBED5**							-1.057				SM.Only.Down.12H
**ZBTB38**							-1.364				SM.Only.Down.12H
**ZC3H6**							-1.15				SM.Only.Down.12H
**ZFP161**							-1.532				SM.Only.Down.12H
**ZFP62**							-1.047				SM.Only.Down.12H
**ZFYVE21**							-1.118				SM.Only.Down.12H
**ZNF132**							-1.114				SM.Only.Down.12H
**ZNF157**							-1.46				SM.Only.Down.12H
**ZNF181**							-1.112				SM.Only.Down.12H
**ZNF215**							-1.285				SM.Only.Down.12H
**ZNF397OS**							-1.927				SM.Only.Down.12H
**ZNF483**							-1.118				SM.Only.Down.12H
**ZNF608**							-1.147				SM.Only.Down.12H
**AARS2**								-2.623			SM.Only.Down.24H
**ALPK3**								-1.646			SM.Only.Down.24H
**CATSPERG**								-1.608			SM.Only.Down.24H
**CKMT2**								-1.587			SM.Only.Down.24H
**GPRASP1**								-1.718			SM.Only.Down.24H
**KCNJ3**								-1.793			SM.Only.Down.24H
**LOC475030**								-1.392			SM.Only.Down.24H
**LOC479686**								-1.486			SM.Only.Down.24H
**LOC609175**								-1.25			SM.Only.Down.24H
**MEF2A**								-2.156			SM.Only.Down.24H
**OVGP1**								-1.629			SM.Only.Down.24H
**PIGO**								-1.588			SM.Only.Down.24H
**RGS5**								-4.353			SM.Only.Down.24H
**SERINC3**								-1.767			SM.Only.Down.24H
**SERPINF1**								-1.59			SM.Only.Down.24H
**STAG2**								-1.334			SM.Only.Down.24H
**ZNF227**								-1.765			SM.Only.Down.24H
**ACADVL**									-1.026		SM.Only.Down.7D
**ACOT12**									-1.228		SM.Only.Down.7D
**ACSBG2**									-1.052		SM.Only.Down.7D
**ALKBH1**									-1.044		SM.Only.Down.7D
**ANKMY1**									-1.113		SM.Only.Down.7D
**ANKRD46**									-1.152		SM.Only.Down.7D
**AP1G2**									-1.253		SM.Only.Down.7D
**APOM**									-1.193		SM.Only.Down.7D
**ARHGAP24**									-1.29		SM.Only.Down.7D
**ARHGAP5**									-1.092		SM.Only.Down.7D
**ARID5A**									-1.431		SM.Only.Down.7D
**ATG4B**									-1.045		SM.Only.Down.7D
**B3GALNT2**									-1.029		SM.Only.Down.7D
**B3GALT5**									-1.108		SM.Only.Down.7D
**BCL2L1**									-1.043		SM.Only.Down.7D
**BIRC6**									-1.623		SM.Only.Down.7D
**BOLA1**									-1.035		SM.Only.Down.7D
**CA8**									-1.04		SM.Only.Down.7D
**CADM2**									-1.182		SM.Only.Down.7D
**CANT1**									-1.057		SM.Only.Down.7D
**CAPZB**									-1.096		SM.Only.Down.7D
**CARD9**									-1.027		SM.Only.Down.7D
**CATSPER4**									-1.07		SM.Only.Down.7D
**CCDC106**									-1.237		SM.Only.Down.7D
**CCDC92**									-1.288		SM.Only.Down.7D
**CCDC97**									-1.337		SM.Only.Down.7D
**CCL24**									-1.205		SM.Only.Down.7D
**CCNL2**									-1.964		SM.Only.Down.7D
**CD34**									-1.533		SM.Only.Down.7D
**CD79A**									-1.086		SM.Only.Down.7D
**CD97**									-1.199		SM.Only.Down.7D
**CDC20B**									-1.448		SM.Only.Down.7D
**CDH29**									-1.095		SM.Only.Down.7D
**CDK2AP2**									-1.368		SM.Only.Down.7D
**CHADL**									-1.347		SM.Only.Down.7D
**CHST9**									-1.305		SM.Only.Down.7D
**CIZ1**									-1.068		SM.Only.Down.7D
**CLDN5**									-1.179		SM.Only.Down.7D
**CLIP4**									-1.208		SM.Only.Down.7D
**CLRN1**									-1.411		SM.Only.Down.7D
**CNR1**									-1.059		SM.Only.Down.7D
**CNTFR**									-1.57		SM.Only.Down.7D

**CNTN1**									-1.153		SM.Only.Down.7D
**cOR7R1**									-1.018		SM.Only.Down.7D
**cOR8V9**									-1.639		SM.Only.Down.7D
**CORO2A**									-1.022		SM.Only.Down.7D
**CPSF6**									-1.039		SM.Only.Down.7D
**CRBN**									-1.184		SM.Only.Down.7D
**CRH**									-1.005		SM.Only.Down.7D
**CRY2**									-1.315		SM.Only.Down.7D
**CSDE1**									-1.348		SM.Only.Down.7D
**CTNNA3**									-1.202		SM.Only.Down.7D
**DHDPSL**									-1.221		SM.Only.Down.7D
**DLX3**									-1.161		SM.Only.Down.7D
**DMXL1**									-1.093		SM.Only.Down.7D
**DNAJB12**									-1.162		SM.Only.Down.7D
**DSC1**									-1.006		SM.Only.Down.7D
**DUSP26**									-1.005		SM.Only.Down.7D
**DUSP3**									-1.006		SM.Only.Down.7D
**EDN1**									-1.245		SM.Only.Down.7D
**EGFL8**									-1.092		SM.Only.Down.7D
**EIF4E3**									-1.43		SM.Only.Down.7D
**ESRP2**									-1.07		SM.Only.Down.7D
**FAT3**									-1.548		SM.Only.Down.7D
**FBF1**									-1.341		SM.Only.Down.7D
**FBXW5**									-1.041		SM.Only.Down.7D
**FGL1**									-1.168		SM.Only.Down.7D
**FGL2**									-1.749		SM.Only.Down.7D
**FLI1**									-1.039		SM.Only.Down.7D
**FOSL2**									-1.133		SM.Only.Down.7D
**FREQ**									-1.249		SM.Only.Down.7D
**GAS7**									-1.046		SM.Only.Down.7D
**GGPS1**									-1.083		SM.Only.Down.7D
**GNPTG**									-1.086		SM.Only.Down.7D
**GRIPAP1**									-1.067		SM.Only.Down.7D
**HHIP**									-1.014		SM.Only.Down.7D
**HIRIP3**									-1.17		SM.Only.Down.7D
**HMG20B**									-1.093		SM.Only.Down.7D
**HOXA9**									-1.239		SM.Only.Down.7D
**HOXB8**									-1.168		SM.Only.Down.7D
**HS3ST1**									-1.065		SM.Only.Down.7D
**IGF2R**									-1.147		SM.Only.Down.7D
**IL17RD**									-1.119		SM.Only.Down.7D
**ISLR2**									-1.082		SM.Only.Down.7D
**ITIH5**									-1.114		SM.Only.Down.7D
**KCNQ4**									-1.708		SM.Only.Down.7D
**KDM5C**									-1.06		SM.Only.Down.7D
**KIF1C**									-1.455		SM.Only.Down.7D
**KIRREL3**									-1.073		SM.Only.Down.7D
**LGALS12**									-1.056		SM.Only.Down.7D
**LIPC**									-1.049		SM.Only.Down.7D
**LOC475369**									-1.825		SM.Only.Down.7D
**LOC475435**									-1.938		SM.Only.Down.7D
**LOC476036**									-1.285		SM.Only.Down.7D
**LOC478160**									-1.064		SM.Only.Down.7D
**LOC478286**									-1.181		SM.Only.Down.7D
**LOC479493**									-1.117		SM.Only.Down.7D
**LOC480782**									-1.807		SM.Only.Down.7D
**LOC481459**									-1.078		SM.Only.Down.7D
**LOC481636**									-1.149		SM.Only.Down.7D
**LOC482078**									-1.1		SM.Only.Down.7D
**LOC482361**									-1.262		SM.Only.Down.7D
**LOC482687**									-1.12		SM.Only.Down.7D
**LOC482885**									-1.107		SM.Only.Down.7D
**LOC483305**									-1.182		SM.Only.Down.7D
**LOC483660**									-1.108		SM.Only.Down.7D
**LOC483848**									-1.629		SM.Only.Down.7D
**LOC484264**									-1.511		SM.Only.Down.7D
**LOC485036**									-1.156		SM.Only.Down.7D
**LOC485606**									-1.083		SM.Only.Down.7D
**LOC485960**									-1.002		SM.Only.Down.7D
**LOC485980**									-1.181		SM.Only.Down.7D
**LOC486280**									-1.008		SM.Only.Down.7D
**LOC486767**									-1.103		SM.Only.Down.7D
**LOC486982**									-2.02		SM.Only.Down.7D
**LOC489446**									-1.37		SM.Only.Down.7D
**LOC489456**									-1.223		SM.Only.Down.7D
**LOC490025**									-1.17		SM.Only.Down.7D
**LOC490625**									-1.017		SM.Only.Down.7D
**LOC490923**									-1.205		SM.Only.Down.7D
**LOC491275**									-1.264		SM.Only.Down.7D
**LOC606973**									-1.147		SM.Only.Down.7D
**LOC607038**									-1.038		SM.Only.Down.7D
**LOC607697**									-1.495		SM.Only.Down.7D
**LOC608537**									-1.153		SM.Only.Down.7D
**LOC608621**									-1.445		SM.Only.Down.7D
**LOC608882**									-1.081		SM.Only.Down.7D
**LOC609646**									-1.247		SM.Only.Down.7D
**LOC610136**									-1.01		SM.Only.Down.7D
**LOC611076**									-1.058		SM.Only.Down.7D
**LOC611305**									-1.33		SM.Only.Down.7D
**LOC611514**									-1.053		SM.Only.Down.7D
**LOC611652**									-1.075		SM.Only.Down.7D
**LOC611704**									-1.74		SM.Only.Down.7D
**LOC611772**									-1.165		SM.Only.Down.7D
**LOC612570**									-1.098		SM.Only.Down.7D
**LRP4**									-1.037		SM.Only.Down.7D
**LTBP4**									-1.701		SM.Only.Down.7D
**MACROD1**									-2.124		SM.Only.Down.7D
**MAP7D1**									-1.247		SM.Only.Down.7D
**MATN1**									-1.201		SM.Only.Down.7D
**MC3R**									-1.159		SM.Only.Down.7D
**MEGF8**									-1.623		SM.Only.Down.7D
**MFN2**									-1.198		SM.Only.Down.7D
**MLLT1**									-1.276		SM.Only.Down.7D
**MMAB**									-1.066		SM.Only.Down.7D
**MMP28**									-1.046		SM.Only.Down.7D
**MMRN1**									-1.586		SM.Only.Down.7D
**MOBKL2C**									-1.042		SM.Only.Down.7D
**MRPS18C**									-1.401		SM.Only.Down.7D
**MUS81**									-1.341		SM.Only.Down.7D
**MVK**									-1.109		SM.Only.Down.7D
**MYCBPAP**									-1.167		SM.Only.Down.7D
**MYH1**									-1.227		SM.Only.Down.7D
**MYH7**									-1.2		SM.Only.Down.7D
**MYH7B**									-1.083		SM.Only.Down.7D
**NACC2**									-1.082		SM.Only.Down.7D
**NAPA**									-1.242		SM.Only.Down.7D
**NEBL**									-2.15		SM.Only.Down.7D
**NEURL4**									-1.166		SM.Only.Down.7D
**NFYC**									-1.098		SM.Only.Down.7D
**NIT2**									-1.084		SM.Only.Down.7D
**NLRP1**									-1.784		SM.Only.Down.7D
**NOS1AP**									-1.134		SM.Only.Down.7D
**NPY2R**									-1.569		SM.Only.Down.7D

**NR1H2**									-1.186		SM.Only.Down.7D
**NR4A2**									-1.379		SM.Only.Down.7D
**NSUN6**									-1.332		SM.Only.Down.7D
**NT5DC3**									-2.572		SM.Only.Down.7D
**NUDT22**									-1.108		SM.Only.Down.7D
**ODZ2**									-1.007		SM.Only.Down.7D
**ORAI3**									-1.166		SM.Only.Down.7D
**PAG1**									-1.092		SM.Only.Down.7D
**PAPLN**									-1.319		SM.Only.Down.7D
**PAX9**									-2.405		SM.Only.Down.7D
**PEX14**									-1.083		SM.Only.Down.7D
**PHF1**									-1.598		SM.Only.Down.7D
**PHF8**									-1.073		SM.Only.Down.7D
**PITPNA**									-1.031		SM.Only.Down.7D
**PLA2G10**									-1.02		SM.Only.Down.7D
**PPIE**									-1.028		SM.Only.Down.7D
**PPP5C**									-1.014		SM.Only.Down.7D
**PRDM16**									-1.01		SM.Only.Down.7D
**PRDM8**									-1.033		SM.Only.Down.7D
**PRSS21**									-1.033		SM.Only.Down.7D
**PSD**									-1.65		SM.Only.Down.7D
**RAD23A**									-1.048		SM.Only.Down.7D
**RASL12**									-1.059		SM.Only.Down.7D
**RGS6**									-1.272		SM.Only.Down.7D
**RHOBTB2**									-1.22		SM.Only.Down.7D
**RNF208**									-1.089		SM.Only.Down.7D
**RNF220**									-1.347		SM.Only.Down.7D
**RNPEPL1**									-1.977		SM.Only.Down.7D
**ROBO4**									-1.245		SM.Only.Down.7D
**RPRD1B**									-1.043		SM.Only.Down.7D
**SAFB2**									-1.205		SM.Only.Down.7D
**SCIN**									-1.756		SM.Only.Down.7D
**SEMA3B**									-1.255		SM.Only.Down.7D
**SEMA4C**									-1.139		SM.Only.Down.7D
**SETD5**									-1.022		SM.Only.Down.7D
**SFRS8**									-1.375		SM.Only.Down.7D
**SGK3**									-1.043		SM.Only.Down.7D
**SH3GLB2**									-1.995		SM.Only.Down.7D
**SHARPIN**									-1.181		SM.Only.Down.7D
**SLC17A3**									-1.113		SM.Only.Down.7D
**SLC35D3**									-1.051		SM.Only.Down.7D
**SLC9A3R2**									-1.397		SM.Only.Down.7D
**SLCO3A1**									-1.057		SM.Only.Down.7D
**SLCO5A1**									-1.25		SM.Only.Down.7D
**SLIT3**									-1.086		SM.Only.Down.7D
**SMC5**									-1.051		SM.Only.Down.7D
**SMOX**									-1.127		SM.Only.Down.7D
**SMPD4**									-1.087		SM.Only.Down.7D
**SNRNP70**									-1.243		SM.Only.Down.7D
**SPEG**									-1.253		SM.Only.Down.7D
**SPOP**									-1.131		SM.Only.Down.7D
**STRN3**									-1.044		SM.Only.Down.7D
**SV2A**									-2		SM.Only.Down.7D
**TAF3**									-1.057		SM.Only.Down.7D
**TAOK2**									-1.019		SM.Only.Down.7D
**TBX2**									-1.262		SM.Only.Down.7D
**TCN1**									-1.037		SM.Only.Down.7D
**TGM2**									-1.361		SM.Only.Down.7D
**TMEM110**									-1.269		SM.Only.Down.7D
**TMEM149**									-1.268		SM.Only.Down.7D
**TMPRSS9**									-1.102		SM.Only.Down.7D
**TOX4**									-1.412		SM.Only.Down.7D
**TRAK1**									-2.615		SM.Only.Down.7D
**TRIAP1**									-1.079		SM.Only.Down.7D
**TRIM28**									-1.113		SM.Only.Down.7D
**TSC22D3**									-1.01		SM.Only.Down.7D
**TSEN54**									-1.384		SM.Only.Down.7D
**TSPYL2**									-1.164		SM.Only.Down.7D
**UBA1**									-1.673		SM.Only.Down.7D
**UBAP1**									-1.167		SM.Only.Down.7D
**UBE2I**									-1.031		SM.Only.Down.7D
**UBXN11**									-1.006		SM.Only.Down.7D
**ULK3**									-1.096		SM.Only.Down.7D
**UNK**									-1.003		SM.Only.Down.7D
**UXS1**									-1.539		SM.Only.Down.7D
**VPS18**									-1.196		SM.Only.Down.7D
**WASL**									-1.07		SM.Only.Down.7D
**WISP2**									-1.246		SM.Only.Down.7D
**YIPF2**									-1.025		SM.Only.Down.7D
**YLPM1**									-1.057		SM.Only.Down.7D
**ZC3H18**									-2.139		SM.Only.Down.7D
**ZDHHC8**									-1.473		SM.Only.Down.7D
**ZFP36L2**									-1.771		SM.Only.Down.7D
**ZFPM2**									-1.077		SM.Only.Down.7D
**ZNF238**									-1.293		SM.Only.Down.7D
**ZNF395**									-1.073		SM.Only.Down.7D
**ZNF467**									-1.015		SM.Only.Down.7D
**ZNF687**									-1.027		SM.Only.Down.7D
**CDKN1A**	1.788	1.604	1.653	1.442	2.41		2.772	2.332	1.335		EN.SM.Up.12H.24H.7D, EN.Only.Up.2H.30D
**CLEC5A**		4.074	3.941	2.247	3.154		4.374	2.638	3.414	3.655	EN.SM.Up.12H.24H.7D.30D
**LAPTM5**	2.513	3.44	4.172	4.705	3.391		3.347	2.635	3.228		EN.SM.Up.12H.24H.7D, EN.Only.Up.2H.30D
**TFPI2**		5.194	5.695	6.444	4.655		6.457	7.567	7.14	5.79	EN.SM.Up.12H.24H.7D.30D
**LYZ**	1.837	1.99	2.631	2.222			4.468	2.573	2.826	2.385	EN.SM.Up.12H.24H.7D, SM.Only.Up.30D, EN.Only.Up.2H
**TMEM49**	1.639	1.426	2.107	1.635			2.792	2.972	3.462	2.854	EN.SM.Up.12H.24H.7D, SM.Only.Up.30D, EN.Only.Up.2H
**BIRC3**	2.939	1.334	1.241	2.5		2.611	1.352	1.646	1.313		EN.SM.Up.2H.12H.24H.7D
**SERPINE1**	4.07	2.405	2.322	2.425		3.144	2.632	3.584	2.761		EN.SM.Up.2H.12H.24H.7D
**SPP1**		3.216	4.01	6.375	2.322		3.144	3.878	3.944		EN.SM.Up.12H.24H.7D, EN.Only.Up.30D
**VCAN**		2.014	3.069	2.92	2.87		3.176	2.598	3.598		EN.SM.Up.12H.24H.7D, EN.Only.Up.30D
**ALOX5AP**		2.897	3.359	3.094	3.191		2.964	2.301	2.271		EN.SM.Up.12H.24H.7D, EN.Only.Up.30D
**IL18**	2.011	3.793	4.205	2.397	3.456		3.2		1.911		EN.SM.Up.12H.7D, EN.Only.Up.2H.24H.30D
**EGR1**	2.202	1.814	1.255		3.59		2.576	1.896		4.904	EN.SM.Up.12H.24H.30D, EN.Only.Up.2H
**CLEC12A**	3.625	2.497	2.794	3.424	4.438		1.732		1.132		EN.SM.Up.12H.7D, EN.Only.Up.2H.24H.30D
**SERPINE2**		1.371	2.765	2.559	5.204		2.697	2.065	2.331		EN.SM.Up.12H.24H.7D, EN.Only.Up.30D
**AIM1**	1.66	1.673	2.497	2.427			1.723	1.831	1.967		EN.SM.Up.12H.24H.7D, EN.Only.Up.2H
**ARHGAP9**	1.935	3.579	3.522	1.828			3.182	2.404	3.187		EN.SM.Up.12H.24H.7D, EN.Only.Up.2H
**CD44**	3.769	1.535	2.507	1.621			1.665	1.441	1.088		EN.SM.Up.12H.24H.7D, EN.Only.Up.2H
**FYB**	3.838	4.165	4.009	3.502			4.275	3.028	1.977		EN.SM.Up.12H.24H.7D, EN.Only.Up.2H
**GK**	2.487	3.16	3.575	2.585			3.708	2.092	1.378		EN.SM.Up.12H.24H.7D, EN.Only.Up.2H
**GMFG**	2.373	2.5	2.788	2.039			2.57	2.518	2.491		EN.SM.Up.12H.24H.7D, EN.Only.Up.2H
**IL8**	5.879	5.714	5.692	1.531			7.304	6.283	1.456		EN.SM.Up.12H.24H.7D, EN.Only.Up.2H
**KMO**	2.798	1.867	2.035	2.107			3.012	2.058	1.466		EN.SM.Up.12H.24H.7D, EN.Only.Up.2H
**LCP1**	2.251	3.573	4.38	4.434			3.466	2.382	2.555		EN.SM.Up.12H.24H.7D, EN.Only.Up.2H
**MTHFD2**	1.584	2.005	1.687	1.479			1.739	1.658	1.393		EN.SM.Up.12H.24H.7D, EN.Only.Up.2H
**NCKAP1L**	2.924	1.313	3.885	3.674			1.866	2.884	2.418		EN.SM.Up.12H.24H.7D, EN.Only.Up.2H
**NRG1**	2.216	2.808	2.964	2.454			1.998	1.678	1.223		EN.SM.Up.12H.24H.7D, EN.Only.Up.2H
**PLAUR**	2.498	3.647	2.729	1.568			3.729	4.981	2.917		EN.SM.Up.12H.24H.7D, EN.Only.Up.2H
**PRKCB**	2.517	3.375	3.317	3.132			3.852	4.842	2.487		EN.SM.Up.12H.24H.7D, EN.Only.Up.2H
**SERPINA1**	2.839	4.306	3.191	2.638			4.366	2.328	2.755		EN.SM.Up.12H.24H.7D, EN.Only.Up.2H
**SLC11A1**	2.582	1.59	2.143	3.667			1.898	2.346	2.411		EN.SM.Up.12H.24H.7D, EN.Only.Up.2H
**TACC3**	1.983	2.103	2.362	2.348			2.693	2.359	1.514		EN.SM.Up.12H.24H.7D, EN.Only.Up.2H
**TMEM2**	2.276	1.383	1.384	1.077			2.781	2.8	1.502		EN.SM.Up.12H.24H.7D, EN.Only.Up.2H
**TYROBP**	2.548	3.724	4.339	3.978			4.401	2.441	2.739		EN.SM.Up.12H.24H.7D, EN.Only.Up.2H
**LCP2**	2.944	2.84	2.716	1.684			2.883	2.283	1.246		EN.SM.Up.12H.24H.7D, SM.Only.Down.7D, EN.Only.Up.2H

**FCGR1A**		4.354	5.554	3.768			4.404	3.628	3.938	2.886	EN.SM.Up.12H.24H.7D, SM.Only.Up.30D
**CCL4**	2.776	2.235	2.613			3.469	3.731	2.914	1.661		EN.SM.Up.2H.12H.24H, SM.Only.Up.7D
**SH3BP1**		2.836	3.278	4.131	3.433		3.42	2.512			EN.SM.Up.12H.24H, EN.Only.Up.7D.30D
**TOP2A**		1.579	3.215	5.564	3.839				5.416	4.067	EN.SM.Up.7D.30D, EN.Only.Up.12H.24H
**CCL7**	4.131	5.131	3.068	1.816			6.655	4.777			EN.SM.Up.12H.24H, EN.Only.Up.2H.7D
**CD4**	3.107	4.312	3.726	1.375			4.12	3.86			EN.SM.Up.12H.24H, EN.Only.Up.2H.7D
**LOC608848**	2.104	2.628	3.454	2.745			2.613	2.419			EN.SM.Up.12H.24H, EN.Only.Up.2H.7D
**OBFC2A**	1.988	3.059	3.349	2.009			3.407	2.793			EN.SM.Up.12H.24H, EN.Only.Up.2H.7D
**S100A8**	4.538	5.488	4.738	3.253			7.53	4.993			EN.SM.Up.12H.24H, EN.Only.Up.2H.7D
**SDC4**	2.998	1.558	1.641	1.155			1.39	2.175			EN.SM.Up.12H.24H, EN.Only.Up.2H.7D
**SORL1**	2.844	3.973	3.892	3.569			3.777	3.066			EN.SM.Up.12H.24H, EN.Only.Up.2H.7D
**CXCR4**		2.63	2.955	3.126		2.244	2.362	2.418			EN.SM.Up.12H.24H, SM.Only.Up.2H, EN.Only.Up.7D
**GNPNAT1**	2.624	1.918	1.223				2.214	1.899	1.089		EN.SM.Up.12H.24H, SM.Only.Up.7D, EN.Only.Up.2H
**HAS2**	2.526	2.591	1.767				5.049	4.378	1.572		EN.SM.Up.12H.24H, SM.Only.Up.7D, EN.Only.Up.2H
**ACOT7**		1.699	2.978	1.448			1.235	3.252	1.434		EN.SM.Up.12H.24H.7D
**APBB1IP**		2.63	4.285	2.675			1.203	3.302	1.974		EN.SM.Up.12H.24H.7D
**ASNS**		1.584	1.825	1.679			1.175	3.265	1.562		EN.SM.Up.12H.24H.7D
**ATP8B4**		2.396	2.649	2.05			2.223	2.514	1.645		EN.SM.Up.12H.24H.7D
**BCAT1**		1.14	3.159	3.978			1.382	3.214	2.848		EN.SM.Up.12H.24H.7D
**BCL2A1**		2.111	2.489	2.418			5.511	4.267	3.515		EN.SM.Up.12H.24H.7D
**BIN2**		3.622	2.451	2.641			3.146	2.492	2.493		EN.SM.Up.12H.24H.7D
**CALR**		1.267	1.829	1.26			1.018	1.917	1.07		EN.SM.Up.12H.24H.7D
**CCL2**		4.902	4.114	4.553			6.679	5.702	3.701		EN.SM.Up.12H.24H.7D
**CD48**		3.161	4.497	3.251			4.51	3.08	2.972		EN.SM.Up.12H.24H.7D
**CKAP4**		1.638	3.257	1.404			1.171	3.402	1.294		EN.SM.Up.12H.24H.7D
**CLEC4E**		2.998	3.597	3.2			3.572	2.656	2.312		EN.SM.Up.12H.24H.7D
**GLIPR1**		2.188	2.14	1.432			2.633	1.744	1.086		EN.SM.Up.12H.24H.7D
**HMGCR**		1.419	1.958	1.356			1.808	2.1	1.949		EN.SM.Up.12H.24H.7D
**IL6**		5.68	4.35	2.746			8.776	8.078	3.379		EN.SM.Up.12H.24H.7D
**LOC482041**		1.719	2.325	2.147			2.749	1.881	1.448		EN.SM.Up.12H.24H.7D
**LOC489680**		3.388	3.904	1.91			3.15	2.168	1.09		EN.SM.Up.12H.24H.7D
**LOC491080**		2.579	2.557	1.537			2.715	2.495	1.476		EN.SM.Up.12H.24H.7D
**LYN**		2.034	2.588	2.758			2.132	2.028	2.199		EN.SM.Up.12H.24H.7D
**MMP19**		1.683	3.667	3.284			2.856	2.963	2.26		EN.SM.Up.12H.24H.7D
**NCBP2**		1.819	1.598	1.41			1.258	1.597	1.331		EN.SM.Up.12H.24H.7D
**NME1**		2.278	2.651	2.148			3.477	3.528	1.995		EN.SM.Up.12H.24H.7D
**PLA2G7**		2.401	2.367	2.233			2.027	1.859	1.597		EN.SM.Up.12H.24H.7D
**PSAT1**		2.57	2.574	1.676			1.232	2.173	1.404		EN.SM.Up.12H.24H.7D
**PTPRC**		3.868	3.635	2.877			2.996	2.533	1.927		EN.SM.Up.12H.24H.7D
**RUNX1**		2.652	1.578	1.225			3.086	2.599	1.954		EN.SM.Up.12H.24H.7D
**RUNX2**		2.292	2.021	1.893			1.598	2.051	2.195		EN.SM.Up.12H.24H.7D
**SELL**		1.94	3.247	2.658			2.112	6.835	3.564		EN.SM.Up.12H.24H.7D
**SLC20A1**		1.353	2.234	1.522			1.842	1.916	1.604		EN.SM.Up.12H.24H.7D
**STK17B**		2.433	1.627	1.397			2.109	2.334	1.725		EN.SM.Up.12H.24H.7D
**TIMP1**		1.747	2.093	2.501			4.016	3.566	2.464		EN.SM.Up.12H.24H.7D
**UBASH3B**		2.558	2.666	1.547			3.134	3.54	1.857		EN.SM.Up.12H.24H.7D
**VASH2**		3.769	2.849	1.996			3.931	3.897	3.132		EN.SM.Up.12H.24H.7D
**SKAP2**		2.865	3.559	2.447			3.43	3.61	2.872		EN.SM.Up.12H.24H.7D, SM.Only.Down.7D
**AIF1**	1.734	2.175	3.452	2.21			2.614		2.326		EN.SM.Up.12H.7D, EN.Only.Up.2H.24H
**GNG2**	2.075	1.307	1.531	3.061			1.262		1.337		EN.SM.Up.12H.7D, EN.Only.Up.2H.24H
**GPR65**	1.794	3.649	3.913	3.105			2.696		2.357		EN.SM.Up.12H.7D, EN.Only.Up.2H.24H
**RAC2**	3.531	5.662	5.568	4.448			5.974		2.036		EN.SM.Up.12H.7D, EN.Only.Up.2H.24H
**SLA**	1.79	1.806	1.651	3.506			1.77		2.757		EN.SM.Up.12H.7D, EN.Only.Up.2H.24H
**CYBB**	2.056	2.791	4.138	4.586			1.697		1.744		EN.SM.Up.12H.7D, SM.Only.Down.7D, EN.Only.Up.2H.24H
**CD80**		1.302	1.337	1.762			1.091		1.556	2.864	EN.SM.Up.12H.7D, SM.Only.Up.30D, EN.Only.Up.24H
**COL5A2**				1.771	2.04			1.373	2.916	1.974	EN.SM.Up.7D.30D, SM.Only.Up.24H
**MPEG1**			1.287	1.444	3.386		1.878		3.563		EN.SM.Up.7D, SM.Only.Up.12H, EN.Only.Up.24H.30D
**DLA-DRA1**			4.017	3.803	3.588		1.682		3.174		EN.SM.Up.7D, SM.Only.Up.12H, EN.Only.Up.24H.30D
**C1S**				2.825	4.282		1.16		2.057	2.655	EN.SM.Up.7D.30D, SM.Only.Up.12H
**CLDN1**	3.842			2.811	4.495		1.429		3.432		EN.SM.Up.7D, SM.Only.Up.12H, EN.Only.Up.2H.30D
**DOCK8**		2.929	2.557	1.852			1.128		2.47		EN.Only.Down.24H, EN.SM.Up.12H.7D, EN.SM.Down.12H, EN.Only.Up.24H
**CFP**	2.341	3.453	4.012	2.495			3.435				EN.SM.Up.12H, EN.Only.Up.2H.24H.7D
**CORO1A**	2.552	2.015	1.701	2.656			1.676				EN.SM.Up.12H, EN.Only.Up.2H.24H.7D
**EMR1**	2.733	1.636	4.232	1.745			3.276				EN.SM.Up.12H, EN.Only.Up.2H.24H.7D
**FCER1G**	3.147	3.535	3.793	3.239			3.16				EN.SM.Up.12H, EN.Only.Up.2H.24H.7D
**LOC609461**	2.624	3.411	2.801	2.122			3.261				EN.SM.Up.12H, EN.Only.Up.2H.24H.7D
**NCF2**	1.452	1.871	1.827	1.563			1.635				EN.SM.Up.12H, EN.Only.Up.2H.24H.7D
**NCF4**	2.459	2.161	1.512	2.02			2.199				EN.SM.Up.12H, EN.Only.Up.2H.24H.7D
**PLEK**	2.966	3.766	2.918	1.812			3.727				EN.SM.Up.12H, EN.Only.Up.2H.24H.7D
**SELE**	5.598	5.393	4.482	1.041			1.934				EN.SM.Up.12H, EN.Only.Up.2H.24H.7D
**STK17A**	1.797	2.508	2.867	1.228			2.975				EN.SM.Up.12H, EN.Only.Up.2H.24H.7D
**TREM1**	2.263	4.408	4.856	2.409			4.159				EN.SM.Up.12H, EN.Only.Up.2H.24H.7D
**WDFY4**	3.168	1.435	3.046	3.586			1.24				EN.SM.Up.12H, EN.Only.Up.2H.24H.7D
**ZCWPW1**	3.599	1.094	4.374	3.026			4.023				EN.SM.Up.12H, EN.Only.Up.2H.24H.7D
**FOSL1**	3.455	2.772		1.03			3.781	2.799			EN.SM.Up.12H, SM.Only.Up.24H, EN.Only.Up.2H.7D
**P2RY13**	2.751	5.079	4.179				3.579		1.923		EN.SM.Up.12H, SM.Only.Up.7D, EN.Only.Up.2H.24H
**AQP9**	4.218	5.734	4.777				5.828	4.607			EN.SM.Up.12H.24H, EN.Only.Up.2H
**BAZ1A**	2.23	1.682	1.512				2.234	1.848			EN.SM.Up.12H.24H, EN.Only.Up.2H
**C5AR1**	3.88	5.132	6.033				3.339	3.908			EN.SM.Up.12H.24H, EN.Only.Up.2H
**CSF3R**	1.948	2.901	2.014				3.037	3.355			EN.SM.Up.12H.24H, EN.Only.Up.2H
**DGAT2**	4.075	1.305	4.201				1.43	3.658			EN.SM.Up.12H.24H, EN.Only.Up.2H
**DUSP5**	3.697	4.146	2.794				3.704	2.76			EN.SM.Up.12H.24H, EN.Only.Up.2H
**IL1R2**	3.56	3.61	3.521				4.361	2.786			EN.SM.Up.12H.24H, EN.Only.Up.2H
**IL22RA2**	3.01	5.746	4.67				6.344	4.257			EN.SM.Up.12H.24H, EN.Only.Up.2H
**LAMC2**	2.843	4.154	4.71				3.038	2.525			EN.SM.Up.12H.24H, EN.Only.Up.2H
**LOC479340**	3.472	4.899	5.33				4.681	3.433			EN.SM.Up.12H.24H, EN.Only.Up.2H
**LOC484866**	2.667	5.558	3.33				5.162	3.467			EN.SM.Up.12H.24H, EN.Only.Up.2H
**LOC488247**	1.741	2.816	2.217				2.761	5.086			EN.SM.Up.12H.24H, EN.Only.Up.2H
**S100A9**	3.566	4.9	3.168				6.078	3.669			EN.SM.Up.12H.24H, EN.Only.Up.2H
**SERPINB2**	2.507	2.694	2.73				2.656	3.074			EN.SM.Up.12H.24H, EN.Only.Up.2H
**TRIB1**	4.186	2.148	2.519				3.662	2.426			EN.SM.Up.12H.24H, EN.Only.Up.2H
**CDA**		1.107	2.385	1.394			1.443	1.607			EN.SM.Up.12H.24H, EN.Only.Up.7D
**CPLX2**		2.37	2.492	1.401			1.369	2.731			EN.SM.Up.12H.24H, EN.Only.Up.7D
**GGH**		2.123	2.175	1.046			2.283	2.041			EN.SM.Up.12H.24H, EN.Only.Up.7D
**HMGA1**		2.186	2.944	2.073			1.771	2.467			EN.SM.Up.12H.24H, EN.Only.Up.7D
**IL1RN**		3.382	3.95	3.705			3.175	2.282			EN.SM.Up.12H.24H, EN.Only.Up.7D
**LY9**		2.646	2.204	1.631			2.481	2.061			EN.SM.Up.12H.24H, EN.Only.Up.7D
**MARS**		1.586	2.009	1.107			1.761	2.275			EN.SM.Up.12H.24H, EN.Only.Up.7D
**RGS14**		3.347	3.526	1.971			2.916	2.085			EN.SM.Up.12H.24H, EN.Only.Up.7D
**S100P**		3.78	4.311	3.689			4.385	2.677			EN.SM.Up.12H.24H, EN.Only.Up.7D
**SRGN**		3.209	3.081	1.389			5.115	3.511			EN.SM.Up.12H.24H, EN.Only.Up.7D
**ST6GALNAC4**		1.615	1.862	1.425			1.799	1.915			EN.SM.Up.12H.24H, EN.Only.Up.7D
**SYK**		3.501	2.976	1.309			1.14	1.97			EN.SM.Up.12H.24H, EN.Only.Up.7D
**GRB2**		1.253	1.722				1.451	1.397	1.669		EN.SM.Up.12H.24H, SM.Only.Up.7D
**KITLG**		1.878	1.948				1.757	2.026	1.303		EN.SM.Up.12H.24H, SM.Only.Up.7D
**LOC478287**		1.401	2.108				5.432	4.368	1.109		EN.SM.Up.12H.24H, SM.Only.Up.7D
**LOC488474**		1.531	1.81				2.003	1.498	1.761		EN.SM.Up.12H.24H, SM.Only.Up.7D
**LOC491435**		3.558	3.329				3.2	2.683	2.402		EN.SM.Up.12H.24H, SM.Only.Up.7D
**LOC492140**		1.126	1.592				2.951	2.384	2.21		EN.SM.Up.12H.24H, SM.Only.Up.7D
**LPCAT2**		1.282	1.795				2.486	2.254	2.818		EN.SM.Up.12H.24H, SM.Only.Up.7D
**MCPH1**		1.505	2.457				1.958	1.701	1.638		EN.SM.Up.12H.24H, SM.Only.Up.7D
**ARPC2**		1.64	1.675	1.513			2.178		1.435		EN.SM.Up.12H.7D, EN.Only.Up.24H
**BPI**		1.97	3.018	3.208			1.847		2.238		EN.SM.Up.12H.7D, EN.Only.Up.24H
**BTK**		1.891	2.44	2.29			1.286		1.883		EN.SM.Up.12H.7D, EN.Only.Up.24H
**CAPZA1**		1.003	1.182	1.014			1.251		1.034		EN.SM.Up.12H.7D, EN.Only.Up.24H
**CASP10**		1.081	1.129	1.605			1.079		1.444		EN.SM.Up.12H.7D, EN.Only.Up.24H
**CD180**		2.207	2.748	2.145			1.687		1.739		EN.SM.Up.12H.7D, EN.Only.Up.24H
**CLEC7A**		3.202	2.531	1.144			3.499		1.359		EN.SM.Up.12H.7D, EN.Only.Up.24H
**CSK**		1.092	1.249	1.378			2.174		1.054		EN.SM.Up.12H.7D, EN.Only.Up.24H
**CTSH**		1.509	2.153	2.263			1.314		1.368		EN.SM.Up.12H.7D, EN.Only.Up.24H
**EVI2B**		4.083	2.629	2.791			2.645		1.828		EN.SM.Up.12H.7D, EN.Only.Up.24H
**GALNT1**		1.424	1.418	2.28			1.103		2.073		EN.SM.Up.12H.7D, EN.Only.Up.24H
**KDR**		3.406	3.142	3.1			1.721		1.136		EN.SM.Up.12H.7D, EN.Only.Up.24H

**LMNB1**		1.837	2.077	2.027			1.797		1.743		EN.SM.Up.12H.7D, EN.Only.Up.24H
**LOC476222**		1.424	1.735	1.281			1.186		1.273		EN.SM.Up.12H.7D, EN.Only.Up.24H
**LOC610177**		2.035	2.001	1.671			2.481		1.075		EN.SM.Up.12H.7D, EN.Only.Up.24H
**LOC612065**		1.31	3.366	2.574			1.464		1.475		EN.SM.Up.12H.7D, EN.Only.Up.24H
**NID2**		1.621	2.387	2.491			1.029		2.072		EN.SM.Up.12H.7D, EN.Only.Up.24H
**PCNA**		1	1.156	1.124			1.345		1.295		EN.SM.Up.12H.7D, EN.Only.Up.24H
**PDIA6**		1.002	1.224	1.399			1.171		1.277		EN.SM.Up.12H.7D, EN.Only.Up.24H
**PLIN2**		1.806	1.358	1.424			1.219		1.25		EN.SM.Up.12H.7D, EN.Only.Up.24H
**PMEPA1**		1.932	1.004	1.772			1.408		1.777		EN.SM.Up.12H.7D, EN.Only.Up.24H
**PYCR1**		2.107	1.664	1.866			1.294		2.393		EN.SM.Up.12H.7D, EN.Only.Up.24H
**RGS18**		3.333	3.002	2.136			1.841		2.514		EN.SM.Up.12H.7D, EN.Only.Up.24H
**SLC25A5**		1.397	1.311	1.376			1.572		1.252		EN.SM.Up.12H.7D, EN.Only.Up.24H
**SLC2A9**		1.013	1.239	1.05			3.822		2.953		EN.SM.Up.12H.7D, EN.Only.Up.24H
**STK10**		1.704	2.195	1.511			1.8		1.285		EN.SM.Up.12H.7D, EN.Only.Up.24H
**TBXAS1**		3.113	3.027	3.702			3.031		2.097		EN.SM.Up.12H.7D, EN.Only.Up.24H
**TCIRG1**		1.383	1.485	1.681			1.434		1.114		EN.SM.Up.12H.7D, EN.Only.Up.24H
**TLR1**		2.674	3.4	2.189			3.624		2.051		EN.SM.Up.12H.7D, EN.Only.Up.24H
**TLR2**		3.399	3.509	2.028			1.012		1.141		EN.SM.Up.12H.7D, EN.Only.Up.24H
**TMEM173**		2.818	3.268	2.93			1.525		1.553		EN.SM.Up.12H.7D, EN.Only.Up.24H
**ST3GAL5**	1.87	1.557		2.737			1.39		1.711		EN.SM.Up.12H.7D, EN.Only.Up.2H
**FABP5**		3.227		3.387			1.902	2.781	3.148		EN.SM.Up.12H.7D, SM.Only.Down.12H, SM.Only.Up.24H
**ARPC1B**		1.446		1.627			1.195	1.329	1.509		EN.SM.Up.12H.7D, SM.Only.Up.24H
**CAPG**		2.22		3.07			3.162	2.504	4.039		EN.SM.Up.12H.7D, SM.Only.Up.24H
**CGREF1**		1.964		1.58			3.294	2.973	2.628		EN.SM.Up.12H.7D, SM.Only.Up.24H
**CRLF3**		1.576		1.06			1.32	1.538	1.597		EN.SM.Up.12H.7D, SM.Only.Up.24H
**EIF4A1**		1.669		1.104			2.404	2.146	1.164		EN.SM.Up.12H.7D, SM.Only.Up.24H
**FSCN1**		1.65		2.827			1.405	2.257	1.982		EN.SM.Up.12H.7D, SM.Only.Up.24H
**LOC481810**		1.338		2.485			1.099	3.438	1.906		EN.SM.Up.12H.7D, SM.Only.Up.24H
**RIPK2**		1.038		1.24			1.824	1.78	1.064		EN.SM.Up.12H.7D, SM.Only.Up.24H
**SLC5A6**		2.151		1.108			3.809	3.201	1.482		EN.SM.Up.12H.7D, SM.Only.Up.24H
**TIAM2**		1.696		2.058			1.874	2.082	1.225		EN.SM.Up.12H.7D, SM.Only.Up.24H
**TUBB2A**		1.351		1.046			1.325	1.971	1.401		EN.SM.Up.12H.7D, SM.Only.Up.24H
**UPP1**		1.201		1.571			1.708	2.191	1.625		EN.SM.Up.12H.7D, SM.Only.Up.24H
**ADK**			1.355	1.576			2.013	1.65	1.88		EN.SM.Up.24H.7D, SM.Only.Up.12H
**CCDC62**			1.121	1.054			1.749	1.418	1.209		EN.SM.Up.24H.7D, SM.Only.Up.12H
**cOR8T4**			1.065	2.551			1.721	1.613	2.476		EN.SM.Up.24H.7D, SM.Only.Up.12H
**LOC489158**			3.979	2.097			2.282	3.449	1.814		EN.SM.Up.24H.7D, SM.Only.Up.12H
**MPP6**			2.317	1.211			2.221	3.562	2.066		EN.SM.Up.24H.7D, SM.Only.Up.12H
**RRM2**			4.203	5.576			1.87	4.157	5.142		EN.SM.Up.24H.7D, SM.Only.Up.12H
**SERPINB6**			1.792	1.463			1.323	1.273	1.39		EN.SM.Up.24H.7D, SM.Only.Up.12H
**TM4SF18**			1.588	1.825			2.669	2.179	1.535		EN.SM.Up.24H.7D, SM.Only.Up.12H
**KLF6**	2.059	1.075				1.942	1.059	1.624			EN.SM.Up.2H.12H, SM.Only.Up.24H
**CYR61**	1.625	1.293		1.408		2.949			1.03		EN.SM.Up.2H.7D, EN.Only.Up.12H
**MT1F**	3.498			2.549			1.914	3.986	1.654		EN.SM.Up.7D, SM.Only.Up.12H.24H, EN.Only.Up.2H
**LMO7**	1.62	1.766		1.669					1.214	4.388	EN.SM.Up.7D, SM.Only.Up.30D, EN.Only.Up.2H.12H
**WDR3**		2.534	2.005	1.675			1.463	1.251			SM.Only.Up.12H.24H
**ITGA4**			2.465	3.224	2.653				2.719		EN.SM.Up.7D, EN.Only.Up.24H.30D
**DLA-DQA1**			3.44	2.902	3.968				2.558		EN.SM.Up.7D, EN.Only.Up.24H.30D
**AMICA1**		1.967	1.797	2.456			2.28				EN.SM.Up.12H, EN.Only.Up.24H.7D
**CASP4**		2.139	2.753	2.141			2.145				EN.SM.Up.12H, EN.Only.Up.24H.7D
**CD53**		3.83	4.057	3.456			2.55				EN.SM.Up.12H, EN.Only.Up.24H.7D
**CYTH4**		2.15	1.83	1.513			2.028				EN.SM.Up.12H, EN.Only.Up.24H.7D
**DOCK2**		1.371	2.344	2.677			1.617				EN.SM.Up.12H, EN.Only.Up.24H.7D
**E2F3**		1.299	1.751	1.874			1.434				EN.SM.Up.12H, EN.Only.Up.24H.7D
**FERMT3**		1.621	1.7	1.764			1.339				EN.SM.Up.12H, EN.Only.Up.24H.7D
**FMNL1**		2.054	1.738	1.329			1.93				EN.SM.Up.12H, EN.Only.Up.24H.7D
**GBP1**		2.552	2.692	5.088			1.406				EN.SM.Up.12H, EN.Only.Up.24H.7D
**GIMAP4**		3.051	2.55	2.626			1.665				EN.SM.Up.12H, EN.Only.Up.24H.7D
**GIMAP5**		2.167	3.27	2.738			1.876				EN.SM.Up.12H, EN.Only.Up.24H.7D
**GJA1**		2.246	2.03	1.969			1.223				EN.SM.Up.12H, EN.Only.Up.24H.7D
**GPR137B**		2.463	2.402	1.018			2.869				EN.SM.Up.12H, EN.Only.Up.24H.7D
**HPSE**		3.076	3.501	2.455			2.58				EN.SM.Up.12H, EN.Only.Up.24H.7D
**HYOU1**		1.518	1.455	1.561			1.113				EN.SM.Up.12H, EN.Only.Up.24H.7D
**IL18RAP**		4.722	4.128	1.627			5.455				EN.SM.Up.12H, EN.Only.Up.24H.7D
**IL2RG**		2.021	1.953	1.61			1.529				EN.SM.Up.12H, EN.Only.Up.24H.7D
**ITGB2**		3.134	1.826	2.624			2.952				EN.SM.Up.12H, EN.Only.Up.24H.7D
**LILRB2**		3.569	3.362	1.864			3.604				EN.SM.Up.12H, EN.Only.Up.24H.7D
**LOC479458**		3.107	1.676	2.784			1.651				EN.SM.Up.12H, EN.Only.Up.24H.7D
**LOC480473**		2.966	3.177	2.291			3.236				EN.SM.Up.12H, EN.Only.Up.24H.7D
**LOC486905**		2.595	4.881	3.322			2.022				EN.SM.Up.12H, EN.Only.Up.24H.7D
**LOC611446**		1.515	2.152	1.249			1.377				EN.SM.Up.12H, EN.Only.Up.24H.7D
**LY86**		2.076	1.694	2.57			1.516				EN.SM.Up.12H, EN.Only.Up.24H.7D
**MYL5**		1.617	1.761	1.252			1.012				EN.SM.Up.12H, EN.Only.Up.24H.7D
**NME2**		2.272	2.083	1.71			2.806				EN.SM.Up.12H, EN.Only.Up.24H.7D
**OLR1**		2.082	1.686	1.092			2.424				EN.SM.Up.12H, EN.Only.Up.24H.7D
**PIK3AP1**		2.667	3.015	2.6			2.084				EN.SM.Up.12H, EN.Only.Up.24H.7D
**PIK3CG**		3.024	2.013	2.021			2.965				EN.SM.Up.12H, EN.Only.Up.24H.7D
**PILRA**		2.154	1.753	1.374			2.189				EN.SM.Up.12H, EN.Only.Up.24H.7D
**PLCB2**		2.1	2.996	3.08			2.133				EN.SM.Up.12H, EN.Only.Up.24H.7D
**PREX1**		2.3	1.953	1.074			2.005				EN.SM.Up.12H, EN.Only.Up.24H.7D
**RASAL3**		1.778	1.175	1.127			2.162				EN.SM.Up.12H, EN.Only.Up.24H.7D
**SAMSN1**		3.874	2.995	2.132			4.164				EN.SM.Up.12H, EN.Only.Up.24H.7D
**SIRPA**		1.193	2.107	2.429			1.427				EN.SM.Up.12H, EN.Only.Up.24H.7D
**SLC2A6**		2.267	1.803	1.321			1.907				EN.SM.Up.12H, EN.Only.Up.24H.7D
**ST8SIA4**		2.836	2.62	1.801			2.885				EN.SM.Up.12H, EN.Only.Up.24H.7D
**SULF2**		3.496	1.893	4.422			1.678				EN.SM.Up.12H, EN.Only.Up.24H.7D
**TMEM154**		2.166	2.868	1.782			2.251				EN.SM.Up.12H, EN.Only.Up.24H.7D
**VAV1**		1.917	1.68	1.78			1.81				EN.SM.Up.12H, EN.Only.Up.24H.7D
**VMA21**		1.09	1.27	1.287			1.036				EN.SM.Up.12H, EN.Only.Up.24H.7D
**WDR37**		2.534	2.005	1.675			2.479				EN.SM.Up.12H, EN.Only.Up.24H.7D
**1-Mar**	3.31	2.81	3.843				3.095				EN.SM.Up.12H, EN.Only.Up.2H.24H
**ARF6**	1.441	1.93	2.076				1.801				EN.SM.Up.12H, EN.Only.Up.2H.24H
**C4BPB**	3.353	1.874	2.276				1.963				EN.SM.Up.12H, EN.Only.Up.2H.24H
**CEACAM21**	3.036	4.562	3.296				4.838				EN.SM.Up.12H, EN.Only.Up.2H.24H
**CSF2RA**	2.885	5.131	4.693				4.975				EN.SM.Up.12H, EN.Only.Up.2H.24H
**EMP1**	2.058	1.298	1.504				1.161				EN.SM.Up.12H, EN.Only.Up.2H.24H
**ERRFI1**	1.769	1.895	1.624				2.103				EN.SM.Up.12H, EN.Only.Up.2H.24H
**HIGD1A**	2.233	1.143	1.133				1.33				EN.SM.Up.12H, EN.Only.Up.2H.24H
**IL1B**	2.061	3.126	3.131				2.869				EN.SM.Up.12H, EN.Only.Up.2H.24H
**ITGAM**	3.103	3.262	4.37				3.839				EN.SM.Up.12H, EN.Only.Up.2H.24H
**LOC608975**	1.855	3.912	3.529				3.362				EN.SM.Up.12H, EN.Only.Up.2H.24H
**LOC611576**	3.611	3.006	3.72				3.125				EN.SM.Up.12H, EN.Only.Up.2H.24H
**MCTP1**	2.375	1.98	2.539				1.995				EN.SM.Up.12H, EN.Only.Up.2H.24H
**MCTP2**	2.789	4.936	3.933				1.442				EN.SM.Up.12H, EN.Only.Up.2H.24H
**MXD1**	2.392	2.62	2.444				2.664				EN.SM.Up.12H, EN.Only.Up.2H.24H
**NFKBID**	4.077	2.044	2.37				2.01				EN.SM.Up.12H, EN.Only.Up.2H.24H
**PFKFB3**	2.21	2.173	1.822				1.133				EN.SM.Up.12H, EN.Only.Up.2H.24H
**PLBD1**	2.998	2.075	4.2				4.07				EN.SM.Up.12H, EN.Only.Up.2H.24H
**PPBP**	4.573	2.284	3.23				1.653				EN.SM.Up.12H, EN.Only.Up.2H.24H
**SLC2A3**	1.992	1.371	2.29				1.325				EN.SM.Up.12H, EN.Only.Up.2H.24H
**TIPARP**	3.923	1.323	1.478				2.233				EN.SM.Up.12H, EN.Only.Up.2H.24H
**CCL3**	1.89	1.308		2.29			1.782				EN.SM.Up.12H, EN.Only.Up.2H.7D
**IER3**	2.585	1.15		1.07			1.52				EN.SM.Up.12H, EN.Only.Up.2H.7D
**ETS1**	2.883	1.208					1.968	2.154			EN.SM.Up.12H, SM.Only.Up.24H, EN.Only.Up.2H
**HSPH1**	1.234	1.764					2.01	1.875			EN.SM.Up.12H, SM.Only.Up.24H, EN.Only.Up.2H
**TNFAIP6**	2.186	1.729					2.474	2.458			EN.SM.Up.12H, SM.Only.Up.24H, EN.Only.Up.2H
**ZFAND2A**	2.468	1.598					1.219	2.58			EN.SM.Up.12H, SM.Only.Up.24H, EN.Only.Up.2H
**CHD7**		1.527		1.526			1.093	2.011			EN.SM.Up.12H, SM.Only.Up.24H, EN.Only.Up.7D
**LOC489252**		1.275		1.075			1.68	1.626			EN.SM.Up.12H, SM.Only.Up.24H, EN.Only.Up.7D
**PRELID1**		1.222		1.055			1.691	1.464			EN.SM.Up.12H, SM.Only.Up.24H, EN.Only.Up.7D
**PROCR**		1.334		1.461			1.786	2.851			EN.SM.Up.12H, SM.Only.Up.24H, EN.Only.Up.7D
**CDC7**		1.048					1.048	2.516	1.314		EN.SM.Up.12H, SM.Only.Up.24H.7D
**DDX21**		1.21					2.388	2.189	1.089		EN.SM.Up.12H, SM.Only.Up.24H.7D

**KIF2A**		1.478					1.794	1.594	1.052		EN.SM.Up.12H, SM.Only.Up.24H.7D
**LOC486736**		1.104					1.209	3.401	1.095		EN.SM.Up.12H, SM.Only.Up.24H.7D
**RPL22L1**		1.294					1.434	1.939	1.031		EN.SM.Up.12H, SM.Only.Up.24H.7D
**SRPX2**		1.846					2.373	1.567	2.298		EN.SM.Up.12H, SM.Only.Up.24H.7D
**TGFB3**		1.089					1.049	2.087	1.038		EN.SM.Up.12H, SM.Only.Up.24H.7D
**GIT2**		1.602	2.014			2.862	1.047				EN.SM.Up.12H, SM.Only.Up.2H, EN.Only.Up.24H
**CREM**		1.592	3.072				2.024		2.212		EN.SM.Up.12H, SM.Only.Up.7D, EN.Only.Up.24H
**HELLS**		2.204	3.977				1.248		2.681		EN.SM.Up.12H, SM.Only.Up.7D, EN.Only.Up.24H
**MAPK6**		1.102	1.022				1.01		1.294		EN.SM.Up.12H, SM.Only.Up.7D, EN.Only.Up.24H
**PLK3**		1.578	1.015				1.512		1.747		EN.SM.Up.12H, SM.Only.Up.7D, EN.Only.Up.24H
**RPS6KC1**		1.238	1.453				1.017		1.081		EN.SM.Up.12H, SM.Only.Up.7D, EN.Only.Up.24H
**SAE1**		1.368	1.575				1.123		1.096		EN.SM.Up.12H, SM.Only.Up.7D, EN.Only.Up.24H
**SLC31A2**		1.33	1.842				1.832		1.327		EN.SM.Up.12H, SM.Only.Up.7D, EN.Only.Up.24H
**TFRC**		2.764	1.782				2.942		1.154		EN.SM.Up.12H, SM.Only.Up.7D, EN.Only.Up.24H
**UBA6**		1.431	1.109				1.078		1.271		EN.SM.Up.12H, SM.Only.Up.7D, EN.Only.Up.24H
**ACSL4**	1.328	1.944					2.428		1.663		EN.SM.Up.12H, SM.Only.Up.7D, EN.Only.Up.2H
**DNAJB1**	2.087	1.933					1.598		1.128		EN.SM.Up.12H, SM.Only.Up.7D, EN.Only.Up.2H
**MED28**	2.08	1.524					1.766		1.435		EN.SM.Up.12H, SM.Only.Up.7D, EN.Only.Up.2H
**UGDH**	2.875	1.462					2.13		1.058		EN.SM.Up.12H, SM.Only.Up.7D, EN.Only.Up.2H
**ADORA2A**		2.681	1.231				3.008	2.402			EN.SM.Up.12H.24H
**CD68**		3.977	3.569				3.975	2.966			EN.SM.Up.12H.24H
**EIF2S1**		1.262	1.33				1.603	1.517			EN.SM.Up.12H.24H
**ESM1**		3.994	4.886				4.599	4.251			EN.SM.Up.12H.24H
**ITGB7**		1.434	1.765				1.519	1.759			EN.SM.Up.12H.24H
**LOC611593**		1.049	1.547				1.099	1.646			EN.SM.Up.12H.24H
**MANF**		1.33	2.009				1.503	1.94			EN.SM.Up.12H.24H
**NAMPT**		2.03	2.795				2.759	2.181			EN.SM.Up.12H.24H
**NDUFAF2**		1.43	1.393				2.211	1.39			EN.SM.Up.12H.24H
**NDUFAF4**		1.651	1.397				1.601	1.758			EN.SM.Up.12H.24H
**PDE4B**		1.207	1.082				1.439	1.398			EN.SM.Up.12H.24H
**PPID**		1.452	1.34				1.632	1.692			EN.SM.Up.12H.24H
**PSMD14**		1.417	1.43				1.174	1.379			EN.SM.Up.12H.24H
**RRAS2**		2.005	1.415				2.461	1.75			EN.SM.Up.12H.24H
**SDF2L1**		1.585	1.635				1.1	1.851			EN.SM.Up.12H.24H
**SIGLEC12**		2.777	2.333				3.244	2.67			EN.SM.Up.12H.24H
**SLC7A5**		2.013	1.158				1.667	1.519			EN.SM.Up.12H.24H
**SNX10**		2.444	2.972				3.05	3.613			EN.SM.Up.12H.24H
**SYPL1**		2.432	1.882				3.181	2.064			EN.SM.Up.12H.24H
**TLR4**		1.238	1.538				2.132	2.019			EN.SM.Up.12H.24H
**TMEM80**		2.954	2.834				4.112	2.718			EN.SM.Up.12H.24H
**TNIP3**		4.111	3.558				4.918	3.252			EN.SM.Up.12H.24H
**WDR12**		1.393	1.369				1.86	1.741			EN.SM.Up.12H.24H
**WDR43**		1.213	1.193				1.331	1.482			EN.SM.Up.12H.24H
**ZCCHC6**		1.108	1.048				1.518	1.191			EN.SM.Up.12H.24H
**CASP8**		1.917		1.646			2.651		1.376		EN.SM.Up.12H.7D
**FKBP11**		1.622		2.006			1.982		2.399		EN.SM.Up.12H.7D
**HN1**		1.314		2.922			1.305		1.78		EN.SM.Up.12H.7D
**LOC480469**		1.269		1.81			1.377		1.731		EN.SM.Up.12H.7D
**MTHFD1L**		1.849		1.346			1.854		1.404		EN.SM.Up.12H.7D
**NANS**		1.693		1.608			2.242		1.543		EN.SM.Up.12H.7D
**NCAPG2**		1.266		2.474			1.518		2.795		EN.SM.Up.12H.7D
**P4HA2**		1.171		1.009			1.086		1.024		EN.SM.Up.12H.7D
**PCBD2**		1.026		1.111			1.283		1.138		EN.SM.Up.12H.7D
**PLAT**		2.641		1.209			1.315		1.658		EN.SM.Up.12H.7D
**PPM1H**		1.529		1.718			1.447		1.311		EN.SM.Up.12H.7D
**RTN3**		1.111		1.025			1.382		1.157		EN.SM.Up.12H.7D
**SAA1**		2.38		5.96			4.774		5.018		EN.SM.Up.12H.7D
**TPM3**		1.617		1.296			2.128		1.206		EN.SM.Up.12H.7D
**TPX2**		1.316		3.011			1.16		3.024		EN.SM.Up.12H.7D
**TUBA4A**		2.28		1.678			1.722		1.596		EN.SM.Up.12H.7D
**CCNYL1**			1.382	1.083			1.761	1.874			EN.SM.Up.24H, SM.Only.Up.12H, EN.Only.Up.7D
**MRPL35**			2.285	1.247			1.399	2.526			EN.SM.Up.24H, SM.Only.Up.12H, EN.Only.Up.7D
**STXBP2**			1.536	1.469			1.295	1.369			EN.SM.Up.24H, SM.Only.Up.12H, EN.Only.Up.7D
**DDX60**			2.782				1.811	2.402	3.687		EN.SM.Up.24H, SM.Only.Up.12H.7D
**HTR2A**			1.865				2.242	2.309	1.063		EN.SM.Up.24H, SM.Only.Up.12H.7D
**ORC1L**			3.975	1.722				1.982	2.573		EN.SM.Up.24H.7D
**CRYAB**	2.249	2.589				2.18	2.301				EN.SM.Up.2H.12H
**DUSP10**	2.676	2.326				2.997	1.838				EN.SM.Up.2H.12H
**IFRD1**	2.152	1.236				2.063	1.011				EN.SM.Up.2H.12H
**CTSC**		1.1	1.913	3.666					2.992		EN.SM.Up.7D, EN.Only.Up.12H.24H
**PCK2**		1.297	1.804	1.815					1.551		EN.SM.Up.7D, EN.Only.Up.12H.24H
**PRKCD**		1.742	2.412	2.022					2.488		EN.SM.Up.7D, EN.Only.Up.12H.24H
**RGS10**		1.237	1.631	3.296					2.035		EN.SM.Up.7D, EN.Only.Up.12H.24H
**RNASEH2B**		1.459	1.53	1.686					1.334		EN.SM.Up.7D, EN.Only.Up.12H.24H
**TRIM22**		1.565	2.96	4.049					3.291		EN.SM.Up.7D, EN.Only.Up.12H.24H
**AMZ2**			1.186	1.429			2.046		1.573		EN.SM.Up.7D, SM.Only.Up.12H, EN.Only.Up.24H
**ANPEP**			1.008	1.174			1.886		3.341		EN.SM.Up.7D, SM.Only.Up.12H, EN.Only.Up.24H
**CARHSP1**			1.773	2.991			1.55		1.758		EN.SM.Up.7D, SM.Only.Up.12H, EN.Only.Up.24H
**CD14**			2.565	3.231			2.508		2.611		EN.SM.Up.7D, SM.Only.Up.12H, EN.Only.Up.24H
**CENPK**			2.264	2.234			1.69		2.841		EN.SM.Up.7D, SM.Only.Up.12H, EN.Only.Up.24H
**COMMD8**			2.31	1.299			1.258		1.027		EN.SM.Up.7D, SM.Only.Up.12H, EN.Only.Up.24H
**CTSB**			1.124	1.37			1.022		1.191		EN.SM.Up.7D, SM.Only.Up.12H, EN.Only.Up.24H
**KCNE3**			2.113	1.775			2.976		1.358		EN.SM.Up.7D, SM.Only.Up.12H, EN.Only.Up.24H
**MCM4**			2.019	1.384			1.074		1.534		EN.SM.Up.7D, SM.Only.Up.12H, EN.Only.Up.24H
**SMPDL3A**			2.052	4.036			1.634		2.579		EN.SM.Up.7D, SM.Only.Up.12H, EN.Only.Up.24H
**WHSC1**			2.563	1.516			1.141		1.493		EN.SM.Up.7D, SM.Only.Up.12H, EN.Only.Up.24H
**CHSY1**				1.149			1.544	1.836	1.256		EN.SM.Up.7D, SM.Only.Up.12H.24H
**LOC475852**				1.097			1.21	1.568	1.483		EN.SM.Up.7D, SM.Only.Up.12H.24H
**MELK**				1.685			1.217	2.635	3.84		EN.SM.Up.7D, SM.Only.Up.12H.24H
**MT2A**				2.058			1.319	1.705	1.363		EN.SM.Up.7D, SM.Only.Up.12H.24H
**RAI14**				1.053			1.296	2.079	1.642		EN.SM.Up.7D, SM.Only.Up.12H.24H
**YARS**				1.189			1.259	1.853	1.065		EN.SM.Up.7D, SM.Only.Up.12H.24H
**KIF15**		1.265		1.517					1.358	1.969	EN.SM.Up.7D, SM.Only.Up.30D, EN.Only.Up.12H
**SLC27A6**	2.363		3.89	2.327			1.625				SM.Only.Up.12H, EN.Only.Up.2H.24H.7D
**NFKBIZ**	3.05						3.215	2.288	2.246		SM.Only.Up.12H.24H.7D, EN.Only.Up.2H
**SLC30A1**	1.475		1.104				1.004		1.397		SM.Only.Up.12H.7D, EN.Only.Up.2H.24H
**COL5A1**				1.723	2.048				1.563		EN.SM.Up.7D, EN.Only.Up.30D
**COL4A1**				1.447	2.167				1.38		EN.SM.Up.7D, EN.Only.Up.30D
**DNAJB13**			3.978		2.652		1.582				SM.Only.Up.12H, EN.Only.Up.24H.30D
**CDH2**				1.189	3.094		1.071				SM.Only.Up.12H, EN.Only.Up.7D.30D
**NDC80**				1.254	4.234				1.264		EN.SM.Up.7D, EN.Only.Up.30D
**DNAH11**			1.682	2.01					1.637		EN.Only.Down.7D, EN.SM.Up.7D, EN.Only.Up.24H
**ARF3**		1.17	1.525	1.212							EN.Only.Up.12H.24H.7D
**CD37**		1.214	1.113	2.114							EN.Only.Up.12H.24H.7D
**CSF1R**		1.616	1.433	1.713							EN.Only.Up.12H.24H.7D
**DENND1C**		1.088	1.732	1.256							EN.Only.Up.12H.24H.7D
**IL31RA**		1.044	1.163	1.573							EN.Only.Up.12H.24H.7D
**LOC480726**		1.116	1.014	1.01							EN.Only.Up.12H.24H.7D
**MRPL1**			1.274				1.044	1.558			EN.Only.Up.24H
**CSF3**	1.586						3.037	3.355			EN.Only.Up.2H
**ADORA3**		2.374	2.561				3.118				EN.SM.Up.12H, EN.Only.Up.24H
**AGPAT9**		1.494	2.141				1.646				EN.SM.Up.12H, EN.Only.Up.24H
**ANKRD22**		3.496	2.986				3.895				EN.SM.Up.12H, EN.Only.Up.24H
**AOAH**		3.518	3.906				3.513				EN.SM.Up.12H, EN.Only.Up.24H
**ARAP1**		1.36	1.482				1.125				EN.SM.Up.12H, EN.Only.Up.24H
**BBS5**		1.287	1.077				1.088				EN.SM.Up.12H, EN.Only.Up.24H
**CACNB4**		1.391	1.907				1.731				EN.SM.Up.12H, EN.Only.Up.24H
**CCR3**		1.681	1.139				2.185				EN.SM.Up.12H, EN.Only.Up.24H
**CCRN4L**		3.23	1.943				2.417				EN.SM.Up.12H, EN.Only.Up.24H
**CCT4**		1.09	1.278				1.03				EN.SM.Up.12H, EN.Only.Up.24H
**CCT5**		1.457	1.408				1.565				EN.SM.Up.12H, EN.Only.Up.24H
**CCT8**		1.338	1.167				1.448				EN.SM.Up.12H, EN.Only.Up.24H
**CLEC4D**		2.028	2.201				2.384				EN.SM.Up.12H, EN.Only.Up.24H

**CSF2RB**		1.533	1.378				1.767				EN.SM.Up.12H, EN.Only.Up.24H
**CYCS**		1.643	1.354				1.803				EN.SM.Up.12H, EN.Only.Up.24H
**DPYD**		2.614	1.773				1.576				EN.SM.Up.12H, EN.Only.Up.24H
**EIF5**		1.103	1.358				1.341				EN.SM.Up.12H, EN.Only.Up.24H
**EMR3**		3.879	2.902				1.21				EN.SM.Up.12H, EN.Only.Up.24H
**GLIPR2**		1.691	1.999				1.484				EN.SM.Up.12H, EN.Only.Up.24H
**HCK**		2.996	1.901				2.867				EN.SM.Up.12H, EN.Only.Up.24H
**HK3**		2.551	2.276				1.883				EN.SM.Up.12H, EN.Only.Up.24H
**HSD17B12**		1.749	1.247				1.273				EN.SM.Up.12H, EN.Only.Up.24H
**HSPA9**		1.058	1.003				1.075				EN.SM.Up.12H, EN.Only.Up.24H
**IGSF2**		2.453	2.474				2.242				EN.SM.Up.12H, EN.Only.Up.24H
**IL1A**		3.245	3.377				3.442				EN.SM.Up.12H, EN.Only.Up.24H
**IL1RAP**		2.041	1.868				1.538				EN.SM.Up.12H, EN.Only.Up.24H
**IMP4**		1.284	1.087				1.937				EN.SM.Up.12H, EN.Only.Up.24H
**ITGAL**		2.52	2.725				1.599				EN.SM.Up.12H, EN.Only.Up.24H
**JRKL**		1.344	1.832				1.104				EN.SM.Up.12H, EN.Only.Up.24H
**KCNJ15**		2.912	1.641				3.268				EN.SM.Up.12H, EN.Only.Up.24H
**LAT2**		1.704	1.624				1.284				EN.SM.Up.12H, EN.Only.Up.24H
**LILRB3**		1.509	1.647				1.377				EN.SM.Up.12H, EN.Only.Up.24H
**LOC477650**		1.18	1.257				1.328				EN.SM.Up.12H, EN.Only.Up.24H
**LOC479292**		1.573	1.946				2.049				EN.SM.Up.12H, EN.Only.Up.24H
**LOC485452**		2.08	1.553				1.301				EN.SM.Up.12H, EN.Only.Up.24H
**LOC491201**		1.339	1.164				1.104				EN.SM.Up.12H, EN.Only.Up.24H
**LOC607469**		1.131	1.412				1.005				EN.SM.Up.12H, EN.Only.Up.24H
**LOC607948**		1.151	1.821				1.081				EN.SM.Up.12H, EN.Only.Up.24H
**LOC612137**		1.5	1.371				1.742				EN.SM.Up.12H, EN.Only.Up.24H
**LRMP**		3.094	3.394				2.836				EN.SM.Up.12H, EN.Only.Up.24H
**LRRC18**		2.175	1.486				1.502				EN.SM.Up.12H, EN.Only.Up.24H
**LYST**		1.393	1.862				1.001				EN.SM.Up.12H, EN.Only.Up.24H
**MAPRE1**		1.037	1.065				1.124				EN.SM.Up.12H, EN.Only.Up.24H
**MDM2**		1.112	1.163				1.072				EN.SM.Up.12H, EN.Only.Up.24H
**MOGAT1**		4.398	2.831				3.913				EN.SM.Up.12H, EN.Only.Up.24H
**NARG1**		1.064	1.306				1.296				EN.SM.Up.12H, EN.Only.Up.24H
**NARS**		1.379	1.436				1.295				EN.SM.Up.12H, EN.Only.Up.24H
**NAT10**		1.3	1.189				1.24				EN.SM.Up.12H, EN.Only.Up.24H
**NCL**		1.046	1.067				1.233				EN.SM.Up.12H, EN.Only.Up.24H
**NHEDC1**		2.716	1.522				1.856				EN.SM.Up.12H, EN.Only.Up.24H
**NLRP3**		1.875	1.638				1.49				EN.SM.Up.12H, EN.Only.Up.24H
**NUDCD1**		1.228	1.179				1.024				EN.SM.Up.12H, EN.Only.Up.24H
**ODC1**		1.408	1.448				1.461				EN.SM.Up.12H, EN.Only.Up.24H
**PADI4**		2.755	1.747				2.769				EN.SM.Up.12H, EN.Only.Up.24H
**PAICS**		1.716	1.7				1.214				EN.SM.Up.12H, EN.Only.Up.24H
**PPIH**		1.26	1.885				1.489				EN.SM.Up.12H, EN.Only.Up.24H
**PSMB2**		1.031	1.066				1.083				EN.SM.Up.12H, EN.Only.Up.24H
**PTGER2**		2.04	3.66				1.977				EN.SM.Up.12H, EN.Only.Up.24H
**PTPN6**		2.049	1.514				1.702				EN.SM.Up.12H, EN.Only.Up.24H
**RASSF2**		1.734	1.268				1.218				EN.SM.Up.12H, EN.Only.Up.24H
**RNF24**		1.766	1.772				1.544				EN.SM.Up.12H, EN.Only.Up.24H
**SAR1B**		1.428	1.12				1.429				EN.SM.Up.12H, EN.Only.Up.24H
**SEC61G**		1.023	1.305				1.168				EN.SM.Up.12H, EN.Only.Up.24H
**SLC27A2**		3.835	1.973				3.898				EN.SM.Up.12H, EN.Only.Up.24H
**SLC41A1**		1.06	1.053				1.056				EN.SM.Up.12H, EN.Only.Up.24H
**SLC4A7**		2.075	1.764				1.136				EN.SM.Up.12H, EN.Only.Up.24H
**SLMO2**		1.171	1.162				1.35				EN.SM.Up.12H, EN.Only.Up.24H
**TBC1D23**		1.403	1.143				1.102				EN.SM.Up.12H, EN.Only.Up.24H
**TGFBI**		3.008	3.668				1.341				EN.SM.Up.12H, EN.Only.Up.24H
**TMC4**		1.418	1.389				1.539				EN.SM.Up.12H, EN.Only.Up.24H
**TMEM183A**		1.319	1.085				1.578				EN.SM.Up.12H, EN.Only.Up.24H
**TNIP1**		1.121	1.284				1.611				EN.SM.Up.12H, EN.Only.Up.24H
**TRAF3IP3**		2.595	1.397				1.149				EN.SM.Up.12H, EN.Only.Up.24H
**TSPAN8**		2.041	3.443				1.585				EN.SM.Up.12H, EN.Only.Up.24H
**UNC45B**		1.638	1.867				2.057				EN.SM.Up.12H, EN.Only.Up.24H
**HBEGF**	4.518	3					3.248				EN.SM.Up.12H, EN.Only.Up.2H
**HOMER1**	2.35	1.165					1.993				EN.SM.Up.12H, EN.Only.Up.2H
**IL18R1**	2.846	4.497					4.356				EN.SM.Up.12H, EN.Only.Up.2H
**LOC484510**	2.468	1.315					1.301				EN.SM.Up.12H, EN.Only.Up.2H
**LOC607623**	2.466	1.753					1.435				EN.SM.Up.12H, EN.Only.Up.2H
**LOC610734**	2.229	1.783					1.62				EN.SM.Up.12H, EN.Only.Up.2H
**SAT1**	1.353	1.357					1.628				EN.SM.Up.12H, EN.Only.Up.2H
**SOCS3**	1.702	1.618					1.405				EN.SM.Up.12H, EN.Only.Up.2H
**THBS1**	2.053	1.054					1.507				EN.SM.Up.12H, EN.Only.Up.2H
**ATOX1**		1.143		1.941			1.02				EN.SM.Up.12H, EN.Only.Up.7D
**CA2**		1.25		2.979			4.826				EN.SM.Up.12H, EN.Only.Up.7D
**CXCL16**		2.287		2.73			3.264				EN.SM.Up.12H, EN.Only.Up.7D
**DAPK1**		2.283		1.611			1.63				EN.SM.Up.12H, EN.Only.Up.7D
**EMR2**		4.599		1.804			5.134				EN.SM.Up.12H, EN.Only.Up.7D
**GPSM3**		3.162		1.678			3.065				EN.SM.Up.12H, EN.Only.Up.7D
**IL7R**		1.679		4.097			1.267				EN.SM.Up.12H, EN.Only.Up.7D
**LOC474756**		1.101		1.324			1.181				EN.SM.Up.12H, EN.Only.Up.7D
**LOC474811**		1.84		1.807			1.534				EN.SM.Up.12H, EN.Only.Up.7D
**LOC608996**		1.577		1.309			1.059				EN.SM.Up.12H, EN.Only.Up.7D
**SELP**		1.533		1.102			1.689				EN.SM.Up.12H, EN.Only.Up.7D
**SLC15A1**		4.275		4.403			4.567				EN.SM.Up.12H, EN.Only.Up.7D
**UBFD1**		1.613		1.747			1.262				EN.SM.Up.12H, EN.Only.Up.7D
**PDE4D**		1.545					1.433	2.019			EN.SM.Up.12H, EN.SM.Down.12H, SM.Only.Up.24H
**ABCF1**		1.055					1.07	1.388			EN.SM.Up.12H, SM.Only.Up.24H
**ADAMTS4**		1.733					2.659	1.7			EN.SM.Up.12H, SM.Only.Up.24H
**DCTPP1**		1.171					1.613	1.866			EN.SM.Up.12H, SM.Only.Up.24H
**DDX47**		1.036					1.175	1.387			EN.SM.Up.12H, SM.Only.Up.24H
**DHX37**		1.26					1.707	1.556			EN.SM.Up.12H, SM.Only.Up.24H
**DKC1**		1.828					1.436	1.554			EN.SM.Up.12H, SM.Only.Up.24H
**DPH3**		1.285					1.026	1.486			EN.SM.Up.12H, SM.Only.Up.24H
**EIF3B**		1.143					1.3	1.908			EN.SM.Up.12H, SM.Only.Up.24H
**EIF4E**		1.393					1.116	1.313			EN.SM.Up.12H, SM.Only.Up.24H
**EIF5A**		1.636					1.445	1.951			EN.SM.Up.12H, SM.Only.Up.24H
**FFAR2**		2.801					2.771	1.858			EN.SM.Up.12H, SM.Only.Up.24H
**FGR**		1.285					2.798	1.89			EN.SM.Up.12H, SM.Only.Up.24H
**FTSJ3**		1.658					2.136	2.354			EN.SM.Up.12H, SM.Only.Up.24H
**GPATCH4**		1.405					1.909	1.619			EN.SM.Up.12H, SM.Only.Up.24H
**GRAMD1A**		1.453					1.359	1.891			EN.SM.Up.12H, SM.Only.Up.24H
**HSPD1**		1.332					1.23	1.634			EN.SM.Up.12H, SM.Only.Up.24H
**IL18BP**		3.551					4.532	2.197			EN.SM.Up.12H, SM.Only.Up.24H
**INHBA**		2.425					3.342	1.751			EN.SM.Up.12H, SM.Only.Up.24H
**LOC476306**		3.649					3.438	1.701			EN.SM.Up.12H, SM.Only.Up.24H
**LOC477652**		1.571					1.271	2.133			EN.SM.Up.12H, SM.Only.Up.24H
**LOC606831**		1.993					2.351	2.149			EN.SM.Up.12H, SM.Only.Up.24H
**LOC607966**		1.727					3	3.371			EN.SM.Up.12H, SM.Only.Up.24H
**MRPL15**		1.139					1.044	1.558			EN.SM.Up.12H, SM.Only.Up.24H
**NIPAL4**		1.649					1.66	1.715			EN.SM.Up.12H, SM.Only.Up.24H
**NOP2**		1.364					1.544	2.379			EN.SM.Up.12H, SM.Only.Up.24H
**NOP58**		1.005					1.497	1.601			EN.SM.Up.12H, SM.Only.Up.24H
**PDCD2L**		1.505					1.52	1.582			EN.SM.Up.12H, SM.Only.Up.24H
**PI3**		2.744					4.031	3.952			EN.SM.Up.12H, SM.Only.Up.24H
**PNO1**		1.161					1.613	1.443			EN.SM.Up.12H, SM.Only.Up.24H
**POLR1C**		1.064					1.435	1.906			EN.SM.Up.12H, SM.Only.Up.24H
**PTGS2**		3.577					7.833	4.946			EN.SM.Up.12H, SM.Only.Up.24H
**PTPN22**		2.233					2.232	1.703			EN.SM.Up.12H, SM.Only.Up.24H
**RASSF8**		1.215					1.076	1.926			EN.SM.Up.12H, SM.Only.Up.24H
**RBM28**		1.667					1.45	2.057			EN.SM.Up.12H, SM.Only.Up.24H
**SACS**		1.467					1.073	1.714			EN.SM.Up.12H, SM.Only.Up.24H
**SETDB2**		2.482					2.393	3.264			EN.SM.Up.12H, SM.Only.Up.24H
**SLC39A14**		1.909					2.729	2.416			EN.SM.Up.12H, SM.Only.Up.24H
**STOML2**		1.346					1.426	1.565			EN.SM.Up.12H, SM.Only.Up.24H

TIMM10		1.218					1.436	1.41			EN.SM.Up.12H, SM.Only.Up.24H
TMEM165		1.946					2.257	1.952			EN.SM.Up.12H, SM.Only.Up.24H
TRMT6		1.734					1.048	1.527			EN.SM.Up.12H, SM.Only.Up.24H
UGP2		1.917					1.741	2.335			EN.SM.Up.12H, SM.Only.Up.24H
UTP6		1.006					1.319	1.45			EN.SM.Up.12H, SM.Only.Up.24H
VARS		1.195					1.526	1.372			EN.SM.Up.12H, SM.Only.Up.24H
WDR77		2.031					1.461	1.827			EN.SM.Up.12H, SM.Only.Up.24H
ZNF593		1.662					2.148	2.266			EN.SM.Up.12H, SM.Only.Up.24H
BAG1		1.158					1.121		1.092		EN.SM.Up.12H, SM.Only.Up.7D
CYTIP		1.527					4.752		1.564		EN.SM.Up.12H, SM.Only.Up.7D
EEF1E1		1.204					1.155		1.329		EN.SM.Up.12H, SM.Only.Up.7D
EIF2B1		1.045					1.106		1.17		EN.SM.Up.12H, SM.Only.Up.7D
GPRC5A		2.139					3.234		1.168		EN.SM.Up.12H, SM.Only.Up.7D
LOC474923		1.316					1.157		1.127		EN.SM.Up.12H, SM.Only.Up.7D
LOC610769		1.127					1.106		1.574		EN.SM.Up.12H, SM.Only.Up.7D
MRPS10		1.819					1.045		1.513		EN.SM.Up.12H, SM.Only.Up.7D
MYD88		1.079					1.47		1.162		EN.SM.Up.12H, SM.Only.Up.7D
PLK2		1.524					1.465		1.517		EN.SM.Up.12H, SM.Only.Up.7D
SGMS1		1.082					1.381		1.13		EN.SM.Up.12H, SM.Only.Up.7D
SLC38A1		1.639					1.007		1.029		EN.SM.Up.12H, SM.Only.Up.7D
SLC39A6		1.1					1.609		1.146		EN.SM.Up.12H, SM.Only.Up.7D
SPCS3		1.071					1.118		1.305		EN.SM.Up.12H, SM.Only.Up.7D
TPM4		1.563					2.321		1.426		EN.SM.Up.12H, SM.Only.Up.7D
BXDC1			1.103				1.19	1.345			EN.SM.Up.24H, SM.Only.Up.12H
HNRPDL			1.16				1.68	1.378			EN.SM.Up.24H, SM.Only.Up.12H
LOC478538			1.228				1.631	1.867			EN.SM.Up.24H, SM.Only.Up.12H
LOC480615			1.229				1.295	1.439			EN.SM.Up.24H, SM.Only.Up.12H
LOC483150			1.787				1.113	1.679			EN.SM.Up.24H, SM.Only.Up.12H
POLR2D			1.464				1.255	2.103			EN.SM.Up.24H, SM.Only.Up.12H
SLC7A11			2.044				2.52	2.019			EN.SM.Up.24H, SM.Only.Up.12H
STK38L	2.023	1.446				1.545					EN.SM.Up.2H, EN.Only.Up.12H
LRP2	2.656		1.466			3.11					EN.SM.Up.2H, EN.Only.Up.24H
BDNF	3.584			1.405		3.091					EN.SM.Up.2H, EN.Only.Up.7D
DCUN1D3	1.839					2.183	1.483				EN.SM.Up.2H, SM.Only.Up.12H
DUSP14	2.203					2.874	1.026				EN.SM.Up.2H, SM.Only.Up.12H
GCH1	2.537					1.917	3.456				EN.SM.Up.2H, SM.Only.Up.12H
NFKB1	2.331					2.434	1.02				EN.SM.Up.2H, SM.Only.Up.12H
5-Sep		1.021		1.799					1.171		EN.SM.Up.7D, EN.Only.Up.12H
DBF4		1.188		1.23					1.678		EN.SM.Up.7D, EN.Only.Up.12H
GNPDA1		1.256		1.12					1.52		EN.SM.Up.7D, EN.Only.Up.12H
LOC486254		1.479		2.063					2.532		EN.SM.Up.7D, EN.Only.Up.12H
LOC606770		1.254		1.1					1.213		EN.SM.Up.7D, EN.Only.Up.12H
LRRC8C		1.432		1.001					1.877		EN.SM.Up.7D, EN.Only.Up.12H
PFKP		1.049		1.446					1.076		EN.SM.Up.7D, EN.Only.Up.12H
TGFBR1		1.148		1.092					1.284		EN.SM.Up.7D, EN.Only.Up.12H
TNFSF13B		1.137		1.87					1.372		EN.SM.Up.7D, EN.Only.Up.12H
ADAP2			1.705	2.765					1.65		EN.SM.Up.7D, EN.Only.Up.24H
CD86			2.286	2.966					1.958		EN.SM.Up.7D, EN.Only.Up.24H
CDC6			2.828	1.13					2.885		EN.SM.Up.7D, EN.Only.Up.24H
CENPH			1.422	2.934					3.386		EN.SM.Up.7D, EN.Only.Up.24H
CTSS			2.046	3.608					2.071		EN.SM.Up.7D, EN.Only.Up.24H
DBI			1.329	1.607					1.223		EN.SM.Up.7D, EN.Only.Up.24H
DLA-DRB1			2.776	3.185					2.539		EN.SM.Up.7D, EN.Only.Up.24H
FEN1			2.059	1.56					1.44		EN.SM.Up.7D, EN.Only.Up.24H
LCTL			1.32	1.247					1.81		EN.SM.Up.7D, EN.Only.Up.24H
LMAN1			1.294	1.292					1.624		EN.SM.Up.7D, EN.Only.Up.24H
LOC476669			1.414	2.468					1.503		EN.SM.Up.7D, EN.Only.Up.24H
LOC607827			1.733	1.805					2.144		EN.SM.Up.7D, EN.Only.Up.24H
LOC607964			1.656	1.438					2.36		EN.SM.Up.7D, EN.Only.Up.24H
LOC610710			3.99	1.632					1.391		EN.SM.Up.7D, EN.Only.Up.24H
LOC613005			1.669	2.338					1.99		EN.SM.Up.7D, EN.Only.Up.24H
LRRC46			1.295	1.951					2.027		EN.SM.Up.7D, EN.Only.Up.24H
LTBP3			1.459	2.235					2.396		EN.SM.Up.7D, EN.Only.Up.24H
MCM3			1.583	1.709					1.299		EN.SM.Up.7D, EN.Only.Up.24H
MCM5			1.57	1.356					1.848		EN.SM.Up.7D, EN.Only.Up.24H
MCM6			1.392	1.88					2.641		EN.SM.Up.7D, EN.Only.Up.24H
MNDA			2.047	1.224					1.749		EN.SM.Up.7D, EN.Only.Up.24H
MYO1B			1.847	1.979					2.203		EN.SM.Up.7D, EN.Only.Up.24H
ORC6L			2.459	2.322					2.132		EN.SM.Up.7D, EN.Only.Up.24H
P4HB			1.59	1.322					1.353		EN.SM.Up.7D, EN.Only.Up.24H
PBK			1.306	1.515					1.762		EN.SM.Up.7D, EN.Only.Up.24H
PPT1			1.119	1.299					1.135		EN.SM.Up.7D, EN.Only.Up.24H
RAD51			2.345	2.876					3.121		EN.SM.Up.7D, EN.Only.Up.24H
RAD51AP1			1.949	2.693					2.694		EN.SM.Up.7D, EN.Only.Up.24H
RPN2			1.079	1.082					1.105		EN.SM.Up.7D, EN.Only.Up.24H
SPC25			1.358	2.815					2.203		EN.SM.Up.7D, EN.Only.Up.24H
SQLE			1.893	2.54					2.411		EN.SM.Up.7D, EN.Only.Up.24H
STEAP1			1.266	1.888					1.567		EN.SM.Up.7D, EN.Only.Up.24H
STIL			1.674	1.785					1.834		EN.SM.Up.7D, EN.Only.Up.24H
UCP2			2.32	3.023					2.134		EN.SM.Up.7D, EN.Only.Up.24H
VCAM1			1.572	1.938					1.733		EN.SM.Up.7D, EN.Only.Up.24H
UBE2C	2.459			3.669					3.139		EN.SM.Up.7D, EN.Only.Up.2H
SURF4				1.034			1.391		1.047		EN.SM.Up.7D, SM.Only.Down.12H, SM.Only.Up.12H
ARPC5				1.046			1.223		1.053		EN.SM.Up.7D, SM.Only.Up.12H
CBFB				1.118			1.09		1.021		EN.SM.Up.7D, SM.Only.Up.12H
CIAPIN1				1.217			1.081		1.047		EN.SM.Up.7D, SM.Only.Up.12H
CSTB				1.809			1.135		1.235		EN.SM.Up.7D, SM.Only.Up.12H
CXCL14				2.256			1.293		1.873		EN.SM.Up.7D, SM.Only.Up.12H
EDEM2				1.279			1.021		1.569		EN.SM.Up.7D, SM.Only.Up.12H
ENTPD7				1.411			1.933		2.128		EN.SM.Up.7D, SM.Only.Up.12H
FBXO5				2.069			1.124		1.991		EN.SM.Up.7D, SM.Only.Up.12H
GLA				1.021			1.776		1.521		EN.SM.Up.7D, SM.Only.Up.12H
IFNAR1				1.046			1.098		1.533		EN.SM.Up.7D, SM.Only.Up.12H
IL12A				1.331			3.001		1.639		EN.SM.Up.7D, SM.Only.Up.12H
KBTBD8				1.483			1.223		1.078		EN.SM.Up.7D, SM.Only.Up.12H
LGALS9				2.059			1.844		3.624		EN.SM.Up.7D, SM.Only.Up.12H
LOC475202				1.281			1.4		1.182		EN.SM.Up.7D, SM.Only.Up.12H
LTBR				3.823			1.338		1.143		EN.SM.Up.7D, SM.Only.Up.12H
MAP4K4				1.37			1.024		1.186		EN.SM.Up.7D, SM.Only.Up.12H
OLFM1				2.098			1.301		3.456		EN.SM.Up.7D, SM.Only.Up.12H
PPIB				1.191			1.058		1.244		EN.SM.Up.7D, SM.Only.Up.12H
SHMT2				1.477			1.021		1.328		EN.SM.Up.7D, SM.Only.Up.12H
SLC37A2				2.042			1.142		1.046		EN.SM.Up.7D, SM.Only.Up.12H
SNHG3-RCC1				1.128			1.105		1.539		EN.SM.Up.7D, SM.Only.Up.12H
KDELC1				1.432				1.248	1.521		EN.SM.Up.7D, SM.Only.Up.24H
SRPX				1.576				1.268	1.646		EN.SM.Up.7D, SM.Only.Up.24H
COL29A1				3.316					4.474	3.672	EN.SM.Up.7D, SM.Only.Up.30D
LOC484897				5.497					3.196	3.475	EN.SM.Up.7D, SM.Only.Up.30D
SFRP2				4.463					4.012	5.699	EN.SM.Up.7D, SM.Only.Up.30D
CD274			1.656	2.079			1.228				SM.Only.Up.12H, EN.Only.Up.24H.7D
EMR4P			2.071	1.753			2.327				SM.Only.Up.12H, EN.Only.Up.24H.7D
FOLR2			2.186	2.266			1.083				SM.Only.Up.12H, EN.Only.Up.24H.7D
GEMIN4			1.532	1.178			1.639				SM.Only.Up.12H, EN.Only.Up.24H.7D
MAP2K3			1.762	1.001			1.611				SM.Only.Up.12H, EN.Only.Up.24H.7D
PID1			1.665	2.219			1.282				SM.Only.Up.12H, EN.Only.Up.24H.7D
PPA1			1.553	1.176			1.994				SM.Only.Up.12H, EN.Only.Up.24H.7D
REL			3.006	1.708			2.389				SM.Only.Up.12H, EN.Only.Up.24H.7D
RHOG			1.417	1.555			1.057				SM.Only.Up.12H, EN.Only.Up.24H.7D
RPS20			1.005	1.034			1.454				SM.Only.Up.12H, EN.Only.Up.24H.7D
SLC9A3R1			1.927	2.244			1.903				SM.Only.Up.12H, EN.Only.Up.24H.7D
SSH2			2.74	1.305			1.541				SM.Only.Up.12H, EN.Only.Up.24H.7D
EREG	3.307						1.948	2.713			SM.Only.Up.12H.24H, EN.Only.Up.2H
MYC	3.064						2.41	1.703			SM.Only.Up.12H.24H, EN.Only.Up.2H

**ATP1A1**				1.414			2.345	1.887			SM.Only.Up.12H.24H, EN.Only.Up.7D
**LOC480628**				1.333			1.126	3.64			SM.Only.Up.12H.24H, EN.Only.Up.7D
**LOC608876**				1.013			1.457	1.417			SM.Only.Up.12H.24H, EN.Only.Up.7D
**LOC612666**				1.07			1.881	2.024			SM.Only.Up.12H.24H, EN.Only.Up.7D
**NOP10**				1.134			1.292	1.764			SM.Only.Up.12H.24H, EN.Only.Up.7D
**PDGFRA**				2.542			1.255	2.434			SM.Only.Up.12H.24H, EN.Only.Up.7D
**SLC13A5**				1.04			2.062	1.337			SM.Only.Up.12H.24H, EN.Only.Up.7D
**TMEFF2**				1.729			1.435	2.081			SM.Only.Up.12H.24H, EN.Only.Up.7D
**TNFRSF1A**				1.355			1.682	2.624			SM.Only.Up.12H.24H, EN.Only.Up.7D
**ABCC4**							1.222	2.24	1.6		SM.Only.Up.12H.24H.7D
**ACLY**							1.958	1.543	1.518		SM.Only.Up.12H.24H.7D
**AK7**							2.914	2.454	2.932		SM.Only.Up.12H.24H.7D
**DPP4**							1.755	2.392	2.472		SM.Only.Up.12H.24H.7D
**ENOPH1**							1.639	2.385	1.671		SM.Only.Up.12H.24H.7D
**LAMB1**							1.262	1.641	1.798		SM.Only.Up.12H.24H.7D
**RRBP1**							1.271	1.4	1.454		SM.Only.Up.12H.24H.7D
**TPBG**							1.312	2.392	2.646		SM.Only.Up.12H.24H.7D
**TTLL12**							1.256	1.567	2.683		SM.Only.Up.12H.24H.7D
**FOSB**	4.367						1.312			4.292	SM.Only.Up.12H.30D, EN.Only.Up.2H
**DNAJC14**			1.386				1.062		1.046		SM.Only.Up.12H.7D, EN.Only.Up.24H
**DPP3**			1.482				1.071		1.378		SM.Only.Up.12H.7D, EN.Only.Up.24H
**GARS**			1.907				1.454		1.291		SM.Only.Up.12H.7D, EN.Only.Up.24H
**LBR**			1.197				1.346		1.135		SM.Only.Up.12H.7D, EN.Only.Up.24H
**LDHA**			1.221				1.264		1.135		SM.Only.Up.12H.7D, EN.Only.Up.24H
**LOC609892**			2.248				1.469		1.363		SM.Only.Up.12H.7D, EN.Only.Up.24H
**OASL**			1.249				1.076		1.168		SM.Only.Up.12H.7D, EN.Only.Up.24H
**PIK3CB**			1.733				1.059		1.181		SM.Only.Up.12H.7D, EN.Only.Up.24H
**RAB20**			2.362				3.546		4.693		SM.Only.Up.12H.7D, EN.Only.Up.24H
**RNF144B**			2.364				1.554		1.801		SM.Only.Up.12H.7D, EN.Only.Up.24H
**JAK2**	1.257						1.097		1.092		SM.Only.Up.12H.7D, EN.Only.Up.2H
**SDF2**		1.585	1.635						1.151		SM.Only.Up.7D
**TM4SF1**			1.588	1.825					3.611		SM.Only.Up.7D
**LOC612129**		2.512	3.804						2.434		SM.Only.Up.7D, EN.Only.Up.12H.24H
**SMS**		1.035	1.379						1.043		SM.Only.Up.7D, EN.Only.Up.12H.24H
**DCN**				1.481	2.486						EN.Only.Up.7D.30D
**ECM1**				1.765	2.717						EN.Only.Up.7D.30D
**BMPER**				5.048	3.143						EN.Only.Up.7D.30D
**LOC478566**				2.035	4.195						EN.Only.Up.7D.30D
**KRT18**				4.832	4.299						EN.Only.Up.7D.30D
**ARRB2**		1.204	1.457								EN.Only.Up.12H.24H
**CBWD2**		1.445	1.976								EN.Only.Up.12H.24H
**EIF4EBP1**		1.259	2.245								EN.Only.Up.12H.24H
**GIMAP7**		2.401	2.431								EN.Only.Up.12H.24H
**MALL**		1.18	1.235								EN.Only.Up.12H.24H
**MGST1**		2.367	2.367								EN.Only.Up.12H.24H
**OPRM1**		1.793	1.549								EN.Only.Up.12H.24H
**PRKAR2A**		1.249	1.112								EN.Only.Up.12H.24H
**SERPINB10**		1.821	2.261								EN.Only.Up.12H.24H
**SLC12A2**		1.587	1.703								EN.Only.Up.12H.24H
**STEAP2**		1.618	2.766								EN.Only.Up.12H.24H
**SYT10**		3.114	2.581								EN.Only.Up.12H.24H
**TSPAN5**		1.068	1.143								EN.Only.Up.12H.24H
**XYLT1**		1.402	1.042								EN.Only.Up.12H.24H
**ARPC5L**		1.06		1.429							EN.Only.Up.12H.7D
**ATG4D**		1.07		1.321							EN.Only.Up.12H.7D
**FRMD4A**		1.088		1.346							EN.Only.Up.12H.7D
**LACTB**		1.007		1.258							EN.Only.Up.12H.7D
**NTN4**		1.236		1.704							EN.Only.Up.12H.7D
**SEC61A1**		1.125		1.25							EN.Only.Up.12H.7D
**SERPINB8**		1.179		1.662							EN.Only.Up.12H.7D
**SLC26A11**		1.078		1.25							EN.Only.Up.12H.7D
**VAV3**		2.004		1.499							EN.Only.Up.12H.7D
**CDCA7**			2.627						1.327		EN.Only.Up.24H
**AHCY**			1.524	1.487							EN.Only.Up.24H.7D
**BMP2K**			1.688	1.096							EN.Only.Up.24H.7D
**CCR5**			1.892	1.633							EN.Only.Up.24H.7D
**CD83**			1.15	1.204							EN.Only.Up.24H.7D
**DLA-DQB1**			2.166	3.371							EN.Only.Up.24H.7D
**EZH2**			1.602	1.509							EN.Only.Up.24H.7D
**LOC480602**			3.161	4.27							EN.Only.Up.24H.7D
**LOC484249**			2.445	2.347							EN.Only.Up.24H.7D
**MASTL**			1.136	1.716							EN.Only.Up.24H.7D
**MCM10**			2.016	2.302							EN.Only.Up.24H.7D
**MMP14**			1.403	1.231							EN.Only.Up.24H.7D
**MS4A7**			2.182	2.029							EN.Only.Up.24H.7D
**NAGA**			1.175	1.327							EN.Only.Up.24H.7D
**PCOLCE2**			2.116	2.126							EN.Only.Up.24H.7D
**PDIA3**			1.311	1.009							EN.Only.Up.24H.7D
**PDIA4**			1.449	1.244							EN.Only.Up.24H.7D
**PGAM1**			1.679	1.152							EN.Only.Up.24H.7D
**SFRP5**			1.253	2.309							EN.Only.Up.24H.7D
**SLC26A2**			2.253	1.511							EN.Only.Up.24H.7D
**TCN2**			1.346	1.735							EN.Only.Up.24H.7D
**TFEC**			2.059	2.16							EN.Only.Up.24H.7D
**UST**			1.979	1.728							EN.Only.Up.24H.7D
**ATF3**	5.068	2.657									EN.Only.Up.2H.12H
**ATXN7**	1.947	1.285									EN.Only.Up.2H.12H
**RCAN1**	3.352	1.545									EN.Only.Up.2H.12H
**ZMYND8**	1.625			1.239							EN.Only.Up.2H.7D
**AATF**		1.273					1.065				EN.SM.Up.12H
**ABCE1**		1.245					1.397				EN.SM.Up.12H
**ABCF2**		1.225					1.285				EN.SM.Up.12H
**ACTR8**		1.084					1.423				EN.SM.Up.12H
**ADAMTS9**		2.249					3.359				EN.SM.Up.12H
**ADSL**		1.434					1.349				EN.SM.Up.12H
**AMD1**		1.232					1.035				EN.SM.Up.12H
**AMPD3**		1.345					1.199				EN.SM.Up.12H
**ANKRD28**		1.364					1.285				EN.SM.Up.12H
**AP3M2**		3.965					2.583				EN.SM.Up.12H
**APG-2**		1.513					1.508				EN.SM.Up.12H
**AREG**		2.797					1.888				EN.SM.Up.12H
**ARSJ**		1.455					1.193				EN.SM.Up.12H
**ATAD2B**		1.305					1.152				EN.SM.Up.12H
**ATP6V0A1**		1.261					1.022				EN.SM.Up.12H
**ATP6V0B**		1.32					1.003				EN.SM.Up.12H
**BACH1**		1.499					1.47				EN.SM.Up.12H
**BAIAP2L1**		1.51					1.262				EN.SM.Up.12H
**BANP**		1.153					1.515				EN.SM.Up.12H
**BNIP1**		1.542					1.062				EN.SM.Up.12H
**BYSL**		1.639					1.037				EN.SM.Up.12H
**CA4**		2.603					2.876				EN.SM.Up.12H
**CAMTA1**		1.527					1.919				EN.SM.Up.12H
**CCRL2**		2.35					3.004				EN.SM.Up.12H
**CCT2**		1.059					1.277				EN.SM.Up.12H
**CDC42SE1**		1.703					1.548				EN.SM.Up.12H
**CHORDC1**		1.589					1.222				EN.SM.Up.12H
**CIRH1A**		1.021					2.066				EN.SM.Up.12H
**CR1**		1.062					1.562				EN.SM.Up.12H
**CSNK1G1**		2.594					1.619				EN.SM.Up.12H
**DCAF12L2**		1.287					1.541				EN.SM.Up.12H
**DDX18**		1.331					1.368				EN.SM.Up.12H
**DDX39**		1.194					1.326				EN.SM.Up.12H
**DNAJC25**		1.067					1.373				EN.SM.Up.12H

**DNAJC7**		1.138					1.38				EN.SM.Up.12H
**DPAGT1**		1.196					1.587				EN.SM.Up.12H
**EIF2S2**		1.008					1.174				EN.SM.Up.12H
**EIF5B**		1.478					1.19				EN.SM.Up.12H
**EIF6**		1.15					1.765				EN.SM.Up.12H
**ELAC2**		1.057					1.321				EN.SM.Up.12H
**ELOVL1**		2.027					1.175				EN.SM.Up.12H
**ERC1**		1.071					1.193				EN.SM.Up.12H
**EXOSC3**		1.071					1.065				EN.SM.Up.12H
**FAR1**		1.109					1.169				EN.SM.Up.12H
**FBL**		1.517					2.264				EN.SM.Up.12H
**FES**		1.513					1.339				EN.SM.Up.12H
**FKBP4**		1.296					1.038				EN.SM.Up.12H
**FMNL2**		1.004					1.198				EN.SM.Up.12H
**G3BP1**		1.452					1.523				EN.SM.Up.12H
**GALNT3**		3.465					3.547				EN.SM.Up.12H
**GEMIN6**		1.296					1.509				EN.SM.Up.12H
**GLRX**		1.133					1.041				EN.SM.Up.12H
**GOSR2**		1.165					1.466				EN.SM.Up.12H
**HEATR3**		1.219					1.032				EN.SM.Up.12H
**HMOX2**		1.205					1.377				EN.SM.Up.12H
**HNRNPAB**		1.075					1.217				EN.SM.Up.12H
**HP**		3.252					4.121				EN.SM.Up.12H
**HSPA8**		1.885					2.495				EN.SM.Up.12H
**IER2**		1.398					1.973				EN.SM.Up.12H
**IFITM2**		3.275					3.401				EN.SM.Up.12H
**IFRD2**		1.109					1.103				EN.SM.Up.12H
**IL15RA**		1.95					2.51				EN.SM.Up.12H
**IL1RL2**		2.836					3.2				EN.SM.Up.12H
**IPO4**		1.63					2.138				EN.SM.Up.12H
**IPO5**		1.04					1.039				EN.SM.Up.12H
**ISG20**		1.932					1.954				EN.SM.Up.12H
**IVNS1ABP**		1.077					1.317				EN.SM.Up.12H
**KCNJ2**		3.637					3.273				EN.SM.Up.12H
**LIF**		1.127					2.599				EN.SM.Up.12H
**LOC474739**		1.487					1.418				EN.SM.Up.12H
**LOC474982**		1.311					1.219				EN.SM.Up.12H
**LOC475487**		1.101					1.198				EN.SM.Up.12H
**LOC475935**		4.503					5.575				EN.SM.Up.12H
**LOC476337**		1.036					1.068				EN.SM.Up.12H
**LOC477835**		1.124					1.084				EN.SM.Up.12H
**LOC478015**		1.235					1.127				EN.SM.Up.12H
**LOC478234**		1.283					1.058				EN.SM.Up.12H
**LOC479798**		1.388					1.533				EN.SM.Up.12H
**LOC480097**		1.707					1.283				EN.SM.Up.12H
**LOC480714**		1.473					2.087				EN.SM.Up.12H
**LOC482733**		1.054					1.397				EN.SM.Up.12H
**LOC484482**		1.254					1.175				EN.SM.Up.12H
**LOC485196**		1.937					1.591				EN.SM.Up.12H
**LOC486535**		1.127					1.212				EN.SM.Up.12H
**LOC487497**		1.172					1.247				EN.SM.Up.12H
**LOC607454**		1.198					1.01				EN.SM.Up.12H
**LOC607910**		1.267					1.353				EN.SM.Up.12H
**LOC608717**		1.059					1.17				EN.SM.Up.12H
**LOC609631**		1.371					1.602				EN.SM.Up.12H
**LOC611809**		2.762					3.17				EN.SM.Up.12H
**LOC611892**		1.67					1.795				EN.SM.Up.12H
**LOC612872**		1.919					2.029				EN.SM.Up.12H
**LRRC63**		1.068					1.508				EN.SM.Up.12H
**LSG1**		1.033					1.081				EN.SM.Up.12H
**LTF**		1.027					3.936				EN.SM.Up.12H
**LTV1**		1.171					1.341				EN.SM.Up.12H
**MAGOH**		1.078					1.51				EN.SM.Up.12H
**MAPKAPK2**		1.404					1.394				EN.SM.Up.12H
**MEGF9**		1.967					1.294				EN.SM.Up.12H
**METTL1**		1.554					3.351				EN.SM.Up.12H
**MFSD2**		1.134					1.61				EN.SM.Up.12H
**MGAM**		2.26					2.336				EN.SM.Up.12H
**MMP1**		1.749					1.445				EN.SM.Up.12H
**MMP8**		4.309					4.835				EN.SM.Up.12H
**MRPS16**		1.49					1.219				EN.SM.Up.12H
**MRTO4**		1.22					1.236				EN.SM.Up.12H
**MTO1**		1.239					1.001				EN.SM.Up.12H
**MYCN**		1.352					1.116				EN.SM.Up.12H
**NAV2**		1.463					1.07				EN.SM.Up.12H
**NCF1**		2.802					2.694				EN.SM.Up.12H
**NDFIP2**		1.093					1.005				EN.SM.Up.12H
**NFIL3**		1.946					2.031				EN.SM.Up.12H
**NHP2**		1.022					1.655				EN.SM.Up.12H
**NOC4L**		1.8					1.328				EN.SM.Up.12H
**NOP56**		1.34					1.423				EN.SM.Up.12H
**NR2C2AP**		1.214					1.035				EN.SM.Up.12H
**NXT1**		1.245					1.221				EN.SM.Up.12H
**OAF**		1.074					1.013				EN.SM.Up.12H
**PADI2**		1.086					1.096				EN.SM.Up.12H
**PAK1IP1**		1.326					1.516				EN.SM.Up.12H
**PDCD11**		1.486					1.593				EN.SM.Up.12H
**PGS1**		1.698					1.445				EN.SM.Up.12H
**PITPNC1**		1.356					1.462				EN.SM.Up.12H
**PLA2G4A**		1.29					1.368				EN.SM.Up.12H
**PLCG2**		2.415					2.296				EN.SM.Up.12H
**PLXNA2**		1.51					1.068				EN.SM.Up.12H
**POLR3E**		1.199					1.306				EN.SM.Up.12H
**PRDM1**		1.474					1.876				EN.SM.Up.12H
**PRDX6**		1.161					1.796				EN.SM.Up.12H
**PRMT1**		1.058					1.283				EN.SM.Up.12H
**PRMT3**		1.082					1.289				EN.SM.Up.12H
**PRMT5**		1.208					1.604				EN.SM.Up.12H
**PSMD9**		1.177					1				EN.SM.Up.12H
**PTAFR**		1.444					1.302				EN.SM.Up.12H
**PTK2**		2.409					1.055				EN.SM.Up.12H
**PTPN1**		1.739					1.614				EN.SM.Up.12H
**PTPRK**		1.991					1.532				EN.SM.Up.12H
**PWP1**		1.072					1.197				EN.SM.Up.12H
**QPCT**		1.573					1.55				EN.SM.Up.12H
**RAB24**		1.871					1.384				EN.SM.Up.12H
**RASSF1**		1.379					1.561				EN.SM.Up.12H
**RBMS1**		1.154					1.207				EN.SM.Up.12H
**RILPL2**		1.682					1.467				EN.SM.Up.12H
**RRP12**		1.342					1.554				EN.SM.Up.12H
**RRP1B**		1.467					1.627				EN.SM.Up.12H
**SEC23B**		1.199					2.039				EN.SM.Up.12H
**SERHL2**		1.353					1.15				EN.SM.Up.12H
**SETD8**		1.06					1.137				EN.SM.Up.12H
**SIRPG**		1.291					1.361				EN.SM.Up.12H
**SLAMF7**		1.141					1.46				EN.SM.Up.12H
**SLC17A2**		1.51					1.29				EN.SM.Up.12H
**SLC28A3**		2.138					1.979				EN.SM.Up.12H
**SLC30A9**		1.189					1.023				EN.SM.Up.12H
**SLC35B1**		1.022					1.211				EN.SM.Up.12H
**SLC9A11**		2.214					2.58				EN.SM.Up.12H
**SLCO6A1**		1.591					1.305				EN.SM.Up.12H
**SMAP2**		1.391					1.031				EN.SM.Up.12H

SNRPA1		1.156					1.543				EN.SM.Up.12H
SNRPF		1.467					1.544				EN.SM.Up.12H
SPI1		1.71					1.095				EN.SM.Up.12H
SPTY2D1		1.283					1.016				EN.SM.Up.12H
STEAP4		3.353					1.524				EN.SM.Up.12H
STIP1		1.295					1.059				EN.SM.Up.12H
STK35		1.379					1.075				EN.SM.Up.12H
STK4		1.324					1.456				EN.SM.Up.12H
TAF9		1.001					1.195				EN.SM.Up.12H
TAGLN3		1.651					1.456				EN.SM.Up.12H
THBD		1.301					1.941				EN.SM.Up.12H
TM9SF4		1.349					1.181				EN.SM.Up.12H
TMEM201		2.057					2.221				EN.SM.Up.12H
TNK2		1.247					1.132				EN.SM.Up.12H
TPD52		1.561					1.991				EN.SM.Up.12H
TREML2		1.992					1.729				EN.SM.Up.12H
TTC27		1.561					2.179				EN.SM.Up.12H
UBE2S		1.603					1.231				EN.SM.Up.12H
UXT		1.012					1.228				EN.SM.Up.12H
WDR46		1.759					1.65				EN.SM.Up.12H
WDR75		1.019					1.45				EN.SM.Up.12H
XDH		1.119					1.5				EN.SM.Up.12H
YRDC		1.288					1.786				EN.SM.Up.12H
ZDHHC7		1.168					1.139				EN.SM.Up.12H
ZNF259		1.214					1.507				EN.SM.Up.12H
ZNF276		1.605					1.463				EN.SM.Up.12H
OTUD1	2.592					2.409					EN.SM.Up.2H
ACTR2				1.14					1.233		EN.SM.Up.7D
ADA				1.501					1.931		EN.SM.Up.7D
ADAM28				2.255					1.692		EN.SM.Up.7D
ADAMTS3				1.614					1.521		EN.SM.Up.7D
ADPGK				1.039					1.14		EN.SM.Up.7D
ALCAM				1.155					1.022		EN.SM.Up.7D
ALDH1L2				1.15					1.9		EN.SM.Up.7D
ANLN				4.227					4.278		EN.SM.Up.7D
ANO1				1.506					1.188		EN.SM.Up.7D
AP1S2				1.004					1.394		EN.SM.Up.7D
ARHGAP11A				1.79					1.783		EN.SM.Up.7D
ARPC4				1.091					1.454		EN.SM.Up.7D
ASPM				3.105					4.328		EN.SM.Up.7D
ATAD2				2.355					2.384		EN.SM.Up.7D
ATMIN				1.421					1.014		EN.SM.Up.7D
AURKA				1.954					2.16		EN.SM.Up.7D
BIRC5				2.119					2.247		EN.SM.Up.7D
BNC2				1.405					1.263		EN.SM.Up.7D
BUB1B				2.889					3.051		EN.SM.Up.7D
C3AR1				3.947					3.154		EN.SM.Up.7D
CASC5				2.319					2.636		EN.SM.Up.7D
CASP6				1.048					1.162		EN.SM.Up.7D
CCNA2				3.865					3.548		EN.SM.Up.7D
CCNB2				1.773					1.699		EN.SM.Up.7D
CCNE2				1.244					1.272		EN.SM.Up.7D
CDC2				2.852					3.193		EN.SM.Up.7D
CDC20				1.514					1.355		EN.SM.Up.7D
CDCA2				1.946					1.809		EN.SM.Up.7D
CDCA3				2.327					2.142		EN.SM.Up.7D
CDKN3				1.103					1.344		EN.SM.Up.7D
CENPF				5.957					5.914		EN.SM.Up.7D
CENPO				1.206					2.181		EN.SM.Up.7D
CENPP				1.248					1.121		EN.SM.Up.7D
CEP170				1.016					1.082		EN.SM.Up.7D
CHST10				1.103					1.392		EN.SM.Up.7D
CHSY3				2.094					2.512		EN.SM.Up.7D
CIB1				1.006					1.351		EN.SM.Up.7D
CKAP2L				2.4					2.283		EN.SM.Up.7D
COL15A1				1.395					1.84		EN.SM.Up.7D
COL4A2				1.437					1.125		EN.SM.Up.7D
COMP				1.722					3.43		EN.SM.Up.7D
CRABP2				2.315					2.395		EN.SM.Up.7D
CTSA				1.974					1.292		EN.SM.Up.7D
DAB2				1.138					1.13		EN.SM.Up.7D
DCK				2.459					2.507		EN.SM.Up.7D
DEPDC1				4.376					5.048		EN.SM.Up.7D
DEPDC1B				1.665					1.741		EN.SM.Up.7D
DIAPH3				4.227					3.743		EN.SM.Up.7D
DLGAP5				3.498					4.043		EN.SM.Up.7D
DNA2				2.461					2.561		EN.SM.Up.7D
DNAJC1				1.09					1.52		EN.SM.Up.7D
DPYSL3				1.265					1.565		EN.SM.Up.7D
DPYSL5				1.782					1.808		EN.SM.Up.7D
DSE				1.131					1.253		EN.SM.Up.7D
E2F8				1.717					1.654		EN.SM.Up.7D
ECT2				2.713					3.966		EN.SM.Up.7D
ERCC6L				1.794					2.876		EN.SM.Up.7D
ERP29				1.322					1.15		EN.SM.Up.7D
ESCO2				2.676					2.824		EN.SM.Up.7D
EXO1				1.534					1.441		EN.SM.Up.7D
FANCM				1.896					2.414		EN.SM.Up.7D
FBN1				1.11					1.513		EN.SM.Up.7D
FCGR3A				3.511					3.046		EN.SM.Up.7D
FKBP1B				1.118					2.175		EN.SM.Up.7D
FMN1				1.164					1.08		EN.SM.Up.7D
FOXM1				4.368					3.755		EN.SM.Up.7D
FSTL1				1.039					1.161		EN.SM.Up.7D
GBA				1.278					1.042		EN.SM.Up.7D
GEN1				1.856					2.191		EN.SM.Up.7D
GLDN				2.92					2.514		EN.SM.Up.7D
GSG2				1.933					1.65		EN.SM.Up.7D
HLA-DMA				1.926					2.411		EN.SM.Up.7D
HMGB3				2.324					1.911		EN.SM.Up.7D
HMMR				1.803					2.092		EN.SM.Up.7D
HORMAD1				1.275					1.399		EN.SM.Up.7D
IL10RB				1.231					1.34		EN.SM.Up.7D
INCENP				1.188					1.424		EN.SM.Up.7D
ITGAV				1.576					1.189		EN.SM.Up.7D
KDELR3				1.11					2.066		EN.SM.Up.7D
KIF11				4.367					5.162		EN.SM.Up.7D
KIF14				2.423					2.719		EN.SM.Up.7D
KIF18A				1.357					1.815		EN.SM.Up.7D
KIF23				3.242					3.886		EN.SM.Up.7D
KIF27				1.267					1.608		EN.SM.Up.7D
KIFC1				1.836					1.515		EN.SM.Up.7D
KLRG1				2.557					2.09		EN.SM.Up.7D
LOC476944				1.213					1.496		EN.SM.Up.7D
LOC477773				2.75					3.246		EN.SM.Up.7D
LOC478258				2.454					2.454		EN.SM.Up.7D
LOC478335				1.274					1.435		EN.SM.Up.7D
LOC478349				1.127					1.305		EN.SM.Up.7D
LOC478384				2.432					1.585		EN.SM.Up.7D
LOC478498				1.782					1.862		EN.SM.Up.7D
LOC478559				1.728					1.486		EN.SM.Up.7D
LOC479820				1.797					1.02		EN.SM.Up.7D
LOC480027				3.837					4.084		EN.SM.Up.7D

**LOC480926**				1.224					1.916		EN.SM.Up.7D
**LOC483842**				1.353					2.68		EN.SM.Up.7D
**LOC485338**				3.523					4.082		EN.SM.Up.7D
**LOC490554**				1.505					1.451		EN.SM.Up.7D
**LOC607731**				1.578					1.726		EN.SM.Up.7D
**LOC608673**				1.473					2.206		EN.SM.Up.7D
**LOC609701**				1.659					1.987		EN.SM.Up.7D
**LOC609897**				2.067					1.622		EN.SM.Up.7D
**LOC610244**				1.476					1.636		EN.SM.Up.7D
**LOC612196**				3.173					3.224		EN.SM.Up.7D
**LOC612298**				3.195					3.237		EN.SM.Up.7D
**MAD2L1**				2.12					1.964		EN.SM.Up.7D
**MAN1A1**				1.639					1.543		EN.SM.Up.7D
**MAN2A1**				1.3					1.744		EN.SM.Up.7D
**MATN3**				3.311					4.091		EN.SM.Up.7D
**MEST**				1.375					1.515		EN.SM.Up.7D
**MFSD11**				1.678					1.188		EN.SM.Up.7D
**MIS12**				1.667					1.338		EN.SM.Up.7D
**MMD**				1.608					1.119		EN.SM.Up.7D
**MSN**				1.015					1.181		EN.SM.Up.7D
**MSR1**				4.458					3.638		EN.SM.Up.7D
**NCAPD2**				1.078					1.215		EN.SM.Up.7D
**NCAPD3**				1.098					1.54		EN.SM.Up.7D
**NCAPG**				1.933					1.421		EN.SM.Up.7D
**NCAPH**				2.052					2.693		EN.SM.Up.7D
**NEGR1**				2.602					1.117		EN.SM.Up.7D
**NEK2**				1.194					1.412		EN.SM.Up.7D
**NNT**				1.006					1.551		EN.SM.Up.7D
**NPC2**				1.452					1.388		EN.SM.Up.7D
**NUF2**				3.155					3.497		EN.SM.Up.7D
**OAS2**				2.07					2.934		EN.SM.Up.7D
**P2RX4**				1.225					1.544		EN.SM.Up.7D
**PHTF1**				1.528					1.39		EN.SM.Up.7D
**PLK1**				3.002					2.368		EN.SM.Up.7D
**PLK4**				1.156					1.385		EN.SM.Up.7D
**PLOD1**				1.419					1.58		EN.SM.Up.7D
**PLOD2**				1.233					1.231		EN.SM.Up.7D
**PMCH**				1.922					1.31		EN.SM.Up.7D
**POLA2**				1.297					1.384		EN.SM.Up.7D
**PPIC**				1.012					1.668		EN.SM.Up.7D
**PPP1CA**				1.432					1.048		EN.SM.Up.7D
**PRC1**				3.672					3.335		EN.SM.Up.7D
**PRKX**				1.584					1.703		EN.SM.Up.7D
**PSMC3IP**				1.167					1.149		EN.SM.Up.7D
**PTTG1**				4.135					4.634		EN.SM.Up.7D
**RACGAP1**				3.903					3.827		EN.SM.Up.7D
**RCC2**				1.369					1.28		EN.SM.Up.7D
**REEP4**				2.466					2.253		EN.SM.Up.7D
**RFC3**				1.253					1.189		EN.SM.Up.7D
**RFC4**				1.161					1.292		EN.SM.Up.7D
**RRM1**				1.09					1.357		EN.SM.Up.7D
**SASS6**				2.026					2.246		EN.SM.Up.7D
**SCG3**				2.452					2.563		EN.SM.Up.7D
**SEC23A**				1.313					1.029		EN.SM.Up.7D
**SERPINH1**				1.572					1.378		EN.SM.Up.7D
**SGOL2**				1.061					1.179		EN.SM.Up.7D
**SGPL1**				1.306					2.295		EN.SM.Up.7D
**SH3KBP1**				1.308					1.705		EN.SM.Up.7D
**SHCBP1**				3.235					3.34		EN.SM.Up.7D
**SLC6A6**				1.613					1.751		EN.SM.Up.7D
**SMC4**				1.198					1.549		EN.SM.Up.7D
**SNAI2**				1.349					1.916		EN.SM.Up.7D
**SPAG5**				2.899					3.149		EN.SM.Up.7D
**STAT1**				1.09					1.227		EN.SM.Up.7D
**STC2**				1.277					2.144		EN.SM.Up.7D
**TANC2**				1.405					1.505		EN.SM.Up.7D
**TF**				3.158					2.278		EN.SM.Up.7D
**TP53INP1**				2.003					1.724		EN.SM.Up.7D
**TRAPPC1**				1.55					1.264		EN.SM.Up.7D
**TRIM36**				2.976					2.831		EN.SM.Up.7D
**TTK**				1.184					1.733		EN.SM.Up.7D
**UGGT2**				1.238					1.633		EN.SM.Up.7D
**UNC119**				1.891					1.334		EN.SM.Up.7D
**XPNPEP1**				1.047					1.265		EN.SM.Up.7D
**ZFHX4**				1.41					1.276		EN.SM.Up.7D
**CFB**							1.575		1.657		SM.Only.Down.12H, SM.Only.Up.12H.7D
**LGALS3**			2.311						1.315		SM.Only.Down.7D, SM.Only.Up.7D, EN.Only.Up.24H
**PLP2**			1.121						1.078		SM.Only.Down.7D, SM.Only.Up.7D, EN.Only.Up.24H
**ADSS**	1.399						1.142				SM.Only.Up.12H
**CCNJ**		1.299					1.044				SM.Only.Up.12H
**DCAF12**		1.287					1.169				SM.Only.Up.12H
**IL15**		1.95					1.029				SM.Only.Up.12H
**SLC7A1**			2.044				1.102				SM.Only.Up.12H
**VIP**		1.185					2.122				SM.Only.Up.12H
**ADRBK1**			1.527				1.713				SM.Only.Up.12H, EN.Only.Up.24H
**AMBP**			1.397				1.575				SM.Only.Up.12H, EN.Only.Up.24H
**CLP1**			1.044				1.168				SM.Only.Up.12H, EN.Only.Up.24H
**COQ10B**			1.653				1.114				SM.Only.Up.12H, EN.Only.Up.24H
**CXCL10**			1.397				1.291				SM.Only.Up.12H, EN.Only.Up.24H
**ECE2**			1.198				1.748				SM.Only.Up.12H, EN.Only.Up.24H
**EPB41**			1.147				1.043				SM.Only.Up.12H, EN.Only.Up.24H
**FGD4**			1.298				1.16				SM.Only.Up.12H, EN.Only.Up.24H
**GPR171**			1.278				1.657				SM.Only.Up.12H, EN.Only.Up.24H
**HHEX**			1.568				3.35				SM.Only.Up.12H, EN.Only.Up.24H
**LOC475958**			1.531				1.363				SM.Only.Up.12H, EN.Only.Up.24H
**LOC477662**			1.447				1.54				SM.Only.Up.12H, EN.Only.Up.24H
**LOC611656**			1.106				1.074				SM.Only.Up.12H, EN.Only.Up.24H
**NMT1**			1.379				1.058				SM.Only.Up.12H, EN.Only.Up.24H
**ODF2L**			1.725				1.477				SM.Only.Up.12H, EN.Only.Up.24H
**PGD**			1.051				1.313				SM.Only.Up.12H, EN.Only.Up.24H
**PIGX**			1.9				1.184				SM.Only.Up.12H, EN.Only.Up.24H
**PIK3CD**			2.08				1.507				SM.Only.Up.12H, EN.Only.Up.24H
**PIM1**			2.194				2.228				SM.Only.Up.12H, EN.Only.Up.24H
**PSMA6**			1.189				1.08				SM.Only.Up.12H, EN.Only.Up.24H
**RGS2**			1.29				1.067				SM.Only.Up.12H, EN.Only.Up.24H
**SLC25A38**			2.726				1.104				SM.Only.Up.12H, EN.Only.Up.24H
**SOAT1**			1.898				1.268				SM.Only.Up.12H, EN.Only.Up.24H
**SPINT1**			2.832				2.471				SM.Only.Up.12H, EN.Only.Up.24H
**STRAP**			1.162				1.559				SM.Only.Up.12H, EN.Only.Up.24H
**TDG**			1.447				1.217				SM.Only.Up.12H, EN.Only.Up.24H
**TMEM138**			1.469				1.749				SM.Only.Up.12H, EN.Only.Up.24H
**TPMT**			2.261				1.443				SM.Only.Up.12H, EN.Only.Up.24H
**TRMT61B**			1.404				1.176				SM.Only.Up.12H, EN.Only.Up.24H
**ARL5B**	2.834						1.266				SM.Only.Up.12H, EN.Only.Up.2H
**BTG2**	2.96						1.084				SM.Only.Up.12H, EN.Only.Up.2H
**EYA4**	2.678						1.607				SM.Only.Up.12H, EN.Only.Up.2H
**FGF19**	2.744						3.316				SM.Only.Up.12H, EN.Only.Up.2H
**GFPT2**	4.866						2.372				SM.Only.Up.12H, EN.Only.Up.2H
**GTF2B**	2.367						1.401				SM.Only.Up.12H, EN.Only.Up.2H
**IRAK2**	2.614						1.562				SM.Only.Up.12H, EN.Only.Up.2H
**KDM6B**	1.563						1.346				SM.Only.Up.12H, EN.Only.Up.2H
**KIN**	1.92						2.203				SM.Only.Up.12H, EN.Only.Up.2H
**LANCL2**	1.348						1.166				SM.Only.Up.12H, EN.Only.Up.2H

LOC481657	1.618						1.239				SM.Only.Up.12H, EN.Only.Up.2H
NDEL1	1.669						1.168				SM.Only.Up.12H, EN.Only.Up.2H
NFATC2	2.77						1.901				SM.Only.Up.12H, EN.Only.Up.2H
PELI1	1.424						1.263				SM.Only.Up.12H, EN.Only.Up.2H
PFKFB2	3.259						1.691				SM.Only.Up.12H, EN.Only.Up.2H
PLAU	1.528						1.294				SM.Only.Up.12H, EN.Only.Up.2H
PTP4A1	1.637						1.202				SM.Only.Up.12H, EN.Only.Up.2H
RNF138	1.662						1.483				SM.Only.Up.12H, EN.Only.Up.2H
RNF19B	2.815						1.694				SM.Only.Up.12H, EN.Only.Up.2H
SEC24A	2.06						1.141				SM.Only.Up.12H, EN.Only.Up.2H
SERTAD1	1.579						1.278				SM.Only.Up.12H, EN.Only.Up.2H
TNFAIP3	3.249						1.357				SM.Only.Up.12H, EN.Only.Up.2H
ZNFX1	1.704						1.047				SM.Only.Up.12H, EN.Only.Up.2H
ATP6V0E1				1.231			1.048				SM.Only.Up.12H, EN.Only.Up.7D
DONSON				1.348			1.232				SM.Only.Up.12H, EN.Only.Up.7D
GOSR1				1.006			1.24				SM.Only.Up.12H, EN.Only.Up.7D
ITGA5				1.225			1.027				SM.Only.Up.12H, EN.Only.Up.7D
LOC608355				1.038			1.35				SM.Only.Up.12H, EN.Only.Up.7D
LOC610510				1.57			1.336				SM.Only.Up.12H, EN.Only.Up.7D
LRFN4				2.135			1.317				SM.Only.Up.12H, EN.Only.Up.7D
NOL9				1.452			1.305				SM.Only.Up.12H, EN.Only.Up.7D
PGLS				1.6			1.25				SM.Only.Up.12H, EN.Only.Up.7D
PLEKHM1				1.096			1.059				SM.Only.Up.12H, EN.Only.Up.7D
PTRH1				1.055			2.033				SM.Only.Up.12H, EN.Only.Up.7D
RELN				1.349			1.895				SM.Only.Up.12H, EN.Only.Up.7D
RNASEK				1.114			1.056				SM.Only.Up.12H, EN.Only.Up.7D
SH3BGRL3				1.052			1.324				SM.Only.Up.12H, EN.Only.Up.7D
SLC2A1				1.272			1.155				SM.Only.Up.12H, EN.Only.Up.7D
SLC43A2				1.056			1.596				SM.Only.Up.12H, EN.Only.Up.7D
TIMM8B				1.396			1.269				SM.Only.Up.12H, EN.Only.Up.7D
TNFAIP2				1.182			1.226				SM.Only.Up.12H, EN.Only.Up.7D
ALG3							1.398	1.85			SM.Only.Up.12H.24H
ANGPT2							1.915	2.229			SM.Only.Up.12H.24H
ANXA2							1.242	1.329			SM.Only.Up.12H.24H
CLPTM1L							1.59	1.652			SM.Only.Up.12H.24H
CUL1							2.546	1.486			SM.Only.Up.12H.24H
DDX54							1.255	1.956			SM.Only.Up.12H.24H
EML4							1.975	1.527			SM.Only.Up.12H.24H
GCSH							1.123	2.472			SM.Only.Up.12H.24H
IL33							1.708	2.396			SM.Only.Up.12H.24H
ILF3							1.613	2.102			SM.Only.Up.12H.24H
ITPR2							1.15	2.241			SM.Only.Up.12H.24H
MAPKSP1							1.01	1.567			SM.Only.Up.12H.24H
MKI67IP							1.608	1.522			SM.Only.Up.12H.24H
NAV3							1.562	2.749			SM.Only.Up.12H.24H
POLR1A							1.489	1.854			SM.Only.Up.12H.24H
POLR3D							1.435	1.624			SM.Only.Up.12H.24H
PSMB10							1.074	2.212			SM.Only.Up.12H.24H
PTGES							2.309	2.181			SM.Only.Up.12H.24H
RANGRF							1.334	2.127			SM.Only.Up.12H.24H
RUVBL1							1.085	1.332			SM.Only.Up.12H.24H
SGK1							1.609	1.309			SM.Only.Up.12H.24H
SLC25A35							1.643	1.462			SM.Only.Up.12H.24H
TCOF1							1.449	1.553			SM.Only.Up.12H.24H
TIMM8A							1.923	1.798			SM.Only.Up.12H.24H
TMEM100							2.774	1.792			SM.Only.Up.12H.24H
TMEM209							1.346	2.025			SM.Only.Up.12H.24H
FOS							1.309			2.634	SM.Only.Up.12H.30D
ACSL5							1.534		2.444		SM.Only.Up.12H.7D
ALDH18A1							1.092		1.211		SM.Only.Up.12H.7D
AMDHD1							2.063		1.206		SM.Only.Up.12H.7D
ANKAR							1.76		1.011		SM.Only.Up.12H.7D
AP3S2							1.195		1.521		SM.Only.Up.12H.7D
ARG1							1.854		1.124		SM.Only.Up.12H.7D
ARG2							1.841		1.35		SM.Only.Up.12H.7D
AZIN1							1.152		1.329		SM.Only.Up.12H.7D
B2M							1.138		1.696		SM.Only.Up.12H.7D
CD200R1							1.344		1.927		SM.Only.Up.12H.7D
CD40							1.97		2.743		SM.Only.Up.12H.7D
CD47							1.478		1.418		SM.Only.Up.12H.7D
CSTF3							1.517		1.352		SM.Only.Up.12H.7D
DCUN1D5							1.184		1.149		SM.Only.Up.12H.7D
EIF1AD							1.071		2.026		SM.Only.Up.12H.7D
ELK3							1.045		1.117		SM.Only.Up.12H.7D
FANK1							1.207		1.794		SM.Only.Up.12H.7D
FGF10							2.18		1.231		SM.Only.Up.12H.7D
FLT1							1.429		1.659		SM.Only.Up.12H.7D
FNDC3B							1.184		1.125		SM.Only.Up.12H.7D
ICAM1							1.548		1.592		SM.Only.Up.12H.7D
IL1R1							1.266		1.989		SM.Only.Up.12H.7D
IL27RA							1.004		1.431		SM.Only.Up.12H.7D
ISG15							1.228		4.621		SM.Only.Up.12H.7D
ITPRIPL1							2.199		1.396		SM.Only.Up.12H.7D
KDELR2							1.376		1.008		SM.Only.Up.12H.7D
LAP3							1.606		1.399		SM.Only.Up.12H.7D
LIMK2							1.13		1.522		SM.Only.Up.12H.7D
LMO2							1.398		1.957		SM.Only.Up.12H.7D
LOC475733							1.218		1.27		SM.Only.Up.12H.7D
LOC478264							1.182		1.005		SM.Only.Up.12H.7D
LOC483799							2.177		1.776		SM.Only.Up.12H.7D
LOC608794							3.068		3.15		SM.Only.Up.12H.7D
LOC612337							1.331		1.324		SM.Only.Up.12H.7D
MARCKSL1							1.687		1.296		SM.Only.Up.12H.7D
MX2							1.467		3.457		SM.Only.Up.12H.7D
NLK							1.044		1.942		SM.Only.Up.12H.7D
NME1-NME2							1.052		1.013		SM.Only.Up.12H.7D
OSTC							1.126		1.748		SM.Only.Up.12H.7D
OSTF1							1.182		1.315		SM.Only.Up.12H.7D
PTPRJ							1.421		1.104		SM.Only.Up.12H.7D
PUS7							1.852		1.224		SM.Only.Up.12H.7D
RAB27A							1.793		1.533		SM.Only.Up.12H.7D
RAB9A							1.185		1.047		SM.Only.Up.12H.7D
RBKS							1.318		1.958		SM.Only.Up.12H.7D
RDH10							3.126		1.134		SM.Only.Up.12H.7D
SDCBP							1.026		1.164		SM.Only.Up.12H.7D
SEC31A							1.062		1.105		SM.Only.Up.12H.7D
Sec61b							1.048		1.202		SM.Only.Up.12H.7D
SHC3							1.062		1.72		SM.Only.Up.12H.7D
SLC11A2							1.512		1.32		SM.Only.Up.12H.7D
SLC16A13							1.068		3.292		SM.Only.Up.12H.7D
SLC41A2							1.089		1.238		SM.Only.Up.12H.7D
SP100							1.333		1.781		SM.Only.Up.12H.7D
STAT4							1.101		1.049		SM.Only.Up.12H.7D
STT3A							1.049		1.015		SM.Only.Up.12H.7D
TJP2							1.285		2.555		SM.Only.Up.12H.7D
TM9SF1							1.245		1.045		SM.Only.Up.12H.7D
TSEN2							1.167		1.048		SM.Only.Up.12H.7D
TXNDC12							1.047		1.055		SM.Only.Up.12H.7D
UAP1							1.464		1.17		SM.Only.Up.12H.7D
ZNF281							1.583		1.116		SM.Only.Up.12H.7D
BMP2		2.064						1.998			SM.Only.Up.24H, EN.Only.Up.12H
EMP3		1.462						2.055			SM.Only.Up.24H, EN.Only.Up.12H
FN1				1.192				1.361			SM.Only.Up.24H, EN.Only.Up.7D

IL13RA1								1.653	1.179		SM.Only.Up.24H.7D
LOC607137								2.021	1.182		SM.Only.Up.24H.7D
SHC1								1.313	1.502		SM.Only.Up.24H.7D
LOC607662						2.515	1.161				SM.Only.Up.2H.12H
NCAM2						1.809	1.004				SM.Only.Up.2H.12H
CKAP2				2.4					1.053		SM.Only.Up.7D
LRRC6		1.068							1.656		SM.Only.Up.7D
ODF2			1.725						1.441		SM.Only.Up.7D
PTPN2		2.233							1.303		SM.Only.Up.7D
CHEK2		2.233							2.083		SM.Only.Up.7D, EN.Only.Up.12H
DNAJC2		1.084							1.192		SM.Only.Up.7D, EN.Only.Up.12H
LOC474524		1.193							1.252		SM.Only.Up.7D, EN.Only.Up.12H
LOC608405		1.2							1.522		SM.Only.Up.7D, EN.Only.Up.12H
P4HA1		1.037							1.294		SM.Only.Up.7D, EN.Only.Up.12H
CCDC99			1.026						1.002		SM.Only.Up.7D, EN.Only.Up.24H
HERC6			1.936						2.53		SM.Only.Up.7D, EN.Only.Up.24H
HRSP12			1.534						1.097		SM.Only.Up.7D, EN.Only.Up.24H
LOC475225			1.156						1.23		SM.Only.Up.7D, EN.Only.Up.24H
LOC480414			2.701						1.318		SM.Only.Up.7D, EN.Only.Up.24H
LOC608702			1.569						1.216		SM.Only.Up.7D, EN.Only.Up.24H
MEMO1			1.043						1.134		SM.Only.Up.7D, EN.Only.Up.24H
NIPA1			1.66						1.451		SM.Only.Up.7D, EN.Only.Up.24H
PMS2			1.138						1.002		SM.Only.Up.7D, EN.Only.Up.24H
RAP2C			1.052						1.152		SM.Only.Up.7D, EN.Only.Up.24H
SLC46A2			4.37						1.361		SM.Only.Up.7D, EN.Only.Up.24H
TRIP13			2.183						2.348		SM.Only.Up.7D, EN.Only.Up.24H
POSTN									6.001	4.421	SM.Only.Up.7D.30D
WASF1					2.173						EN.Only.Up.30D
ABHD12B		1.661									EN.Only.Up.12H
ABHD2		1.386									EN.Only.Up.12H
ABHD5		1.138									EN.Only.Up.12H
ADAM19		1.223									EN.Only.Up.12H
BCL6		1.261									EN.Only.Up.12H
BLZF1		1.104									EN.Only.Up.12H
BTLA		1.054									EN.Only.Up.12H
CAPN2		1.383									EN.Only.Up.12H
CCND1		1.465									EN.Only.Up.12H
CCNJL		1.299									EN.Only.Up.12H
CD46		1.861									EN.Only.Up.12H
CNOT10		1.035									EN.Only.Up.12H
CP		1.674									EN.Only.Up.12H
CTR9		1.023									EN.Only.Up.12H
CUBN		1.978									EN.Only.Up.12H
DERL1		1.045									EN.Only.Up.12H
DLC1		1.133									EN.Only.Up.12H
EIF2C2		1.068									EN.Only.Up.12H
ELL3		1.088									EN.Only.Up.12H
ERI2		1.226									EN.Only.Up.12H
ERMP1		1.193									EN.Only.Up.12H
EXD1		1.095									EN.Only.Up.12H
EXOC5		1.039									EN.Only.Up.12H
FARSB		1.023									EN.Only.Up.12H
FBXO40		2.074									EN.Only.Up.12H
FHL2		1.151									EN.Only.Up.12H
FLNB		1.104									EN.Only.Up.12H
GFM1		1.214									EN.Only.Up.12H
HK1		1.161									EN.Only.Up.12H
HMBS		1.057									EN.Only.Up.12H
HSPA4L		1.144									EN.Only.Up.12H
HSPB7		1.416									EN.Only.Up.12H
ID1		1.895									EN.Only.Up.12H
IGSF10		1.079									EN.Only.Up.12H
ISY1		1.083									EN.Only.Up.12H
KLHL18		1.198									EN.Only.Up.12H
LARGE		1.821									EN.Only.Up.12H
LOC475911		1.105									EN.Only.Up.12H
LOC476873		1.701									EN.Only.Up.12H
LOC478722		1.105									EN.Only.Up.12H
LOC480834		1.155									EN.Only.Up.12H
LOC480940		1.123									EN.Only.Up.12H
LOC481374		1.223									EN.Only.Up.12H
LOC486973		1.192									EN.Only.Up.12H
LOC607817		1.211									EN.Only.Up.12H
LOC609034		1.069									EN.Only.Up.12H
LOC609994		1.145									EN.Only.Up.12H
LOC610658		1.879									EN.Only.Up.12H
LRRC36		1.229									EN.Only.Up.12H
METTL11A		1.176									EN.Only.Up.12H
MTHFD2L		1.199									EN.Only.Up.12H
MYBBP1A		1.007									EN.Only.Up.12H
NOD2		1.144									EN.Only.Up.12H
NOM1		1.023									EN.Only.Up.12H
OSBPL11		1.013									EN.Only.Up.12H
PDE3B		1.939									EN.Only.Up.12H
PDGFA		1.152									EN.Only.Up.12H
PGAP2		1.375									EN.Only.Up.12H
PGPEP1		1.011									EN.Only.Up.12H
PLEKHA8		1.065									EN.Only.Up.12H
PNPT1		1.003									EN.Only.Up.12H
PPP4R1		1.211									EN.Only.Up.12H
PSMA2		1.204									EN.Only.Up.12H
PSMD11		1.547									EN.Only.Up.12H
PXN		1.059									EN.Only.Up.12H
RAB8B		1.067									EN.Only.Up.12H
RAD23B		1.025									EN.Only.Up.12H
RAE1		1.04									EN.Only.Up.12H
RUVBL2		1.15									EN.Only.Up.12H
SARS		1.295									EN.Only.Up.12H
SCML2		1.437									EN.Only.Up.12H
SLC16A3		1.333									EN.Only.Up.12H
SLC25A11		1.056									EN.Only.Up.12H
SLC25A37		1.173									EN.Only.Up.12H
SMARCA4		1.148									EN.Only.Up.12H
SNRNP40		1.428									EN.Only.Up.12H
SPAG9		1.241									EN.Only.Up.12H
SPRED2		1.473									EN.Only.Up.12H
TAF13		1.237									EN.Only.Up.12H
TALDO1		1.124									EN.Only.Up.12H
THAP1		1.387									EN.Only.Up.12H
TIPRL		1.376									EN.Only.Up.12H
TMEM156		1.367									EN.Only.Up.12H
VIPR1		1.185									EN.Only.Up.12H
ADD3			1.505								EN.Only.Up.24H
ANXA5			1.096								EN.Only.Up.24H
ATP2B1			1.228								EN.Only.Up.24H
CBX5			1.131								EN.Only.Up.24H
CIB2			1.274								EN.Only.Up.24H
CNPY3			1.49								EN.Only.Up.24H
COPG			1.314								EN.Only.Up.24H
cOR4S3			1.263								EN.Only.Up.24H
CTH			1.56								EN.Only.Up.24H
DCLRE1B			1.923								EN.Only.Up.24H
DDA1			1.019								EN.Only.Up.24H

**DDHD1**			2.276								EN.Only.Up.24H
**DENR**			1.177								EN.Only.Up.24H
**DFNA5**			1.606								EN.Only.Up.24H
**EXOSC1**			1.285								EN.Only.Up.24H
**EZR**			1.222								EN.Only.Up.24H
**F13A1**			3.609								EN.Only.Up.24H
**FCER1A**			1.912								EN.Only.Up.24H
**GK5**			1.182								EN.Only.Up.24H
**GNB1**			1.05								EN.Only.Up.24H
**GSS**			1.035								EN.Only.Up.24H
**HGF**			2.56								EN.Only.Up.24H
**HSF2**			1.808								EN.Only.Up.24H
**IRF5**			1.175								EN.Only.Up.24H
**ITGA2**			2.946								EN.Only.Up.24H
**JARID2**			1.422								EN.Only.Up.24H
**KCNRG**			2.16								EN.Only.Up.24H
**LIG4**			1.459								EN.Only.Up.24H
**LOC474690**			1.136								EN.Only.Up.24H
**LOC474869**			2.142								EN.Only.Up.24H
**LOC475725**			1.298								EN.Only.Up.24H
**LOC476908**			1.484								EN.Only.Up.24H
**LOC478620**			1.691								EN.Only.Up.24H
**LOC481569**			1.609								EN.Only.Up.24H
**LOC486627**			1.489								EN.Only.Up.24H
**LOC488965**			1.12								EN.Only.Up.24H
**LOC491323**			1.1								EN.Only.Up.24H
**LOC611683**			1.88								EN.Only.Up.24H
**LPIN2**			1.885								EN.Only.Up.24H
**MARCO**			1.404								EN.Only.Up.24H
**MCM7**			1.893								EN.Only.Up.24H
**ME2**			1.027								EN.Only.Up.24H
**MRPL20**			1.09								EN.Only.Up.24H
**MRPS14**			1.518								EN.Only.Up.24H
**NAP1L1**			1.072								EN.Only.Up.24H
**NDUFAB1**			1.408								EN.Only.Up.24H
**NUP107**			1.363								EN.Only.Up.24H
**OGFRL1**			1.269								EN.Only.Up.24H
**OLA1**			1.061								EN.Only.Up.24H
**OSBPL3**			1.932								EN.Only.Up.24H
**PCCA**			1.157								EN.Only.Up.24H
**PLDN**			1.827								EN.Only.Up.24H
**POLR2G**			1.418								EN.Only.Up.24H
**PPM1G**			1.229								EN.Only.Up.24H
**RAP1GDS1**			1.092								EN.Only.Up.24H
**RDH11**			1.162								EN.Only.Up.24H
**SENP8**			1.248								EN.Only.Up.24H
**SERAC1**			1.584								EN.Only.Up.24H
**SLC23A1**			1.372								EN.Only.Up.24H
**SLC25A16**			1.82								EN.Only.Up.24H
**SNAPC4**			1.229								EN.Only.Up.24H
**SPAST**			1.025								EN.Only.Up.24H
**SRD5A2**			1.268								EN.Only.Up.24H
**TATDN3**			1.626								EN.Only.Up.24H
**TBC1D14**			1.125								EN.Only.Up.24H
**TET2**			1.094								EN.Only.Up.24H
**TGS1**			1.147								EN.Only.Up.24H
**TMCC1**			1.532								EN.Only.Up.24H
**TMEM126B**			1.277								EN.Only.Up.24H
**TNFSF12-TNFSF13**			1.451								EN.Only.Up.24H
**TNFSF8**			1.434								EN.Only.Up.24H
**TRMT5**			1.689								EN.Only.Up.24H
**TYRO3**			1.07								EN.Only.Up.24H
**UBLCP1**			1.345								EN.Only.Up.24H
**UNC119B**			1.564								EN.Only.Up.24H
**UPK2**			2.407								EN.Only.Up.24H
**USO1**			1.447								EN.Only.Up.24H
**USP24**			1.174								EN.Only.Up.24H
**VRK1**			1.696								EN.Only.Up.24H
**ZSCAN20**			1.222								EN.Only.Up.24H
**ADSSL1**	1.399										EN.Only.Up.2H
**ALDH4A1**	1.63										EN.Only.Up.2H
**ARIH1**	1.443										EN.Only.Up.2H
**BHLHE40**	1.78										EN.Only.Up.2H
**CDR2**	1.627										EN.Only.Up.2H
**CSF2**	4.862										EN.Only.Up.2H
**ELL2**	2.407										EN.Only.Up.2H
**ETS2**	2.042										EN.Only.Up.2H
**GGNBP2**	2.144										EN.Only.Up.2H
**GNA13**	1.675										EN.Only.Up.2H
**INSIG1**	1.769										EN.Only.Up.2H
**LOC488214**	1.859										EN.Only.Up.2H
**LOC488486**	1.457										EN.Only.Up.2H
**LOC608512**	1.687										EN.Only.Up.2H
**LONRF1**	1.332										EN.Only.Up.2H
**LRRFIP2**	1.311										EN.Only.Up.2H
**PMP22**	1.432										EN.Only.Up.2H
**RBBP6**	1.437										EN.Only.Up.2H
**RGS16**	1.777										EN.Only.Up.2H
**RND1**	1.526										EN.Only.Up.2H
**RND3**	1.661										EN.Only.Up.2H
**SGMS2**	1.762										EN.Only.Up.2H
**TRA2A**	1.668										EN.Only.Up.2H
**TRAPPC9**	1.631										EN.Only.Up.2H
**TSC22D2**	2.333										EN.Only.Up.2H
**ZNF217**	1.363										EN.Only.Up.2H
**ZNF509**	1.64										EN.Only.Up.2H
**ZNF622**	1.558										EN.Only.Up.2H
**AACS**				1.493							EN.Only.Up.7D
**ABCA1**				1.169							EN.Only.Up.7D
**ALDH16A1**				2.012							EN.Only.Up.7D
**ALDH5A1**				1.178							EN.Only.Up.7D
**ALG9**				1.044							EN.Only.Up.7D
**ARD1A**				1.077							EN.Only.Up.7D
**ARHGAP23**				1.169							EN.Only.Up.7D
**ARSB**				2.75							EN.Only.Up.7D
**ATAD5**				1.598							EN.Only.Up.7D
**ATP6AP1**				1.462							EN.Only.Up.7D
**B4GALNT1**				1.438							EN.Only.Up.7D
**BCL10**				1.446							EN.Only.Up.7D
**BLVRB**				1.288							EN.Only.Up.7D
**BMP1**				1.609							EN.Only.Up.7D
**C1R**				2.733							EN.Only.Up.7D
**CALM3**				1.35							EN.Only.Up.7D
**CAMK2A**				2.062							EN.Only.Up.7D
**CCNB3**				1.052							EN.Only.Up.7D
**CCNE1**				1.976							EN.Only.Up.7D
**CD163**				1.712							EN.Only.Up.7D
**CD36**				4.462							EN.Only.Up.7D
**CDC25A**				1.614							EN.Only.Up.7D
**CDC45L**				1.197							EN.Only.Up.7D
**CDIPT**				1.214							EN.Only.Up.7D
**CDT1**				1.276							EN.Only.Up.7D
**CHEK1**				1.525							EN.Only.Up.7D

CLCN5				1.161							EN.Only.Up.7D
CRYBG3				1.056							EN.Only.Up.7D
CTSD				1.682							EN.Only.Up.7D
CTSZ				1.807							EN.Only.Up.7D
CYP19				1.406							EN.Only.Up.7D
DAP				1.225							EN.Only.Up.7D
DIABLO				1.041							EN.Only.Up.7D
DIP2B				1.104							EN.Only.Up.7D
ENG				1.189							EN.Only.Up.7D
ENO1				1.149							EN.Only.Up.7D
F11				1.931							EN.Only.Up.7D
FEZ2				1.01							EN.Only.Up.7D
FKBP10				2.209							EN.Only.Up.7D
FLRT2				1.751							EN.Only.Up.7D
FTL				1.968							EN.Only.Up.7D
GIMAP8				1.017							EN.Only.Up.7D
GLT25D1				1.632							EN.Only.Up.7D
GPNMB				4.376							EN.Only.Up.7D
GRN				1.138							EN.Only.Up.7D
GSR				1.234							EN.Only.Up.7D
GUSB				1.518							EN.Only.Up.7D
HERC2				1.466							EN.Only.Up.7D
HMOX1				1.011							EN.Only.Up.7D
KCNA3				1.196							EN.Only.Up.7D
KIF2C				1.99							EN.Only.Up.7D
KLHL5				1.023							EN.Only.Up.7D
KYNU				1.079							EN.Only.Up.7D
LGALS3BP				1.126							EN.Only.Up.7D
LGMN				1.235							EN.Only.Up.7D
LMLN				1.07							EN.Only.Up.7D
LOC474990				1.668							EN.Only.Up.7D
LOC478556				1.272							EN.Only.Up.7D
LOC479329				2.356							EN.Only.Up.7D
LOC480000				1.113							EN.Only.Up.7D
LOC480764				1.181							EN.Only.Up.7D
LOC482035				1.274							EN.Only.Up.7D
LOC482284				1.673							EN.Only.Up.7D
LOC482624				3.531							EN.Only.Up.7D
LOC482707				1.142							EN.Only.Up.7D
LOC483168				1.281							EN.Only.Up.7D
LOC483279				1.786							EN.Only.Up.7D
LOC491671				1.018							EN.Only.Up.7D
LOC606842				1.173							EN.Only.Up.7D
LOC607130				1.258							EN.Only.Up.7D
LOC607158				1.237							EN.Only.Up.7D
LOC607448				3.981							EN.Only.Up.7D
LOC607800				1.034							EN.Only.Up.7D
LOC607937				1.289							EN.Only.Up.7D
LOC608066				1.101							EN.Only.Up.7D
LOC608500				1.692							EN.Only.Up.7D
LRP1				1.334							EN.Only.Up.7D
LRTOMT				1.197							EN.Only.Up.7D
M6PR				1.016							EN.Only.Up.7D
MAN2B1				1.099							EN.Only.Up.7D
MYBL2				1.384							EN.Only.Up.7D
MYO1D				1.189							EN.Only.Up.7D
MYO5A				1.129							EN.Only.Up.7D
N6AMT2				1.086							EN.Only.Up.7D
NAGLU				1.435							EN.Only.Up.7D
NMRAL1				1.21							EN.Only.Up.7D
NR5A2				1.069							EN.Only.Up.7D
OAS1				3.502							EN.Only.Up.7D
PACS2				1.002							EN.Only.Up.7D
PCOLCE				1.781							EN.Only.Up.7D
PLCL2				1.178							EN.Only.Up.7D
PLVAP				2.03							EN.Only.Up.7D
PPM1J				1.033							EN.Only.Up.7D
PROM1				2.06							EN.Only.Up.7D
PRRG1				1.133							EN.Only.Up.7D
PTGR1				1.295							EN.Only.Up.7D
PYCARD				1.106							EN.Only.Up.7D
RAB6B				1.038							EN.Only.Up.7D
RAD54L				1.753							EN.Only.Up.7D
RGS1				1.238							EN.Only.Up.7D
RNASET2				1.271							EN.Only.Up.7D
SCD				1.322							EN.Only.Up.7D
SEC11C				1.188							EN.Only.Up.7D
SEC16A				1.558							EN.Only.Up.7D
SERPINB1				1.309							EN.Only.Up.7D
SFXN1				1.558							EN.Only.Up.7D
SLC25A39				1.232							EN.Only.Up.7D
SLC4A11				1.419							EN.Only.Up.7D
SLCO2A1				1.954							EN.Only.Up.7D
SMG1				1.325							EN.Only.Up.7D
SNX6				1.202							EN.Only.Up.7D
STAB1				1.687							EN.Only.Up.7D
STK11IP				1.263							EN.Only.Up.7D
SYN3				1.743							EN.Only.Up.7D
TNFRSF11B				1.518							EN.Only.Up.7D
TNNT2				1.044							EN.Only.Up.7D
TREML1				3.343							EN.Only.Up.7D
TTYH3				1.703							EN.Only.Up.7D
YKT6				1.057							EN.Only.Up.7D
LOC477487							1.276				SM.Only.Down.12H, SM.Only.Up.12H
3-Mar							1.241				SM.Only.Up.12H
ABT1							1.462				SM.Only.Up.12H
ACTR1A							1.119				SM.Only.Up.12H
ACVRL1							1.439				SM.Only.Up.12H
ADAM9							1.491				SM.Only.Up.12H
ADCY4							1.46				SM.Only.Up.12H
AGFG1							1.105				SM.Only.Up.12H
AKIRIN2							1.037				SM.Only.Up.12H
ALAS1							1.694				SM.Only.Up.12H
ALG8							1.011				SM.Only.Up.12H
ALX1							1.998				SM.Only.Up.12H
ANGPTL4							1.852				SM.Only.Up.12H
ANKHD1							1.013				SM.Only.Up.12H
ANUBL1							1.023				SM.Only.Up.12H
ANXA1							1.17				SM.Only.Up.12H
AP2B1							1.086				SM.Only.Up.12H
AP2S1							1.025				SM.Only.Up.12H
APEX1							1.011				SM.Only.Up.12H
APITD1							1.546				SM.Only.Up.12H
ARAP3							1.213				SM.Only.Up.12H
ARNTL							1.283				SM.Only.Up.12H
ATF1							1.04				SM.Only.Up.12H
ATG3							1.205				SM.Only.Up.12H
AVIL							1.141				SM.Only.Up.12H
B4GALT5							2.362				SM.Only.Up.12H
BAT1							1.021				SM.Only.Up.12H
BATF3							1.263				SM.Only.Up.12H
BCCIP							1.215				SM.Only.Up.12H
BEST1							2.2				SM.Only.Up.12H

BET1							1.014				SM.Only.Up.12H
BFGF							1.257				SM.Only.Up.12H
BIRC2							1.044				SM.Only.Up.12H
BLOC1S2							1.755				SM.Only.Up.12H
BMS1							1.008				SM.Only.Up.12H
BMX							1.558				SM.Only.Up.12H
BOD1							1.093				SM.Only.Up.12H
BRD2							1.067				SM.Only.Up.12H
BTBD10							1.062				SM.Only.Up.12H
C1QBP							1.18				SM.Only.Up.12H
C2CD2L							1.06				SM.Only.Up.12H
CABYR							1.474				SM.Only.Up.12H
CAMK1D							1.061				SM.Only.Up.12H
CCDC117							1.098				SM.Only.Up.12H
CCL20							2.178				SM.Only.Up.12H
CCNC							1.023				SM.Only.Up.12H
CCT3							1.397				SM.Only.Up.12H
CDK4							1.219				SM.Only.Up.12H
CEACAM1							1.143				SM.Only.Up.12H
CEACAM7							1.167				SM.Only.Up.12H
CFLAR							1.304				SM.Only.Up.12H
CHCHD1							1.034				SM.Only.Up.12H
CHD1L							1.222				SM.Only.Up.12H
CHPT1							1.328				SM.Only.Up.12H
CHST11							1.569				SM.Only.Up.12H
CISD2							1.182				SM.Only.Up.12H
CLEC12B							1.773				SM.Only.Up.12H
CNOT6							1.278				SM.Only.Up.12H
CNTNAP5							1.121				SM.Only.Up.12H
cOR51H3							1.476				SM.Only.Up.12H
cOR9A7							1.872				SM.Only.Up.12H
CR2							1.34				SM.Only.Up.12H
CSTF2							1.106				SM.Only.Up.12H
CUTC							1.054				SM.Only.Up.12H
CXCL12							1.016				SM.Only.Up.12H
CYFIP2							1.151				SM.Only.Up.12H
CYTH1							1.199				SM.Only.Up.12H
DAPP1							1.164				SM.Only.Up.12H
DCAF13							1.17				SM.Only.Up.12H
DDAH1							1.335				SM.Only.Up.12H
DDX27							1.191				SM.Only.Up.12H
DDX3X							1.318				SM.Only.Up.12H
DDX49							1.363				SM.Only.Up.12H
DEDD2							1.235				SM.Only.Up.12H
DENND4A							1.293				SM.Only.Up.12H
DHODH							1.015				SM.Only.Up.12H
DHRS9							1.176				SM.Only.Up.12H
DLA-12							1.054				SM.Only.Up.12H
DNER							1.203				SM.Only.Up.12H
DOC2B							1.237				SM.Only.Up.12H
DRAM1							1.78				SM.Only.Up.12H
DRAP1							1.051				SM.Only.Up.12H
DUS1L							1.18				SM.Only.Up.12H
DUSP1							1.155				SM.Only.Up.12H
DUSP6							1.361				SM.Only.Up.12H
E2F5							2.372				SM.Only.Up.12H
EDARADD							1.252				SM.Only.Up.12H
EHD4							1.203				SM.Only.Up.12H
EIF3J							1.135				SM.Only.Up.12H
ELF1							1.128				SM.Only.Up.12H
ELF2							1.115				SM.Only.Up.12H
ELL							1.144				SM.Only.Up.12H
ELMOD3							1.001				SM.Only.Up.12H
ELP2							1.001				SM.Only.Up.12H
EMG1							1.339				SM.Only.Up.12H
EXOSC4							1.608				SM.Only.Up.12H
FADS1							1.343				SM.Only.Up.12H
FASTKD2							1.103				SM.Only.Up.12H
FBLN5							1.032				SM.Only.Up.12H
FICD							1.273				SM.Only.Up.12H
FLOT1							1.614				SM.Only.Up.12H
FNIP2							1.033				SM.Only.Up.12H
FOXS1							1.148				SM.Only.Up.12H
FRMD6							1.313				SM.Only.Up.12H
G6PD							2.139				SM.Only.Up.12H
GAR1							1.122				SM.Only.Up.12H
GARNL3							1.351				SM.Only.Up.12H
GCAT							1.406				SM.Only.Up.12H
GEMIN5							1.317				SM.Only.Up.12H
GLIS3							1.025				SM.Only.Up.12H
GLUL							1.17				SM.Only.Up.12H
GMEB1							1.12				SM.Only.Up.12H
GNAT2							1.005				SM.Only.Up.12H
GNL2							1.048				SM.Only.Up.12H
GPN2							1.332				SM.Only.Up.12H
GPR97							1.435				SM.Only.Up.12H
GREM1							1.684				SM.Only.Up.12H
GRINA							1.161				SM.Only.Up.12H
GRK5							1.075				SM.Only.Up.12H
GRPEL2							1.147				SM.Only.Up.12H
GSPT1							1.15				SM.Only.Up.12H
HCFC2							1.809				SM.Only.Up.12H
HDHD2							1.109				SM.Only.Up.12H
HIF1A							1.014				SM.Only.Up.12H
HK2							1.448				SM.Only.Up.12H
HNRNPH1							1.028				SM.Only.Up.12H
HNRNPU							1.159				SM.Only.Up.12H
HSD11B1							1.262				SM.Only.Up.12H
HUS1							1.216				SM.Only.Up.12H
IARS							1.164				SM.Only.Up.12H
IFNGR1							1.103				SM.Only.Up.12H
IGF2BP3							1.113				SM.Only.Up.12H
IL17RA							1.214				SM.Only.Up.12H
ILF2							1.309				SM.Only.Up.12H
IMMT							1.455				SM.Only.Up.12H
IMPDH2							1.092				SM.Only.Up.12H
IRF1							1.302				SM.Only.Up.12H
ITGB3							2.176				SM.Only.Up.12H
JAGN1							1.339				SM.Only.Up.12H
JMJD6							1.582				SM.Only.Up.12H
JPH1							1.013				SM.Only.Up.12H
KBTBD7							1.095				SM.Only.Up.12H
KCTD5							1.037				SM.Only.Up.12H
KLC1							1.356				SM.Only.Up.12H
KLF10							1.078				SM.Only.Up.12H
KLF5							1.657				SM.Only.Up.12H
KLHDC3							1.152				SM.Only.Up.12H
KPNA6							1.214				SM.Only.Up.12H
LAMB3							1.38				SM.Only.Up.12H
LARS							1.013				SM.Only.Up.12H
LETM1							1.009				SM.Only.Up.12H
LMBR1L							1.801				SM.Only.Up.12H
LMNB2							1.023				SM.Only.Up.12H

**LOC474535**							1.96				SM.Only.Up.12H
**LOC474540**							1.001				SM.Only.Up.12H
**LOC474543**							1.139				SM.Only.Up.12H
**LOC474769**							1.041				SM.Only.Up.12H
**LOC474919**							1.829				SM.Only.Up.12H
**LOC475553**							1.029				SM.Only.Up.12H
**LOC476056**							1.264				SM.Only.Up.12H
**LOC476149**							1.634				SM.Only.Up.12H
**LOC477038**							1.011				SM.Only.Up.12H
**LOC477532**							1.164				SM.Only.Up.12H
**LOC478087**							1.377				SM.Only.Up.12H
**LOC478495**							1.18				SM.Only.Up.12H
**LOC478694**							1.142				SM.Only.Up.12H
**LOC478865**							1.151				SM.Only.Up.12H
**LOC479205**							1.009				SM.Only.Up.12H
**LOC479365**							1.356				SM.Only.Up.12H
**LOC480535**							2.27				SM.Only.Up.12H
**LOC482293**							1.003				SM.Only.Up.12H
**LOC482428**							1.313				SM.Only.Up.12H
**LOC482756**							1.462				SM.Only.Up.12H
**LOC484461**							1.068				SM.Only.Up.12H
**LOC485831**							1.223				SM.Only.Up.12H
**LOC485858**							1.532				SM.Only.Up.12H
**LOC486214**							1.664				SM.Only.Up.12H
**LOC488631**							1.098				SM.Only.Up.12H
**LOC490690**							1.041				SM.Only.Up.12H
**LOC490810**							1.105				SM.Only.Up.12H
**LOC492219**							1.169				SM.Only.Up.12H
**LOC607369**							1.082				SM.Only.Up.12H
**LOC607690**							1.162				SM.Only.Up.12H
**LOC607889**							1.084				SM.Only.Up.12H
**LOC607911**							1.015				SM.Only.Up.12H
**LOC607957**							1.319				SM.Only.Up.12H
**LOC609286**							1.254				SM.Only.Up.12H
**LOC609339**							1.853				SM.Only.Up.12H
**LOC609365**							1.25				SM.Only.Up.12H
**LOC609534**							1.053				SM.Only.Up.12H
**LOC609677**							1.324				SM.Only.Up.12H
**LOC610012**							1.116				SM.Only.Up.12H
**LOC610626**							1.03				SM.Only.Up.12H
**LOC610942**							1.081				SM.Only.Up.12H
**LOC611900**							1.247				SM.Only.Up.12H
**LOC612537**							1.3				SM.Only.Up.12H
**LRG1**							1.175				SM.Only.Up.12H
**LRRC8B**							1.376				SM.Only.Up.12H
**LRRCC1**							1.037				SM.Only.Up.12H
**LSM1**							1.096				SM.Only.Up.12H
**LSM2**							1.352				SM.Only.Up.12H
**LSM7**							1.573				SM.Only.Up.12H
**LTBP1**							1.409				SM.Only.Up.12H
**LY75**							1.302				SM.Only.Up.12H
**LYG1**							1.016				SM.Only.Up.12H
**MAD2L1BP**							1.035				SM.Only.Up.12H
**MAGMAS**							1.006				SM.Only.Up.12H
**MAMLD1**							1.082				SM.Only.Up.12H
**MAN1C1**							1.188				SM.Only.Up.12H
**MAPK13**							1.972				SM.Only.Up.12H
**MARK2**							1.174				SM.Only.Up.12H
**MDN1**							1.067				SM.Only.Up.12H
**METT10D**							1.516				SM.Only.Up.12H
**METTL3**							1.18				SM.Only.Up.12H
**MIDN**							1.427				SM.Only.Up.12H
**MLEC**							1.147				SM.Only.Up.12H
**MOCOS**							1.133				SM.Only.Up.12H
**MPHOSPH6**							1.024				SM.Only.Up.12H
**MRPS22**							1.279				SM.Only.Up.12H
**MRPS30**							1.251				SM.Only.Up.12H
**NAP1L4**							1.073				SM.Only.Up.12H
**NARS2**							1.105				SM.Only.Up.12H
**NAT13**							1.156				SM.Only.Up.12H
**NECAP2**							1.086				SM.Only.Up.12H
**NETO2**							1.115				SM.Only.Up.12H
**NFAM1**							1.351				SM.Only.Up.12H
**NFKB2**							1.644				SM.Only.Up.12H
**NFKBIA**							1.509				SM.Only.Up.12H
**NFKBIB**							1.335				SM.Only.Up.12H
**NFYA**							1.204				SM.Only.Up.12H
**NGF**							1.268				SM.Only.Up.12H
**NIACR1**							1.789				SM.Only.Up.12H
**NIP7**							1.062				SM.Only.Up.12H
**NIPA2**							1.182				SM.Only.Up.12H
**NOL10**							1.175				SM.Only.Up.12H
**NOLC1**							1.122				SM.Only.Up.12H
**NOP16**							1.244				SM.Only.Up.12H
**NOVA1**							1.015				SM.Only.Up.12H
**NP**							1.188				SM.Only.Up.12H
**NQO1**							1.259				SM.Only.Up.12H
**NT5C3**							1.038				SM.Only.Up.12H
**NUDT5**							1.024				SM.Only.Up.12H
**NUDT6**							1.346				SM.Only.Up.12H
**NUP210L**							1.509				SM.Only.Up.12H
**OPN1SW**							1.228				SM.Only.Up.12H
**OVCA2**							1.048				SM.Only.Up.12H
**PA2G4**							1.005				SM.Only.Up.12H
**PAK2**							1.188				SM.Only.Up.12H
**PDE12**							1.265				SM.Only.Up.12H
**PDXK**							1.045				SM.Only.Up.12H
**PFDN2**							1.126				SM.Only.Up.12H
**PHACTR2**							1.397				SM.Only.Up.12H
**PI15**							1.147				SM.Only.Up.12H
**PIGV**							1.031				SM.Only.Up.12H
**PION**							1.12				SM.Only.Up.12H
**PIP5K1A**							1.297				SM.Only.Up.12H
**PIR**							1.053				SM.Only.Up.12H
**PITRM1**							1.267				SM.Only.Up.12H
**PLA2G4E**							1.429				SM.Only.Up.12H
**PLEKHG6**							1.302				SM.Only.Up.12H
**PMPCA**							1.038				SM.Only.Up.12H
**POLR2H**							1.017				SM.Only.Up.12H
**POLR3K**							1.57				SM.Only.Up.12H
**POMP**							1.039				SM.Only.Up.12H
**PPIL3**							1.166				SM.Only.Up.12H
**PPP2R3C**							1.081				SM.Only.Up.12H
**PPP3CB**							1.618				SM.Only.Up.12H
**PPPDE2**							1.784				SM.Only.Up.12H
**PPRC1**							1.7				SM.Only.Up.12H
**PREB**							1.507				SM.Only.Up.12H
**PRKAR1A**							1.806				SM.Only.Up.12H
**PRPS1**							1.055				SM.Only.Up.12H
**PSEN1**							1.132				SM.Only.Up.12H
**PSMB1**							1.005				SM.Only.Up.12H
**PSMB8**							1.355				SM.Only.Up.12H
**PSMD13**							1.12				SM.Only.Up.12H

PSMD8							1.259				SM.Only.Up.12H
PSME2							1.123				SM.Only.Up.12H
PSME3							1.493				SM.Only.Up.12H
PTGER3							1.326				SM.Only.Up.12H
PTGIS							1.157				SM.Only.Up.12H
PTPN9							1.27				SM.Only.Up.12H
PUS1							1.292				SM.Only.Up.12H
QTRT1							1.104				SM.Only.Up.12H
RAB11FIP1							1.565				SM.Only.Up.12H
RAB11FIP4							1.127				SM.Only.Up.12H
RAB18							1.108				SM.Only.Up.12H
RALBP1							1.114				SM.Only.Up.12H
RANBP1							1.708				SM.Only.Up.12H
RASGRP4							1.286				SM.Only.Up.12H
RBM14							1.355				SM.Only.Up.12H
RBM19							1.207				SM.Only.Up.12H
RBM23							1.185				SM.Only.Up.12H
RBM8A							1.13				SM.Only.Up.12H
RCL1							1.345				SM.Only.Up.12H
RFXANK							1.755				SM.Only.Up.12H
RGS4							2.152				SM.Only.Up.12H
RHAG							1.073				SM.Only.Up.12H
RNF114							1.438				SM.Only.Up.12H
RNF145							1.084				SM.Only.Up.12H
RNF185							1.903				SM.Only.Up.12H
RNF25							1.461				SM.Only.Up.12H
ROGDI							1.012				SM.Only.Up.12H
ROMO1							1.269				SM.Only.Up.12H
RPAP3							1.007				SM.Only.Up.12H
RPIA							1.152				SM.Only.Up.12H
RPL10A							1.719				SM.Only.Up.12H
RPL32							1.553				SM.Only.Up.12H
RPL7L1							1.087				SM.Only.Up.12H
RPP14							1.027				SM.Only.Up.12H
RPS12							1.433				SM.Only.Up.12H
RPS19							1.111				SM.Only.Up.12H
RPS19BP1							1.161				SM.Only.Up.12H
RRP7A							1.737				SM.Only.Up.12H
SAG							1.906				SM.Only.Up.12H
SART3							1.139				SM.Only.Up.12H
SEC22B							1.095				SM.Only.Up.12H
SFRS7							1.172				SM.Only.Up.12H
SHB							1.737				SM.Only.Up.12H
SHROOM4							1.428				SM.Only.Up.12H
SLC16A7							1.132				SM.Only.Up.12H
SLC18A2							1.025				SM.Only.Up.12H
SLC1A5							1.037				SM.Only.Up.12H
SLC25A33							1.146				SM.Only.Up.12H
SLC26A8							1.549				SM.Only.Up.12H
SLC30A7							1.245				SM.Only.Up.12H
SLC36A4							1.006				SM.Only.Up.12H
SLC37A3							1.267				SM.Only.Up.12H
SLC38A5							1.574				SM.Only.Up.12H
SLC6A14							2.331				SM.Only.Up.12H
SLC9A6							1.403				SM.Only.Up.12H
SMN							1.092				SM.Only.Up.12H
SMYD5							1.209				SM.Only.Up.12H
SNRPC							1.351				SM.Only.Up.12H
SNRPD2							1.036				SM.Only.Up.12H
SOD2							1.807				SM.Only.Up.12H
SPAG17							1.414				SM.Only.Up.12H
SPATA2							1.427				SM.Only.Up.12H
SPP2							1.659				SM.Only.Up.12H
SPTLC2							1.289				SM.Only.Up.12H
SRFBP1							1.088				SM.Only.Up.12H
SRPR							1.203				SM.Only.Up.12H
SRR							1.204				SM.Only.Up.12H
SRRT							1.006				SM.Only.Up.12H
STARD3							1.047				SM.Only.Up.12H
STX11							1.165				SM.Only.Up.12H
STX12							1.118				SM.Only.Up.12H
SUPV3L1							1.104				SM.Only.Up.12H
SYCP2							1.485				SM.Only.Up.12H
SYNCRIP							1.069				SM.Only.Up.12H
SYT17							1.073				SM.Only.Up.12H
TAF1D							1.03				SM.Only.Up.12H
TAF4B							1.003				SM.Only.Up.12H
TAGLN2							1.258				SM.Only.Up.12H
TBC1D13							1.191				SM.Only.Up.12H
TBC1D9							1.04				SM.Only.Up.12H
TCEB1							1.085				SM.Only.Up.12H
TCEB3							1.173				SM.Only.Up.12H
TEAD4							1.825				SM.Only.Up.12H
TEX28							1.729				SM.Only.Up.12H
THAP5							1.479				SM.Only.Up.12H
TIMM17A							1.333				SM.Only.Up.12H
TIMM50							1				SM.Only.Up.12H
TKT							1.44				SM.Only.Up.12H
TMBIM1							1.106				SM.Only.Up.12H
TMEM167A							1.002				SM.Only.Up.12H
TMEM30C							1.24				SM.Only.Up.12H
TMEM33							1.275				SM.Only.Up.12H
TNFRSF21							1.122				SM.Only.Up.12H
TNFRSF25							1.13				SM.Only.Up.12H
TOMM40							1.143				SM.Only.Up.12H
TOMM5							1.251				SM.Only.Up.12H
TOP1							1.445				SM.Only.Up.12H
TPST2							1.488				SM.Only.Up.12H
TRIM26							1.35				SM.Only.Up.12H
TRIOBP							1.788				SM.Only.Up.12H
TRMT112							1.057				SM.Only.Up.12H
TRMT12							1.082				SM.Only.Up.12H
TSHZ2							1.503				SM.Only.Up.12H
TSPAN15							1.523				SM.Only.Up.12H
TWISTNB							1.29				SM.Only.Up.12H
TXNDC9							1.429				SM.Only.Up.12H
UBA2							1.351				SM.Only.Up.12H
UBE2N							1.107				SM.Only.Up.12H
UBR2							1.344				SM.Only.Up.12H
UCHL3							1.007				SM.Only.Up.12H
UFM1							1.042				SM.Only.Up.12H
UGCG							1.56				SM.Only.Up.12H
UMPS							1.274				SM.Only.Up.12H
USP32							1.024				SM.Only.Up.12H
USP38							1.04				SM.Only.Up.12H
USP48							1.008				SM.Only.Up.12H
UTP14A							1.371				SM.Only.Up.12H
UTP15							1.294				SM.Only.Up.12H
UTP20							1.13				SM.Only.Up.12H
UTP3							1.205				SM.Only.Up.12H
WDR24							1.162				SM.Only.Up.12H
XRCC5							1.168				SM.Only.Up.12H
YEATS2							1.043				SM.Only.Up.12H

YWHAG							1.137				SM.Only.Up.12H
ZC3H12C							1.059				SM.Only.Up.12H
ZDHHC23							1.232				SM.Only.Up.12H
ZDHHC9							1.056				SM.Only.Up.12H
ZFAND5							1.014				SM.Only.Up.12H
ZFHX2							1.208				SM.Only.Up.12H
ZNF41							1.445				SM.Only.Up.12H
ZNF462							1.223				SM.Only.Up.12H
ZNF684							1.033				SM.Only.Up.12H
ZNHIT6							1.402				SM.Only.Up.12H
ZSWIM6							1.008				SM.Only.Up.12H
ZZZ3							1.069				SM.Only.Up.12H
AIF1L								1.236			SM.Only.Up.24H
ARL6IP6								1.507			SM.Only.Up.24H
CEP76								2.278			SM.Only.Up.24H
DET1								1.405			SM.Only.Up.24H
EDC3								1.469			SM.Only.Up.24H
ELOVL6								1.686			SM.Only.Up.24H
FLOT2								1.776			SM.Only.Up.24H
LOC611949								1.592			SM.Only.Up.24H
LRP8								1.387			SM.Only.Up.24H
MAEL								1.469			SM.Only.Up.24H
MRPS33								1.483			SM.Only.Up.24H
PKIA								2.048			SM.Only.Up.24H
POP5								1.596			SM.Only.Up.24H
RIMS1								1.689			SM.Only.Up.24H
SRGAP1								2.165			SM.Only.Up.24H
SYT16								1.636			SM.Only.Up.24H
USP50								1.582			SM.Only.Up.24H
XPO4								2.378			SM.Only.Up.24H
ABRA						2.859					SM.Only.Up.2H
LMCD1						2.419					SM.Only.Up.2H
LOC481896						1.82					SM.Only.Up.2H
SAMD4A						1.397					SM.Only.Up.2H
15-Sep									1.142		SM.Only.Up.7D
ABHD3									1.38		SM.Only.Up.7D
ACTN1									1.222		SM.Only.Up.7D
ADAMTS12									1.87		SM.Only.Up.7D
ADCY7									1.254		SM.Only.Up.7D
ADIPOR2									1.118		SM.Only.Up.7D
AP3B1									1.053		SM.Only.Up.7D
ARL1									1.042		SM.Only.Up.7D
ARL6IP5									1.056		SM.Only.Up.7D
ARPC1A									1.05		SM.Only.Up.7D
ATP6V1H									1.049		SM.Only.Up.7D
AUTS2									1.183		SM.Only.Up.7D
B3GALTL									1.105		SM.Only.Up.7D
B4GALT1									1.202		SM.Only.Up.7D
BACE2									1.411		SM.Only.Up.7D
BARD1									1.137		SM.Only.Up.7D
BCL2L11									1.326		SM.Only.Up.7D
BGN									1.317		SM.Only.Up.7D
BICC1									1.449		SM.Only.Up.7D
BID									1.105		SM.Only.Up.7D
BTG3									1.036		SM.Only.Up.7D
C1QTNF3									3.241		SM.Only.Up.7D
CALU									1.277		SM.Only.Up.7D
CASP3									1.24		SM.Only.Up.7D
CCDC109B									1.742		SM.Only.Up.7D
CCDC8									1.456		SM.Only.Up.7D
CCM2									1.466		SM.Only.Up.7D
CCNG1									1.091		SM.Only.Up.7D
CDCA7L									1.327		SM.Only.Up.7D
CDH11									1.909		SM.Only.Up.7D
CENPC1									1.131		SM.Only.Up.7D
CEP350									1.064		SM.Only.Up.7D
CERCAM									1.791		SM.Only.Up.7D
CHD4									1.074		SM.Only.Up.7D
CHFR									1.544		SM.Only.Up.7D
CLIC2									2.321		SM.Only.Up.7D
cln5									1.002		SM.Only.Up.7D
CLU									1.839		SM.Only.Up.7D
COG5									1.044		SM.Only.Up.7D
COL10A1									1.467		SM.Only.Up.7D
COL12A1									1.992		SM.Only.Up.7D
COPA									1.047		SM.Only.Up.7D
COPB1									1.042		SM.Only.Up.7D
COPB2									1.295		SM.Only.Up.7D
cOR9K3									1.1		SM.Only.Up.7D
CPD									1.107		SM.Only.Up.7D
CPNE8									1.743		SM.Only.Up.7D
CPT2									1.15		SM.Only.Up.7D
CPZ									1.211		SM.Only.Up.7D
CREB3L1									1.841		SM.Only.Up.7D
CSPP1									1.048		SM.Only.Up.7D
DEGS1									1.055		SM.Only.Up.7D
DERL2									1.1		SM.Only.Up.7D
DIS3L									1.061		SM.Only.Up.7D
DNAJC10									1.027		SM.Only.Up.7D
DNAJC15									1.04		SM.Only.Up.7D
DNMT1									1.089		SM.Only.Up.7D
DOCK4									1.214		SM.Only.Up.7D
DRAM2									1.082		SM.Only.Up.7D
DTL									2.408		SM.Only.Up.7D
DTX3L									1.241		SM.Only.Up.7D
EDIL3									2.031		SM.Only.Up.7D
EFCAB10									1.201		SM.Only.Up.7D
EIF2AK2									1.238		SM.Only.Up.7D
ENPP1									2.19		SM.Only.Up.7D
ERBB2IP									1.11		SM.Only.Up.7D
ETV1									1.109		SM.Only.Up.7D
EXT2									1.234		SM.Only.Up.7D
FANCD2									2.106		SM.Only.Up.7D
FASTKD3									1.356		SM.Only.Up.7D
FBXO18									1.079		SM.Only.Up.7D
FGFR1OP									1.153		SM.Only.Up.7D
FIBCD1									1.113		SM.Only.Up.7D
FKBP1A									1.017		SM.Only.Up.7D
FKBP3									1.076		SM.Only.Up.7D
FMNL3									1.133		SM.Only.Up.7D
FNBP1L									1.282		SM.Only.Up.7D
FUCA1									1.731		SM.Only.Up.7D
FUT11									1.303		SM.Only.Up.7D
GALNT7									1.063		SM.Only.Up.7D
GGCX									1.264		SM.Only.Up.7D
GMNN									1.172		SM.Only.Up.7D
GNMT									1.134		SM.Only.Up.7D
GOLM1									1.17		SM.Only.Up.7D
GOLT1B									1.01		SM.Only.Up.7D
GPR180									1.148		SM.Only.Up.7D
HEATR5A									1.277		SM.Only.Up.7D
HERC5									3.2		SM.Only.Up.7D
HHAT									1.398		SM.Only.Up.7D

HMGCS1									1.081		SM.Only.Up.7D
HTR2B									1.609		SM.Only.Up.7D
HYAL1									1.877		SM.Only.Up.7D
IAH1									1.27		SM.Only.Up.7D
ICMT									1.754		SM.Only.Up.7D
IFIT1									3.238		SM.Only.Up.7D
IFT74									1.095		SM.Only.Up.7D
IFT80									1.685		SM.Only.Up.7D
IRAK4									1.457		SM.Only.Up.7D
IREB2									1.009		SM.Only.Up.7D
IRF9									1.578		SM.Only.Up.7D
ITGA6									1.217		SM.Only.Up.7D
K-RAS									1.138		SM.Only.Up.7D
KRCC1									1.263		SM.Only.Up.7D
KRT7									1.318		SM.Only.Up.7D
LEPREL2									1.384		SM.Only.Up.7D
LOC474866									1.289		SM.Only.Up.7D
LOC475374									1.44		SM.Only.Up.7D
LOC475708									3.089		SM.Only.Up.7D
LOC476202									1.198		SM.Only.Up.7D
LOC477808									1.413		SM.Only.Up.7D
LOC478059									1.112		SM.Only.Up.7D
LOC478554									1.486		SM.Only.Up.7D
LOC478738									1.149		SM.Only.Up.7D
LOC478809									1.076		SM.Only.Up.7D
LOC479057									1.251		SM.Only.Up.7D
LOC479179									1.093		SM.Only.Up.7D
LOC479283									1.06		SM.Only.Up.7D
LOC479636									1.077		SM.Only.Up.7D
LOC479777									1.154		SM.Only.Up.7D
LOC480336									1.94		SM.Only.Up.7D
LOC480340									1.565		SM.Only.Up.7D
LOC480791									1.141		SM.Only.Up.7D
LOC481273									1.11		SM.Only.Up.7D
LOC482619									1.22		SM.Only.Up.7D
LOC484759									1.149		SM.Only.Up.7D
LOC487760									1.48		SM.Only.Up.7D
LOC489294									1.21		SM.Only.Up.7D
LOC492149									1.003		SM.Only.Up.7D
LOC607009									1.232		SM.Only.Up.7D
LOC607494									1.208		SM.Only.Up.7D
LOC608303									1.848		SM.Only.Up.7D
LOC608438									2.756		SM.Only.Up.7D
LOC608808									1.007		SM.Only.Up.7D
LOC608999									1.04		SM.Only.Up.7D
LOC609430									1.099		SM.Only.Up.7D
LOC609700									2.376		SM.Only.Up.7D
LOC610209									1.047		SM.Only.Up.7D
LOC610238									1.322		SM.Only.Up.7D
LOC610249									1.051		SM.Only.Up.7D
LOC610815									2.699		SM.Only.Up.7D
LOC611775									1.909		SM.Only.Up.7D
LOC612621									2.096		SM.Only.Up.7D
LOC612917									2.201		SM.Only.Up.7D
LOC613008									1.088		SM.Only.Up.7D
LRRTM2									1.105		SM.Only.Up.7D
MAGED1									1.576		SM.Only.Up.7D
MAGED2									1.132		SM.Only.Up.7D
MALT1									1.357		SM.Only.Up.7D
MANBA									2.011		SM.Only.Up.7D
MDM1									1.065		SM.Only.Up.7D
METTL2B									1.113		SM.Only.Up.7D
MFSD5									1.065		SM.Only.Up.7D
MGAT2									1.331		SM.Only.Up.7D
MGAT5									1.264		SM.Only.Up.7D
MPP4									1.981		SM.Only.Up.7D
MRAP2									1.802		SM.Only.Up.7D
MVP									1.345		SM.Only.Up.7D
MYCBP									1.048		SM.Only.Up.7D
MYH9									1.319		SM.Only.Up.7D
MYL1									1.729		SM.Only.Up.7D
MYO1E									1.011		SM.Only.Up.7D
N4BP2									1.497		SM.Only.Up.7D
NEIL3									1.734		SM.Only.Up.7D
NMB									1.118		SM.Only.Up.7D
NMS									1.567		SM.Only.Up.7D
NPL									1.303		SM.Only.Up.7D
NSUN4									1.472		SM.Only.Up.7D
NSUN7									1.556		SM.Only.Up.7D
NT5DC1									1.116		SM.Only.Up.7D
NUDT12									1.039		SM.Only.Up.7D
NUP43									1.25		SM.Only.Up.7D
OPA1									1.52		SM.Only.Up.7D
PAPSS1									1.361		SM.Only.Up.7D
PARP11									1.023		SM.Only.Up.7D
PARP14									2.535		SM.Only.Up.7D
PARP9									1.91		SM.Only.Up.7D
PCTK1									1.139		SM.Only.Up.7D
PGM3									1.179		SM.Only.Up.7D
PHF5A									1.138		SM.Only.Up.7D
POLE2									1.382		SM.Only.Up.7D
POLH									1.28		SM.Only.Up.7D
PPIL5									1.211		SM.Only.Up.7D
PRCP									1.472		SM.Only.Up.7D
PRDM15									1.513		SM.Only.Up.7D
PRDX4									1.567		SM.Only.Up.7D
PRKD1									1.273		SM.Only.Up.7D
PSMB9									1.329		SM.Only.Up.7D
PSPH									1.113		SM.Only.Up.7D
PTPN12									1.18		SM.Only.Up.7D
PTPRG									1.63		SM.Only.Up.7D
PXK									1.607		SM.Only.Up.7D
RAB31									1.157		SM.Only.Up.7D
RANBP3									1.36		SM.Only.Up.7D
RARA									1.755		SM.Only.Up.7D
RBBP8									1.057		SM.Only.Up.7D
RCN2									1.076		SM.Only.Up.7D
RHBDL2									1.617		SM.Only.Up.7D
RHOBTB1									1.972		SM.Only.Up.7D
RHOJ									1.575		SM.Only.Up.7D
RNF213									2.17		SM.Only.Up.7D
ROBO1									1.583		SM.Only.Up.7D
ROD1									1.433		SM.Only.Up.7D
RPGRIP1L									1.53		SM.Only.Up.7D
RPN1									1.256		SM.Only.Up.7D
RYK									1.082		SM.Only.Up.7D
SAAL1									1.282		SM.Only.Up.7D
SAMD9L									2.747		SM.Only.Up.7D
SAP30									1.041		SM.Only.Up.7D
SAR1A									1.09		SM.Only.Up.7D
SCARB1									1.799		SM.Only.Up.7D
SCLT1									1.148		SM.Only.Up.7D
SIX4									1.748		SM.Only.Up.7D

**SLBP**									1.231		SM.Only.Up.7D
**SLC15A2**									1.224		SM.Only.Up.7D
**SLC1A4**									1.719		SM.Only.Up.7D
**SLC25A44**									1.096		SM.Only.Up.7D
**SLC30A8**									1.431		SM.Only.Up.7D
**SLC35A3**									1.325		SM.Only.Up.7D
**SLC35F2**									1.009		SM.Only.Up.7D
**SMC2**									1.931		SM.Only.Up.7D
**SMC6**									1.17		SM.Only.Up.7D
**SNX24**									1.163		SM.Only.Up.7D
**SNX29**									1.32		SM.Only.Up.7D
**SNX7**									1.616		SM.Only.Up.7D
**SPATA17**									1.389		SM.Only.Up.7D
**SRP54**									1.03		SM.Only.Up.7D
**SRP9**									1.024		SM.Only.Up.7D
**SS18**									1.112		SM.Only.Up.7D
**STAT2**									1.462		SM.Only.Up.7D
**TAC1**									1.152		SM.Only.Up.7D
**TBC1D8B**									1.49		SM.Only.Up.7D
**THBS2**									5.074		SM.Only.Up.7D
**TMEM106C**									1.286		SM.Only.Up.7D
**TMEM107**									1.137		SM.Only.Up.7D
**TMEM194B**									1.366		SM.Only.Up.7D
**TMEM30A**									1.049		SM.Only.Up.7D
**TMEM45A**									1.081		SM.Only.Up.7D
**TMPO**									1.222		SM.Only.Up.7D
**TOR1B**									1.078		SM.Only.Up.7D
**TPST1**									1.512		SM.Only.Up.7D
**TRPS1**									1.124		SM.Only.Up.7D
**TSPAN17**									1.674		SM.Only.Up.7D
**TST**									2.286		SM.Only.Up.7D
**TUBA1C**									1.929		SM.Only.Up.7D
**TUBA3D**									2.63		SM.Only.Up.7D
**TUBD1**									2.033		SM.Only.Up.7D
**UBA7**									1.35		SM.Only.Up.7D
**UBAC2**									1.062		SM.Only.Up.7D
**UBR7**									1.936		SM.Only.Up.7D
**UCHL1**									1.286		SM.Only.Up.7D
**UHMK1**									1.602		SM.Only.Up.7D
**UHRF1BP1**									1.563		SM.Only.Up.7D
**UHRF2**									1.206		SM.Only.Up.7D
**USP18**									1.869		SM.Only.Up.7D
**VKORC1**									1.021		SM.Only.Up.7D
**WIPI1**									1.563		SM.Only.Up.7D
**WSB1**									1.286		SM.Only.Up.7D
**XPOT**									1.061		SM.Only.Up.7D
**YES1**									1.254		SM.Only.Up.7D
**YIPF5**									1.375		SM.Only.Up.7D
**ZDHHC13**									1.161		SM.Only.Up.7D
**ZNF133**									1.129		SM.Only.Up.7D
**ZNF214**									1.115		SM.Only.Up.7D
**ZNF81**									2.445		SM.Only.Up.7D
**ZPLD1**									1.168		SM.Only.Up.7D
**ZUFSP**									1.135		SM.Only.Up.7D
**ZW10**									1.009		SM.Only.Up.7D
**ZWINT**									1.204		SM.Only.Up.7D
