# Supplementary material for: Temporal Network Based Analysis of Cell Specific Vein Graft Transcriptome Defines Key Pathways and Hub Genes in Implantation Injury
Source: PLoS One. 2012 Jun 15;7(6):e39123. doi: 10.1371/journal.pone.0039123 (PMC3376111; doi:10.1371/journal.pone.0039123)
Supplement: Table S3 — Gene Ontology analysis of K-means clusters. A) EC, and B) SMC: This analysis is performed using the Database for Annotation, Visualization and Integrated Discovery (DAVID) v6.7 and gene ontology categories with multiple tests (Holm–Bonferroni method) corrected P value <0.05 were considered significant. (PDF) [file pone.0039123.s013.pdf]

Table S3A: **Gene Ontology enrichment analysis of EC K-means clusters.**

Cluster I..

Primary Processes	**Term**	Genes	Count	%	PValue	FDR
	**GO:0042981~regulation of apoptosis**	DLC1, IER3, CSF2, PTGS2, HSPA1A, NFKB1, GCH1, PLAGL1, EDNRB, BDNF, THBS1, MYC, FOSL1, IL6, CRYAB, SOCS3, CYCS, AARS, NR4A1, BIRC3, BTG2, IL12A, RIPK2, DCUN1D3, TNFAIP3	25	17.4825175	2.04E-07	3.39E-04
	**GO:0009611~response to wounding**	IRAK2, GNA13, SELP, KLF6, NFKBIZ, IL6, NFKBID, MAP2K3, C4BPB, NFKB1, TNFAIP6, ADM, EREG, RIPK2, HBEGF, THBS1, SELE, KDM6B	18	12.5874126	5.92E-06	0.00985864
Related Processes	**GO:0048870~cell motility**	FGF19, SELP, IL6, VCAM1, EDNRB, NDEL1, ID1, IL12A, DNAJA1, HBEGF, THBS1, PPAP2B, SELE	13	9.09090909	2.28E-05	0.0380667
	**GO:0006954~inflammatory response**	IRAK2, NFKBIZ, SELP, IL6, NFKBID, MAP2K3, NFKB1, C4BPB, TNFAIP6, RIPK2, THBS1, SELE, KDM6B	13	9.09090909	3.99E-05	0.06652501
	**GO:0043067~regulation of programmed cell death**	DLC1, IER3, CSF2, PTGS2, HSPA1A, NFKB1, GCH1, PLAGL1, EDNRB, BDNF, THBS1, MYC, FOSL1, IL6, CRYAB, SOCS3, CYCS, AARS, NR4A1, BIRC3, BTG2, IL12A, RIPK2, DCUN1D3, TNFAIP3	25	17.4825175	2.44E-07	4.07E-04
	**GO:0010941~regulation of cell death**	DLC1, IER3, CSF2, PTGS2, HSPA1A, NFKB1, GCH1, PLAGL1, EDNRB, BDNF, THBS1, MYC, FOSL1, IL6, CRYAB, SOCS3, CYCS, AARS, NR4A1, BIRC3, BTG2, IL12A, RIPK2, DCUN1D3, TNFAIP3	25	17.4825175	2.61E-07	4.35E-04
	**GO:0043066~negative regulation of apoptosis**	CSF2, IER3, IL6, SOCS3, CRYAB, AARS, HSPA1A, NFKB1, BIRC3, EDNRB, BDNF, BTG2, RIPK2, TNFAIP3, THBS1, MYC	16	11.1888112	7.43E-07	0.00123809
	**GO:0043069~negative regulation of programmed cell death**	CSF2, IER3, IL6, SOCS3, CRYAB, AARS, HSPA1A, NFKB1, BIRC3, EDNRB, BDNF, BTG2, RIPK2, TNFAIP3, THBS1, MYC	16	11.1888112	8.86E-07	0.0014772
	**GO:0060548~negative regulation of cell death**	CSF2, IER3, IL6, SOCS3, CRYAB, AARS, HSPA1A, NFKB1, BIRC3, EDNRB, BDNF, BTG2, RIPK2, TNFAIP3, THBS1, MYC	16	11.1888112	9.18E-07	0.00152974
	**GO:0006916~anti-apoptosis**	CSF2, IER3, BDNF, SOCS3, CRYAB, RIPK2, NFKB1, HSPA1A, TNFAIP3, BIRC3, THBS1, MYC	12	8.39160839	2.72E-06	0.00452938

Cluster II

Primary Processes	**Term**	Genes	Count	%	PValue	FDR
	**GO:0006955~immune response**	IL1R2, AQP9, NCF1C, CCL7, CFP, NUDCD1, IL1RAP, LTF, IL1B, CD4, CLEC4D, IL1A, IL18R1, IL18RAP, OLR1, IL8, NCF2, NCF4, IL1RL2, NLRP3, TNFSF8, PRELID1, LAT2, IL18BP, PPBP, LILRB3, LYST, PLCG2, CLEC7A, TREM1, HSPD1, CLEC5A, PTAFR	33	15.3488372	5.81E-10	9.81E-07
	**GO:0006954~inflammatory response**	BMP2, IL18RAP, IL8, S100A8, OLR1, ADORA2A, S100A9, NLRP3, CCL7, CFP, TFRC, AOAH, CCR3, IL1RAP, IL1B, CLEC7A, IL1A, PTAFR	18	8.37209302	1.71E-06	0.00289533
	**GO:0030595~leukocyte chemotaxis**	IL8, LYST, S100A9, IL1B, ITGAM, SYK	6	2.79069767	1.25E-04	0.21084822
	**GO:0019220~regulation of phosphate metabolic process**	C13ORF18, BMP2, PLEK, HMGCR, ADORA2A, KITLG, INHBA, SPRED2, IL1B, CD4, NRG1, FGD4, SYK, IL22RA2, HTR2A	15	6.97674419	0.00566398	9.14741415
	**GO:0051174~regulation of phosphorus metabolic process**	C13ORF18, BMP2, PLEK, HMGCR, ADORA2A, KITLG, INHBA, SPRED2, IL1B, CD4, NRG1, FGD4, SYK, IL22RA2, HTR2A	15	6.97674419	0.00566398	9.14741415
	**GO:0006801~superoxide metabolic process**	CYB5R4, NCF2, PREX1, NCF1C	4	1.86046512	0.00436282	7.11849035
	**GO:0016072~rRNA metabolic process**	NOP2, RRP1B, DKC1, NOP58, FBL, FTSJ3	6	2.79069767	0.00930976	14.612509
Related Processes						
	**GO:0006935~chemotaxis**	CCRL2, PPBP, IL8, LYST, CCR3, S100A9, IL1B, ITGAM, CCL7, PTAFR, SYK	11	5.11627907	5.78E-05	0.09754602
	**GO:0022613~ribonucleoprotein complex biogenesis**	EIF6, NOP2, RRP1B, DKC1, WDR77, NOP58, SNRPF, FBL, FTSJ3, MRTO4	10	4.65116279	7.07E-04	1.18754184
	**GO:0060326~cell chemotaxis**	IL8, LYST, S100A9, IL1B, ITGAM, SYK	6	2.79069767	1.62E-04	0.27246362

**GO:0002768~immune response-regulating cell surface receptor signaling pathway**	LAT2, PLCG2, PTPN22, CACNB4, CLEC7A, SYK	6	2.79069767	2.31E-04	0.38950546
**GO:0002253~activation of immune response**	CFP, LAT2, PLCG2, PTPN22, CACNB4, CLEC7A, HSPD1, SYK	8	3.72093023	2.63E-04	0.4426927
**GO:0045321~leukocyte activation**	LAT2, IL8, PREX1, PLCG2, PTPN22, BCL6, CD4, TREML2, CLEC7A, HSPD1, ITGAM, SYK	12	5.58139535	4.14E-04	0.69618021

Cluster III

	**Term**	Genes	Count	%	PValue	FDR
Primary Processes						
	**GO:0009259~ribonucleotide metabolic process**	TCIRG1, NME1, ADK, ADSL, ATP1A1, PAICS	6	4.28571429	0.00721917	11.1943138
	**GO:0045321~leukocyte activation**	ITGAL, CPLX2, DOCK2, CHD7, GIMAP5, TPD52, HELLS	7	5	0.01468331	21.5241278
	**GO:0002520~immune system development**	SGPL1, DOCK2, CHD7, GIMAP5, CASP8, SLC25A38, TPD52, KDR	8	5.71428571	0.00764275	11.813136
Related Processes						
	**GO:0002521~leukocyte differentiation**	DOCK2, CHD7, GIMAP5, CASP8, TPD52	5	3.57142857	0.02271658	31.3753295
	**GO:0009156~ribonucleoside monophosphate biosynthetic process**	ADK, ADSL, PAICS	3	2.14285714	0.01508621	22.0482739
	**GO:0009152~purine ribonucleotide biosynthetic process**	TCIRG1, NME1, ADSL, ATP1A1, PAICS	5	3.57142857	0.01567157	22.8039335

Cluster IV

	**Term**	Genes	Count	%	PValue	FDR
Primary Processes						
	**GO:0006954~inflammatory response**	CCL2, ADORA3, LYN, AIF1, LY86, TLR1, IL1RN, TLR2, C1R, CCL4, SLC11A1, CYBB, CXCR4, IL10RB, PLA2G7, SERPINA1, CD14	17	8.5	1.79E-06	0.00295887
	**GO:0002252~immune effector process**	SLC11A1, PTPRC, TNFSF13B, LYN, TUBB2C, FCER1G, C1R, LY9, PRKCD, HLA-DRA	10	5	3.30E-05	0.0545404
	**GO:0045321~leukocyte activation**	PTPRC, LYN, TLR1, TLR2, ITGA4, SKAP2, PRKCD, VAV1, CD48, SLC11A1, CD80, CXCR4, LCP1, LCP2	14	7	6.82E-06	0.01128355
	**GO:0006928~cell motion**	PLAT, CCL2, VAV3, S100P, WASF1, TUBB2C, ARF6, ITGA4, CCL4, TPM3, PLAUR, ARPC1B, CORO1A, CXCR4, ARPC2, CXCL16, VCAN	17	8.5	1.86E-04	0.30725174
	**GO:0001817~regulation of cytokine production**	CD83, SLC11A1, BPI, CD80, IL18, TLR1, PYCARD, TLR2, FCER1G, CD14	10	5	3.29E-04	0.54236694
Related Processes						
	**GO:0006955~immune response**	CCL2, LY86, IL18, GPSM3, TUBB2C, TLR1, TLR2, GPR65, C1R, LY9, CCL4, IL31RA, SLC11A1, CLEC4E, CXCR4, IL10RB, FCGR1A, FCER1G, IL2RG, FYB, PTPRC, C5AR1, LYN, IL1RN, CD1A, VAV1, PRKCD, CD83, CORO1A, CYBB, OASL, BPI, TNFSF13B, CXCL16, CD14, LCP1, LCP2, HLA-DRA	38	19	6.69E-15	1.10E-11
	**GO:0002696~positive regulation of leukocyte activation**	CD83, PTPRC, CDKN1A, CORO1A, PLDN, TNFSF13B, CD80, IL18, IL2RG	9	4.5	3.97E-05	0.06564029
	**GO:0050865~regulation of cell activation**	CD83, PTPRC, CDKN1A, CORO1A, BPI, PLDN, TNFSF13B, CD80, IL18, IL2RG, IL31RA	11	5.5	4.82E-05	0.07978162
	**GO:0050867~positive regulation of cell activation**	CD83, PTPRC, CDKN1A, CORO1A, PLDN, TNFSF13B, CD80, IL18, IL2RG	9	4.5	5.52E-05	0.09135682
	**GO:0002443~leukocyte mediated immunity**	SLC11A1, LYN, TUBB2C, FCER1G, C1R, LY9, PRKCD, HLA-DRA	8	4	7.54E-05	0.12466234
	**GO:0042981~regulation of apoptosis**	PIK3CG, PTPRC, CCL2, VAV3, BCL2A1, TUBB2C, PIM1, TLR2, STK17B, STK17A, HGF, ANXA5, CALR, VAV1, IL31RA, BTK, CASP10, CDKN1A, CASP4, TNFSF13B, TIAM2, PYCARD, CSTB, HSPA5	24	12	8.70E-05	0.14380631

**GO:0043067~regulation of programmed cell death**	PIK3CG, PTPRC, CCL2, VAV3, BCL2A1, TUBB2C, PIM1, TLR2, STK17B, STK17A, HGF, ANXA5, CALR, VAV1, IL31RA, BTK, CASP10, CDKN1A, CASP4, TNFSF13B, TIAM2, PYCARD, CSTB, HSPA5	24	12	1.01E-04	0.16684037
**GO:0010941~regulation of cell death**	PIK3CG, PTPRC, CCL2, VAV3, BCL2A1, TUBB2C, PIM1, TLR2, STK17B, STK17A, HGF, ANXA5, CALR, VAV1, IL31RA, BTK, CASP10, CDKN1A, CASP4, TNFSF13B, TIAM2, PYCARD, CSTB, HSPA5	24	12	1.07E-04	0.17629307

Cluster V

**Term**	**Genes**	**Count**	**%**	**PValue**	**FDR**
**GO:0007067~mitosis**	KIF23, KIFC1, NEK2, ANLN, PTTG1, KIF2C, SPC25, NCAPH, NCAPG, CDCA2, CCNA2, ASPM, ERCC6L, CDCA3, CDC6, KIF11, DLGAP5, KIF15, TPX2, NUF2, KIF18A, BIRC5, NDC80, PBK, UBE2C, NCAPD3, CCNB1, MAD2L1, RCC2, BUB1B, C21ORF45	31	19.8717949	7.50E-26	1.20E-22
**GO:0002495~antigen processing and presentation of peptide antigen via MHC class II**	IFI30, HLA-DMA, CD74	3	1.92307692	0.00269999	4.24266828
**GO:0002504~antigen processing and presentation of peptide or polysaccharide antigen via MHC class II**	HLA-DQB1, IFI30, HLA-DMB, HLA-DMA, CD74, HLA-DQA1	6	3.84615385	1.81E-05	0.02894663
**GO:0000278~mitotic cell cycle**	KIF23, KIFC1, PRC1, NEK2, ANLN, PTTG1, CCNE1, KIF2C, SPC25, NCAPH, NCAPG, CDCA2, CCNA2, ASPM, ERCC6L, CDCA3, CDC6, KIF11, DLGAP5, KIF15, TPX2, NUF2, KIF18A, BIRC5, NDC80, PBK, UBE2C, CDKN3, NCAPD3, CCNB1, MAD2L1, RCC2, BUB1B, C21ORF45	34	21.7948718	1.96E-22	3.14E-19
**GO:0000070~mitotic sister chromatid segregation**	KIFC1, NCAPH, MAD2L1, NEK2, NCAPG, DLGAP5, KIF18A, NDC80, NCAPD3	9	5.76923077	2.02E-09	3.25E-06
**GO:0007052~mitotic spindle organization**	KIF23, SPC25, KIF11, PRC1, NDC80	5	3.20512821	1.22E-05	0.0196269
**GO:0019882~antigen processing and presentation**	HLA-DQB1, IFI30, HLA-DMB, HLA-DMA, CD74, HLA-DQA1	6	3.84615385	0.00147849	2.34459356
**GO:0007346~regulation of mitotic cell cycle**	CDC6, MAD2L1, NEK2, DLGAP5, BUB1B, BIRC5, ANLN, UBE2C, CCNA2	9	5.76923077	1.48E-04	0.23660116
**GO:0019886~antigen processing and presentation of exogenous peptide antigen via MHC class II**	IFI30, HLA-DMA, CD74	3	1.92307692	0.00269999	4.24266828
**GO:0002478~antigen processing and presentation of exogenous peptide antigen**	IFI30, HLA-DMA, CD74	3	1.92307692	0.00519973	8.01966124

Cluster VI

	**Term**	Genes	Count	%	PValue	FDR
Primary Processes	**GO:0030199~collagen fibril organization**	LUM, COL3A1, COL1A2, COL1A1, LOX, ADAMTS3, COL5A2, ADAMTS2, COL5A1	9	7.5	3.22E-11	5.19E-08
	**GO:0030198~extracellular matrix organization**	LUM, COL3A1, COL1A2, DCN, COL1A1, LOX, ADAMTS3, COL5A2, ADAMTS2, COL5A1	10	8.33333333	9.11E-08	1.47E-04
	**GO:0051604~protein maturation**	C1QA, C7, C6, KLKB1, C1S, ADAMTS3, C1QC, ADAMTS2	8	6.66666667	3.95E-05	0.06366734
	**GO:0002455~humoral immune response mediated by circulating immunoglobulin**	C1QA, C7, C6, C1S, C1QC	5	4.16666667	8.51E-05	0.1371088
Related Processes						
	**GO:0043062~extracellular structure organization**	LUM, COL3A1, NLGN1, DCN, COL5A2, COL5A1, COL1A2, LOX, COL1A1, AGRN, ADAMTS3, ADAMTS2, F2R	13	10.8333333	3.70E-09	5.96E-06
	**GO:0065004~protein-DNA complex assembly**	HIST1H2BB, HIST1H2BC, HIST1H2BD, HIST1H2BE, HIST1H2BF, HIST1H2BG, HIST1H2BH, CENPF, HIST1H2BO, HIST1H2BN, H2BFS, HIST1H2BK, HIST2H2BE, HIST1H2BI, HIST1H2BJ, HIST3H3	12	10	8.25E-11	1.33E-07

**GO:0006334~nucleosome assembly**	HIST1H2BB, HIST1H2BC, HIST1H2BD, HIST1H2BE, HIST1H2BF, HIST1H2BG, HIST1H2BH, HIST1H2BO, HIST1H2BN, H2BFS, HIST1H2BK, HIST2H2BE, HIST1H2BI, HIST1H2BJ, HIST3H3	11	9.16666667	7.26E-10	1.17E-06
**GO:0031497~chromatin assembly**	HIST1H2BB, HIST1H2BC, HIST1H2BD, HIST1H2BE, HIST1H2BF, HIST1H2BG, HIST1H2BH, HIST1H2BO, HIST1H2BN, H2BFS, HIST1H2BK, HIST2H2BE, HIST1H2BI, HIST1H2BJ, HIST3H3	11	9.16666667	1.03E-09	1.66E-06
**GO:0006323~DNA packaging**	HIST1H2BB, HIST1H2BC, HIST1H2BD, HIST1H2BE, HIST1H2BF, HIST1H2BG, HIST1H2BH, NUSAP1, HIST1H2BO, HIST1H2BN, H2BFS, HIST1H2BK, HIST2H2BE, HIST1H2BI, HIST1H2BJ, HIST3H3	12	10	1.29E-09	2.07E-06
**GO:0034728~nucleosome organization**	HIST1H2BB, HIST1H2BC, HIST1H2BD, HIST1H2BE, HIST1H2BF, HIST1H2BG, HIST1H2BH, HIST1H2BO, HIST1H2BN, H2BFS, HIST1H2BK, HIST2H2BE, HIST1H2BI, HIST1H2BJ, HIST3H3	11	9.16666667	2.01E-09	3.24E-06
**GO:0032963~collagen metabolic process**	COL3A1, COL1A1, ADAMTS3, ADAMTS2, MMP2, COL5A1	6	5	1.98E-06	0.00319726
**GO:0043588~skin development**	COL3A1, COL1A2, COL1A1, COL5A2, ADAMTS2, COL5A1	6	5	2.38E-06	0.00384008

Cluster VII

	Term	Genes	Count	%	PValue	FDR
Primary Processes						
	**GO:0006936~muscle contraction**	KCNMA1, FXYD1, SLC8A1, GNAO1, PDE4D, EDNRA, ACTG2, DES, SMTN, SLMAP, RYR2, MYOM1, CHRNA1, SGCA, DTNA	15	4.42477876	1.28E-06	0.00216832
	**GO:0030182~neuron differentiation**	KCNMA1, CCK, GNAO1, TUBB2B, NTF3, MDGA1, EMX2, PTPRR, SOX5, LIFR, RORB, RORA, SLIT2, GPR98, NTRK3, EFHD1, ALDH1A2, DGKG, DMD, BCL2, NTRK2, SMARCA1, SLITRK6, FEZ1	24	7.07964602	1.06E-05	0.01803256
	**GO:0050880~regulation of blood vessel size**	KCNMA1, EDNRA, ACTG2, P2RX1, NTS, KCNJ8, PDE5A, GUCY1A3	8	2.35988201	6.02E-05	0.10203775
	**GO:0009187~cyclic nucleotide metabolic process**	ADCY5, PDE5A, GUCY1A2, GUCY1A3, PDE8B, PDE4D, RORA	7	2.06489676	7.35E-05	0.12461839
	**GO:0003018~vascular process in circulatory system**	KCNMA1, EDNRA, ACTG2, P2RX1, NTS, KCNJ8, PDE5A, GUCY1A3	8	2.35988201	1.08E-04	0.1835343
	**GO:0008016~regulation of heart contraction**	SLC8A1, DES, CYP2J2, GNAO1, ATP2A2, RYR2, S100A1, DMPK	8	2.35988201	6.38E-04	1.07666117
	**GO:0009991~response to extracellular stimulus**	RBP4, SLC8A1, A2M, CCK, GATM, TIMP3, PPARGC1A, ALDH1A2, GSN, BCL2, RYR2, SLC22A3, VLDLR	13	3.83480826	0.00103136	1.73547215
Related Processes						
	**GO:0044057~regulation of system process**	KCNMA1, SLC8A1, SNCAIP, CCK, CYP2J2, GNAO1, NTF3, PPP1R12B, EPHX2, KCNMB2, MYL9, DMPK, PLCE1, DES, P2RX1, ATP2A2, NTRK2, RYR2, GUCY1A3, CNN1, LGI1, S100A1	22	6.48967552	4.58E-07	7.77E-04
	**GO:0055082~cellular chemical homeostasis**	KCNMA1, FXYD1, PLP1, SLC8A1, CCK, NTF3, EPHX2, PPARGC1A, ITPR1, KCNMB2, DMPK, EDNRA, PLCE1, P2RX1, ATP2A2, BCL2, RYR2, RGN, CHRNA1	19	5.60471976	3.52E-04	0.59596295
	**GO:0048878~chemical homeostasis**	KCNMA1, FXYD1, RBP4, SLC8A1, PLP1, CCK, NTF3, EPHX2, PPARGC1A, TCF7L2, ITPR1, KCNMB2, DMPK, EDNRA, PLCE1, GOT1, P2RX1, ATP2A2, BCL2, RYR2, RGN, CHRNA1	22	6.48967552	7.80E-04	1.31524496
	**GO:0006873~cellular ion homeostasis**	KCNMA1, FXYD1, PLP1, SLC8A1, CCK, NTF3, EPHX2, ITPR1, KCNMB2, DMPK, EDNRA, PLCE1, P2RX1, ATP2A2, BCL2, RYR2, RGN, CHRNA1	18	5.30973451	8.14E-04	1.37241534
	**GO:0009123~nucleoside monophosphate metabolic process**	ADCY5, PDE5A, GUCY1A2, GUCY1A3, PDE8B, PDE4D, RORA	7	2.06489676	0.00155329	2.6028806
	**GO:0042490~mechanoreceptor differentiation**	KCNMA1, NTRK3, NTF3, NTRK2, GPR98	5	1.47492625	0.00207938	3.4699109

Cluster VIII

	Term	Genes	Count	%	PValue	FDR
Primary Processes						
	**GO:0019220~regulation of phosphate metabolic process**	ALS2, BMP4, CDKN2C, CCDC88C, EFNA1, MAP3K1, IGF1, PDGFC, PDCD4, INSR, TRIB2, GHR	12	6.28272251	0.00976646	14.7884872

	**GO:0051174~regulation of phosphorus metabolic process**	ALS2, BMP4, CDKN2C, CCDC88C, EFNA1, MAP3K1, IGF1, PDGFC, PDCD4, INSR, TRIB2, GHR	12	6.28272251	0.00976646	14.7884872
	**GO:0060429~epithelium development**	BMP4, LAMA3, SHROOM3, HOXA5, PPL, IFT172, JAG1, NR2F2	8	4.18848168	0.008026	12.3132323
	**GO:0043405~regulation of MAP kinase activity**	EFNA1, MAP3K1, PDCD4, INSR, TRIB2, GHR	6	3.14136126	0.01389214	20.3964949
	**GO:0043471~regulation of cellular carbohydrate catabolic process**	PPP1R3C, IGF1, INSR	3	1.57068063	0.01097828	16.4730326
	**GO:0043470~regulation of carbohydrate catabolic process**	PPP1R3C, IGF1, INSR	3	1.57068063	0.01097828	16.4730326
Related Processes						
	**GO:0001932~regulation of protein amino acid phosphorylation**	BMP4, CCDC88C, MAP3K1, IGF1, PDGFC, PDCD4, INSR, GHR	8	4.18848168	0.00181501	2.91877842
	**GO:0042325~regulation of phosphorylation**	ALS2, BMP4, CDKN2C, CCDC88C, EFNA1, MAP3K1, IGF1, PDGFC, PDCD4, INSR, TRIB2, GHR	12	6.28272251	0.00735354	11.338912
	**GO:0002009~morphogenesis of an epithelium**	BMP4, SHROOM3, HOXA5, IFT172, JAG1	5	2.61780105	0.01885406	26.6822636
	**GO:0045859~regulation of protein kinase activity**	ALS2, CDKN2C, EFNA1, MAP3K1, PDGFC, PDCD4, INSR, TRIB2, GHR	9	4.71204189	0.02322012	31.8248538

Table S3B: Gene Ontology enrichment analysis of SMC K-means clusters

Cluster I

Primary Processes	Term	Genes	Count	%	PValue	FDR
	GO:0042981~regulation of apoptosis	IER3, CCL2, PTGS2, STK17B, NFKBIA, NFKB1, GCH1, PLAGL1, EDNRB, BDNF, THBS1, MYC, FOSL1, ANGPTL4, IL6, CRYAB, SOCS3, CYCS, NR4A2, NR4A1, MALT1, BIRC3, BIRC2, CDKN1A, DUSP1, BTG2, TNFAIP3, DCUN1D3	28	17.3913044	3.35E-08	5.60E-05
	GO:0006952~defense response	SELP, NFKBIZ, IL6, IL1R1, CCL2, NFKBID, MAP2K3, IL1RN, MALT1, NFKB1, CD40, CCL4, GCH1, HDAC4, FOS, TNFAIP6, KCNJ8, CXCL16, THBS1, FOSL1, TNIP1, SELE	22	13.6645963	1.06E-06	0.00177337
	GO:0050900~leukocyte migration	VCAM1, ICAM1, SELP, EDNRB, IL6, CCL2, CXCL16, SELE	8	4.9689441	1.71E-06	0.00285081
	GO:0048870~cell motility	FGF19, ICAM1, SELP, IL6, CCL2, NR4A2, VCAM1, EDNRB, NDEL1, CXCL16, DNAJA1, HBEGF, THBS1, SELE	14	8.69565217	1.53E-05	0.02553435
Related Processes						
	GO:0010033~response to organic substance	IL1R1, CCL2, PTGS2, NFKBIA, GCH1, TRIB1, FOS, PLIN2, DNAJA1, THBS1, MYC, FOSL1, HSPA8, CYR61, EGR1, SELP, IL6, CRYAB, SOCS3, IL1RN, NR4A2, MALT1, BIRC2, HDAC4, CDKN1A, GLUL, THBD, BTG2, DUSP1, KCNJ8, CXCL16, DNAJB1, PTPN1, SELE	34	21.1180124	1.68E-13	2.81E-10
	GO:0002237~response to molecule of bacterial origin	SELP, IL6, CCL2, PTGS2, SOCS3, NFKBIA, MALT1, GCH1, TRIB1, FOS, THBD, KCNJ8, SELE	13	8.07453416	6.29E-11	1.05E-07
	GO:0032496~response to lipopolysaccharide	SELP, FOS, CCL2, THBD, PTGS2, KCNJ8, SOCS3, NFKBIA, SELE, TRIB1, GCH1	11	6.83229814	5.39E-09	9.01E-06
	GO:0051173~positive regulation of nitrogen compound metabolic process	NFKBIA, NFKB1, PLAGL1, FOS, MEIS2, REL, TEAD4, ABRA, NFATC2, MYC, FOSL1, HBB, SERTAD1, EGR1, KLF5, KLF6, ICAM1, IL6, MAP2K3, NR4A2, NR4A1, CD40, HHEX, HDAC4, IRF1	25	15.5279503	3.13E-08	5.24E-05
	GO:0043067~regulation of programmed cell death	IER3, CCL2, PTGS2, STK17B, NFKBIA, NFKB1, GCH1, PLAGL1, EDNRB, BDNF, THBS1, MYC, FOSL1, ANGPTL4, IL6, CRYAB, SOCS3, CYCS, NR4A2, NR4A1, MALT1, BIRC3, BIRC2, CDKN1A, DUSP1, BTG2, TNFAIP3, DCUN1D3	28	17.3913044	4.11E-08	6.88E-05
	GO:0010941~regulation of cell death	IER3, CCL2, PTGS2, STK17B, NFKBIA, NFKB1, GCH1, PLAGL1, EDNRB, BDNF, THBS1, MYC, FOSL1, ANGPTL4, IL6, CRYAB, SOCS3, CYCS, NR4A2, NR4A1, MALT1, BIRC3, BIRC2, CDKN1A, DUSP1, BTG2, TNFAIP3, DCUN1D3	28	17.3913044	4.44E-08	7.42E-05
	GO:0031328~positive regulation of cellular biosynthetic process	NFKBIA, NFKB1, PLAGL1, FOS, MEIS2, REL, TEAD4, ABRA, THBS1, NFATC2, MYC, FOSL1, HBB, SERTAD1, EGR1, KLF5, KLF6, ICAM1, IL6, MAP2K3, NR4A2, NR4A1, HHEX, HDAC4, IRF1	25	15.5279503	1.00E-07	1.67E-04
	GO:0043066~negative regulation of apoptosis	IER3, IL6, CCL2, SOCS3, CRYAB, NR4A2, NFKBIA, MALT1, NFKB1, BIRC3, EDNRB, BDNF, CDKN1A, BTG2, TNFAIP3, THBS1, MYC, ANGPTL4	18	11.1801242	1.16E-07	1.95E-04
	GO:0009891~positive regulation of biosynthetic process	NFKBIA, NFKB1, PLAGL1, FOS, MEIS2, REL, TEAD4, ABRA, THBS1, NFATC2, MYC, FOSL1, HBB, SERTAD1, EGR1, KLF5, KLF6, ICAM1, IL6, MAP2K3, NR4A2, NR4A1, HHEX, HDAC4, IRF1	25	15.5279503	1.31E-07	2.19E-04
	GO:0043069~negative regulation of programmed cell death	IER3, IL6, CCL2, SOCS3, CRYAB, NR4A2, NFKBIA, MALT1, NFKB1, BIRC3, EDNRB, BDNF, CDKN1A, BTG2, TNFAIP3, THBS1, MYC, ANGPTL4	18	11.1801242	1.42E-07	2.38E-04
	GO:0060548~negative regulation of cell death	IER3, IL6, CCL2, SOCS3, CRYAB, NR4A2, NFKBIA, MALT1, NFKB1, BIRC3, EDNRB, BDNF, CDKN1A, BTG2, TNFAIP3, THBS1, MYC, ANGPTL4	18	11.1801242	1.48E-07	2.48E-04
	GO:0045944~positive regulation of transcription from RNA polymerase II promoter	EGR1, KLF6, IL6, NR4A2, NFKBIA, NR4A1, NFKB1, PLAGL1, HDAC4, FOS, HHEX, MEIS2, TEAD4, IRF1, ABRA, NFATC2, FOSL1, MYC	18	11.1801242	2.27E-07	3.80E-04

GO:0045941~positive regulation of transcription	KLF5, EGR1, KLF6, IL6, MAP2K3, NR4A2, NFKBIA, NR4A1, NFKB1, PLAGL1, HDAC4, HHEX, FOS, MEIS2, REL, TEAD4, IRF1, ABRA, NFATC2, FOSL1, MYC, SERTAD1	22	13.6645963	2.58E-07	4.31E-04
GO:0045935~positive regulation of nucleobase, nucleoside, nucleotide and nucleic acid metabolic process	KLF5, EGR1, KLF6, IL6, MAP2K3, NR4A2, NFKBIA, NR4A1, NFKB1, CD40, PLAGL1, HDAC4, HHEX, FOS, MEIS2, REL, TEAD4, IRF1, ABRA, NFATC2, FOSL1, MYC, SERTAD1	23	14.2857143	3.30E-07	5.52E-04
GO:0045893~positive regulation of transcription, DNA-dependent	EGR1, KLF6, IL6, MAP2K3, NR4A2, NFKBIA, NR4A1, NFKB1, PLAGL1, HDAC4, FOS, HHEX, MEIS2, REL, TEAD4, IRF1, ABRA, NFATC2, FOSL1, MYC	20	12.4223603	3.68E-07	6.15E-04
GO:0051254~positive regulation of RNA metabolic process	EGR1, KLF6, IL6, MAP2K3, NR4A2, NFKBIA, NR4A1, NFKB1, PLAGL1, HDAC4, FOS, HHEX, MEIS2, REL, TEAD4, IRF1, ABRA, NFATC2, FOSL1, MYC	20	12.4223603	4.18E-07	6.98E-04
GO:0010628~positive regulation of gene expression	KLF5, EGR1, KLF6, IL6, MAP2K3, NR4A2, NFKBIA, NR4A1, NFKB1, PLAGL1, HDAC4, HHEX, FOS, MEIS2, REL, TEAD4, IRF1, ABRA, NFATC2, FOSL1, MYC, SERTAD1	22	13.6645963	4.21E-07	7.04E-04
GO:0009617~response to bacterium	SELP, IL6, CCL2, PTGS2, SOCS3, NFKBIA, MALT1, GCH1, TRIB1, FOS, THBD, KCNJ8, SELE	13	8.07453416	6.41E-07	0.00107157
GO:0010557~positive regulation of macromolecule biosynthetic process	KLF5, EGR1, KLF6, IL6, MAP2K3, NR4A2, NFKBIA, NR4A1, NFKB1, PLAGL1, HDAC4, HHEX, FOS, MEIS2, REL, TEAD4, IRF1, ABRA, NFATC2, THBS1, FOSL1, MYC, SERTAD1	23	14.2857143	7.31E-07	0.00122266
GO:0034097~response to cytokine stimulus	HDAC4, FOS, IL1R1, PTGS2, SOCS3, CXCL16, SELE, FOSL1, GCH1	9	5.59006211	1.36E-06	0.00226845
GO:0010604~positive regulation of macromolecule metabolic process	NFKBIA, NFKB1, PLAGL1, FOS, EDNRB, MEIS2, REL, TEAD4, ABRA, THBS1, NFATC2, MYC, FOSL1, SERTAD1, EGR1, KLF5, KLF6, IL6, MAP2K3, NR4A2, NR4A1, CD40, HDAC4, HHEX, IRF1, SELE	26	16.1490683	1.62E-06	0.00271515
GO:0042127~regulation of cell proliferation	KLF5, NAMPT, IL6, TP53I11, CCL2, PTGS2, MARCKSL1, NFKBIA, CD40, TRIB1, VCAM1, HDAC4, HHEX, EDNRB, BDNF, CDKN1A, BTG2, CD274, SERPINE1, HBEGF, THBS1, MYC, FOSL1, SERTAD1	24	14.9068323	4.38E-06	0.00732742
GO:0006954~inflammatory response	SELP, NFKBIZ, IL6, CCL2, NFKBID, MAP2K3, IL1RN, NFKB1, CD40, CCL4, HDAC4, FOS, TNFAIP6, THBS1, SELE	15	9.31677019	5.81E-06	0.00971089
GO:0009611~response to wounding	SELP, KLF6, NFKBIZ, IL6, CCL2, NFKBID, MAP2K3, IL1RN, NFKB1, CD40, CCL4, HDAC4, FOS, TNFAIP6, THBD, SERPINE1, HBEGF, THBS1, SELE	19	11.8012422	7.27E-06	0.01215208
GO:0006916~anti-apoptosis	IER3, BDNF, CCL2, SOCS3, CRYAB, NFKBIA, NFKB1, MALT1, TNFAIP3, BIRC3, THBS1, MYC	12	7.45341615	8.32E-06	0.01390483
GO:0042493~response to drug	HDAC4, FOS, TOP1, CDKN1A, BDNF, CCL2, PTGS2, PLIN2, SOCS3, ATP1A1, LRP2, FOSL1	12	7.45341615	1.30E-05	0.02175714
GO:0051384~response to glucocorticoid stimulus	FOS, CDKN1A, IL6, CCL2, PTGS2, DUSP1, IL1RN, FOSL1	8	4.9689441	1.43E-05	0.02384076
GO:0051674~localization of cell	FGF19, ICAM1, SELP, IL6, CCL2, NR4A2, VCAM1, EDNRB, NDEL1, CXCL16, DNAJA1, HBEGF, THBS1, SELE	14	8.69565217	1.53E-05	0.02553435
GO:0031960~response to corticosteroid stimulus	FOS, CDKN1A, IL6, CCL2, PTGS2, DUSP1, IL1RN, FOSL1	8	4.9689441	2.51E-05	0.04197572
GO:0016477~cell migration	FGF19, ICAM1, SELP, IL6, CCL2, NR4A2, VCAM1, EDNRB, NDEL1, CXCL16, HBEGF, THBS1, SELE	13	8.07453416	2.54E-05	0.04248735
GO:0048545~response to steroid hormone stimulus	FOS, CDKN1A, IL6, CCL2, PTGS2, DUSP1, SOCS3, CRYAB, IL1RN, THBS1, FOSL1	11	6.83229814	2.69E-05	0.04499526
GO:0006928~cell motion	FGF19, SELP, ICAM1, IL6, CCL2, PTGS2, NR4A2, CCL4, VCAM1, EDNRB, BDNF, NDEL1, CXCL16, DNAJA1, HBEGF, THBS1, SELE	17	10.5590062	2.69E-05	0.04501342
GO:0010647~positive regulation of cell communication	FGF19, IL6, CCL2, PTGS2, SLC20A1, MALT1, CD40, BIRC2, HHEX, REL, PLK2, LANCL2, ABRA, THBS1	14	8.69565217	3.16E-05	0.05279643
GO:0032570~response to progesterone stimulus	FOS, CCL2, SOCS3, THBS1, FOSL1	5	3.10559006	4.42E-05	0.07394669
GO:0009719~response to endogenous stimulus	IL6, CCL2, PTGS2, SOCS3, CRYAB, IL1RN, NR4A2, BIRC2, FOS, CDKN1A, DUSP1, BTG2, PTPN1, THBS1, FOSL1	15	9.31677019	6.69E-05	0.11183924
GO:0009628~response to abiotic stimulus	CCL2, SOCS3, CRYAB, FOS, CDKN1A, THBD, BTG2, DUSP1, KCNJ8, LRP2, DCUN1D3, THBS1, FOSL1, MYC	14	8.69565217	9.92E-05	0.16567463

	GO:0007243~protein kinase cascade	CCL2, SOCS3, CRYAB, MAP2K3, NFKBIB, DUSP10, STK17B, NFKBIA, MALT1, TRIB1, STAT4, DUSP16, THBS1, STK38L	14	8.69565217	1.05E-04	0.17491221
	GO:0006357~regulation of transcription from RNA polymerase II promoter	EGR1, KLF6, IL6, NR4A2, LMCD1, NFKBIA, NR4A1, NFKB1, FOSB, PLAGL1, HDAC4, FOS, HHEX, MEIS2, TEAD4, IRF1, ABRA, NFATC2, FOSL1, MYC	20	12.4223603	1.45E-04	0.24251693
	GO:0009967~positive regulation of signal transduction	FGF19, HHEX, IL6, PLK2, REL, SLC20A1, LANCL2, ABRA, MALT1, CD40, THBS1, BIRC2	12	7.45341615	2.18E-04	0.36305116
	GO:0014070~response to organic cyclic substance	FOS, CDKN1A, PTGS2, PLIN2, BTG2, SOCS3, BIRC2, FOSL1	8	4.9689441	2.37E-04	0.3955129
	GO:0008284~positive regulation of cell proliferation	KLF5, NAMPT, IL6, CCL2, PTGS2, MARCKSL1, CD40, VCAM1, HDAC4, CDKN1A, HBEGF, FOSL1, MYC, SERTAD1	14	8.69565217	3.15E-04	0.52583047
	GO:0009725~response to hormone stimulus	IL6, CCL2, PTGS2, SOCS3, CRYAB, IL1RN, FOS, CDKN1A, BTG2, DUSP1, PTPN1, THBS1, FOSL1	13	8.07453416	3.75E-04	0.62588376
	GO:0010243~response to organic nitrogen	CDKN1A, CCL2, PTGS2, BTG2, NR4A2, BIRC2	6	3.72670808	4.49E-04	0.74832714
	GO:0070482~response to oxygen levels	HMOX2, CDKN1A, CCL2, SOCS3, NR4A2, THBS1, BIRC2, ANGPTL4	8	4.9689441	5.99E-04	0.9964909
	GO:0051090~regulation of transcription factor activity	ICAM1, HDAC4, IL6, NFKBIA, ABRA, MALT1, TRIB1	7	4.34782609	6.43E-04	1.06892347
	GO:0001775~cell activation	EGR1, VCAM1, ICAM1, HDAC4, SHB, KLF6, IL6, IRF1, MALT1, NFKB2, CD40	11	6.83229814	7.17E-04	1.19155876
	GO:0051789~response to protein stimulus	EGR1, FOS, DNAJA1, NR4A2, DNAJB1, HSPA8, CYR61	7	4.34782609	7.86E-04	1.30549949
	GO:0043122~regulation of I-kappaB kinase/NF-kappaB cascade	PLK2, REL, SLC20A1, MALT1, CD40, TNFAIP3, BIRC2	7	4.34782609	7.86E-04	1.30549949
	GO:0051591~response to cAMP	FOS, THBD, DUSP1, BIRC2, FOSL1	5	3.10559006	8.59E-04	1.42666915
	GO:0044093~positive regulation of molecular function	ICAM1, IL6, MAP2K3, CYCS, NR4A2, MALT1, SDC4, HOMER1, GCH1, HDAC4, EDNRB, NDEL1, ABRA, THBS1, SELE, MYC	16	9.9378882	9.37E-04	1.55597463
	GO:0050867~positive regulation of cell activation	VCAM1, SELP, CDKN1A, IL6, MALT1, CD40, THBS1	7	4.34782609	9.52E-04	1.58019924
	GO:0046649~lymphocyte activation	EGR1, VCAM1, ICAM1, HDAC4, SHB, KLF6, IRF1, MALT1, CD40	9	5.59006211	9.89E-04	1.64130302

Cluster II

	Term	Genes	Count	%	PValue	FDR
Primary Proc						
	GO:0022613~ribonucleoprotein complex biogenesis	EIF6, GTPBP4, NIP7, BYSL, FBL, MRTO4, RCL1, NOP2, DKC1, PRMT5, WDR77, NOP58, ZNHIT6, IMP4, FTSJ3	15	7.31707317	3.82E-08	6.36E-05
	GO:0042254~ribosome biogenesis	EIF6, RCL1, GTPBP4, NOP2, DKC1, NIP7, BYSL, NOP58, IMP4, FBL, FTSJ3, MRTO4	12	5.85365854	2.61E-07	4.35E-04
	GO:0006396~RNA processing	RPL36A, MAGOH, CWC15, POLR2D, PRPF4, PUS7, TRMT61B, FBL, HNRPDL, DDX39, NOP2, DKC1, METTL1, TRMT6, PRMT5, WDR77, NOP58, PPIL3, RPL10A, RBM28, IMP4, FTSJ3	22	10.7317073	2.84E-06	0.00473734
	GO:0034660~ncRNA metabolic process	NOP2, DKC1, TRMT6, METTL1, MAEL, NOP58, MKI67IP, IMP4, PUS7, FBL, TRMT61B, FTSJ3	12	5.85365854	1.13E-04	0.18874753
	GO:0034470~ncRNA processing	NOP2, DKC1, TRMT6, METTL1, NOP58, IMP4, PUS7, FBL, TRMT61B, FTSJ3	10	4.87804878	4.57E-04	0.75949461
	GO:0016072~rRNA metabolic process	NOP2, DKC1, NOP58, MKI67IP, IMP4, FBL, FTSJ3	7	3.41463415	0.00109257	1.80506122
Related Proc	GO:0006412~translation	EIF6, RPL36A, EIF3B, PTRH1, TRMT6, EEF1E1, RPL7L1, EIF4A1, MRPS10, RPL10A, RPL22L1, MRPL35	12	5.85365854	0.00235385	3.85074027
	GO:0000375~RNA splicing, via transesterification reactions	DDX39, RPL36A, PRMT5, MAGOH, CWC15, WDR77, POLR2D, PRPF4	8	3.90243902	0.00259858	4.24301053

	Term	Genes	Count	%	PValue	FDR
	GO:0000398~nuclear mRNA splicing, via spliceosome	DDX39, RPL36A, PRMT5, MAGOH, CWC15, WDR77, POLR2D, PRPF4	8	3.90243902	0.00259858	4.24301053
	GO:0000377~RNA splicing, via transesterification reactions with bulged adenosine as nucleophile	DDX39, RPL36A, PRMT5, MAGOH, CWC15, WDR77, POLR2D, PRPF4	8	3.90243902	0.00259858	4.24301053
	GO:0009451~RNA modification	DKC1, METTL1, PUS7, TRMT61B, FTSJ3	5	2.43902439	0.00267535	4.3657565
	GO:0006364~rRNA processing	NOP2, DKC1, NOP58, IMP4, FBL, FTSJ3	6	2.92682927	0.00519049	8.30610929
	GO:0008380~RNA splicing	DDX39, RPL36A, PRMT5, MAGOH, CWC15, WDR77, PPIL3, POLR2D, RBM28, PRPF4	10	4.87804878	0.00780393	12.2384864

Cluster III

Primary Proc	Term	Genes	Count	%	PValue	FDR
	GO:0006399~tRNA metabolic process	YARS, ELAC2, PUS1, GARS, QTRT1, VARS, TSEN2, MARS	8	7.07964602	1.42E-05	0.02239822
	GO:0009116~nucleoside metabolic process	NME1, ADK, NT5C3, QTRT1, TPMT	5	4.42477876	8.95E-04	1.40216712
Related Proc	GO:0034660~ncRNA metabolic process	YARS, ELAC2, PUS1, GARS, QTRT1, VARS, TSEN2, MARS	8	7.07964602	9.04E-04	1.41652659
	GO:0044271~nitrogen compound biosynthetic process	TCIRG1, NME1, ADK, SLC25A38, RSAD1, ASNS, ENOPH1, QTRT1, SMS	9	7.96460177	0.00153366	2.39249423
	GO:0044093~positive regulation of molecular function	UBE2N, MYD88, MAPKSP1, NME1, HTR7, PLCG2, KITLG, LRP8, DPH3, PLCB2, ADAM9	11	9.73451327	0.00590763	8.92470746

Cluster IV

Primary Proc	Term	Genes	Count	%	PValue	FDR
	GO:0006954~inflammatory response	ITGAL, IL18RAP, OLR1, S100A9, C4BPB, CFP, CCL20, CCR3, AOAH, IL1RAP, IL1B, SERPINA1, CLEC7A, IL1A	14	8.1871345	3.00E-05	0.04890493
	GO:0030595~leukocyte chemotaxis	CORO1A, S100A9, IL1B, ITGAM, SYK	5	2.92397661	5.42E-04	0.87908483
	GO:0006955~immune response	ITGAL, AQP9, GPSM3, NCF1C, LY9, LIF, CFP, CCL20, IL1RAP, LTF, IL1B, IL2RG, CLEC4D, IL1A, IL18R1, IL18RAP, OLR1, NCF2, NCF4, IL1RL2, C4BPB, VAV1, CORO1A, IL18BP, PPBP, LILRB3, VEGFA, CLEC7A, TREM1, LCP2	30	17.5438597	6.20E-11	1.01E-07
Related Proc	GO:0006952~defense response	ITGAL, S100A9, NCF1C, CFP, CCL20, AOAH, IL1RAP, LTF, IL1B, CSF3R, SERPINA1, IL1A, IL18R1, IL18RAP, NCF2, OLR1, IL1RL2, HCK, C4BPB, INHBA, CORO1A, PPBP, LILRB3, CCR3, CLEC7A	25	14.619883	1.50E-08	2.45E-05
	GO:0042330~taxis	CCRL2, CORO1A, PPBP, CCL20, RALBP1, CCR3, S100A9, IL1B, FGF10, ITGAM, SYK	11	6.43274854	5.82E-06	0.00948284
	GO:0006935~chemotaxis	CCRL2, CORO1A, PPBP, CCL20, RALBP1, CCR3, S100A9, IL1B, FGF10, ITGAM, SYK	11	6.43274854	5.82E-06	0.00948284
	GO:0045087~innate immune response	CFP, IL18R1, CORO1A, IL18RAP, NCF2, IL1RL2, IL1RAP, NCF1C, C4BPB, CLEC7A	10	5.84795322	1.23E-05	0.02002625
	GO:0007626~locomotory behavior	RALBP1, S100A9, FGF10, CACNB4, ITGAM, CCRL2, CORO1A, CCL20, PPBP, CCR3, SLC18A2, IL1B, SYK	13	7.60233918	2.54E-05	0.04142546
	GO:0009611~response to wounding	PTPN6, ITGAL, IL18RAP, PLEK, OLR1, S100A9, FGF10, C4BPB, CFP, CCL20, AOAH, CCR3, IL1RAP, SERPINB2, IL1B, SERPINA1, CLEC7A, IL1A	18	10.5263158	3.05E-05	0.04962044
	GO:0007229~integrin-mediated signaling pathway	ITGAL, PLEK, PRAM1, ITGB7, VAV1, ITGAM, SYK	7	4.09356725	8.07E-05	0.13139977

GO:0060326~cell chemotaxis	CORO1A, S100A9, IL1B, ITGAM, SYK	5	2.92397661	6.64E-04	1.07653676
GO:0001775~cell activation	ITGAL, PLEK, PREX1, PTPN22, TREML2, CLEC7A, TPD52, VAV1, ITGAM, LCP2, SYK	11	6.43274854	7.58E-04	1.22792834
GO:0002684~positive regulation of immune system process	CFP, CORO1A, VEGFA, PTPN22, IL1B, C4BPB, IL2RG, CACNB4, CLEC7A, SYK	10	5.84795322	7.84E-04	1.26886637
GO:0045321~leukocyte activation	ITGAL, PREX1, PTPN22, TREML2, CLEC7A, TPD52, VAV1, ITGAM, LCP2, SYK	10	5.84795322	8.82E-04	1.42687384
GO:0007166~cell surface receptor linked signal transduction	VIP, ITGAL, IFITM1, FFAR2, FST, PTPN22, FGF10, CACNB4, ITGAM, LIF, CCRL2, CCL20, ITGB7, CDA, SYK, PIK3CG, PTPN6, IL18RAP, PLEK, GPR171, AREGB, VAV1, INHBA, P2RY13, PPBP, EMR1, CCR3, LILRB3, PRAM1, VEGFA, EMR3, AREG, CLEC7A, EMR2, LCP2	34	19.8830409	9.58E-04	1.54990673
GO:0042110~T cell activation	ITGAL, PTPN22, TREML2, CLEC7A, VAV1, ITGAM, SYK	7	4.09356725	0.00189753	3.04697029
GO:0007155~cell adhesion	C1ORF38, ITGAL, PLEK, OLR1, SIGLEC10, LY9, ITGAM, CORO1A, LAMB3, EMR1, CCR3, ITGB7, CSF3R, CLEC7A, AMICA1, SYK, ALX1	17	9.94152047	0.00221498	3.54813005
GO:0022610~biological adhesion	C1ORF38, ITGAL, PLEK, OLR1, SIGLEC10, LY9, ITGAM, CORO1A, LAMB3, EMR1, CCR3, ITGB7, CSF3R, CLEC7A, AMICA1, SYK, ALX1	17	9.94152047	0.00224697	3.59848876
GO:0050900~leukocyte migration	CORO1A, S100A9, IL1B, ITGAM, SYK	5	2.92397661	0.00276595	4.41216402
GO:0002253~activation of immune response	CFP, PTPN22, C4BPB, CACNB4, CLEC7A, SYK	6	3.50877193	0.00281395	4.48709287
GO:0048584~positive regulation of response to stimulus	CFP, VEGFA, PTPN22, IL1B, FGF10, C4BPB, CACNB4, CLEC7A, SYK	9	5.2631579	0.0030256	4.8168407
GO:0007610~behavior	RALBP1, S100A9, FGF10, CACNB4, ITGAM, CCRL2, CORO1A, CCL20, PPBP, CCR3, SLC18A2, IL1B, SYK	13	7.60233918	0.00325759	5.17703507
GO:0050778~positive regulation of immune response	CFP, PTPN22, IL1B, C4BPB, CACNB4, CLEC7A, SYK	7	4.09356725	0.00383039	6.0609122
GO:0046649~lymphocyte activation	ITGAL, PTPN22, TREML2, CLEC7A, TPD52, VAV1, ITGAM, SYK	8	4.67836257	0.00447055	7.03959297

Cluster V

	Term	Genes	Count	%	PValue	FDR
Primary Proc						
	GO:0007017~microtubule-based process	DNAH11, TUBB, DYNLL1, TUBB2C, TUBD1, TUBA4A, FBXO5, NDC80, KPNA2	9	8.57142857	2.56E-04	0.39998269
	GO:0051258~protein polymerization	TUBB, TUBB2C, TUBD1, TUBA4A, FBXO5	5	4.76190476	2.96E-04	0.46157181
	GO:0002822~regulation of adaptive immune response based on somatic recombination of immune receptors built from immunoglobulin superfamily domains	CD86, IL27RA, IL7R, DPP4, B2M	5	4.76190476	4.62E-04	0.7201387
Related Proc	GO:0002819~regulation of adaptive immune response	CD86, IL27RA, IL7R, DPP4, B2M	5	4.76190476	4.95E-04	0.77147907
	GO:0000278~mitotic cell cycle	CDC7, BCAT1, TUBB, PSME2, NCAPG2, TPX2, FBXO5, NDC80, KPNA2, PSMB8	10	9.52380952	7.18E-04	1.1174803
	GO:0051052~regulation of DNA metabolic process	IL27RA, S100A11, PDGFRA, PCNA, IL7R, KPNA2	6	5.71428571	9.20E-04	1.42876731
	GO:0022402~cell cycle process	CDC7, BCAT1, TUBB, PSME2, NCAPG2, TPX2, FBXO5, NDC80, KPNA2, PSMB8, TRIP13	11	10.4761905	0.00390912	5.94055509
	GO:0022403~cell cycle phase	CDC7, BCAT1, TUBB, NCAPG2, TPX2, FBXO5, NDC80, KPNA2, TRIP13	9	8.57142857	0.00584996	8.76570817
	GO:0006461~protein complex assembly	TUBB, RRM2, TUBB2C, RRM1, CAPG, TUBD1, TUBA4A, GJA1, FBXO5, OLFM1	10	9.52380952	0.00589806	8.83470827
	GO:0070271~protein complex biogenesis	TUBB, RRM2, TUBB2C, RRM1, CAPG, TUBD1, TUBA4A, GJA1, FBXO5, OLFM1	10	9.52380952	0.00589806	8.83470827
	GO:0007018~microtubule-based movement	DNAH11, TUBB, TUBB2C, TUBD1, TUBA4A	5	4.76190476	0.00653716	9.74682825
	GO:0043933~macromolecular complex subunit organization	TUBB, RRM2, TUBB2C, RRM1, CAPG, H2AFZ, TUBD1, TUBA4A, GJA1, FBXO5, SCARB1, OLFM1	12	11.4285714	0.00673567	10.0284016
	GO:0006952~defense response	BPI, CYBB, TUBB, IL27RA, LY86, TUBB2C, MNDA, CLIC1, MX2, HLA-DRA, SPP1	11	10.4761905	0.00703059	10.4452065

GO:0009611~response to wounding	PLAT, CYBB, ACVRL1, FBLN5, LY86, PDGFRA, VCAN, SCARB1, CTSB, SPP1	10	9.52380952	0.00800189	11.8051737

Cluster VI

	Term	Genes	Count	%	PValue	FDR
Primary Proc						
Related Proc	GO:0006955~immune response	FYB, PSMB10, PTPRC, SP100, LYN, IL18, TLR1, GPR65, TLR2, CD180, SLC11A1, TMEM173, CXCL14, CLEC4E, NUDCD1, CXCR4, FCGR1A, IL12A, FCER1G, CLEC5A, LCP1, CD14, RAB27A, GBP1	24	17.9104478	4.14E-10	6.78E-07
	GO:0045321~leukocyte activation	PTPRC, GIMAP5, LYN, TLR1, TLR2, SKAP2, CD48, SLC11A1, DOCK2, CD80, CXCR4, LCP1, RAB27A	13	9.70149254	1.52E-07	2.49E-04
	GO:0006952~defense response	PTPRC, ADORA3, SP100, LYN, AIF1, TLR1, TLR2, CD180, CD48, SLC11A1, TMEM173, CXCR4, FCGR1A, IL12A, PLA2G7, CLEC5A, CD14, RAB27A, TYROBP	19	14.1791045	3.13E-07	5.13E-04
	GO:0001775~cell activation	PTPRC, GIMAP5, LYN, TLR1, TLR2, SKAP2, CD48, SLC11A1, DOCK2, CD80, CXCR4, LCP1, RAB27A	13	9.70149254	9.45E-07	0.00154951
	GO:0042110~T cell activation	CD48, SLC11A1, PTPRC, DOCK2, GIMAP5, CD80, CXCR4, LCP1, RAB27A	9	6.71641791	3.27E-06	0.00536646
	GO:0046649~lymphocyte activation	CD48, SLC11A1, PTPRC, DOCK2, GIMAP5, CD80, CXCR4, SKAP2, LCP1, RAB27A	10	7.46268657	1.26E-05	0.02064852
	GO:0002274~myeloid leukocyte activation	CD48, SLC11A1, GIMAP5, LYN, TLR1, TLR2	6	4.47761194	1.86E-05	0.03042426
	GO:0001819~positive regulation of cytokine production	SLC11A1, GIMAP5, IL18, TLR2, IL12A, FCER1G, CD14	7	5.2238806	4.46E-05	0.07305091
	GO:0002822~regulation of adaptive immune response based on somatic recombination of immune receptors built from immunoglobulin superfamily domains	SLC11A1, PTPRC, GIMAP5, CD80, IL12A, FCER1G	6	4.47761194	4.48E-05	0.07336351
	GO:0001817~regulation of cytokine production	SLC11A1, GIMAP5, CD80, IL18, TLR1, TLR2, IL12A, FCER1G, CD14	9	6.71641791	4.61E-05	0.07559009
	GO:0002819~regulation of adaptive immune response	SLC11A1, PTPRC, GIMAP5, CD80, IL12A, FCER1G	6	4.47761194	4.89E-05	0.08009918
	GO:0002824~positive regulation of adaptive immune response based on somatic recombination of immune receptors built from immunoglobulin superfamily domains	SLC11A1, PTPRC, GIMAP5, IL12A, FCER1G	5	3.73134328	5.90E-05	0.09670491
	GO:0045087~innate immune response	SLC11A1, TMEM173, SP100, FCGR1A, TLR1, TLR2, CD180, RAB27A	8	5.97014925	5.90E-05	0.09678765
	GO:0002821~positive regulation of adaptive immune response	SLC11A1, PTPRC, GIMAP5, IL12A, FCER1G	5	3.73134328	6.74E-05	0.11041638
	GO:0002366~leukocyte activation during immune response	SLC11A1, LYN, TLR2, LCP1, RAB27A	5	3.73134328	1.23E-04	0.20102704
	GO:0002263~cell activation during immune response	SLC11A1, LYN, TLR2, LCP1, RAB27A	5	3.73134328	1.23E-04	0.20102704
	GO:0002684~positive regulation of immune system process	SLC11A1, PTPRC, GIMAP5, CD80, LYN, IL18, TLR2, IL12A, FCER1G	9	6.71641791	3.07E-04	0.50174854
	GO:0051240~positive regulation of multicellular organismal process	SLC11A1, PLA2G4A, GIMAP5, IL18, TLR2, IL12A, FCER1G, CA2, CD14	9	6.71641791	3.62E-04	0.59273258
	GO:0006954~inflammatory response	SLC11A1, ADORA3, LYN, AIF1, CXCR4, TLR1, TLR2, PLA2G7, CD180, CD14	10	7.46268657	5.34E-04	0.87140994

GO:0050778~positive regulation of immune response	SLC11A1, PTPRC, GIMAP5, LYN, TLR2, IL12A, FCER1G	7	5.2238806	6.07E-04	0.99020974
GO:0007243~protein kinase cascade	FYB, PTPRC, GRB2, CXCR4, NLK, TLR1, TLR2, STK17A, BTK, DUSP6	10	7.46268657	0.00133468	2.16639754
GO:0032844~regulation of homeostatic process	PTPRC, PLA2G4A, GIMAP5, LMO2, LYN, CA2	6	4.47761194	0.00135426	2.19785736
GO:0009611~response to wounding	SLC11A1, ADORA3, LYN, AIF1, CXCR4, TLR1, TLR2, PLA2G7, TFPI2, CD180, CD14, RAB27A	12	8.95522388	0.00142073	2.30455542
GO:0030097~hemopoiesis	SGPL1, PTPRC, DOCK2, GIMAP5, LMO2, LYN, CASP8, TACC3	8	5.97014925	0.00152656	2.47420135
GO:0048584~positive regulation of response to stimulus	SLC11A1, PTPRC, PLA2G4A, GIMAP5, LYN, TLR2, IL12A, FCER1G	8	5.97014925	0.00152656	2.47420135
GO:0031349~positive regulation of defense response	PLA2G4A, GIMAP5, TLR2, IL12A, FCER1G	5	3.73134328	0.00185199	2.99417328
GO:0050870~positive regulation of T cell activation	PTPRC, GIMAP5, CD80, IL18, IL12A	5	3.73134328	0.00214764	3.46428549
GO:0048534~hemopoietic or lymphoid organ development	SGPL1, PTPRC, DOCK2, GIMAP5, LMO2, LYN, CASP8, TACC3	8	5.97014925	0.0026395	4.24167752
GO:0006468~protein amino acid phosphorylation	FYB, PTPRC, LIMK2, LYN, NLK, PIK3CD, STK17A, PRKCB, BTK, PLK3, GMFG, CXCR4, KALRN	13	9.70149254	0.00286047	4.58900599
GO:0002237~response to molecule of bacterial origin	SLC11A1, PLA2G4A, TLR2, IL12A, CD14	5	3.73134328	0.00336653	5.37998779
GO:0002520~immune system development	SGPL1, PTPRC, DOCK2, GIMAP5, LMO2, LYN, CASP8, TACC3	8	5.97014925	0.00367301	5.8560279
GO:0043065~positive regulation of apoptosis	PTPRC, PLA2G4A, CASP4, CASP8, TLR2, IL12A, STK17A, RAB27A, BTK, KALRN	10	7.46268657	0.00368226	5.87035608
GO:0006915~apoptosis	SGPL1, RNF144B, TMEM173, CASP4, LITAF, CXCR4, CASP8, GPR65, STK17A, DRAM1, CD14, KALRN	12	8.95522388	0.00382149	6.08584671
GO:0043068~positive regulation of programmed cell death	PTPRC, PLA2G4A, CASP4, CASP8, TLR2, IL12A, STK17A, RAB27A, BTK, KALRN	10	7.46268657	0.00385406	6.13618968
GO:0010942~positive regulation of cell death	PTPRC, PLA2G4A, CASP4, CASP8, TLR2, IL12A, STK17A, RAB27A, BTK, KALRN	10	7.46268657	0.00397206	6.31835898
GO:0012501~programmed cell death	SGPL1, RNF144B, TMEM173, CASP4, LITAF, CXCR4, CASP8, GPR65, STK17A, DRAM1, CD14, KALRN	12	8.95522388	0.00427257	6.78078515

Cluster VII

	Term	Genes	Count	%	PValue	FDR
Primary Proc						
	GO:0007067~mitosis	KIF23, KIFC1, NEK2, ANLN, AURKA, PTTG1, SPC25, NCAPH, NCAPG, CDCA2, CCNA2, HELLS, ASPM, ERCC6L, CDCA3, CDC6, KIF11, DLGAP5, KIF15, NUF2, KIF18A, CENPF, NUSAP1, CDC20, BIRC5, PBK, UBE2C, SMC2, NCAPD2, CDC25B, CCNB1, MAD2L1, CCNB2, SPAG5, PLK1, BUB1B, C21ORF45	37	19.2708333	2.42E-31	3.97E-28
	GO:0007059~chromosome segregation	CENPO, KIFC1, NEK2, DLGAP5, NUF2, KIF18A, CENPF, NUSAP1, BIRC5, PTTG1, SMC2, CENPH, NCAPD2, SPC25, NCAPH, MAD2L1, NCAPG, TOP2A	18	9.375	4.23E-17	6.93E-14
	GO:0000910~cytokinesis	PRC1, PLK1, NUSAP1, BIRC5, ANLN, MYH9, RACGAP1	7	3.64583333	7.68E-06	0.01257355
	GO:0051726~regulation of cell cycle	CDC6, NEK2, DLGAP5, HERC5, CENPF, NUSAP1, TTK, ANLN, BIRC5, CHEK2, UBE2C, CDKN3, CCNB1, PLK4, MAD2L1, BUB1B, CCNA2	17	8.85416667	1.77E-06	0.00290096

Related Proc	GO:0022402~cell cycle process	KIF23, KIFC1, PRC1, TTK, AURKA, PTTG1, CDCA2, CCNA2, ASPM, CDCA3, CDC6, KIF11, KIF15, NUSAP1, PBK, MYH9, UBE2C, RAD51, NCAPD2, SASS6, MAD2L1, FANCD2, SPAG5, BUB1B, STMN1, NEK2, ANLN, SPC25, NCAPH, NCAPG, HELLS, ERCC6L, EXO1, DLGAP5, NUF2, KIF18A, CENPF, BIRC5, CDC20, CDKN3, RACGAP1, SMC2, SC65, PSMB9, CDC25B, CCNB1, CCNB2, PLK1, C21ORF45, TP53INP1	50	26.0416667	1.38E-29	2.25E-26
	GO:0000279~M phase	KIF23, KIFC1, PRC1, NEK2, TTK, ANLN, AURKA, PTTG1, SPC25, NCAPH, NCAPG, CDCA2, CCNA2, ASPM, HELLS, ERCC6L, CDCA3, EXO1, CDC6, KIF11, DLGAP5, KIF15, NUF2, KIF18A, CENPF, NUSAP1, CDC20, BIRC5, PBK, MYH9, UBE2C, SMC2, SC65, NCAPD2, CDC25B, RAD51, CCNB1, MAD2L1, CCNB2, FANCD2, SPAG5, PLK1, BUB1B, C21ORF45, STMN1	45	23.4375	1.63E-34	2.67E-31
	GO:0000087~M phase of mitotic cell cycle	KIF23, KIFC1, NEK2, ANLN, AURKA, PTTG1, SPC25, NCAPH, NCAPG, CDCA2, CCNA2, HELLS, ASPM, ERCC6L, CDCA3, CDC6, KIF11, DLGAP5, KIF15, NUF2, KIF18A, CENPF, NUSAP1, CDC20, BIRC5, PBK, UBE2C, SMC2, NCAPD2, CDC25B, CCNB1, MAD2L1, CCNB2, SPAG5, PLK1, BUB1B, C21ORF45	37	19.2708333	4.73E-31	7.74E-28
	GO:0048285~organelle fission	KIF23, KIFC1, NEK2, ANLN, AURKA, PTTG1, SPC25, NCAPH, NCAPG, CDCA2, CCNA2, HELLS, ASPM, ERCC6L, CDCA3, CDC6, KIF11, DLGAP5, KIF15, NUF2, KIF18A, CENPF, NUSAP1, CDC20, BIRC5, PBK, UBE2C, SMC2, NCAPD2, CDC25B, CCNB1, MAD2L1, CCNB2, SPAG5, PLK1, BUB1B, C21ORF45	37	19.2708333	1.07E-30	1.75E-27
	GO:0007049~cell cycle	KIF23, KIFC1, PRC1, E2F8, TTK, AURKA, PTTG1, CDCA2, CCNA2, ASPM, CDCA3, CDC6, KIF11, KIF15, NUSAP1, PBK, MYH9, UBE2C, ESCO2, NCAPD2, RAD51, MCM6, SASS6, MAD2L1, FANCD2, SPAG5, BUB1B, STMN1, NEK2, FOXM1, ANLN, CHEK2, SPC25, NCAPH, NCAPG, HELLS, ERCC6L, EXO1, DLGAP5, NUF2, KIF18A, CENPF, BIRC5, CDC20, RACGAP1, CDKN3, SMC2, GSG2, SC65, PSMB9, CDC25B, CCNB1, CCNB2, PLK1, C21ORF45, TP53INP1	56	29.1666667	6.02E-29	9.87E-26
	GO:0007076~mitotic chromosome condensation	NCAPH, NCAPG, NUSAP1, SMC2, NCAPD2	5	2.60416667	1.21E-05	0.01981661
	GO:0051276~chromosome organization	KIFC1, NEK2, DLGAP5, KIF18A, CENPF, NUSAP1, PTTG1, SMC2, GSG2, SC65, CENPH, NCAPD2, NCAPH, MAD2L1, NCAPG, FANCD2, MYST1, TOP2A, HELLS	19	9.89583333	1.58E-05	0.02590806
	GO:0000075~cell cycle checkpoint	CDC6, MAD2L1, BUB1B, CENPF, TTK, BIRC5, CHEK2, CCNA2	8	4.16666667	9.61E-05	0.15732027
	GO:0051327~M phase of meiotic cell cycle	EXO1, NEK2, FANCD2, PLK1, MYH9, SC65, CDC25B, RAD51	8	4.16666667	1.53E-04	0.25096862
	GO:0051439~regulation of ubiquitin-protein ligase activity during mitotic cell cycle	CCNB1, MAD2L1, PLK1, BUB1B, CDC20, UBE2C, PSMB9	7	3.64583333	1.83E-04	0.29921539
	GO:0006259~DNA metabolic process	EXO1, CDC6, CCDC88A, GEN1, NEIL3, CENPF, PTTG1, ESCO2, MCM5, RAD51, MCM6, DNA2, FANCM, FANCD2, ORC6L, TOP2A, HELLS	17	8.85416667	3.05E-04	0.49918476
	GO:0051438~regulation of ubiquitin-protein ligase activity	CCNB1, MAD2L1, PLK1, BUB1B, CDC20, UBE2C, PSMB9	7	3.64583333	3.07E-04	0.50111519
	GO:0051340~regulation of ligase activity	CCNB1, MAD2L1, PLK1, BUB1B, CDC20, UBE2C, PSMB9	7	3.64583333	3.76E-04	0.61480635
	GO:0007088~regulation of mitosis	MAD2L1, NEK2, DLGAP5, CENPF, NUSAP1, TTK	6	3.125	4.96E-04	0.80966376
	GO:0051783~regulation of nuclear division	MAD2L1, NEK2, DLGAP5, CENPF, NUSAP1, TTK	6	3.125	4.96E-04	0.80966376
	GO:0051640~organelle localization	DLGAP5, KIF18A, CENPF, NUSAP1, BIRC5, MYH9, ASPM	7	3.64583333	7.44E-04	1.21172991
	GO:0031145~anaphase-promoting complex-dependent proteasomal ubiquitin-dependent protein catabolic process	CCNB1, MAD2L1, BUB1B, CDC20, UBE2C, PSMB9	6	3.125	9.86E-04	1.60251399
	GO:0031396~regulation of protein ubiquitination	CCNB1, MAD2L1, PLK1, BUB1B, CDC20, UBE2C, PSMB9	7	3.64583333	0.00115159	1.86997828
	GO:0051656~establishment of organelle localization	DLGAP5, KIF18A, CENPF, NUSAP1, BIRC5, MYH9	6	3.125	0.00129156	2.09501527

Term	Genes	Count	%	PValue	FDR
GO:0007093~mitotic cell cycle checkpoint	MAD2L1, BUB1B, CENPF, TTK, CCNA2	5	2.60416667	0.00158734	2.56897269
GO:0006260~DNA replication	DNA2, CDC6, CCDC88A, ORC6L, CENPF, TOP2A, MCM5, MCM6, RAD51	9	4.6875	0.00182363	2.94603368
GO:0007018~microtubule-based movement	KIF23, KIF14, KIFC1, KIF11, KIF27, KIF15, KIF18A	7	3.64583333	0.00215479	3.47220079
GO:0006323~DNA packaging	NCAPH, NCAPG, NUSAP1, TOP2A, SMC2, HELLS, NCAPD2	7	3.64583333	0.00256719	4.12370221

Cluster VIII

	Term	Genes	Count	%	PValue	FDR
Primary Clust						
	GO:0007229~integrin-mediated signaling pathway	COL3A1, ITGA11, ITGB5, COL16A1, ADAMDEC1, ITGBL1	6	5.2631579	7.64E-05	0.11740818
Related Clust	GO:0007155~cell adhesion	SVEP1, COL3A1, ITGA11, NLGN1, ITGB5, CERCAM, COL16A1, ITGBL1, THY1, OMD, LAMA3, MFAP4, COL8A1, SPON1	14	12.2807018	4.01E-04	0.6149094
	GO:0022610~biological adhesion	SVEP1, COL3A1, ITGA11, NLGN1, ITGB5, CERCAM, COL16A1, ITGBL1, THY1, OMD, LAMA3, MFAP4, COL8A1, SPON1	14	12.2807018	4.06E-04	0.62332736
	GO:0007160~cell-matrix adhesion	COL3A1, ITGA11, ITGB5, THY1, ITGBL1	5	4.38596491	0.00236221	3.57202894
	GO:0031589~cell-substrate adhesion	COL3A1, ITGA11, ITGB5, THY1, ITGBL1	5	4.38596491	0.00334888	5.02841499
	GO:0043062~extracellular structure organization	RXFP1, LUM, COL3A1, NLGN1, COL1A1, LOX	6	5.2631579	0.00360847	5.4081486
	GO:0030198~extracellular matrix organization	RXFP1, LUM, COL3A1, COL1A1, LOX	5	4.38596491	0.00414351	6.18634275
	GO:0006790~sulfur metabolic process	BGN, ENPP1, PHGDH, IDH1, GLCE	5	4.38596491	0.00591425	8.71936966

Cluster IX

	Term	Genes	Count	%	PValue	FDR
Primary Proc						
	GO:0003012~muscle system process	ACTG2, SLMAP, EDN1, GUCY1A3, ADRA1A, TTN, KCNQ1, SGCA, DTNA	9	3.96475771	7.32E-04	1.19516252
	GO:0006936~muscle contraction	ACTG2, SLMAP, EDN1, ADRA1A, TTN, KCNQ1, SGCA, DTNA	8	3.52422908	0.00196627	3.17954208
	GO:0001568~blood vessel development	RECK, FGF18, NRP1, EDN1, TGFBR3, NOS2, JAG1, NR2F2, FGF1, TCF7L2	10	4.40528634	0.00214782	3.46827582
Related Proc	GO:0001944~vasculature development	RECK, FGF18, NRP1, EDN1, TGFBR3, NOS2, JAG1, NR2F2, FGF1, TCF7L2	10	4.40528634	0.00252843	4.07098448
	GO:0007517~muscle organ development	MEF2C, BVES, SVIL, FHL1, TGFBR3, CACNB2, TTN, NR2F2, SGCA, IGFBP5	10	4.40528634	7.60E-04	1.24083767
	GO:0040007~growth	AR, LAMB2, GATM, HOXA5, FHL1, TGFBR3, SEPP1, TIMP3, IGFBP5	9	3.96475771	0.0012671	2.0599911
	GO:0016055~Wnt receptor signaling pathway	CCDC88C, SOSTDC1, SFRP4, TLE1, AXIN2, TCF7L2, CALCOCO1	7	3.08370044	0.00440416	6.98993794
	GO:0043009~chordate embryonic development	AR, SHROOM3, SYVN1, HOXA5, C6, EDN1, IFT172, HOXA7, TGFBR3, AXIN2, TCF7L2	11	4.84581498	0.00504497	7.96787594
	GO:0009792~embryonic development ending in birth or egg hatching	AR, SHROOM3, SYVN1, HOXA5, C6, EDN1, IFT172, HOXA7, TGFBR3, AXIN2, TCF7L2	11	4.84581498	0.00536912	8.45888533
	GO:0008217~regulation of blood pressure	ACTG2, EDN1, EPHX2, GUCY1A3, NOS2, GNB3	6	2.64317181	0.0059808	9.37872233
	GO:0008219~cell death	MEF2C, C7, MAGI3, AR, ARHGEF6, C6, ITSN1, PRUNE2, SYNE1, CDKN1B, UBA1, KRT8, ADRA1A, TNFRSF19, CABC1, AXIN2, HIP1	17	7.48898678	0.00883266	13.5539207
	GO:0016265~death	MEF2C, C7, MAGI3, AR, ARHGEF6, C6, ITSN1, PRUNE2, SYNE1, CDKN1B, UBA1, KRT8, ADRA1A, TNFRSF19, CABC1, AXIN2, HIP1	17	7.48898678	0.00940285	14.3667236

Cluster X

Primary Proc	Term	Genes	Count	%	PValue	FDR
	GO:0019725~cellular homeostasis	KCNMA1, SLC8A1, PLP1, CCK, TACR1, SNCA, MAL, PPARGC1A, KCNMB2, ADM, TXNDC16, DMD, GRID2, RYR2, RGN, STC1, CHRNA1, MT3	18	7.65957447	1.98E-04	0.3270096
	GO:0006873~cellular ion homeostasis	KCNMA1, PLP1, SLC8A1, CCK, TACR1, SNCA, MAL, KCNMB2, ADM, GRID2, RYR2, RGN, STC1, CHRNA1, MT3	15	6.38297872	5.68E-04	0.93507639
	GO:0042391~regulation of membrane potential	KCNMA1, PLP1, CCK, TACR1, SNCA, GRID2, MAL, CHRNA1, KCNMB2	9	3.82978723	4.70E-04	0.77561159
Related Proc	GO:0009187~cyclic nucleotide metabolic process	ADM, ADCY5, PDE5A, GUCY1A2, PDE8B	5	2.12765957	0.00167004	2.72763814
	GO:0044057~regulation of system process	KCNMA1, SLC8A1, CYP2J2, CCK, TACR1, PPP1R12B, SNCA, KCNMB2, ADM, NTRK2, RYR2, CNN1, LGI1	13	5.53191489	0.00102014	1.67458538
	GO:0050801~ion homeostasis	KCNMA1, PLP1, SLC8A1, CCK, TACR1, SNCA, MAL, KCNMB2, ADM, GRID2, RYR2, RGN, STC1, CHRNA1, MT3	15	6.38297872	0.00134591	2.20376637
	GO:0009123~nucleoside monophosphate metabolic process	ADM, ADCY5, PDE5A, GUCY1A2, PDE8B, NT5E	6	2.55319149	0.00196721	3.20561851
	GO:0001508~regulation of action potential	KCNMA1, PLP1, TACR1, MAL, CHRNA1, KCNMB2	6	2.55319149	0.00224537	3.65101117
	GO:0030182~neuron differentiation	KCNMA1, CCK, STMN2, NTNG1, SOX5, NRXN1, HOXD10, NTRK3, ADM, ANK3, DGKG, DMD, MAP2, NTRK2, SLITRK6	15	6.38297872	0.00253823	4.1178648
