# Supplementary material for: Temporal Network Based Analysis of Cell Specific Vein Graft Transcriptome Defines Key Pathways and Hub Genes in Implantation Injury
Source: PLoS One. 2012 Jun 15;7(6):e39123. doi: 10.1371/journal.pone.0039123 (PMC3376111; doi:10.1371/journal.pone.0039123)
Supplement: Table S4 — List of genes from significantly enriched canonical pathways in vein grafts at different time points. A) EC, B) SMC. This analysis is performed using Igenuity Pathway Analysis System and Pathways with multiple test (Holm–Bonferroni method) corrected P value <0.01 was considered significant. (PDF) [file pone.0039123.s014.pdf]

Table S4A: List of genes from significantly enriched canonical pathways in grafted EC at different time points

Pathways	2H Symbols	2H P_value	12H Symbols	12H P_value	24H Symbols	24H P_value	7D Symbols	7D P_value	30D Symbols	30D P_value
**Prothrombin Activation**	NA	#VALUE!	COL1A2, COL1A1, KLKB1, PROS1, THBD, COL3A1	0.294442163	NA	#VALUE!	COL1A2, COL1A1, COL3A1	0.595662144	COL1A2, COL1A1, COL3A1	0.002570396
**Glucocorticoid Receptor Signaling**	IL8, SELE, HSPA1A, JAK2, NFKB1, BCL2, IL1R2, GTF2B, CDKN1A, NFATC2, IL1B, SERPINE1, CSF2	0.003235937	TGFBR1, PBX1, IL6, TAF13, HSPA5, FCGR1A, SMARCA4, IL1R2, HSPA4, NFAT5, PCK2, CCL2, BAG1, VIPR1, PIK3CG, BGLAP, AKT3, SERPINE1, MMP1 (includes EG:4312), TAF12 (includes EG:6883), IL8, TAF9, SELE, GRB2, MAP3K1, HSPA9, HSPA2, PIK3R3, HSPA8, RRAS2, IL1RN, SMARCA2, CDKN1A, FKBP4, TGFB3, HSP90AA1, IL1B, A2M	0.110917482	POLR2D, SMAD3, PBX1, IL6, HSPA5, FCGR1A, IL1R2, CCL2, PCK2, PIK3CG, SERPINE1, IL8, SELE, VCAM1, GRB2, HSPA9, HSPA2, TAF9B, POLR2G, PIK3R3, RRAS2, IL1RN, SMARCA2, CDKN1A, IL1B, PIK3CB, PIK3CD, A2M	0.023988329	IL8, VCAM1, SELE, TGFBR1, IL6, HSPA5, CD163, NR3C1, FCGR1A, HSPA2, BCL2, CCL2, PCK2, IL1RN, PIK3CG, CDKN1A, SERPINE1, STAT1, ESR1	0.557185749	CDKN1A	0.523600437
**Fcg RIIB Signaling in B Lymphocytes**	NA	#VALUE!	BTK, PIK3R3, RRAS2, GRB2, PLCG2, PIK3CG, SYK, LYN	0.207491352	BTK, PIK3R3, RRAS2, GRB2, PIK3CG, SYK, LYN, PIK3CB, PIK3CD	0.008709636	BTK, PIK3CG, SYK, LYN	0.521194711	NA	#VALUE!
**Antiproliferative Role of SSTR2**	CDKN1A, GNG2	0.479733449	PIK3R3, PTPN6, GNG11, GNB3, RRAS2, GUCY1A3, PIK3CG, CDKN1A, GNB5, GNG2, CDKN1B, GUCY1B3	0.247742206	PTPN6, GUCY1A3, PIK3R3, GNB1, GNG11, RRAS2, ADCY5, PIK3CG, CDKN1A, PIK3CD, PIK3CB, GNG2, CDKN1B, GUCY1B3	0.003548134	GUCY1A3, ADCY5, PIK3CG, CDKN1A, GUCY1A2, GNG2, GUCY1B3	0.43052661	CDKN1A	0.348337315
**Macropinocytosis Signaling**	ARF6, PRKCB	0.44874539	PIK3R3, ARF6, RRAS2, PDGFA, PRKCD, PLCG2, PIK3CG, ITGB8, PDGFC, ITGB7, PRKCB	0.257039578	PDGFC, CSF1R, ITGB7, PIK3R3, ARF6, RRAS2, PRKCD, PIK3CG, HGF, CD14, PIK3CD, PIK3CB, PDGFD, ITGB5, PRKCB	0.000831764	PAK1, PRKCD, PIK3CG, ITGA5, CD14, CSF1R, PRKCB	0.363078055	NA	#VALUE!
**GPCR Signaling**	GPR65, RGS16, RGS12, NFKB1, ADRA1A, PRKCB	0.221309471	ADRA2B, PLCB2, RGS18, RGS12, CREB5, PDE4D, PIK3CG, RGS10, HTR7, AKT3, RGS14, ADORA3, GRK4, EDNRB, OPRM1, GRB2, GNAI1, PRKAR2A, PDE4B, PIK3R3, PLCB4, RRAS2, GNAO1, GPR65, EDNRA, ADORA2A, ADRA1A, PRKCB	0.251767693	ADRA2B, PLCB2, RGS18, ADRBK1, ADCY5, PIK3CG, RGS10, HTR7, RGS14, HTR2A, ADORA3, RGS2, OPRM1, GRB2, PRKAR2A, PDE4B, PIK3R3, PLCB4, RRAS2, GPR65, GNAO1, EDNRA, PIK3CB, PIK3CD, ADRA2C, PDE8B, ADORA2A, PRKCB	0.003467369	ADRA2B, PLCB2, RGS18, EDNRB, CREB5, CAMK2A, CAMK2D, ADCY5, PIK3CG, GPR65, GNAO1, RGS10, EDNRA, RGS14, PDE8B, ADRA1A, CAMK2G, PRKCB	0.331894458	NA	#VALUE!
**NF-kB Activation by Viruses**	CD4, NFKB1, PRKCB	0.230674719	PIK3R3, RRAS2, PRKCD, CD4, PIK3CG, MAP3K1, AKT3, ITGAL, PRKCB	0.564936975	CCR5, CD4, ITGA2, ITGAL, PIK3R3, RRAS2, PRKCD, PIK3CG, PIK3CB, PIK3CD, ITGB5, ITGA4, PRKCB	0.008709636	CCR5, PRKCD, CD4, PIK3CG, ITGAV, ITGA5, ITGA4, PRKCB	0.30974193	ITGA4	0.348337315
**Caveolar-mediated Endocytosis Signaling**	NA	#VALUE!	FLNB, ITSN1, ACTA2, CD48, ITGB8, ACTG1, ITGAL, ITGB7, ITGAM, HLA-A, ITGA11, FLNA, PTPN1, INSR, ITGA7	0.1	COPZ1, ITSN1, ACTA2, ITGA2, CD48, COPG2, ITGAL, ITGB7, COPG, ITGAM, ITGA11, FLNA, ITGB5, ITGA4	0.004073803	FLNA, FLNC, ITGAV, CD48, ITGA5, ACTG2 (includes EG:72), ACTG1, ITGA4	0.30974193	ITGA4	0.348337315
**CXCR4 Signaling**	RND3, CD4, EGR1, GNA13, GNG2, PRKCB	0.111943788	PLCB2, CD4, MYL5, GNB5, GNA14, PTK2, GNB3, GNG11, PIK3CG, AKT3, PXN, CXCR4, EGR1, GNAI1, ITPR1, PIK3R3, MYL9 (includes EG:10398), PLCB4, RRAS2, PAK3, PRKCD, GNAO1, LYN, GNG2, PRKCB	0.125025903	PLCB2, CXCR4, EGR1, CD4, MYL5, ITPR1, MYL6B, GNB1, MYL9 (includes EG:10398), PIK3R3, PLCB4, RHOG, RRAS2, GNG11, PAK3, ADCY5, PIK3CG, PRKCD, GNAO1, LYN, PIK3CB, PIK3CD, GNG2, PRKCB	0.00144544	PLCB2, CXCR4, CD4, MYL5, ITPR1, MYL9 (includes EG:10398), PAK1, RHOG, ADCY5, PRKCD, PIK3CG, GNAO1, LYN, GNG2, PRKCB	0.244343055	EGR1	0.404575892
**Fc Epsilon RI Signaling**	FCER1G, CSF2, LCP2, PRKCB	0.17538805	GRB2, BTK, PIK3R3, PLA2G4A, RRAS2, GAB1, PRKCD, PLCG2, PIK3CG, SYK, VAV3, LYN, FCER1G, AKT3, VAV1, LCP2, PRKCB	0.128528666	GRB2, FCER1A, BTK, PIK3R3, RRAS2, PRKCD, PIK3CG, SYK, LYN, FCER1G, MAP2K3, PIK3CD, VAV1, PIK3CB, LCP2, PRKCB	0.006309573	BTK, PRKCD, PIK3CG, SYK, VAV3, FCER1G, LYN, VAV1, MAP2K3, LCP2, PRKCB	0.194088588	NA	#VALUE!
**IL-8 Signaling**	IL8, ITGAM, RND3, NCF2, CYBB, HBEGF, GNA13, GNG2, NFKB1, BCL2, PRKCB, IRAK2	0.00047863	NAPEPLD, PLCB2, GNB5, CCND1, EIF4EBP1, PTK2, GNB3, GNG11, CXCR2, PIK3CG, GPLD1, CYBB, AKT3, IL8, GNAI1, HBEGF, PIK3R3, ITGAM, RRAS2, PRKCD, NCF2, KDR, PTGS2, GNG2, PRKCB	0.181551566	IL8, VCAM1, PLCB2, MMP2, EIF4EBP1, GNB1, PIK3R3, RRAS2, GNG11, ITGAM, RHOG, CXCR2, PIK3CG, PRKCD, KDR, NCF2, CYBB, PIK3CB, PIK3CD, GNG2, PRKCB	0.020892961	IL8, VCAM1, PLCB2, MAP4K4, MMP2, CSTB, BCL2, HMOX1, RHOG, CCND2, PRKCD, PIK3CG, KDR, NCF2, CYBB, GNG2, PRKCB	0.185780446	NA	#VALUE!
**IL-4 Signaling**	NFATC2, JAK2	0.477529274	PIK3R3, IL2RG, PTPN6, NFAT5, RRAS2, GRB2, PIK3CG, HMGA1, AKT3	0.506990708	HLA-DMA, IL2RG, PTPN6, HLA-DOA, GRB2, HMGA1, HLA-DQA1, HLA-DMB, HLA-DQB1, PIK3R3, RRAS2, PIK3CG, HLA-DRA, PIK3CB, PIK3CD	0.001174898	HLA-DQB1, HLA-DMA, IL2RG, PIK3CG, HLA-DRA, HLA-DQA1, HMGA1, HLA-DMB, NR3C1	0.158854675	HLA-DRA, HLA-DQA1	0.111943788
**GM-CSF Signaling**	CSF2RA (includes EG:1438), JAK2, CSF2, PRKCB	0.081283052	GRB2, CCND1, PIK3R3, CSF2RB, RRAS2, CSF2RA (includes EG:1438), PIK3CG, HCK, LYN, AKT3, RUNX1, BCL2A1, PRKCB	0.125025903	GRB2, PIK3R3, CSF2RB, RRAS2, CSF2RA (includes EG:1438), PIM1, PIK3CG, HCK, LYN, PIK3CD, PIK3CB, RUNX1, BCL2A1, PRKCB	0.001412538	CAMK2D, CAMK2A, PIK3CG, LYN, RUNX1, STAT1, BCL2A1, CAMK2G, PRKCB	0.107646521	NA	#VALUE!
**IL-6 Signaling**	IL1R2, IL8, IL18, IL1B, JAK2, TNFAIP6, NFKB1	0.009772372	IL6ST, IL8, IL1A, GRB2, IL6, IL1R2, COL1A1, IL18, RRAS2, IL1RN, IL1B, HSPB7, LBP, MAPKAPK2, TNFAIP6, A2M	0.128528666	IL8, ABCB1, IL1A, GRB2, IL6, IL1R2, IL18, RRAS2, IL1RN, CD14, IL1B, MAP2K3, A2M	0.021379621	COL1A1, IL18, CYP19A1, IL1RN, TNFRSF1A, SRF, CD14, MAP4K4, MAP2K3, IL6, TNFRSF11B	0.0616595	COL1A1, IL18	0.111943788
**NFAT in Reg of the IR**	CD4, FCER1G, NFATC2, GNA13, GNG2, NFKB1, LCP2, RCAN1	0.037153523	PLCB2, CSNK1G1, CD4, RCAN2, GNB5, GNA14, FCGR1A, GNB3, NFAT5, GNG11, PIK3CG, AKT3, AKAP5, GRB2, GNAI1, ITPR1, RCAN1, BTK, PIK3R3, PLCB4, RRAS2, CD80, PLCG2, SYK, GNAO1, FCER1G, LYN, MEF2C, GNG2, LCP2	0.069183097	HLA-DOA, PLCB2, CD4, RCAN2, FCER1A, HLA-DQA1, HLA-DMB, FCGR1A, GNB1, GNG11, PIK3CG, HLA-DRA, HLA-DMA, GRB2, ITPR1, HLA-DQB1, BTK, PIK3R3, PLCB4, RRAS2, CD80, SYK, GNAO1, LYN, FCER1G, CD86, PIK3CB, PIK3CD, GNG2, LCP2	0.000144544	HLA-DMA, PLCB2, CD4, HLA-DQA1, HLA-DMB, ITPR1, FCGR1A, HLA-DQB1, BTK, CD80, PIK3CG, SYK, GNAO1, HLA-DRA, FCER1G, LYN, CD86, MEF2C, GNG2, LCP2	0.0616595	HLA-DRA, HLA-DQA1	0.248885732
**Aldosterone Sig in Epithelial Cells**	NA	#VALUE!	PLCB2, SLC12A2, ITPR1, HSPA5, PIK3R3, HSPA8, PLCB4, PLCE1, PRKCD, PLCG2, PIK3CG, HSP90AA1, PRKCB	0.247742206	PLCB2, PDIA3, SLC12A2, PIP5K1B, ITPR1, HSPA5, PIK3R3, PLCB4, PLCE1, PRKCD, PIK3CG, PIK3CD, PIK3CB, PRKCB, AHCY	0.003467369	PLCB2, PLCE1, PDIA3, PRKCD, PIK3CG, PLCL2, PIP5K1B, ITPR1, HSPA5, PLCL1, PRKCB, AHCY	0.040738028	NA	#VALUE!
**Innate and Adaptive Immune Cells**	IL8, IL18, CCL4, CD4, FCER1G, IL1B, CSF2	0.009772372	IL8, TLR1, IL18, CCL4, CD4, TLR2, IL18, CCL4, HLA-A, CD80, IL1RN, FCER1G, IL1B, TLR3, TNFSF13B	0.251767693	TLR1, IL8, IL1A, CD4, CD83, CXCL10, TLR2, IL18, CCL4, CD80, IL1RN, HLA-DRA, FCER1G, CD86, IL1B	0.00691831	IL8, TLR1, IL12A, CD4, CD83, TLR2, IL18, CD80, IL1RN, HLA-DRA, FCER1G, CD86, TNFSF13B	0.040738028	IL18, HLA-DRA	0.111943788
**Leukocyte Extravasation Signaling**	ITGAM, ARHGAP9, CLDN1, NCF2, CYBB, NCF4, PRKCB	0.095499259	CD99 (includes EG:4267), NCF1C, MMP16, ACTA2, JAM2, PTK2, TIMP1, MMP8, PIK3CG, CYBB, MMP1 (includes EG:4312), MMP19, TIMP2, TIMP3, PXN, ARHGAP6, CXCR4, GNAI1, NCF4, DLC1, ITGAL, ACTG1, PIK3R3, BTK, ITGAM, ARHGAP9, VAV3, PRKCD, PLCG2, NCF2, VAV1, PRKCB	0.0616595	MMP14, ACTA2, JAM2, TIMP1, EZR, PIK3CG, CYBB, MMP19, TIMP2, ITGA4, TIMP3, VCAM1, CXCR4, MMP2, NCF4, ITGAL, BTK, PIK3R3, ITGAM, ARHGAP9, PRKCD, NCF2, PIK3CD, VAV1, PIK3CB, PRKCB	0.003630781	TIMP3, VCAM1, CXCR4, MMP14, MMP2, NCF4, ACTG1, BTK, ARHGAP9, TIMP1, CLDN1, PIK3CG, VAV3, PRKCD, NCF2, CYBB, VAV1, ACTG2 (includes EG:72), MMP19, ITGA4, MSN, PRKCB	0.040738028	CLDN1, ITGA4	0.259417936
**Thrombin Signaling**	RND3, GNA13, GNG2, NFKB1, PRKCB	0.298538262	PLCB2, F2RL2, MYL5, GNB5, GNA14, MYLK, PTK2, GNB3, PLCE1, GNG11, PIK3CG, AKT3, ARHGEF3, ARHGEF12, GRB2, GNAI1, ITPR1, PIK3R3, MYL9 (includes EG:10398), PLCB4, RRAS2, PLCG2, PRKCD, GNAO1, ARHGEF6, PPP1R12B, PPP1R12A, GNG2, PRKCB	0.128528666	PLCB2, PDIA3, MYL5, GNB1, GNG11, PLCE1, RHOG, ADCY5, PIK3CG, GATA6, ARHGEF3, GRB2, ARHGEF15, ITPR1, MYL6B, PIK3R3, MYL9 (includes EG:10398), PLCB4, RRAS2, PRKCD, GNAO1, PPP1R12B, PIK3CD, PIK3CB, GNG2, PRKCB	0.004168694	PLCB2, F2R, PDIA3, ARHGEF15, MYL5, ITPR1, PLCL2, MYL9 (includes EG:10398), CAMK2A, CAMK2D, PLCE1, RHOG, ADCY5, PIK3CG, PRKCD, GNAO1, PPP1R12B, PPP1R12A, ARHGEF3, GNG2, PLCL1, CAMK2G, PRKCB	0.030199517	NA	#VALUE!
**Glycine, Serine and Threonine Metabolism**	NA	#VALUE!	AOC3, PSAT1, PLCB2, BHMT, DMGDH, AMT (includes EG:275), PLCB4, MAOB, PLCE1, PLCG2, CHKA, GOT1, SARS	0.189670592	PSAT1, PLCB2, BHMT, PDIA3, DMGDH, GARS, AMT (includes EG:275), PLCB4, MAOB, PLCE1, GOT1, CTH, GATM, MAOA	0.004073803	PSAT1, PLCB2, MAOB, PLCE1, BHMT, PDIA3, CHKA, PHGDH, GOT1, PLCL2, PLCL1, SHMT2	0.029512092	NA	#VALUE!
**Calcium-induced T Lymphocyte Apoptosis**	CD4, NR4A1, FCER1G, NFATC2, PRKCB	0.032359366	PRKCD, CD4, FCER1G, CAPN2, ITPR1, ATP2A2, PRKCB	0.656145266	HLA-DQB1, HLA-DMA, HLA-DOA, PRKCD, CD4, HLA-DRA, HLA-DQA1, FCER1G, HLA-DMB, ITPR1, ATP2A2, PRKCB	0.00676083	HLA-DQB1, HLA-DMA, PRKCD, CD4, HLA-DRA, HLA-DQA1, FCER1G, HLA-DMB, ITPR1, ATP2A2, PRKCB	0.021877616	HLA-DRA, HLA-DQA1	0.101859139
**Endothelin-1 Signaling**	MYC, GNA13, PRKCB	0.615176873	NAPEPLD, PLCB2, CASP4, GNA14, PRDX6, PLCE1, PIK3CG, GPLD1, CASP8, GUCY1B3, CASP10, EDNRB, GUCY1A3, GRB2, PTGS1, MAPK6, GNAI1, ITPR1, LAMB2, PIK3R3, PLA2G4A, PLCB4, RRAS2, GAB1, PRKCD, PLCG2, GNAO1, EDNRA, PTGS2, PTGER2, PRKCB	0.042657952	PLCB2, PDIA3, CASP4, PLCE1, ADCY5, ECE2, PIK3CG, ECE1, GUCY1B3, CASP10, GUCY1A3, GRB2, PTGS1, MAPK6, ITPR1, PIK3R3, LAMB2, PLCB4, RRAS2, PRKCD, GNAO1, EDNRA, PIK3CD, PIK3CB, PTGER2, PRKCB	0.001202264	PLCB2, EDNRB, GUCY1A3, PDIA3, CASP4, ITPR1, PLCL2, LAMB2, CASP6, HMOX1, PLCE1, ADCY5, PIK3CG, PRKCD, GNAO1, GUCY1A2, EDNRA, CASP8, PLCL1, GUCY1B3, PRKCB, CASP10	0.016218101	NA	#VALUE!
**Cdc42 Signaling**	NA	#VALUE!	NA	#VALUE!	HLA-DMA, HLA-DOA, MYL5, ITGA2, HLA-DQA1, HLA-DMB, MYL6B, HLA-DQB1, MYL9 (includes EG:10398), PAK3, ARPC2, HLA-DRA, FCER1G, VAV1, ITGA4	0.233883724	HLA-DMA, ACTR2, ARPC1B, ARPC5L (includes EG:81873), DIAPH3, MYL5, ARPC5, HLA-DQA1, ITGA5, HLA-DMB, MYL9 (includes EG:10398), HLA-DQB1, IQGAP2, PAK1, CDC42BPA, ARPC2, HLA-DRA, ARPC4, FCER1G, PPP1R12A, VAV1, ITGA4	0.01	HLA-DRA, HLA-DQA1, ITGA4	0.091201084
**G2/M DNA Damage Checkpoint Reg**	CDKN1A	0.619441075	CDC25B, CDKN1A, TOP2A, MDM2, CHEK2	0.592925325	CDKN1A, TOP2A, MDM2	0.625172693	CDC25B, CCNB1, CCNB3, CDKN1A, TOP2A, CCNB2, PLK1, CDK1, CHEK1	0.009332543	CDKN1A, TOP2A	0.060255959
**Chemokine Signaling**	CCL4, CCL7, PRKCB	0.230674719	CCR3, PLCB2, CXCR4, GNAI1, PTK2, PLCB4, RRAS2, CCL4, CCL2, CCL7, PLCG2, PIK3CG, PPP1R12B, PPP1R12A, PRKCB	0.085113804	CCR3, PLCB2, PLCB4, CCR5, CCL4, RRAS2, CCL2, CCL7, CXCR4, PIK3CG, PPP1R12B, PRKCB	0.015488166	PLCB2, CCR5, CXCR4, CAMK2D, CAMK2A, CCL2, CCL7, PIK3CG, PPP1R12B, PPP1R12A, PPP1CA, CAMK2G, PRKCB	0.008317638	NA	#VALUE!
**RhoA Signaling**	ARHGAP9, GNA13	0.623734835	ARHGAP6, ARHGEF12, ARPC1B, ARPC5L (includes EG:81873), ACTA2, MYL5, SEPT4, MYLK3, DLC1, ACTG1, MYL9 (includes EG:10398), MYLK, PTK2, IGF1, ARHGAP9, LPAR1, ARPC2, IGF1R, PPP1R12B, PPP1R12A, SEPT6	0.0616595	MYL9 (includes EG:10398), ARHGAP9, ARPC2, EZR, ACTA2, MYL5, PPP1R12B, PIP5K1B, MYL6B, SEPT6	0.295120923	ACTR2, ARPC1B, ARPC5L (includes EG:81873), MYL5, ARPC5, ANLN, PIP5K1B, ACTG1, TTN, MYL9 (includes EG:10398), ARHGAP9, ARPC2, ARPC4, PPP1R12B, PPP1R12A, ACTG2 (includes EG:72), MSN	0.007762471	WASF1	0.356451133

**PI3K Signaling in B Lymphocytes**	ATF3, NFATC2, NFKB1, PRKCB	0.297166603	PLCB2, ATF3, ITPR1, BTK, PTPRC, PLCB4, RRAS2, NFAT5, PLCE1, CD180, VAV3, PLCG2, PIK3CG, SYK, LYN, AKT3, VAV1, PIK3AP1, PRKCB	0.291071712	PLCB2, PDIA3, ITPR1, PTPRC, BTK, PLCB4, PLCE1, RRAS2, CD180, PIK3CG, SYK, LYN, PIK3CB, VAV1, PIK3AP1, PIK3CD, PRKCB	0.034673685	PLCB2, PDIA3, ITPR1, PLCL2, PTPRC, BTK, CAMK2A, CAMK2D, PLCE1, CD180, BCL10, VAV3, PIK3CG, SYK, LYN, VAV1, PIK3AP1, PLCL1, CAMK2G, PRKCB	0.007413102	NA	#VALUE!
**LXR/RXR Activation**	IL1R2, IL18, CCL7, IL1B, NFKB1	0.035481339	IL1A, ECHS1, IL6, IL1R2, APOC1, IL18, CCL2, CCL7, IL1RN, IL1B, TLR3, LBP, PLTP, HMGCR, HADH	0.079432823	IL1R2, APOC1, IL1A, IL18, CCL2, CCL7, IL1RN, CD14, IL1B, IL6, HMGCR	0.024547089	SCD, MSR1, TNFRSF1A, CD36, IL6, ABCA1, IL18, CCL2, CCL7, IL1RN, CD14, HMGCR, TNFRSF11B	0.007413102	IL18	0.348337315
**B Cell Development**	NA	#VALUE!	NA	#VALUE!	HLA-DQB1, PTPRC, HLA-DMA, HLA-DOA, CD80, HLA-DRA, HLA-DQA1, CD86, HLA-DMB	0.005128614	IL7R, HLA-DQB1, PTPRC, HLA-DMA, CD80, HLA-DRA, HLA-DQA1, CD86, HLA-DMB	0.007079458	HLA-DRA, HLA-DQA1	0.060255959
**PKC? Signaling in T Lymphocytes**	CD4, FCER1G, NFATC2, NFKB1, LCP2	0.117489755	GRB2, CD4, MAP3K1, PIK3R3, NFAT5, RRAS2, CD80, PLCG2, PIK3CG, VAV3, FCER1G, VAV1, LCP2	0.592925325	HLA-DMA, HLA-DOA, GRB2, CD4, HLA-DQA1, HLA-DMB, HLA-DQB1, PIK3R3, RRAS2, CD80, PIK3CG, HLA-DRA, FCER1G, CD86, PIK3CB, VAV1, PIK3CD, LCP2	0.005495409	HLA-DMA, CD4, HLA-DQA1, HLA-DMB, MAP3K5 (includes EG:4217), HLA-DQB1, CAMK2A, CAMK2D, CD80, BCL10, PIK3CG, VAV3, HLA-DRA, FCER1G, CD86, VAV1, LCP2, CAMK2G	0.007079458	HLA-DRA, HLA-DQA1	0.139636836
**CTLA4 Signaling in CTL**	CD4, FCER1G, JAK2, LCP2	0.171001532	PIK3R3, PTPN6, CD80, GRB2, CD4, PIK3CG, SYK, FCER1G, AKT3, PPP2R1B, LCP2, PTPN22	0.474241985	HLA-DMA, HLA-DOA, PTPN6, GRB2, CD4, HLA-DQA1, HLA-DMB, HLA-DQB1, PIK3R3, CD80, PIK3CG, SYK, HLA-DRA, FCER1G, CD86, PIK3CB, PIK3CD, PPP2R1B, LCP2	0.000676083	HLA-DMA, AP1S2, CD4, HLA-DQA1, TRAT1, HLA-DMB, HLA-DQB1, CD80, PPM1J, PIK3CG, SYK, HLA-DRA, FCER1G, CD86, PPP2R1B, LCP2	0.00691831	HLA-DRA, HLA-DQA1	0.112201845
**Dendritic Cells and Natural Killer Cells**	IL18, TYROBP, CSF2, NFKB1	0.169044093	IL2RG, IL15RA, TYROBP, ACTA2, IL6, ACTG1, ITGAL, CSF2RB, IL18, HLA-A, CD80, FSCN1, TLR3	0.349140315	CSF2RB, IL2RG, IL18, CD80, TYROBP, ACTA2, HLA-DRA, CD86, CD83, IL6, ITGAL	0.112201845	IL2RG, IL12A, TYROBP, CD83, IL6, ACTG1, IL18, CAMK2A, CAMK2D, CD80, FSCN1, HLA-DRA, CD86, ACTG2 (includes EG:72), LTBR, CAMK2G	0.005623413	IL18, HLA-DRA	0.111943788
**TREM1 Signaling**	IL8, TREM1, IL18, CCL7, TYROBP, IL1B, JAK2, CSF2, NFKB1	2.00E-05	TLR1, IL8, TREM1, GRB2, TYROBP, LAT2, IL6, TLR2, NOD2, IL18, CCL2, CCL7, PLCG2, AKT3, IL1B, TLR3	0.012022644	TLR1, IL8, TREM1, GRB2, TYROBP, LAT2, CD83, IL6, TLR2, IL18, CCL2, CCL7, IL1B, CD86	0.000691831	TLR2, IL8, TLR1, TREM1, IL18, CCL2, CCL7, TYROBP, ITGA5, CD86, CD83, IL6	0.005623413	IL18	0.325087297
**Melatonin Signaling**	PRKCB	0.727779805	PLCB2, PLCB4, PLCE1, RORA, PRKCD, PLCG2, GNAO1, GNAI1, PRKAR2A, PRKCB	0.368977599	PLCB2, PLCB4, PLCE1, PDIA3, PRKCD, GNAO1, PRKAR2A, MAP2K3, RORB, PRKCB	0.054954087	PLCB2, PDIA3, PLCL2, CAMK2A, PLCE1, CAMK2D, RORA, PRKCD, GNAO1, MAP2K3, PLCL1, RORB, PRKCB, CAMK2G	0.003090295	NA	#VALUE!
**Regulation of Actin-based Motility by Rho**	NA	#VALUE!	MYLK, MYL9 (includes EG:10398), ARPC1B, PAK3, ARPC5L (includes EG:81873), ARPC2, ACTA2, MYL5, PPP1R12B, PPP1R12A, GSN	0.474241985	MYL9 (includes EG:10398), RHOG, PAK3, ARPC2, ACTA2, MYL5, PPP1R12B, PIP5K1B, GSN, MYL6B	0.137088177	ACTR2, ARPC1B, ARPC5L (includes EG:81873), MYL5, ARPC5, PIP5K1B, GSN, MYL9 (includes EG:10398), PAK1, RHOG, ARPC2, ARPC4, PPP1R12B, PPP1R12A, ACTG2 (includes EG:72), PPP1CA	0.003090295	WASF1	0.348337315
**iCOS-iCOSL Signaling in Th Cells**	CD4, FCER1G, NFATC2, NFKB1, LCP2	0.1	IL2RG, GRB2, CD4, CSK, ITPR1, PTPRC, PIK3R3, NFAT5, CD80, PIK3CG, FCER1G, AKT3, VAV1, LCP2	0.434510224	HLA-DMA, HLA-DOA, IL2RG, GRB2, CD4, CSK, HLA-DQA1, HLA-DMB, ITPR1, PIK3R3, HLA-DQB1, PTPRC, CD80, PIK3CG, HLA-DRA, FCER1G, PIK3CB, VAV1, PIK3CD, LCP2	0.000851138	HLA-DMA, IL2RG, CD4, CSK, HLA-DQA1, TRAT1, HLA-DMB, ITPR1, HLA-DQB1, PTPRC, CAMK2A, CAMK2D, CD80, PIK3CG, HLA-DRA, FCER1G, VAV1, LCP2, CAMK2G	0.002344229	HLA-DRA, HLA-DQA1	0.130918192
**Mitotic Roles of Polo-Like Kinase**	NA	#VALUE!	CDC25B, PLK2, CDC7, HSP90AA1, PPP2R1B, CHEK2	0.751622894	NA	#VALUE!	KIF23, CCNB1, CDC20, PTTG1, PRC1, CCNB3, CCNB2, PLK1, CDK1, CDC25B, PLK4, PPM1J, PPP2R1B, KIF11, CDC25A	0.00018197	NA	#VALUE!
**Dendritic Cell Maturation**	IL18, TYROBP, CD1A, FCER1G, IL1B, JAK2, CSF2, NFKB1	0.033113112	IL1A, LEP, TYROBP, MYD88, IL6, CREB5, FCGR1A, COL1A2, PIK3R3, TLR2, COL1A1, IL18, CD80, HLA-A, IL1RN, CD1A, PIK3CG, FSCN1, FCER1G, AKT3, IL1B, TLR3, COL3A1	0.247742206	HLA-DMA, HLA-DOA, IL1A, TYROBP, HLA-DQA1, HLA-DMB, CD83, IL6, FCGR1A, PIK3R3, HLA-DQB1, TLR2, IL18, CD80, IL1RN, CD1A, PIK3CG, HLA-DRA, FCER1G, CD86, IL1B, PIK3CB, PIK3CD, COL3A1	0.002884032	IL12A, HLA-DQA1, HLA-DMB, CD83, IL6, CREB5, FCGR1A, COL1A2, CD1A, PIK3CG, HLA-DRA, LTBR, STAT1, TNFRSF11B, HLA-DMA, TNFRSF1A, TYROBP, TLR2, HLA-DQB1, COL1A1, IL18, CD80, IL1RN, FSCN1, FCER1G, CD86, IFNAR1, COL3A1	0.000109648	COL1A2, COL1A1, IL18, HLA-DRA, HLA-DQA1, COL3A1	0.000446684
**T Helper Cell Differentiation**	IL18, FCER1G, IL18R1	0.230674719	IL6ST, IL2RG, IL18, TGFBR1, CD80, FCER1G, IL6, BCL6, IL18R1	0.493173804	HLA-DQB1, HLA-DMA, IL2RG, IL18, HLA-DOA, CD80, HLA-DRA, HLA-DQA1, FCER1G, CD86, HLA-DMB, IL6	0.013182567	HLA-DMA, IL2RG, IL12A, TGFBR1, TNFRSF1A, HLA-DQA1, HLA-DMB, IL6, HLA-DQB1, IL18, CD80, HLA-DRA, IL10RB, FCER1G, CD86, STAT1, TNFRSF11B	0.000109648	IL18, HLA-DRA, HLA-DQA1	0.017378008
**Actin Cytoskeleton Signaling**	GNA13, NCKAP1L, FGF19 (includes EG:9965)	0.707945784	ABI2, ARPC1B, PDGFA, ACTA2, MYL5, MYLK3, MYH11, PDGFC, FGF13, PTK2, MYLK, PIK3CG, LBP, FGF16, PXN, ARHGEF12, GRB2, ARPC5L (includes EG:81873), CSK, GSN, ACTG1, MYL9 (includes EG:10398), PIK3R3, RRAS2, TIAM2 (includes EG:26230), PAK3, VAV3, ARPC2, ARHGEF6, SSH3, PPP1R12B, PPP1R12A, VAV1, NCKAP1L	0.085113804	MYH10, ABI2, ACTA2, MYL5, MYH11, PIP5K1B, SSH1, PDGFC, FGF13, PIK3CG, EZR, SSH2, ITGA4, GRB2, CSK, ITGA2, GSN, MYL6B, MYL9 (includes EG:10398), PIK3R3, RRAS2, PAK3, ARPC2, SSH3, PPP1R12B, CD14, PIK3CB, VAV1, PIK3CD, NCKAP1L, PDGFD	0.000851138	ABI2, ARPC1B, F2R, DIAPH3, ARPC5, MYL5, MYH11, PIP5K1B, FGF13, IQGAP2, PAK1, PIK3CG, ARPC4, ACTG2 (includes EG:72), PPP1CA, SSH2, ITGA4, ACTR2, ARPC5L (includes EG:81873), CSK, ITGA5, GSN, ACTG1, TTN, MYL9 (includes EG:10398), TIAM2 (includes EG:26230), VAV3, ARPC2, PPP1R12B, PPP1R12A, CD14, VAV1, NCKAP1L, MSN	0.000102329	WASF1, ITGA4	0.291071712
**Fcg Receptor-mediated Phagocytosis**	ARF6, CSF2, FYB, LCP2, PRKCB	0.069183097	NAPEPLD, ARPC1B, NCF1C, ACTA2, FCGR1A, ARF6, RAB11B, PIK3CG, GPLD1, HCK, AKT3, FGR, PXN, ARPC5L (includes EG:81873), FYB, ACTG1, PIK3R3, ARPC2, PRKCD, SYK, VAV3, LYN, VAV1, LCP2, PRKCB	0.000870964	ACTA2, FYB, FCGR1A, PIK3R3, ARF6, PRKCD, ARPC2, EZR, PIK3CG, SYK, LYN, HCK, VAV1, LCP2, PRKCB	0.008709636	ACTR2, ARPC1B, ARPC5L (includes EG:81873), ARPC5, FYB, FCGR1A, ACTG1, MYO5A, HMOX1, PAK1, VAV3, SYK, PRKCD, ARPC2, PIK3CG, ARPC4, LYN, VAV1, ACTG2 (includes EG:72), LCP2, PRKCB	8.32E-05	NA	#VALUE!
**CD28 Signaling in TH**	CD4, FCER1G, NFATC2, NFKB1, LCP2	0.124738351	PTPN6, ARPC1B, ARPC5L (includes EG:81873), GRB2, CD4, CSK, MAP3K1, ITPR1, PIK3R3, PTPRC, NFAT5, CD80, SYK, PIK3CG, ARPC2, FCER1G, AKT3, VAV1, LCP2	0.176603782	HLA-DMA, HLA-DOA, PTPN6, GRB2, CD4, CSK, HLA-DQA1, HLA-DMB, ITPR1, PIK3R3, PTPRC, HLA-DQB1, CD80, SYK, PIK3CG, ARPC2, HLA-DRA, FCER1G, CD86, VAV1, PIK3CB, PIK3CD, LCP2	0.000199526	HLA-DMA, ACTR2, ARPC1B, ARPC5L (includes EG:81873), CD4, CSK, ARPC5, HLA-DQA1, HLA-DMB, ITPR1, PTPRC, HLA-DQB1, PAK1, CD80, BCL10, SYK, PIK3CG, ARPC2, HLA-DRA, ARPC4, FCER1G, CD86, VAV1, LCP2	8.32E-05	HLA-DRA, HLA-DQA1	0.142232879

Table S4B: List of genes from significantly enriched canonical pathways in grafted SMC.

Pathways	2H Symbols	2H P_value	12H Symbols	12H P_value	24H Symbols	24H P_value	7D Symbols	7D P_value	30D Symbols	30D P_value
**Mitotic Roles of Polo-Like Kinase**	NA	#VALUE!	NA	#VALUE!	NA	#VALUE!	KIF23,CCNB1,CDC20,PTTG1,PRC1,CDC7,CCNB2,PLK1,PPP2R5A,CDK1,CDC25B,PLK4,PLK2,PPP2R2B,KIF11,CHEK2	0.002344229	NA	#VALUE!
**Actin Cytoskeleton Signaling**	NA	#VALUE!	ABI2,ARPC1B,ARPC5,ACTA2,MYL5,MYLK3,MYH11,LIMK2,PIP5K1B,PDGFC,FGF13,PTK2,MYLK,IQGAP2,CYFIP2 (includes EG:26999),FGF10,FGF18,PIK3CG,PIK3R2,SSH2,FGF19 (includes EG:9965),PAK2,GRB2,CSK,ITGA5,GSN,MYL6B,ROCK1,MYL9 (includes EG:10398),TIAM2 (includes EG:26230),RRAS2,PAK3,CFL2,ARPC2,ARHGEF6,SSH3,PPP1R12B,CD14,PPP1R12A,VAV1,PIK3CB,PIK3CD,ACTN4,NCKAP1L,PDGFD	0.033884416	MYL9 (includes EG:10398),SHC1,TIAM2 (includes EG:26230),RRAS2,ARPC1B,GRB2,PPP1R12B,PIK3R2,PIP5K1B,NCKAP1L,GSN,FGF1	0.261818301	MYH10,ARPC1B,DIAPH3,ARPC5,LIMK2,PIP5K1B,MYH7B (includes EG:57644),FGF13,SHC1,FGF10,WASL (includes EG:8976),FGF18,ARPC4,ARPC1A,ACTG2 (includes EG:72),LBP,PPP1CA,ACTN1,ITGA4,MYH1,ACTR2,GRB2,CSK,MYH7,GSN,ACTG1,MYL1,TTN,FGF1,TIAM2 (includes EG:26230),ARPC2,PPP1R12B,CD14,MYH9,PPP1R12A,PIK3CB,NCKAP1L,MSN	0.001202264	NA	#VALUE!
**Reactive Oxygen in Macrophages**	NFKB1	0.256448404	NCF1C,PPP1R3C,ARG2,JAK2,MAPK13,NFKB1,PPP1R14B,SPI1,RHOG,NFKBIA,PIK3CG,HOXA10,CYBB,AKT3,PIK3R2,NOS2,NFKBIB,PRKCA,PTPN6,TNFRSF1A,IFNGR1,NFKB2,NCF4,PPP2R5A,IRF1,TLR2,FOS,PRKCI,RHOQ,PPP2R3A,PLCG2,PPP2R2B,NCF2,PPP1R12A,PIK3CB,PIK3CD,SIRPA,PRKCB	0.009772372	TNFRSF1A,PPP1R3C,PIK3R2,NOS2,PPP1R14B,PRKCB	0.666806769	PPP1R3C,RHOJ,ARG2,MAP3K5 (includes EG:4217),JAK2,PPP2R5A,TLR2,PRKCD,PPP2R2B,CYBB,PPP1R12A,PIK3CB,STAT1,PPP1CA,PRKD1,PRKCB	0.555904257	FOS	0.213304491
**IL-10 Signaling**	NFKB1	0.223872114	MAP2K6,SOCS3,IL1A,ARG2,MAP4K4,IL6,MAPK13,IL1R1,NFKB2,NFKB1,IL1R2,FOS,IL18,NFKBIA,IL1RN,CD14,IL1B,MAP2K3,NFKBIB	0.009332543	IL1R2,IL1RN,IL6	0.566239289	IL18,IL10RB,CD14,ARG2,MAP4K4,IL1R1,LBP,IL6	0.405508535	FOS	0.166341265
**NF-kB Signaling**	NFKB1	0.250034536	MAP2K6,TLR1,IL1A,BMP4,UBE2N,TNFAIP3,MAP4K4,NGF,NFKB1,IL1R2,NFKBIA,BMPR1A,PIK3CG,PDGFRA,AKT3,LTBR,TRAF5,PIK3R2,NFKBIB,TNFRSF1A,MYD88,NFKB2,IL1R1,TLR2,IL18,RRAS2,GHR,CD40,IL1RN,PLCG2,FCER1G,PRKACA,IL1B,PIK3CB,PIK3CD,INSR,PRKCB	0.008912509	IL1R2,GHR,RRAS2,IL1RN,TNFRSF1A,BMP2,PDGFRA,PIK3R2,PRKCB	0.261818301	TLR1,MYD88,MAP4K4,IL1R1,MALT1,TLR2,IL18,GHR,CD40,PIK3CB,EIF2AK2,LTBR,TNFSF13B,IRAK4,PRKCB	0.592925325	NA	#VALUE!
**CCR3 Signaling in Eosinophils**	NA	#VALUE!	CCR3,PLCB2,GNB5,LIMK2,MAPK13,MYLK,GNB4,GNG11,GNB3,PIK3CG,PIK3R2,PRKCA,PAK2,ITPR2,GNAI1,ITPR1,ROCK1,PLA2G4A,PLCB4,PRKCI,RRAS2,PAK3,CFL2,PPP1R12B,PPP1R12A,PIK3CB,PIK3CD,GNG2,OPN1SW,PRKCB	0.00724436	RRAS2,ITPR2,PPP1R12B,PIK3R2,ITPR1,PRKCB	0.420726628	PLA2G10,GNAI1,CCL24,LIMK2,ITPR1,PLCB4,PRKCD,PPP1R12B,PPP1R12A,PIK3CB,GNG2,PPP1CA,PRKD1,PRKCB	0.331131121	NA	#VALUE!
**Renin-Angiotensin Signaling**	NFKB1	0.225423921	ADCY4,SHC3,JAK2,MAPK13,NFKB1,PTK2,CCL2,ADCY5,PIK3CG,PIK3R2,PRKCA,PTPN6,PAK2,GRB2,ITPR2,ADCY6,NFKB2,ITPR1,FOS,PRKCI,RRAS2,PAK3,PLCG2,PRKACA,PIK3CB,PIK3CD,PTGER2,PRKCB,PRKAR1A	0.00724436	SHC1,RRAS2,CCL2,GRB2,ITPR2,PIK3R2,ITPR1,PRKCB	0.174984669	SHC1,CCL2,GRB2,PRKCD,SHC3,PIK3CB,JAK2,ITPR1,STAT1,ADCY7,PRKD1,PRKCB	0.472063041	FOS	0.171790839
**RAR Activation**	NFKB1	0.263026799	RDH10,ADCY4,SMAD3,NR2F2,JAK2,MAPK13,NFKB1,VEGFA,NR2F1,PRMT1,ALDH1A1,ADCY5,PIK3CG,RARB,AKT3,SORBS3,NR2F6,PIK3R2,MAPKAPK2,ZBTB16,CITED2,MMP1 (includes EG:4312),PRKCA,SMAD9,CSK,ADCY6,NFKB2,CSF2RB,FOS,PRKCI,DHRS9,SMARCA2,DUSP1,PRKACA,TGFB3,PIK3CB,PIK3CD,RBP4,PRKAR1A,PPARGC1A,PRKCB	0.00724436	ALDH1A1,SMARCA2,TGFB3,SNW1,PIK3R2,CITED2,PPARGC1A,PRKCB	0.442588372	RDH10,CSK,MAP3K5 (includes EG:4217),JAK2,VEGFA,ALDH1A1,RARA,PRKCD,CRABP2,TGFB3,PIK3CB,ADCY7,ZBTB16,PRKD1,PRKCB,PPARGC1A	0.645654229	FOS	0.216271852
**ILK Signaling**	NFKB1	0.272270131	MAP2K6,ACTA2,MYL5,MYH11,HIF1A,ITGB8,CREB5,NFKB1,PPP1R14B,ITGB7,PTK2,VEGFA,MYC,TGFB1I1,RHOG,FLNA,PIK3CG,AKT3,PIK3R2,ITGB4,NOS2,ITGB5,PARVA,FBLIM1,TNFRSF1A,NACA,NFKB2,MYL6B,PPP2R5A,ITGB3,MYL9 (includes EG:10398),FOS,RHOQ,PPP2R3A,CFL2,IRS1,PPP2R2B,ARHGEF6,PIK3CB,RSU1,RPS6KA5,PIK3CD,PTGS2,ACTN4	0.007079458	MYL9 (includes EG:10398),MYC,TNFRSF1A,RPS6KA5,PIK3R2,PTGS2,NOS2,PPP1R14B,ITGB7	0.420726628	MYH10,MYH7,RHOJ,CREB5,MYH7B (includes EG:57644),ACTG1,PPP2R5A,MYL1,VEGFA,FLNC,FLNA,SNAI2,PPP2R2B,MYH9,PIK3CB,RPS6KA5,ACTG2 (includes EG:72),ITGB4,ACTN1,MYH1	0.404575892	FOS	0.223357222
**Macropinocytosis Signaling**	NA	#VALUE!	ITGA5,ITGB8,NGF,PDGFC,ITGB7,ITGB3,ARF6,RRAS2,PRKCI,PLCG2,PIK3CG,CD14,PIK3CB,PIK3CD,ITGB4,PIK3R2,ACTN4,PDGFD,ITGB5,PRKCA,PRKCB	0.00676083	RRAS2,PIK3R2,ITGB7,PRKCB	0.423642966	PRKCD,CD14,PIK3CB,ITGB4,PRKD1,PRKCB	0.76913044	NA	#VALUE!
**Acute Phase Response Signaling**	SERPINE1,NFKB1	0.223872114	MAP2K6,SOCS3,IL1A,ITIH3,FTL,TCF4,NOLC1,AMBP,C9,MAPK13,JAK2,IL6,NFKB1,NFKBIA,SOD2,PIK3CG,CFB,AKT3,SERPINA1,PIK3R2,NFKBIB,SERPINE1,IL1RAP,C4BPB,MYD88,GRB2,TNFRSF1A,C1S,IL1R1,NFKB2,HMOX2,FOS,KLKB1,IL18,RRAS2,IL1RN,IL1B,PIK3CB,MAP2K3,PIK3CD,A2M,RBP4	0.00676083	SHC1,ITIH3,RRAS2,GRB2,IL1RN,TNFRSF1A,SERPINF1,SERPINA1,PIK3R2,IL6,SERPINE1,A2M	0.141905752	ITIH3,GRB2,MYD88,C1S,C9,IL6,JAK2,IL1R1,MAP3K5 (includes EG:4217),KLKB1,SHC1,IL18,TF,CRABP2,CFB,SERPINA1,PIK3CB,LBP,SERPINE1	0.404575892	FOS,C1S	0.12246162
**Relaxin Signaling**	NFKB1	0.233345806	ADCY4,GNB5,PDE1A,NFKB1,PDE4D,VEGFA,GNB4,GNG11,GNB3,NFKBIA,ADCY5,GNAT2,PIK3CG,AKT3,PIK3R2,NOS2,NFKBIB,GUCY1B3,GUCY1A3,GNAI1,ADCY6,PDE4B,NFKB2,FOS,RXFP1,GNAO1,PRKACA,PIK3CB,PDE8B,PIK3CD,GNG2,NPR2,GNAL,PRKAR1A	0.006606934	GNAO1,PDE8B,PIK3R2,PDE4B,NOS2,PDE4D	0.500034535	NA	#VALUE!	FOS	0.187931682
**P2Y Purigenic Receptor Sig**	NFKB1	0.225423921	PLCB2,ADCY4,GNB5,NFKB1,CREB5,MYC,GNB4,GNG11,PLCE1,GNB3,ADCY5,PIK3CG,AKT3,PIK3R2,PLCL1,PRKCA,GNAI1,ADCY6,NFKB2,ITGB3,FOS,PLCB4,PRKCI,RRAS2,PLCG2,PRKACA,PIK3CB,PIK3CD,GNG2,OPN1SW,PRKCB,PRKAR1A	0.006606934	NA	#VALUE!	PLCB4,PLCE1,PRKCD,GNAI1,PIK3CB,GNG2,CREB5,ADCY7,PRKD1,PRKCB	0.805563908	FOS	0.176603782
**Dendritic Cell Maturation**	NFKB1	0.261818301	B2M,IL1A,IL12A,ICAM1,LEP,MAPK13,JAK2,IL6,CREB5,NFKB1,FCGR1A,COL1A2,NFKBIA,HLA-A,CD1A,PIK3CG,HLA-DRA,AKT3,LY75,LTBR,PIK3R2,NFKBIB,TYROBP,TNFRSF1A,MYD88,IL15,NFKB2,STAT4,TLR2,COL1A1,IL18,CD80,CD40,IL1RN,FSCN1,FCER1G,IL1B,PIK3CB,PIK3CD,IFNAR1,COL3A1	0.006606934	IL1RN,TNFRSF1A,TYROBP,CD1A,FSCN1,PIK3R2,IL6,FCGR1A	0.427562886	B2M,IL12A,ICAM1,HLA-DQA1,HLA-DMB,JAK2,IL6,CREB5,FCGR1A,COL1A2,HLA-A,HLA-DRA,COL10A1,LTBR,STAT1,HLA-DMA,TYROBP,MYD88,TLR2,STAT4,COL1A1,IL18,CD40,CD80,FSCN1,CD86,PIK3CB,STAT2,IFNAR1,COL3A1	0.002570396	COL1A2,COL1A1,CD80,FCGR1A	0.005248075
**IL-1 Signaling**	NFKB1	0.223872114	MAP2K6,IL1A,ADCY4,GNB5,MAPK13,NFKB1,GNB4,GNG11,GNB3,NFKBIA,ADCY5,GNAT2,NFKBIB,IL1RAP,MYD88,GNAI1,ADCY6,IL1R1,NFKB2,FOS,GNAO1,PRKACA,MAP2K3,GNG2,GNAL,IRAK2,PRKAR1A	0.005754399	NA	#VALUE!	NA	#VALUE!	FOS	0.167494288
**CXCR4 Signaling**	CXCR4	0.256448404	PLCB2,CD4,ADCY4,MYL5,GNB5,PTK2,GNB4,RHOG,GNB3,GNG11,ADCY5,PIK3CG,GNAT2,AKT3,PIK3R2,PRKCA,PAK2,CXCR4,ITPR2,EGR1,ADCY6,GNAI1,ITPR1,MYL6B,ROCK1,MYL9 (includes EG:10398),FOS,PLCB4,PRKCI,RRAS2,RHOQ,PAK3,GNAO1,LYN,PIK3CB,PIK3CD,GNG2,OPN1SW,GNAL,PRKCB	0.005128614	MYL9 (includes EG:10398),RRAS2,CXCR4,ITPR2,CD4,EGR1,GNAO1,LYN,PIK3R2,ITPR1,PRKCB	0.149279441	GNAI1,RHOJ,ITPR1,MYL1,PLCB4,PRKCD,GNAO1,LYN,PIK3CB,GNG2,ADCY7,PRKD1,PRKCB	0.805563908	FOS,EGR1	0.10964782
**Chemokine Signaling**	CCL4,CXCR4	0.126182753	CCR3,PLCB2,CAMK1D,CXCR4,GNAI1,LIMK2,MAPK13,PTK2,FOS,PLCB4,CAMK2D,RRAS2,CCL4,CCL2,CCL7,PIK3CG,PLCG2,PPP1R12B,PPP1R12A,OPN1SW,PRKCA,CAMK2G,PRKCB	0.005128614	CCL4,RRAS2,CCL2,CCL7,CXCR4,PPP1R12B,PRKCB	0.128233058	PLCB4,CCL4,CAMK2D,CCL2,GNAI1,PPP1R12B,PPP1R12A,CCL24,LIMK2,PPP1CA,PRKCB	0.244906324	FOS	0.166341265

**TREM1 Signaling**	NFKB1	0.223872114	TLR1,IL8,TREM1,ICAM1,TYROBP,GRB2,LAT2,ITGA5,IL6,JAK2,NFKB2,NFKB1,TLR2,IL18,CCL7,CCL2,CD40,PLCG2,AKT3,IL1B	0.005011872	IL8,CCL2,CCL7,GRB2,TYROBP,IL6	0.135831345	TLR2,IL8,TLR1,IL18,ICAM1,CCL2,CD40,GRB2,TYROBP,CD86,JAK2,IL6	0.060255959	NA	#VALUE!
**PI3K in B Lymphocytes**	NFKB1	0.236047823	CD81,PLCB2,NFKB1,PTPRC,PLCE1,CAMK2D,NFKBIA,PPP3CB,PIK3CG,AKT3,PIK3R2,NFKBIB,PLCL1,CR2,ATF1,ITPR2,ITPR1,NFKB2,BTK,FOS,PLCB4,PRKCI,RRAS2,CD180,DAPP1,CD40,SYK,IRS1,PLCG2,LYN,NFATC2,PIK3CB,PIK3AP1,VAV1,PIK3CD,CAMK2G,PRKCB	0.005011872	PTPRC,RRAS2,ITPR2,SYK,LYN,PIK3R2,ITPR1,PRKCB	0.309029543	MALT1,ITPR1,CD79A,PTPRC,BTK,PLCB4,PLCE1,CAMK2D,CD180,CD40,LYN,PIK3CB,PRKCB	0.669884609	FOS	0.193642196
**IL-6 Signaling**	NFKB1	0.223872114	MAP2K6,HSPB3,IL1A,MAP4K4,IL6,JAK2,MAPK13,NFKB1,IL1R2,NFKBIA,NFKBIB,MAPKAPK2,IL8,GRB2,TNFRSF1A,IL1R1,NFKB2,COL1A1,FOS,IL18,RRAS2,IL1RN,IL1B,CD14,MAP2K3,TNFAIP6,A2M	0.005011872	IL1R2,IL8,SHC1,RRAS2,GRB2,IL1RN,TNFRSF1A,IL6,TNFAIP6,A2M	0.066069345	COL1A1,IL8,SHC1,IL18,GRB2,SRF,CD14,MAP4K4,IL1R1,LBP,JAK2,IL6	0.299916252	COL1A1,FOS	0.0616595
**Thrombin Signaling**	NFKB1	0.276057786	PLCB2,CAMK1D,ADCY4,MYL5,GNB5,MAPK13,PTK2,MYLK,GNB4,RHOG,GNB3,PLCE1,CAMK2D,ADCY5,PIK3CG,PLCL1,ITPR2,ITPR1,NFKB2,MYL6B,MYL9 (includes EG:10398),RHOQ,PLCG2,GNAO1,ARHGEF6,PPP1R12B,PPP1R12A,PIK3CD,GNG2,OPN1SW,GNAL,CAMK2G,F2RL2,NFKB1,GNG11,GNAT2,AKT3,GATA6,PIK3R2,PRKCA,GRB2,GNAI1,ADCY6,ROCK1,PLCB4,PRKCI,RRAS2,PIK3CB,PRKCB	0.003548134	MYL9 (includes EG:10398),SHC1,RRAS2,GRB2,ITPR2,GNAO1,PPP1R12B,GATA6,PIK3R2,ITPR1,PRKCB	0.261818301	F2RL2,GRB2,ARHGEF15,GNAI1,RHOJ,ITPR1,MYL1,SHC1,PLCB4,CAMK2D,PLCE1,PRKCD,GNAO1,PPP1R12B,PPP1R12A,PIK3CB,ARHGEF3,GNG2,ADCY7,PRKD1,PRKCB	0.404575892	NA	#VALUE!
**fMLP Signaling in Neutrophils**	NFKB1	0.225423921	PLCB2,ARPC1B,NCF1C,ARPC5,GNB5,NFKB1,GNB4,GNG11,GNB3,NFKBIA,PPP3CB,PIK3CG,CYBB,PIK3R2,NFKBIB,PRKCA,NOX4,ITPR2,GNAI1,ITPR1,NFKB2,PLCB4,PRKCI,RRAS2,ARPC2,NCF2,NFATC2,PIK3CB,PIK3CD,GNG2,OPN1SW,PRKCB	0.003548134	RRAS2,ARPC1B,ITPR2,PIK3R2,ITPR1,PRKCB	0.414954043	ACTR2,ARPC1B,ARPC5,GNAI1,ITPR1,PLCB4,PRKCD,ARPC2,ARPC4,CYBB,ARPC1A,PIK3CB,GNG2,PRKD1,PRKCB	0.244906324	NA	#VALUE!
**IL-8 Signaling**	NFKB1	0.264850014	ANGPT2,NAPEPLD,PLCB2,ICAM1,GNB5,MAP4K4,LIMK2,NFKB1,BCL2,VEGFA,PTK2,GNB4,GNG11,GNB3,RHOG,CXCR2,PIK3CG,GPLD1,CYBB,AKT3,PIK3R2,NFKBIB,CR2,PRKCA,IL8,NOX4,PAK2,GNAI1,HBEGF,MMP2,CSTB,ROCK1,ITGAM,PRKCI,RRAS2,RHOQ,NCF2,KDR,PIK3CB,PIK3CD,PTGS2,GNG2,OPN1SW,PRKCB,IRAK2	0.003548134	IL8,ANGPT2,RRAS2,CXCR2,GPLD1,PIK3R2,PTGS2,PRKCB	0.456036916	IL8,VCAM1,ICAM1,GNAI1,LIMK2,RHOJ,MAP4K4,CSTB,BCL2,VEGFA,BCL2L1,CCND2,PRKCD,KDR,CYBB,PIK3CB,GNG2,IRAK4,PRKD1,PRKCB	0.325087297	NA	#VALUE!
**Endothelin-1 Signaling**	NA	#VALUE!	PLCB2,ADCY4,PLA2R1,SHC3,MAPK13,MYC,PLCE1,LCAT,ECE2,ADCY5,PIK3CG,PLCL1,PROCA1,GUCY1B3,CASP10,GUCY1A3,ITPR2,ITPR1,GAB1,PLCG2,GNAO1,PIK3CD,PTGER2,GNAL,OPN1SW,NAPEPLD,CASP4,PRDX6,GNAT2,GPLD1,PIK3R2,CASP8,NOS2,PRKCA,EDNRB,GRB2,GNAI1,ADCY6,MAPK6,LAMB2,PLA2G4A,FOS,PLCB4,PRKCI,RRAS2,EDNRA,PIK3CB,PTGS2,PRKCB	0.000316228	GRB2,ITPR2,ITPR1,MYC,LAMB2,SHC1,RRAS2,GAB1,GPLD1,GNAO1,PIK3R2,PTGS2,NOS2,PRKCB	0.066069345	PLA2G10,SHC3,WISP2,CASP6,SHC1,PLCE1,EDN1,CASP8,PRKD1,CASP10,EDNRB,GUCY1A3,CASP3,GRB2,YWHAZ,GNAI1,MAPK6,ITPR1,LAMB2,PLCB4,PRKCD,GNAO1,GUCY1A2,PIK3CB,ADCY7,PRKCB	0.060255959	FOS	0.218272991
