# Supplementary material for: Temporal Network Based Analysis of Cell Specific Vein Graft Transcriptome Defines Key Pathways and Hub Genes in Implantation Injury
Source: PLoS One. 2012 Jun 15;7(6):e39123. doi: 10.1371/journal.pone.0039123 (PMC3376111; doi:10.1371/journal.pone.0039123)
Supplement: Table S5 — List of genes from significantly enriched disease pathways in vein grafts at different time points. A) EC, B) SMC. This analysis is performed using Ingenuity Pathway Analysis System and Pathways with multiple test (Holm–Bonferroni method) corrected P value <0.01 was considered significant. (PDF) [file pone.0039123.s015.pdf]

Table S5A: List of genes from significantly enriched disease pathways in EC vein grafts at different time points.

Disease Pathways	2H Symbols	2H P_value	12H Symbols	12H P_value	24H Symbols	2H P_value2	7D Symbols	7D P_value	30D Symbols	30D P_value
**Atherosclerosis Signaling**	IL8, SELE, IL18, IL1B, NFKB1	0.081283052	IL8, CCR3, SELE, IL1A, PDGFA, CXCR4, IL6, PDGFC, PRDX6, LAMB2, COL1A2, COL1A1, PLA2G4A, IL18, SELP, CCL2, IL1RN, IL1B, MMP1 (includes EG:4312), COL3A1	0.0616595	IL8, CCR3, SELE, IL1A, VCAM1, CXCR4, IL6, PDGFC, LAMB2, IL18, CCL2, IL1RN, IL1B, PDGFD, ITGA4, COL3A1	0.00724436	IL8, SELE, VCAM1, MSR1, CXCR4, CD36, IL6, LAMB2, COL1A2, COL1A1, IL18, CCL2, SELP, IL1RN, ITGA4, COL3A1	0.00851138	COL1A2, COL1A1, IL18, COL3A1, ITGA4	0.000446684
**Graft-versus-Host Disease Signaling**	IL18, FCER1G, IL1B	0.124738351	IL1A, IL18, HLA-A, CD80, IL1RN, FCER1G, IL1B, IL6	0.276694165	HLA-DMA, IL1A, HLA-DOA, HLA-DQA1, HLA-DMB, IL6, HLA-DQB1, IL18, CD80, IL1RN, HLA-DRA, FCER1G, CD86, IL1B	0.000112202	HLA-DQB1, HLA-DMA, IL18, CD80, IL1RN, HLA-DRA, HLA-DQA1, FCER1G, CD86, HLA-DMB, IL6	0.003090295	IL18, HLA-DRA, HLA-DQA1	0.005888437
**Systemic Lupus Erythematosus Signaling**	IL18, FCER1G, NFATC2, IL1B	0.304088503	PTPN6, IL1A, CREM, GRB2, C9, IL6, FCGR1A, PIK3R3, PTPRC, IL18, RRAS2, NFAT5, CD80, HLA-A, IL1RN, PIK3CG, PLCG2, FCER1G, LYN, AKT3, IL1B, TNFSF13B	0.176603782	PTPN6, IL1A, CREM, GRB2, IL6, FCGR1A, PTPRC, PIK3R3, IL18, RRAS2, CD80, IL1RN, PIK3CG, FCER1G, LYN, C7, CD86, IL1B, PIK3CB, PIK3CD	0.008709636	PTPRC, IL18, CD80, IL1RN, PIK3CG, FCER1G, LYN, CD86, C6, IL6, FCGR1A, TNFSF13B	0.461317575	IL18	0.39994475
**Type I Diabetes Mellitus Signaling**	SOCS3, FCER1G, IL1B, JAK2, NFKB1, BCL2	0.03801894	SOCS3, ICA1, HLA-A, CD80, MYD88, FCER1G, CYCS (includes EG:54205), IL1B, HSPD1, CASP8, CPE, IL1RAP	0.610942025	HLA-DMA, HLA-DOA, ICA1, HLA-DQA1, HLA-DMB, HLA-DQB1, CD80, HLA-DRA, FCER1G, IL1B, CYCS (includes EG:54205), CD86, MAP2K3, IL1RAP, CPE	0.021379621	HLA-DMA, IL12A, TNFRSF1A, HLA-DQA1, HLA-DMB, MAP3K5 (includes EG:4217), BCL2, HLA-DQB1, CD80, HLA-DRA, FCER1G, CD86, MAP2K3, CASP8, STAT1, CPE, TNFRSF11B	0.007762471	HLA-DRA, HLA-DQA1	0.130918192
**Breast Cancer Regulation by Stathmin1**	CDKN1A, GNA13, GNG2, PRKCB	0.477529274	PLCB2, PPP1R3C, GNB5, TUBB, PPP1R14B, GNB3, GNG11, PIK3CG, RB1CC1, ARHGEF3, ARHGEF12, TUBB2C, GRB2, GNAI1, TUBA4A, PRKAR2A, ARHGEF17, ITPR1, PIK3R3, PLCB4, RRAS2, PRKCD, CDKN1A, ARHGEF6, PPP1R12A, GNG2, CDKN1B, PPP2R1B, PRKCB	0.125025903	PLCB2, GRB2, ARHGEF15, PPP1R3C, PRKAR2A, ITPR1, PPP1R14B, GNB1, PIK3R3, PLCB4, RRAS2, GNG11, ADCY5, PIK3CG, PRKCD, CDKN1A, PIK3CB, PIK3CD, ARHGEF3, GNG2, CDKN1B, PPP2R1B, PRKCB	0.016595869	PLCB2, PPP1R3C, TUBB, PPP1R14B, PAK1, STMN1, CAMK2D, CAMK2A, ADCY5, PPM1J, PIK3CG, ARHGEF3, PPP1CA, CCNE2, ARHGEF15, TUBA4A, ITPR1, CDK1, CCNE1, PRKCD, CDKN1A, PPP1R12A, GNG2, PPP2R1B, PRKCB, CAMK2G	0.003981072	CDKN1A	0.432513831
**Glioma Invasiveness Signaling**	RND3, PLAUR, PLAU	0.171001532	PTK2, PIK3R3, TIMP3, RRAS2, TIMP1, PIK3CG, PLAUR, TIMP2	0.434510224	PIK3R3, TIMP3, RHOG, RRAS2, TIMP1, PIK3CG, PLAUR, MMP2, PIK3CB, PIK3CD, ITGB5, TIMP2	0.003630781	TIMP3, RHOG, F2R, TIMP1, PIK3CG, HMMR, ITGAV, PLAUR, MMP2	0.060255959	NA	#VALUE!
**HER-2 Signaling in Breast Cancer**	CDKN1A, NRG1, PRKCB	0.250034536	GRB2, NRG1, MDM2, ITGB8, CCND1, ITGB7, AREG, PIK3R3, RRAS2, PRKCD, PIK3CG, CDKN1A, AKT3, CDKN1B, PRKCB	0.120781384	GRB2, NRG1, MDM2, MMP2, ITGB7, PIK3R3, RRAS2, PRKCD, PIK3CG, CDKN1A, PIK3CD, PIK3CB, CDKN1B, ITGB5, PRKCB	0.002754229	CCNE2, CCNE1, PRKCD, PIK3CG, CDKN1A, NRG1, MMP2, MAP3K5 (includes EG:4217), PRKCB	0.216271852	CDKN1A	0.348337315
**T Cell & B Cell Signal in Rheumatoid Arthritis**	IL18, FCER1G, IL1B, CSF2, NFKB1	0.054954087	TLR2, TLR1, IL1A, IL18, SPP1, CD80, IL1RN, FCER1G, IL1B, IL6, TLR3, TNFSF13B	0.364753947	TLR1, HLA-DMA, IL1A, HLA-DOA, SPP1, HLA-DQA1, HLA-DMB, IL6, HLA-DQB1, TLR2, IL18, CD80, IL1RN, HLA-DRA, FCER1G, CD86, IL1B	0.001023293	TLR1, HLA-DMA, SPP1, IL12A, HLA-DQA1, HLA-DMB, IL6, HLA-DQB1, TLR2, IL18, CD80, IL1RN, HLA-DRA, FCER1G, CD86, TNFSF13B	0.003090295	IL18, SPP1, HLA-DRA, HLA-DQA1	0.002570396
**AML Signaling**	CSF3R, MYC, CSF2RA (includes EG:1438), NFKB1	0.124738351	CSF3R, GRB2, CCND1, SPI1, EIF4EBP1, KITLG, PIK3R3, CSF2RB, RRAS2, CSF2RA (includes EG:1438), PIK3CG, AKT3, JUP, RUNX1, TCF7L2 (includes EG:6934)	0.127350308	CSF3R, GRB2, CSF1R, EIF4EBP1, KITLG, PIK3R3, CSF2RB, RRAS2, CSF2RA (includes EG:1438), PIM1, PIK3CG, PIK3CB, PIK3CD, MAP2K3, JUP, RUNX1, TCF7L2 (includes EG:6934)	0.000691831	NA	#VALUE!	NA	#VALUE!
**Hepatic Fibrosis /Stellate Cell Activation**	IL1R2, IL8, IL1B, NFKB1, BCL2	0.155238701	IL1A, TGFBR1, LEP, PDGFA, ACTA2, MYL5, MYH11, IL6, IL1R2, COL1A2, IGF1, CCL2, TIMP1, IGF1R, LBP, MMP1 (includes EG:4312), TIMP2, IL8, EDNRB, FGFR2, IGFBP5, MYL9 (includes EG:10398), COL1A1, KDR, TGFB3, EDNRA, IL1B, A2M, COL3A1	0.003548134	MYH10, IL1A, CCR5, SMAD3, ACTA2, MYL5, MYH11, IL6, IL1R2, CCL2, TIMP1, HGF, ECE1, TIMP2, IL8, VCAM1, IGFBP5, MMP2, MYL6B, MYL9 (includes EG:10398), KDR, CD14, EDNRA, IL1B, A2M, COL3A1	7.59E-05	IL8, CCR5, VCAM1, TGFBR1, EDNRB, TNFRSF1A, MYL5, MYH11, MMP2, IL6, BCL2, COL1A2, MYL9 (includes EG:10398), COL1A1, CCL2, TIMP1, KDR, PDGFRA, CD14, EDNRA, STAT1, IFNAR1, TNFRSF11B, COL3A1	0.000128825	COL1A2, COL1A1, COL3A1	0.060255959
**Endothelial Cells in Rheumatoid Arthritis**	IL8, SOCS3, SELE, DAAM1, JAK2, NFKB1, IL18R1, MYC, IL1R2, IL18, C5AR1, IL1B, NFATC2, CSF2, IRAK2, PRKCB	0.001096478	IL6ST, IL18RAP, SOCS3, TLR1, PLCB2, IL1A, PDGFA, IL6, CREB5, FCGR1A, CCND1, PDGFC, IL18R1, IL1R2, C5AR1, NFAT5, PLCE1, CCL2, DKK3, PIK3CG, AKT3, DKK2, TLR3, MAPKAPK2, TNFSF13B, MMP1 (includes EG:4312), ADAMTS4, SFRP4, IL8, SELE, MYD88, TLR2, PIK3R3, PLCB4, IL18, RRAS2, IL1RN, PRKCD, PLCG2, GNAO1, FZD3, FZD6, IL1B, TCF7L2 (includes EG:6934), PRSS35, PRKCB	0.128528666	IL18RAP, TLR1, IL1A, PLCB2, PDIA3, IL6, FCGR1A, PDGFC, IL1R2, PLCE1, C5AR1, CCL2, DKK3, PIK3CG, SFRP5, SFRP4, IL8, VCAM1, SELE, DAAM1, PIK3R3, TLR2, PLCB4, IL18, RRAS2, IL1RN, PRKCD, GNAO1, IL1B, PIK3CB, MAP2K3, PIK3CD, PDGFD, TCF7L2 (includes EG:6934), PRSS35, PRKCB	0.013803843	IL18RAP, TLR1, PLCB2, SFRP2, PDIA3, IL6, CREB5, FCGR1A, C1R, PLCE1, CAMK2D, CAMK2A, CCL2, PIK3CG, SFRP5, LTBR, PLCL1, TNFSF13B, TNFRSF11B, IL8, SFRP4, VCAM1, SELE, TNFRSF1A, C1S, DAAM1, PLCL2, TLR2, IL18, IL1RN, PRKCD, GNAO1, MAP2K3, LRP1, TCF7L2 (includes EG:6934), CAMK2G, PRKCB	0.009549926	IL18, C1S	0.349140315

Table S5B: List of genes from significantly enriched disease pathways in SMC vein grafts at different time points.

Disease Pathways	2H Symbols	2H P_value	12H Symbols	12H P_value	24H Symbols	24H P_value	7D Symbols	7D P_value	30D Symbols	30D P_value
**AML Signaling**	NFKB1	0.223872114	CSF3R,MAP2K6,TCF4,GRB2,NFKB2,TCF7L1,NFKB1,SPI1,MYC,KITLG,CSF2RB,CCNA1,RRAS2,PIM1,CSF2RA (includes EG:1438),PIK3CG,AKT3,PIK3CB,MAP2K3,PIK3CD,PIK3R2,RUNX1,TCF7L2 (includes EG:6934)	0.009772372	CSF3R,KITLG,MYC,RRAS2,GRB2,RUNX1,PIK3R2,TCF7L2 (includes EG:6934)	0.104712855	NA	#VALUE!	NA	#VALUE!
**Molecular Mechanisms of Cancer**	NFKB1,BIRC3	0.223872114	PLCB2,BMP4,ADCY4,SMAD3,CDKN2C,MAPK13,MYC,PTK2,CAMK2D,RHOG,ADCY5,PIK3CG,NFKBIB,BIRC3,CASP10,SMAD9,ARHGEF17,NFKB2,RALBP1,RASGRF2,RHOQ,GAB1,CDK4,IRS1,GNAO1,ARHGEF6,TGFB3,PRKACA,CYCS (includes EG:54205),PIK3CD,MAP2K3,FZD5,CFLAR,GNAL,CAMK2G,MAP2K6,TCF4,PA2G4,HIF1A,JAK2,NFKB1,BCL2,CDC25B,MAPKSP1,NFKBIA,NLK,BMPR1A,GNAT2,E2F5,AKT3,PIK3R2,CASP8,PRKCA,PRKDC,PAK2,GRB2,ADCY6,GNAI1,MDM2,FOS,PLCB4,RRAS2,PRKCI,FZD4,PAK3,CDKN1A,PIK3CB,CDKN1B,BIRC2,PRKAR1A,PSEN1,PRKCB	0.008128305	GRB2,BMP2,MYC,SHC1,MAPKSP1,RRAS2,GAB1,GNAO1,CDKN1A,TGFB3,PIK3R2,CDKN1B,BIRC3,PRKCB	0.50234259	TGFBR1,CDKN2C,MAP3K5 (includes EG:4217),JAK2,BCL2,CDC25B,SHC1,CASP6,HHAT,NLK,CAMK2D,FANCD2,BID,ARHGEF3,CASP8,BIRC3,PRKD1,CHEK2,CASP10,CCNE2,CASP3,GRB2,ARHGEF15,GNAI1,RHOJ,ARHGEF17,AURKA,BCL2L1,PLCB4,CCND2,PRKCD,CDKN1A,GNAO1,TGFB3,PIK3CB,CDKN1B,ADCY7,BCL2L11,PRKCB	0.197242274	FOS	0.365594792
**Breast Cancer Regulation by Stathmin1**	NA	NA	PLCB2,CAMK1D,PPP1R3C,ADCY4,GNB5,LIMK2,TUBB,PPP1R14B,GNB4,STMN1,GNB3,GNG11,CAMK2D,ADCY5,PIK3CG,E2F5,PIK3R2,PRKCA,ITPR2,TUBB2C,GRB2,ADCY6,GNAI1,TUBA4A,ARHGEF17,ITPR1,PPP2R5A,ROCK1,PLCB4,PRKCI,RRAS2,PPP2R3A,PPP2R2B,ARHGEF6,CDKN1A,PRKACA,PPP1R12A,PIK3CB,PIK3CD,GNG2,CDKN1B,OPN1SW,PRKAR1A,CAMK2G,PRKCB	0.007079458	SHC1,RRAS2,GRB2,ITPR2,PPP1R3C,CDKN1A,PIK3R2,ITPR1,CDKN1B,TUBB,PPP1R14B,PRKCB	0.173780083	PPP1R3C,LIMK2,TUBA3C,TUBB,SHC1,STMN1,CAMK2D,ARHGEF3,PPP1CA,PRKD1,CCNE2,GRB2,ARHGEF15,GNAI1,TUBA4A,ARHGEF17,ITPR1,PPP2R5A,CDK1,PLCB4,PRKCD,CDKN1A,PPP2R2B,PPP1R12A,PIK3CB,UHMK1,GNG2,CDKN1B,ADCY7,PRKCB	0.026915348	NA	#VALUE!
**Endothelial Cells in Rheumatoid Arthritis**	NFKB1	0.413999675	TLR1,IL18RAP,SOCS3,PLCB2,IL6,CREB5,FCGR1A,IL18R1,MYC,VEGFA,IL1R2,CAMK2D,PLCE1,PIK3CG,DKK2,TRAF5,LTBR,NFKBIB,PLCL1,ADAMTS4,IL8,SELE,C1S,TLR2,IL18,IL1RN,PLCG2,GNAO1,PIK3CD,FZD5,MAP2K3,PDGFD,PRSS35,CAMK2G,MAP2K6,IL1A,TCF4,ICAM1,JAK2,NFKB1,IL17RA,PDGFC,C5AR1,NFKBIA,NLK,CCL2,PPP3CB,CFB,AKT3,PIK3R2,MAPKAPK2,NOS2,MMP1 (includes EG:4312),PRKCA,MYD88,TNFRSF1A,IL15,IL1R1,TCF7L1,KLK4,ROCK1,FOS,PLCB4,RRAS2,PRKCI,FZD4,NFATC2,IL1B,PIK3CB,TCF7L2 (includes EG:6934),PRKCB,IRAK2	0.006606934	IL8,SFRP4,TNFRSF1A,IL6,FCGR1A,MYC,IL1R2,C5AR1,RRAS2,CCL2,IL1RN,GNAO1,PIK3R2,NOS2,TCF7L2 (includes EG:6934),PRKCB,ADAMTS4	0.261818301	TLR1,SFRP2,ICAM1,JAK2,IL6,CREB5,FCGR1A,VEGFA,PLCE1,NLK,CAMK2D,CCL2,CFB,DKK2,LTBR,PRKD1,TNFSF13B,IL8,SFRP4,VCAM1,MYD88,C1S,DAAM1,IL1R1,KLK4,TLR2,PLCB4,IL18,PRKCD,GNAO1,PIK3CB,IRAK4,TCF7L2 (includes EG:6934),RYK,PRSS35,PRKCB	0.299916252	FOS,SFRP2,C1S,FCGR1A	0.019498446
**Role of NFAT in Cardiac Hypertrophy**	HDAC4	0.272270131	MAP2K6,PLCB2,LIF,CAMK1D,ADCY4,RCAN2,GNB5,MAPK13,IL6,GNB4,PLCE1,GNG11,CAMK2D,GNB3,PPP3CB,ADCY5,PIK3CG,IGF1R,AKT3,PIK3R2,PLCL1,PRKCA,AKAP5,HDAC4,ITPR2,GRB2,GNAI1,ADCY6,ITPR1,HDAC5,PLCB4,PRKCI,RRAS2,PLCG2,PRKACA,TGFB3,PIK3CB,MEF2C,MAP2K3,PIK3CD,GNG2,SLC8A1,OPN1SW,PRKAR1A,CAMK2G,PRKCB	0.005128614	AKAP5,GRB2,ITPR2,RCAN2,MEF2A (includes EG:4205),ITPR1,IL6,SHC1,RRAS2,TGFB3,MEF2C,PIK3R2,PRKCB	0.128233058	TGFBR1,GRB2,GNAI1,IL6,ITPR1,SHC1,PLCB4,CAMK2D,PLCE1,PRKCD,TGFB3,PIK3CB,GNG2,SLC8A1,ADCY7,PRKD1,PRKCB	0.679203633	NA	#VALUE!
**Hepatic Fibrosis Cell Activation**	NFKB1	0.233345806	IL1A,LEP,ICAM1,SMAD3,ACTA2,MYL5,MYH11,IL6,NFKB1,BCL2,IL1R2,VEGFA,COL1A2,CCL2,TIMP1,IGF1R,PDGFRA,MMP1 (includes EG:4312),TIMP2,IL8,EDNRB,TNFRSF1A,FGFR2,MMP2,IFNGR1,IGFBP5,IL1R1,NFKB2,MYL6B,MYL9 (includes EG:10398),COL1A1,CD40,KDR,TGFB3,CD14,EDNRA,IL1B,A2M,IFNAR1,COL3A1	0.000316228	IL1R2,MYL9 (includes EG:10398),IL8,CCL2,TIMP1,TNFRSF1A,PDGFRA,TGFB3,IGFBP5,IL6,A2M,FGF1	0.066069345	MYH10,TGFBR1,ICAM1,IL6,MYH7B (includes EG:57644),BCL2,VEGFA,COL1A2,CCL2,EDN1,TIMP1,LBP,STAT1,MYH1,IL8,VCAM1,EDNRB,IGFBP5,MYH7,IL1R1,MYL1,FGF1,COL1A1,CD40,KDR,TGFB3,CD14,MYH9,IFNAR1,COL3A1	7.08E-05	COL1A2,COL1A1	0.083176377
**Atherosclerosis Signaling**	CXCR4,NFKB1	0.130918192	CCR3,IL1A,ICAM1,PLA2R1,ALOX12,IL6,NFKB1,PDGFC,PRDX6,COL1A2,LCAT,CCL2,PROCA1,MMP1 (includes EG:4312),IL8,SELE,CXCR4,NFKB2,F3,LAMB2,COL1A1,PLA2G4A,IL18,SELP,CD40,IL1RN,IL1B,PDGFD,COL3A1	0.005128614	LAMB2,IL8,CCL2,CXCR4,IL1RN,IL6	0.354813389	IL8,VCAM1,ICAM1,MSR1,PLA2G10,YWHAZ,IL6,F3,WISP2,LAMB2,COL1A2,COL1A1,IL18,CCL2,CD40,COL10A1,ITGA4,COL3A1	0.056234133	COL1A2,COL1A1	0.070794578
